# Ru(ii)-catalyzed regioselective (3 + 2)-annulation of anilines with allenes to access 2-vinylindoles

**DOI:** 10.1039/d5sc06303e

**Published:** 2025-12-16

**Authors:** Om Prakash Dash, Anurag Singh, Rahul K. Shukla, Chandra M. R. Volla

**Affiliations:** a Department of Chemistry, Indian Institute of Technology Bombay Powai Mumbai-400076 India Chandra.volla@chem.iitb.ac.in

## Abstract

Direct access to 2-vinylindole motifs from commercially available aniline precursors is an appealing yet challenging task. Conventional strategies often rely on pre-functionalized indoles or require harsh reaction conditions and so direct annulation of simple anilines for their synthesis remains an attractive alternative. Herein, we disclose a cost-effective Ru(ii)-catalyzed regioselective (3 + 2)-annulation of *N*-pyridyl anilines with allenyl carbinol acetates to access 2-vinylindoles at room temperature. The reaction proceeds through an unprecedented 3,2-migratory insertion of allenyl carbinol acetates to form a Ru-alkenyl intermediate, which is elusive so far in C–H activation. Catalyst screening revealed that the regioselectivity of migratory insertion of allene is governed by the nature of the metal-salt. While Ru(ii) favors the desired 3,2-insertion, Co(iii) promotes 2,1-insertion leading to a Co-σ-allyl intermediate. The synthetic process allows access to a large library of 2-vinylindole derivatives from commercially available anilines in good to moderate yields under mild conditions. Interestingly, bis-annulation with the substrates having di-amino functionalities was also successfully carried out to access highly conjugated bisindole architectures. Additionally, the versatility of the protocol was showcased by carrying out late-stage modification of various natural products, gram-scale synthesis, and further functionalization of the products along with photophysical studies of 2-vinylindole derivatives.

## Abstract 2

Ru(ii)-catalyzed highly regioselective annulation of *N*-pyridyl anilines with allenyl carbinol acetates was demonstrated at room temperature for the facile access to biologically relevant 2-vinylindole derivatives.

## Graphical abstract

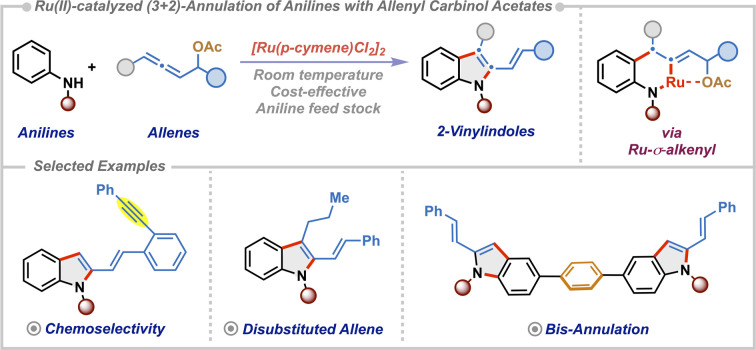

## Introduction

**Scheme 1:**
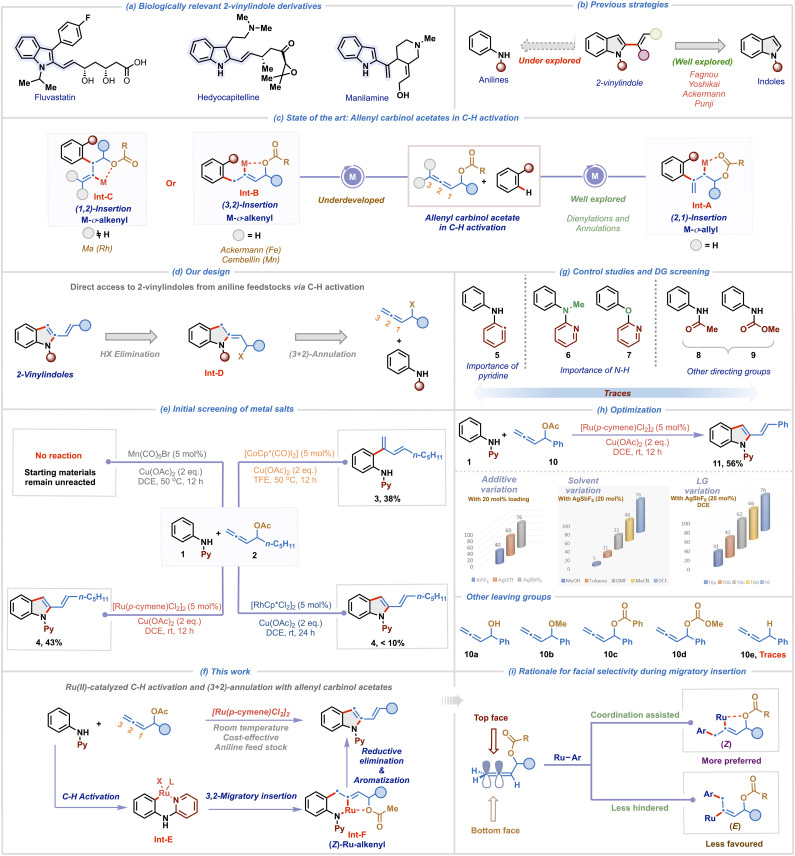
Overview of the work.

2-Vinylindoles and their derivatives represent a prominent class of heterocycles due to their widespread occurrence in natural products and pharmacologically relevant compounds (Scheme 1a).^1^ Furthermore, the alkenyl functionality at the C-2 position of indole serves as a versatile synthetic linchpin, enabling downstream diversification through a broad range of transformations, including cycloadditions, pericyclic reactions, ring-closing metathesis, and macrocyclization reactions.^2,3^ Consequently, the development of efficient and selective methodologies for the construction of 2-vinylindole frameworks has garnered significant attention.^4^ While traditional approaches such as the Fujiwara–Moritani reaction, hetero-Cope rearrangement, and Wittig-type olefinations are synthetically valuable, they often suffer from various limitations such as reliance on pre-functionalized starting materials, poor functional group tolerance, and the requirement for harsh reaction conditions, limiting their overall utility.^1*a*^ These challenges underscore the need for alternative strategies that offer operational simplicity and exhibit broad substrate scope under mild conditions. Transition metal-catalyzed C–H activation has emerged as a powerful synthetic platform for the construction of functionalized molecules with remarkable efficiency.^5^ This has prompted the exploration of C–H activation methodologies for the synthesis of 2-vinylindoles *via* C2-functionalization of indoles through either hydroindolation or cross-coupling approaches (Scheme 1b).^6–9^ Although elegant, these C–H activation methodologies rely on indole motifs as starting materials and entail direct vinylation to access 2-vinylindoles. In this context, a more flexible and strategic alternative lies in the *de novo* construction of 2-vinylindoles *via* annulation of readily available anilines. Developing a rapid and straightforward route to 2-vinylindoles from commercially available anilines through C–H activation offers a more sustainable and economical approach, significantly enhancing their utility, particularly for large-scale applications.

The unique structural features and rich reactivity profile of allenes have long intrigued synthetic chemists to investigate their potential in catalytic transformations for the rapid generation of molecular complexity.^10^ However, the presence of two orthogonal double bonds in allene presents significant challenges,^11^ specifically regioselectivity, positional control and chemoselectivity during migratory insertion, impeding their utilization in C–H activation processes.^12–14^ A promising solution to mitigate these issues involves the use of allenes having a tethered directing group for governing the migratory insertion. In this regard, allenyl carbinol acetates have recently emerged as exceptionally versatile and efficient coupling partners in transition metal-catalyzed C–H activation (Scheme 1c).^15–21^ The heteroatom at the α-position plays a pivotal role by coordinating with the metal center to direct selective 2,1-migratory insertion leading to an M–σ-allyl intermediate Int-A.^15–18^ Tailoring upon this concept, Glorius and co-workers in a pioneering study demonstrated Rh(iii)-catalyzed *ortho* C–H dienylation of benzamides employing allenyl carbinol carbonates.^15^ Subsequently, Ma,^16^ our group^17^ and others^18^ have engaged these allenes to develop a diverse range of C–H dienylation and annulation reactions. While the formation and reactivity of M–σ-allyl intermediates Int-A are now well understood, alternate migratory insertion modes leading to M–σ-alkenyl intermediates Int-B or Int-C*via* 3,2- or 1,2-insertion pathways remain comparatively underexplored, with only three reports existing in the literature to date.^19–21^ Ackermann group was the first to harness an Fe–alkenyl intermediate (aka Int-B) using iron-catalysts to access isoquinolinone derivatives *via* (4 + 2)-annulation of benzamides.^19^ More recently, Cembellín and co-workers demonstrated a Mn(i)-catalyzed C2-linear dienylation of indoles proceeding through a Mn–alkenyl intermediate (aka Int-B).^20^ In contrast to these 3,2-insertion pathways, Ma and co-workers achieved a reversal in insertion selectivity by leveraging substituents at the C3 position of allenyl carbinol acetates. Employing tri-substituted allenes, they facilitated 1,2-migratory insertion leading to the formation of a Rh–alkenyl intermediate (aka Int-C), enabling highly regioselective C2-allylation of indoles under Rh(iii) catalysis.^21^

## Results and discussion

Inspired by these seminal advancements and the broad relevance of 2-vinylindole scaffolds, we aimed to investigate the largely untapped reactivity of M–σ-alkenyl intermediate Int-B for synthesizing 2-vinylindoles from readily available anilines (Scheme 1d). We rationalized that the strategic incorporation of a removable directing group on the aniline substrate would facilitate regioselective C–H activation and promote chelation-assisted 3,2-migratory insertion of allenes. Subsequent reductive elimination was anticipated to generate Int-D, which upon HX elimination would furnish the desired 2-vinylindole scaffold. Given the critical influence of the metal center in dictating the migratory insertion pathway of allenes,^11^ we systematically investigated the reactivity of various metal salts to delineate their effect on the reaction outcome (Scheme 1e). Using readily available *N*-pyridyl aniline 1 and allenyl carbinol acetate 2 as model substrates, our initial trial with 5 mol% of [CoCp*(CO)I_2_] in TFE at 50 °C furnished the branched C–H dienylation product 3 in 38% yield. This is consistent with our earlier observation,^17*a*^ where the Co(iii)-catalyst promoted 2,1-migratory insertion during C8-H dienylation of quinoline-*N*-oxides. Interestingly, its heavier congener [RhCp*Cl_2_]_2_ (5 mol%) led exclusively to the formation of the desired 2-vinylindole 4, *albeit* in low yield and with no observable dienylation product. This suggests that Rh(iii) preferentially facilitates 3,2-migratory insertion, leading to the formation of Rh–alkenyl intermediate, underscoring the metal-dependent nature of carbometallation. In recent years, ruthenium catalysts, particularly [RuCl_2_(*p*-cymene)]_2_, have gained prominence as highly efficient and cost-effective alternatives to the more expensive Rh(iii) catalysts.^22^ Owing to their exceptional stability in air and moisture, we screened the reaction with Ru(ii)-catalysts, which resulted in the formation of 4 in an improved yield of 43%. Notably, Mn(CO)_5_Br, which was effective in promoting linear dienylation^20^*via* Mn–alkenyl pathway, was found to be ineffective under our reaction conditions. These findings collectively show the pivotal influence of the metal center in governing the regioselectivity of allene insertion with organometallic species.

Building upon these initial findings, herein we unveil a Ru(ii)-catalyzed regioselective (3 + 2)-annulation of anilines and allenyl carbinol acetates, enabling the efficient synthesis of 2-vinylindole derivatives at room-temperature (Scheme 1f). The transformation proceeds *via* initial C–H activation to generate a cyclometalated intermediate Int-E, followed by coordination assisted regioselective 3,2-migratory insertion of the allene generating a stable six-membered Ru-σ-alkenyl intermediate Int-F. Subsequent reductive elimination and aromatization *via* elimination of acetic acid yields the desired 2-vinylindoles. To elucidate the role of the directing group, a series of control experiments were performed (Scheme 1g). When diphenylamine 5 was subjected, instead of 1, to the standard reaction conditions with allenyl carbinol acetate 2, no product formation was observed, highlighting the importance of the pyridine moiety for the generation of Int-E. Additionally, two control reactions using *N*-methyl-*N*-phenylpyridin-2-amine 6 and 2-phenoxypyridine 7 with 2 also resulted in no product formation, indicating the critical role of the N–H group in coordinating with the Ru(ii)–alkenyl intermediate Int-F. Other directing groups, such as acetate (acetanilide 8) and carbonate (phenylcarbamate 9) also failed to yield 2-vinylindoles, showing the unique and indispensable role of the pyridine-based directing group in enabling the transformation. With a comprehensive understanding of the role of the directing group, we aimed to improve the yield of the desired transformation (Scheme 1h). Substituting the allene 2 with 10 afforded a slight enhancement in yield of the corresponding product 11 to 56%. Addition of AgSbF_6_ (20 mol%) significantly enhanced the reaction efficiency further, resulting in 76% yield of 11. In contrast, other additives, such as KPF_6_ and AgOTf were found to be ineffective. This suggests that AgSbF_6_ plays a crucial role in enhancing the reactivity of the Ru(ii)-catalyst by abstracting the chloride anion. We then screened various solvents using 20 mol% AgSbF_6_ as an additive to further improve the yield. Notably, the reaction is sluggish in other solvents such as MeOH, toluene, DMF and CH_3_CN highlighting the key role of DCE as the solvent. In addition, the impact of various leaving groups on the allene substrate was also investigated using AgSbF_6_ (20 mol%) in DCE. Leaving groups such as hydroxy 10a, methoxy 10b, benzoate 10c, and methyl carbonate 10d were found to be less effective than acetate 10 in promoting the annulation. Intriguingly, the benzyl-substituted allene 10e completely failed to deliver the desired product 11, which is likely due to the absence of a coordinating heteroatom at the α-position further ratifying the importance of chelation for the formation of Int-F. These observations corroborate the crucial role of both the electronic nature and coordination ability of the leaving group in facilitating the transformation. The success of this transformation hinges on the selective formation of the (*Z*)-Ru-alkenyl intermediate Int-F, which can be rationalized by preferential top-face insertion of the allene into the aryl-ruthenium species (Scheme 1i). As the π-system of allene is oriented perpendicular to the plane, two insertion pathways (top and bottom) with the terminal double bond of allene are feasible. Although the bottom approach offers less steric hindrance, the top-face insertion is favored due to the stabilizing coordination between the oxygen atom and the ruthenium center, thereby selectively promoting the formation of the (*Z*)-configured Ru–alkenyl species Int-F.

After rigorous optimization studies by varying different reaction parameters such as solvent, base, oxidant and catalyst (see the SI), the optimal reaction conditions were found to be [Ru(*p*-cymene)Cl_2_]_2_ (5 mol%), AgSbF_6_ (20 mol%) and Cu(OAc)_2_ (2 equiv.) in dry DCE at room temperature for 12 h, affording the desired product 11 in 76% yield (isolated yield of 74%) (Table 1, entry 1). Cu(OAc)_2_ proved to be the best oxidant for this transformation as other oxidants such as Ag_2_CO_3_, AgOAc and BQ were found to be ineffective in furnishing the desired product (entry 2). Attempts to enhance the yield through the addition of external bases such as NaOAc, CsOAc, Na_2_CO_3_, and Cs_2_CO_3_ produced 11 only in reduced yields (entry 3). Increasing the temperature to 50 °C resulted in a diminished yield (58%), indicating that room temperature is the optimal temperature for the protocol (entry 4). When the reaction was performed with 1 eq. of Cu(OAc)_2_ under an O_2_ balloon, 64% yield of 3 was observed (entry 5). As expected, no product was observed in the absence of either Cu(OAc)_2_ or the Ru(ii) catalyst (entries 6 and 7). Other metal catalysts like Pd(OAc)_2_ and NiCl_2_ were ineffective, with no detectable formation of 2-vinylindole 11 in the crude ^1^H-NMR of the reaction mixture (entries 8 and 9), indicating the crucial role of [Ru(*p*-cymene)Cl_2_]_2_.

**Table 1:** Optimization of reaction conditions

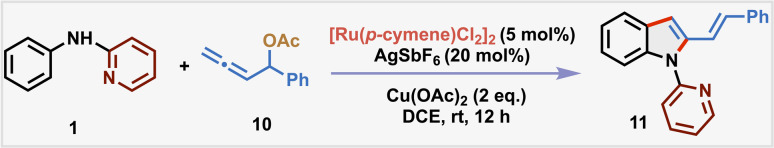		
Entry	Deviation from standard conditions	Yield^a^ (%)
1^a^	None	76^b^ (74)^c^
2	AgOAc, Ag_2_CO_3_, BQ instead of Cu(OAc)_2_	—
3^d^	NaOAC, CsOAc, Na_2_CO_3_, Cs_2_CO_3_	<72
4	*T* = 50 °C, 10 h	58
5	1 eq. Cu(OAc)_2_ under O_2_ balloon	64
6	Without Cu(OAc)_2_	—
7	Without [Ru(*p*-cymene)Cl_2_]_2_	—
8^e^	Pd(OAc)_2_ instead of [Ru(*p*-cymene)Cl_2_]_2_	—
9^e^	NiCl_2_ instead of [Ru(*p*-cymene)Cl_2_]_2_	—

a Reaction conditions: 1 (0.15 mmol), 10 (0.10 mmol), [Ru(*p*-cymene) Cl_2_]_2_ (5 mol%), AgSbF_6_ (20 mol%) and Cu(OAc)_2_ (0.20 mmol), DCE (1.0 mL) at rt for 12 h.

b Yield is calculated based on ^1^H NMR of the crude reaction mixture using 1,3,5-trimethoxybenzene as an internal standard.

c Yield in parentheses refers to isolated yield.

d As an additive (1 eq.) along with standard conditions.

e 10 mol% catalyst loading.

**Scheme 2:**
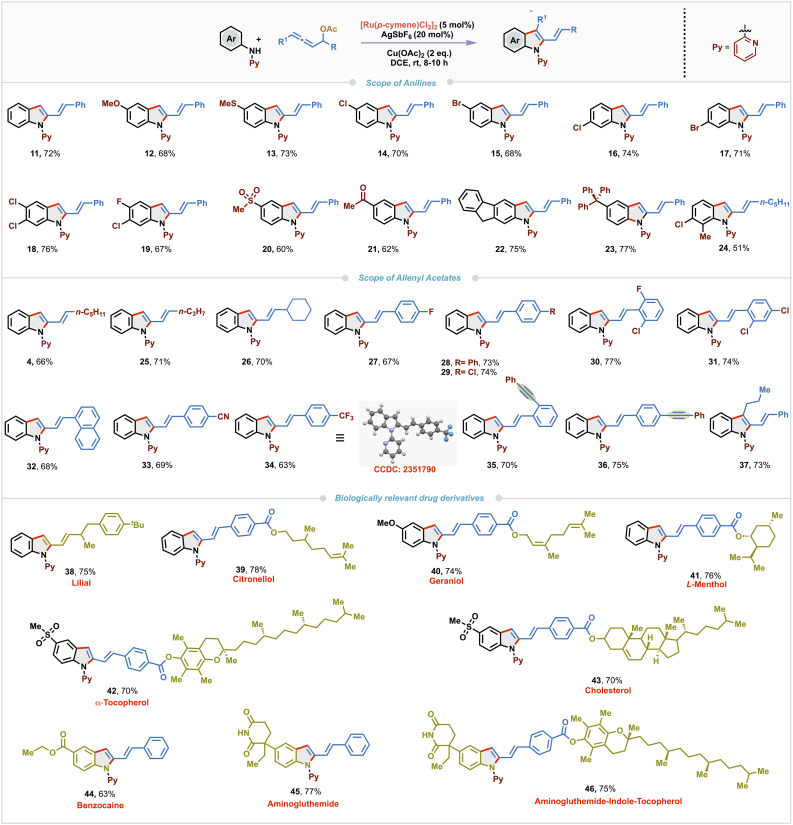
Substrate scope of 2-vinylindoles.

With the optimized conditions in hand, we explored the substrate scope of the Ru(ii)-catalyzed regioselective (3 + 2)-annulation using a variety of *N*-aryl-2-aminopyridine derivatives with allenyl acetate 10 (Scheme 2). Pleasingly, both electron-donating and -withdrawing substituents such as methoxy, sulfide, chloro and bromo at the *para*-position of the aniline ring were well tolerated under the standard reaction conditions, delivering the corresponding annulated products 12–15 in moderate to good yields (68–73%). Remarkably, *meta*-substituted *N*-aryl-2-aminopyridines afforded the corresponding 2-vinyldindoles 16 and 17 by selective activation of the less sterically hindered C–H bond in good yields (74% and 71% respectively). Furthermore, dihalo-substituted *N-*aryl-2-aminopyridines furnished 18 and 19 in comparable yields (76% and 67%, respectively). Substrates bearing strongly electron-withdrawing groups, such as sulfonyl (–SO_2_Me) and acetyl (–COMe) at the *para*-position produced 20 and 21 in slightly lower yields (60% and 62%). Interestingly, substrates derived from 2-aminofluorene and 4-tritylaniline also underwent the annulation to deliver 22 and 23 in amenable yields (75% and 77%, respectively). *Ortho*-substituted aniline was found to be slightly less efficient in this protocol and gave product 24 in 51% yield. To further explore the synthetic versatility of this protocol, various allenyl carbinol acetates were tested. Allenes bearing aliphatic substituents such as *n*-pentyl, *n*-propyl and cyclohexyl fared well and provided the corresponding 2-vinylindoles 4, 25 and 26 in 66–71% yields. Both electron-rich and-deficient aryl substituted allenes were compatible, yielding the products 27–34 in 63–77% yields. Single-crystal X-ray diffraction analysis of 34 unambiguously confirmed the structure of the 2-vinylindole derivatives. To examine the chemoselectivity of the (3 + 2)-annulation, allenyl acetates having both allene and alkyne functionalities were tested. Notably, annulation occurred selectively with the allene moiety and resulted in 35 and 36 (70% and 75%) leaving the alkyne untouched. 1,3-Disubstituted allene also worked well to afford C-3 substituted 2-vinylindole 37 in 73% yield. Given the biological relevance of 2-vinylindole motifs, we carried out late-stage functionalization using allene derived from a naturally occurring aldehyde such as lilial; the reaction underwent smoothly to furnish 38 in 75% yield. Similarly, allenes tethered with naturally derived alcohols such as citronellol, geraniol, (*L*)-menthol, α-tocopherol, and cholesterol were also found to be amenable substrates and the corresponding 2-vinylindole derivatives 39–43 were isolated in 70–78% yields. Biologically significant aniline derivatives such as benzocaine and aminoglutethimide also reacted smoothly with allenyl acetate 10 to provide 44 and 45 in 63% and 77% yields respectively. A modular strategy reacting an aminoglutethimide-derived *N*-aryl-2-aminopyridine with a tocopherol-derived allene enabled the synthesis of a complex 2-vinylindole conjugate 46 in 75% yield.

**Scheme 3:**
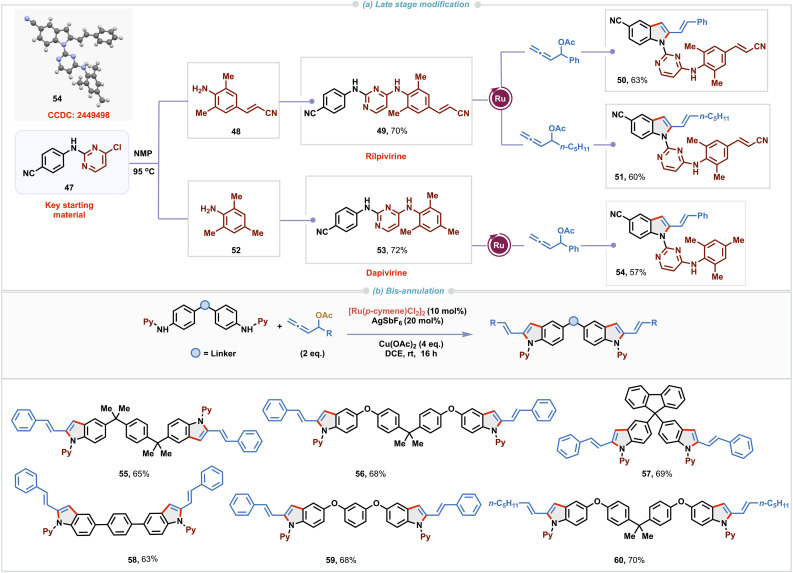
Late-stage functionalization and substrate scope of bis-annulation.

Moreover, our methodology proved effective in complex settings such as late-stage modification of non-nucleoside reverse transcriptase inhibitors^23^ rilpivirine 49 and dapivirine 53 (Scheme 3a). These derivatives were prepared from the key starting material 47 and corresponding anilines 48 or 52 by heating in NMP at 95 °C. Excellent regioselectivity was observed with these substrates containing multiple potential reaction sites under standard reaction conditions with allenyl acetates having aryl or alkyl substituents, delivering corresponding functionalized 2-vinylindole derivatives 50, 51 and 54. Single-crystal X-ray diffraction analysis of 54 unambiguously confirmed the structure of the 2-vinylindole derivative derived from dapivirine. Bis-indole derivatives exhibit a broad spectrum of biological activities including antiviral, analgesic, antifungal, and anti-inflammatory activities.^24^ As a result, there is growing interest in developing cost-effective, efficient and straightforward methods for their synthesis. Captivatingly, our Ru(ii)-catalyzed (3 + 2)-annulation was found to be suitable for enabling one pot bis-cyclization with di-amino derivatives employing an excess of allenyl acetate (Scheme 3b). Double (3 + 2) annulation proceeded under slightly modified reaction conditions to access bis-vinylindole derivatives 55–60 in 63–70% yields).

**Scheme 4:**
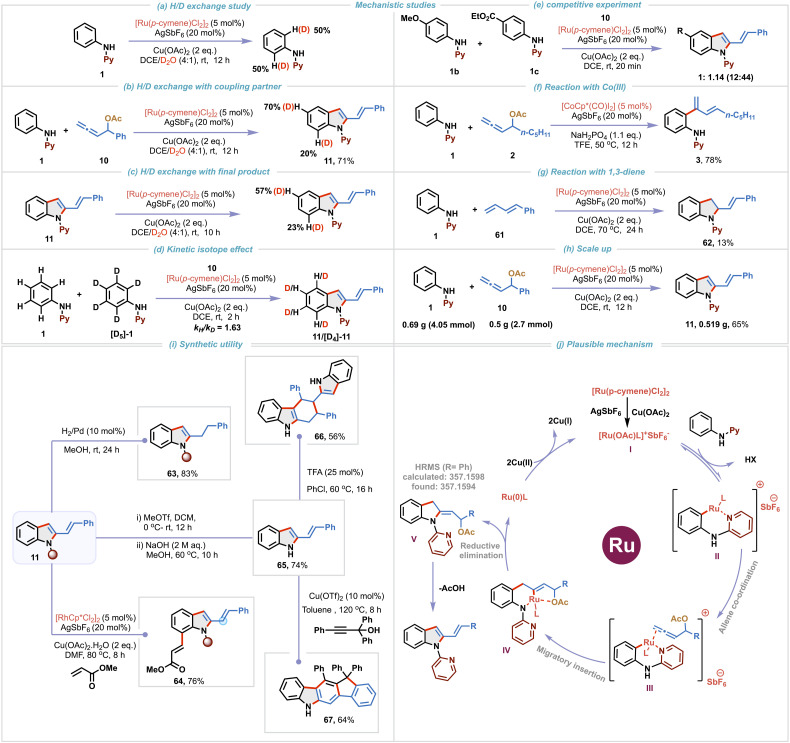
Mechanistic studies, scale up, further functionalization and the proposed mechanism.

After exploring the substrate scope of the transformation, various deuterium exchange and competitive experiments were conducted in order to gain mechanistic insights (Scheme 4). A deuterium exchange experiment of 1 in the absence of allenyl acetate, using a 4 : 1 mixture of DCE and D_2_O resulted in 50% deuterium incorporation at the *ortho* C–H position of the phenyl ring (Scheme 4a). When the same experiment was carried out in the presence of allenyl acetate 10, 20% deuterium incorporation at the C-7 position of the indole was observed along with 70% deuterium incorporation at the C-5 position (Scheme 4b). These observations, reveal that C–H bond cleavage is reversible in nature and indicate that C–H activation might be proceeding *via* the typical concerted metalation–deprotonation (CMD) mechanism. To gain more understanding of the deuteration at the C-5 position, compound 11 was subjected to the standard reaction conditions in the presence of a D_2_O/DCE mixture (Scheme 4c). Interestingly, 57% deuterium incorporation at the C-5 position was observed suggesting that C-5 deuteration occurs after annulation. To further probe the C–H activation mechanism, a kinetic isotope effect (KIE) study employing a 1 : 1 mixture of 1/[D_5_]-1 with 10 was conducted, which resulted in a competitive isotopic value (*k*_H_/*k*_D_) of 1.63, suggesting that C–H bond cleavage might not be involved in the rate-limiting step (Scheme 4d). An intermolecular competitive experiment between 4-methoxy and 4-ester substituted *N*-aryl-2-aminopyridines 1b and 1c with allene 10 resulted in a product ratio of 1 : 1.14 for 12 : 44, implying that the annulation proceeds preferentially with electron deficient *N*-aryl-2-aminopyridine, shedding light on the C–H activation step (Scheme 4e). In line with our initial observation (Scheme 1e), use of 5 mol% [CoCp*(CO)I_2_] provided the C–H dienylation product 3 selectively in 78% yield (Scheme 4f). When we replaced allene 11 with 1,3-diene 61 as a coupling partner, 2-vinylindoline 62 was obtained in 13% yield instead of the desired 2-vinylindoles, clearly demonstrating the potential of our protocol for accessing 2-vinylindole derivatives (Scheme 4g). A scale-up reaction using 0.69 g of 1 (4.05 mmol) and 0.5 g of 10 (2.7 mmol) under optimized conditions delivered 0.519 g of 2-vinylindole 11 in 65% yield, demonstrating the scalability of the protocol (Scheme 4h). Next, to demonstrate the synthetic utility of 2-vinylindoles, further functionalization of these motifs was explored (Scheme 4i). Pd-catalyzed hydrogenation of 2-vinylindole 11 gave 2-alkylindole 63 in 83% yield. Regioselective C7 C–H functionalization of 11 using a Rh(iii)-catalyst and methyl acrylate afforded 64 in 76% yield. Removal of the pyridine directing group under basic conditions yielded 65 in 74% yield. As 2-vinylindole motifs are useful diene precursors,^2^ acid-catalyzed [4 + 2] self-dimerization of 65 furnished 66 in 56% yield and Cu(ii)-catalyzed cyclization with propagyl alcohol provided polycyclic scaffold 67 in 64% yield.

Finally, based on the preliminary mechanistic studies, we propose the following reaction mechanism (Scheme 4j). Active cationic Ru(ii)-catalytic species I is generated *via* halide abstraction from [Ru(*p*-cymene)Cl_2_]_2_ in the presence of AgSbF_6_ and Cu(OAc)_2_. Directed *ortho*-metalation with *N*-aryl-2-aminopyridine leads to the formation of the key six-membered 16 electron-ruthenacycle intermediate II. Coordination of allene followed by regioselective 3,2-migratory insertion gives intermediate IV. To decipher the contribution of oxygen coordination, we computationally evaluated the free-energies of the two conformers of *Z*-intermediate IV*i.e.* with and without coordination to oxygen and found that the coordinated intermediate is ∼6 kcal mol^−1^ lower in energy in both triplet and singlet multiplicities, clearly indicating the additional stabilization due to coordination of oxygen (see the SI for more details). Reductive elimination forms intermediate V and reduced Ru-species. Our efforts to isolate intermediate V met with no success. However, its formation has been confirmed by HRMS analysis of the crude reaction mixture. Cu(ii) oxidizes Ru(0) to regenerate the active Ru(ii)-catalyst. Finally, aromatization of intermediate V occurs *via* elimination of acetic acid to deliver the desired 2-vinylindole derivative.

**Scheme 5:**
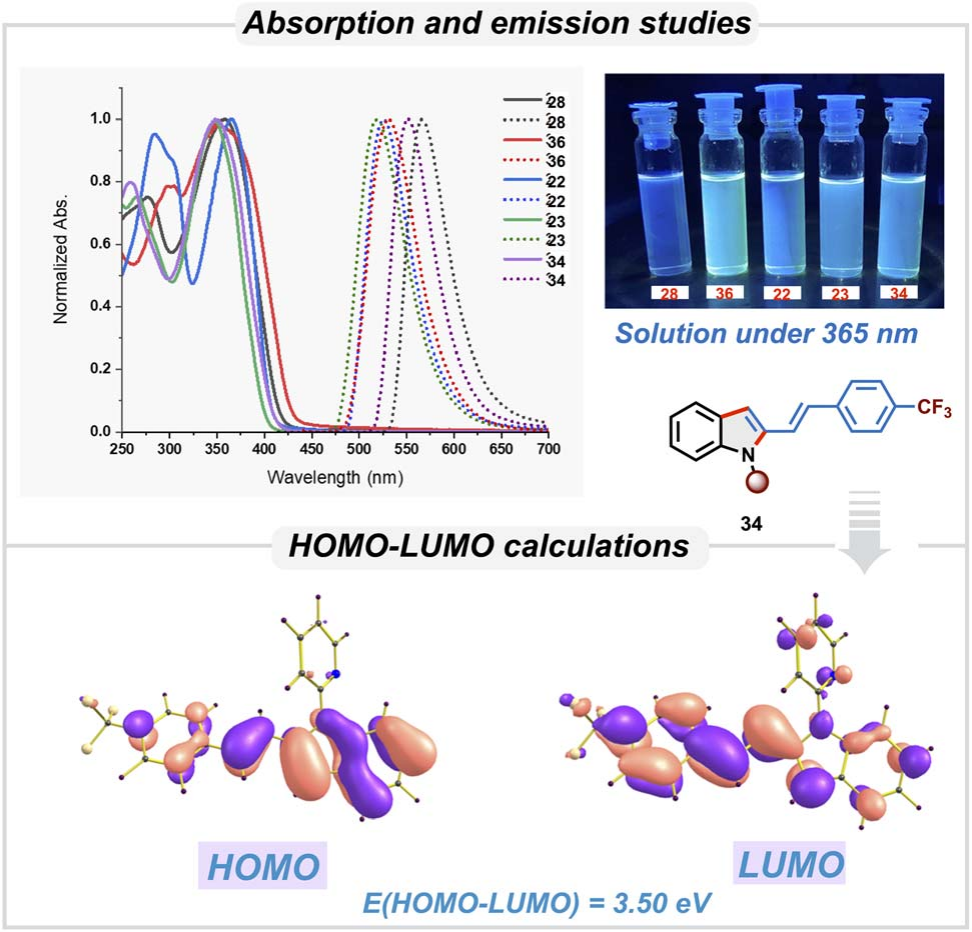
Photophysical studies.

We then studied the photophysical properties of 2-vinylindole derivatives having different substituents in order to investigate their applicability for optoelectronic applications (Scheme 5). Compounds 22, 23, 28, 34, and 36 showed significant absorption in the ultraviolet region (350–400 nm). These absorption bands were attributed to π–π* transitions of the conjugated systems. Substituent variations resulted in a slight change in the absorption maxima. The emission spectra of these derivatives showed maxima in the visible region around 500–600 nm. Notably large Stokes shifts were observed: 161 nm for 22, 171 nm for 23, and 208 nm for 28, 203 nm for 34 and 177 nm for 36. With the crystal structure of 34, the electronic properties were studied using time-dependent density functional theory (TD-DFT) without further optimizing the structure. The electron distribution of the HOMO and LUMO of 34 was found to be as shown in Scheme 5. The energy of the HOMO was found to be −5.35 eV, while that of the LUMO was −1.84 eV. The HOMO–LUMO gap was 3.5 eV, which correlates well with observed value of 3.49 eV from absorption spectra. Based on these studies, these derivatives are expected to be useful in fluorescent probes and optoelectronic devices.

## Conclusions

In conclusion, we have developed a highly efficient and rational strategy for the construction of 2-vinylindole frameworks *via* Ru(ii)-catalyzed (3 + 2)-annulation of feedstock anilines and allenyl carbinol acetates under mild conditions at room temperature. To the best of our knowledge, this represents the first example of utilizing aniline substrates with allenes to access 2-vinylindole scaffolds. The reaction proceeds *via* a Ru-σ-alkenyl intermediate which has been elusive so far with allenyl acetates. Systematic investigation of various metal-salts revealed that the regioselectivity of migratory insertion of allenes with organometallic intermediate depends on the metal-catalyst (Ru(ii) *vs.* Co(iii)) providing valuable mechanistic insights into the carbometallation pathway. This protocol demonstrates broad substrate scope, exhibiting excellent compatibility with a diverse range of functional groups. Furthermore, the practical utility was showcased through late-stage functionalization of various natural products, gram scale synthesis and photophysical studies. Overall, this work provides a new avenue for the construction of 2-vinylindole frameworks *via* C–H annulation of anilines with allenyl carbinol acetates.

## Author contributions

O. P. D. and A. S. conducted all the experiments and characterized the new compounds. O. P. D., R. K. S and C. M. R. V. designed the experiments. O. P. D. and C. M. R. V. wrote the manuscript. C. M. R. V. directed the research.

## Conflicts of interest

There are no conflicts to declare.

## Supplementary Material

SC-OLF-D5SC06303E-s001

SC-OLF-D5SC06303E-s002

## Data Availability

CCDC 2351790 and 2449498 contain the supplementary crystallographic data for this paper.^25*a*,*b*^ The data underlying this study are available in the published article and its supporting information (SI). Supplementary information: general information, experimental procedures for the synthesis of starting and final compounds, spectroscopic characterization data, NMR spectra for all the obtained compounds and X-ray crystallographic analysis data for compounds 34 and 54. See DOI: https://doi.org/10.1039/d5sc06303e.
