# Supplementary material for: Ru(ii)-catalyzed regioselective (3 + 2)-annulation of anilines with allenes to access 2-vinylindoles
Source: Chem Sci. 2025 Dec 16;17(12):5944–52. doi: 10.1039/d5sc06303e (PMC12862352; doi:10.1039/d5sc06303e)
Supplement: SC-017-D5SC06303E-s001 [file SC-017-D5SC06303E-s001.pdf]

Supplementary Information (SI) for Chemical Science.
This journal is © The Royal Society of Chemistry 2025

Ru(II)-Catalyzed Regioselective (3+2)-Annulation of Anilines with Allenes to Access 2-Vinylindoles

Om Prakash Dash, Anurag Singh, Rahul K. Shukla, Chandra M. R. Volla*

Department of Chemistry, Indian Institute of Technology Bombay

Powai, Mumbai-400076, India.

E–mail: chandra.volla@chem.iitb.ac.in

Supporting Information

Table of contents

1	**General considerations and reagent information**	**S2**
2	**General procedures for the synthesis of starting materials**	**S(2-4)**
3	**Optimization of the reaction conditions**	**S5**
4	**Experimental procedures**	**S(6-8)**
5	**Mechanistic studies**	**S(8-14)**
6	**Mechanism**	**S15**
7	**Control studies**	**S(16-17)**
8	**X-ray Crystallography data for the compound**	**S(17-20)**
9	**Functionalization**	**S(20-22)**
10	**Photophysical Studies**	**S(22-25)**
11	**DFT studies**	**S(25-30)**
12	**Computational details**	**S(30-44)**
13	**References**	**S(44-45)**
14	**Spectroscopy data for new starting materials**	**S(45-48)**
15	**Spectroscopic data of final products**	**S(48-74)**
16	**^{1}H and ^{13}C NMR spectra of the compounds**	**S(74-135)**

1. General considerations.

All reactions were carried out under air in screw cap reaction tubes. Unless otherwise noted, all the chemicals were purchased from commercial suppliers and used as received. Reactions were monitored using thin-layer chromatography (SiO_2). A gradient elution using petroleum ether and ethyl acetate was performed based on Merck aluminum TLC sheets (silica gel $60F_{254}$). TLC plates were visualized with UV light (254 nm) or $KMnO_4$ stain. For column chromatography, silica gel (100–200 mesh) from Finar Co. was used. All isolated compounds are characterized by ^{1}H NMR, ^{13}C NMR spectroscopy. In addition, all the compounds are further characterized by HRMS. HRMS were recorded with Bruker MaXis impact mass spectrometer using ESI-TOF techniques. Copies of ^{1}H NMR and ^{13}C NMR can be found in the supporting information. Nuclear magnetic resonance spectra were recorded either on a Bruker 500 or a 400 MHz instrument. All ^{1}H NMR experiments are reported in units, parts per million (ppm), and was measured relative to the signals for residual chloroform (7.26 ppm) in the deuterated solvent, unless otherwise stated. All ^{13}C NMR spectra was reported in ppm relative to deuterochloroform (77.16 ppm) unless otherwise stated, and all was obtained with ^{1}H decoupling.

2. General procedure for the synthesis of starting materials.

(a) General procedure for the synthesis of pyridinyl arylamines [1-5]:

A 25 mL Schlenk tube with a magnetic stir bar was charged with aniline (1.4 g, 15 mmol), 2-bromopyridine (2.4 g, 15 mmol). The reaction mixture was stirred at 160 ^oC (oil bath) for 7 h under an atmosphere of argon. Upon completion, saturated $NaHCO_3$ was added, and the mixture was extracted with EtOAc (3×15 mL). The combined organic phase was washed with brine and dried over Na_2SO_4. The solid was filtered off and the filtrate was evaporated in vacuum. The crude product was purified by flash column chromatography (*n*-hexanes/EtOAc) to give *N*-phenylpyridin-2-amine.

(b) General procedure for the synthesis of bis pyridinyl arylamines.

A 25 mL Schlenk tube with a magnetic stir bar was charged with aniline (1.4 g, 15 mmol), 2-bromopyridine (4.8 g, 30 mmol). The reaction mixture was stirred at 160 ^oC (oil bath) for 15 h under an atmosphere of argon. Upon completion, saturated $NaHCO_3$ was added, and the mixture was extracted with EtOAc (3×15 mL). The combined organic phase was washed with brine and dried over Na_2SO_4. The solid was filtered off and the filtrate was evaporated in vacuum. The crude product was purified by flash column chromatography (*n*-hexanes/EtOAc) to give bis *N*-phenylpyridin-2-amine.

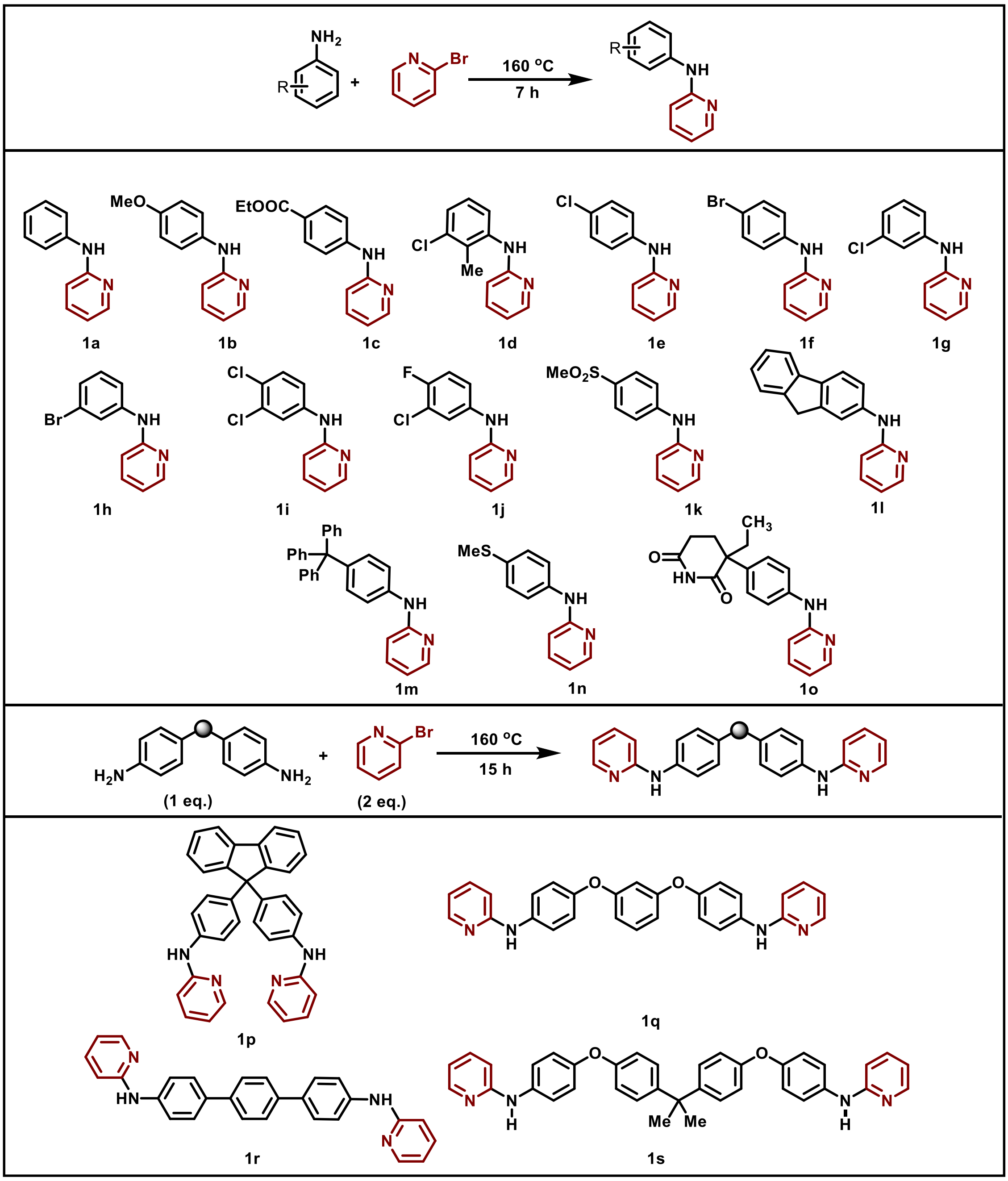

(c) General procedure for the synthesis of allenyl carbinol acetate [6-10]

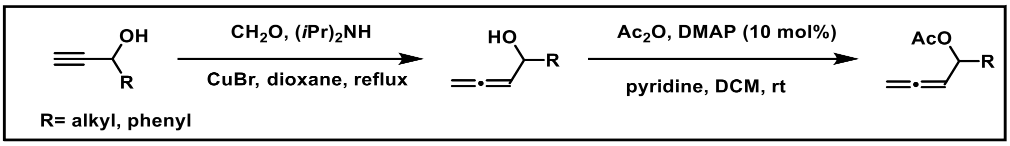

Step 1: To a two-necked round bottom-flask equipped with a magnetic stir bar were added under argon propargylic alcohol (10 mmol), 15 mL dioxane, 0.72 g of cuprous bromide, 0.74 g of paraformaldehyde, and 1.85 g of diisopropylamine. The reaction mixture was refluxed for 2 h and then cooled to room temperature. To mixture was filtered through a Celite plug. The filtrate is diluted with water followed by diethyl ether and acidified with 6 N HCl to pH

2. The organic layer was separated and the aqueous phase was extracted with diethyl ether for additional two times. The organic phase was then washed with saturated $NaHCO_3$, brine and dried over $MgSO_4$. After filtration and evaporation under reduced pressure, the residue was subjected directly for the next step.

Step 2: To a round bottom-flask equipped with a magnetic stir bar were added under argon allenyl carbinol, DMAP (122 mg, 1.0 mmol, 0.2 equiv), pyridine (790 mg, 10 mmol, 2.0 equiv)and dichloromethane (0.3 M). The mixture was cooled to 0 °C and the chloro methyl formate (708.8 mg, 7.5 mmol, 1.5 equiv) was slowly added. The reaction was allowed to stir at room temperature until completion (typically 1 – 16 h). The mixture was diluted with dichloromethane and washed successively with 1 N HCl, saturated $NaHCO_3$, and brine. The organic phase was dried over $MgSO_4$, filtered and evaporated under reduced pressure. The residue was purified by flash column chromatography to yield the desired product.

(d) Synthesis of rilpivirine and dapivirine drug

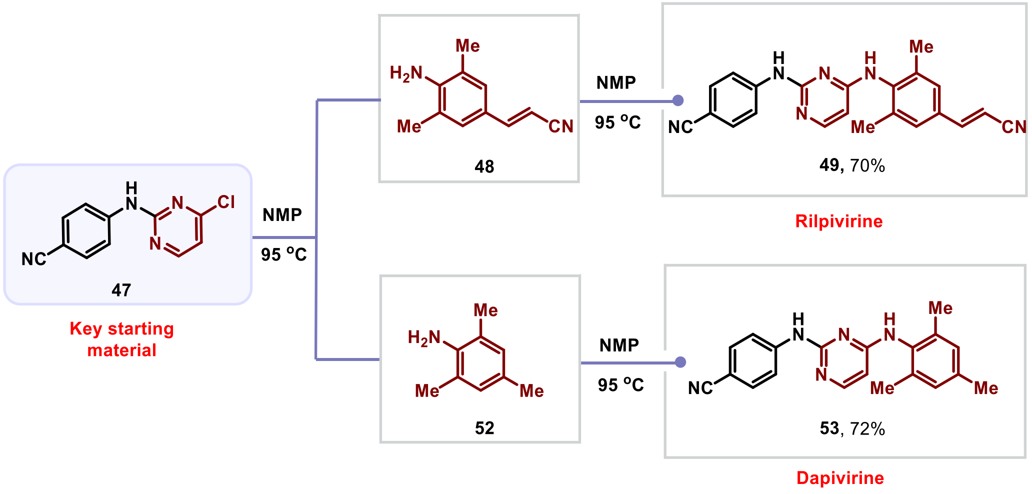

Synthesis of the target compound rilpivirine[15] (**49**) and dapivirine[16] (**53**) was achieved by allowing the reaction between (2E)-3-(4-amino-3,5-dimethylphenyl) prop-2-enenitrile hydrochloride **47** (1.2 equiv.) as a key starting material for both drug derivative and 4-[(4-chloropyrimidin-2-yl) amino] benzonitrile **48** (1 equiv.) or 2,4,6-trimethyl aniline **52** (1 equiv.) in NMP solvent at 95 °C for 24 h to yield 70% and 72% respectively as white solid.

3. Optimization

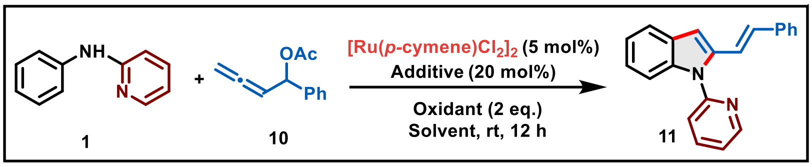

Entry [a]	Solvent	Oxidant	Base	Additive	Yield (%) [b]
1	TFE	Cu $(OAc)_2$	-	-	n.r
2	MeOH	Cu $(OAc)_2$	-	-	5
3	Toluene	Cu $(OAc)_2$	-	-	11
4	1,4-dioxane	Cu $(OAc)_2$	-	-	n.r
5	CH_3CN	Cu $(OAc)_2$	-	-	48
6	DMF	Cu $(OAc)_2$	-	-	32
7	DCE	Cu $(OAc)_2$	-	-	56
8	DCE	BQ	-		n.r
9	DCE	Ag_2CO_3	-		n.r
10	DCE	AgOAc	-		n.r
11	DCE	Cu $(OAc)_2$	-	$AgSbF_6$	76 (74)[e]
12[c]	DCE	Cu $(OAc)_2$	-	$AgSbF_6$	64
13	DCE	Cu $(OAc)_2$	-	AgOTf	60
14	DCE	Cu $(OAc)_2$	-	KPF_6	40
15[d]	DCE	Cu $(OAc)_2$	NaOAc	$AgSbF_6$	68
16[d]	DCE	Cu $(OAc)_2$	Na_2CO_3	$AgSbF_6$	72
17[d]	DCE	Cu $(OAc)_2$	CsOAc	$AgSbF_6$	64
18[d]	DCE	Cu $(OAc)_2$	Cs_2CO_3	$AgSbF_6$	62
19[d]	DCE	Cu $(OAc)_2$	K_2CO_3	$AgSbF_6$	67
20[f]	DCE	Cu $(OAc)_2$	-	$AgSbF_6$	n.r

[a] 1a (0.15 mmol), 2a (0.10 mmol), [Ru(*p*-cymene) $Cl_2]_2$ (5 mol %), solvent (1.0 mL) at rt for 12 h. [b] Yield is calculated based on ^{1}H NMR of crude reaction mixture using 1,3,5-trimethoxybenzene as internal standard. [c] 1eq. of $Cu(OAc)_2$ under O_2 balloon [d] External base additive (1 equiv.). [e] Yield in parentheses refers to isolated yield. [f] $Pd(OAc)_2$ (10 mol%) instead of Ru(*p*-cymene) $Cl_2]_2$ (5 mol %).

4. Experimental procedures

(a) General procedure for the synthesis of 2-vinyl indole derivatives.

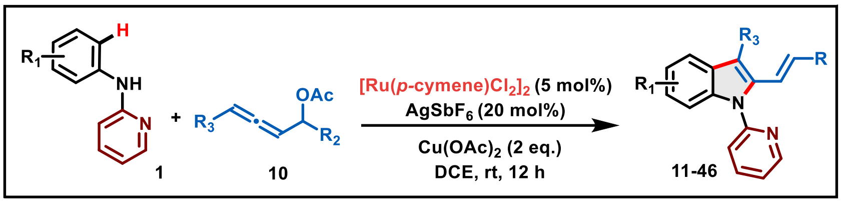

In an oven-dried reaction tube, charged with magnetic stir-bar, [*Ru(p-cymene)Cl*$_2$]$_2$ (5 mol%), and $Cu(OAc)_2$ (0.4 mmol), $AgSbF_6$ (20 mol %)($AgSbF_6$ was taken inside the glove box), N-aryl aminopyridines **1** (0.30 mmol,1.5 equiv) and allenyl acetates **10** (0.2 mmol, 1.equiv) were added followed by addition of 2 ml of 1,2-dichloroethane via syringe. The reaction mixture was allowed to stir at room temperature for 12 h. Then, the mixture was cooled and diluted with CH_2Cl_2 (10 mL). The mixture was filtered through a Celite pad and washed with CH_2Cl_2 (3 × 10 mL). The filtrate was concentrated under reduced pressure. The residue was purified by silica gel column chromatography using hexane/ethyl acetate as eluent to afford the desired pure product **11** to **46**.

(b) General procedure for late stage modification.

(i) Synthesis of 50:

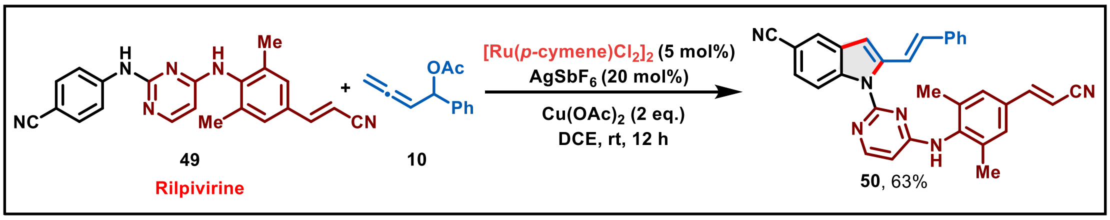

In an oven-dried reaction tube, charged with magnetic stir-bar, [*Ru(p-cymene)Cl*$_2$]$_2$ (5 mol%), and $Cu(OAc)_2$ (2 equiv.), $AgSbF_6$ (20 mol %)($AgSbF_6$ was taken inside the glove box), Rilpivirine **49** (0.15 mmol,1.5 equiv) and allenyl carbinol acetates **10** (0.1 mmol, 1.equiv) were added followed by addition of 1 ml of 1,2-dichloroethane via syringe. The reaction mixture was allowed to stir at room temperature for 12 h. Then, the mixture was cooled and diluted with CH_2Cl_2 (10 mL). The mixture was filtered through a Celite pad and washed with CH_2Cl_2 (3 × 10 mL). The filtrate was concentrated under reduced pressure. The residue was purified by silica gel column chromatography using hexane/ethyl acetate as eluent to afford the desired pure product as sticky solid **50** in (31 mg, 63% yield).

(ii) Synthesis of 51:

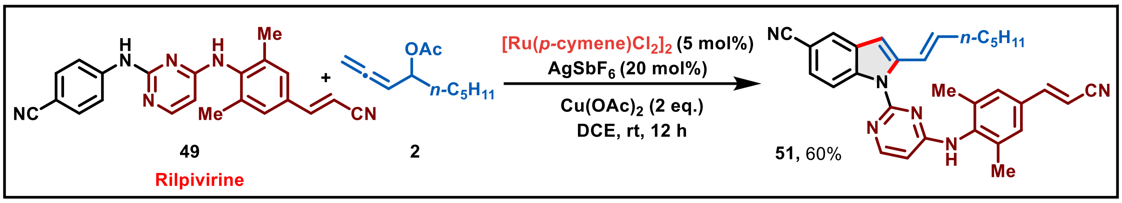

In an oven-dried reaction tube, charged with magnetic stir-bar, [*Ru(p-cymene)Cl*$_2$]$_2$ (5 mol%), and Cu(OAc)$_2$ (2 equiv.), $AgSbF_6$ (20 mol %)($AgSbF_6$ was taken inside the glove box), Rilpivirine **49** (0.15 mmol,1.5 equiv) and allenyl carbinol acetates **2** (0.1 mmol, 1.equiv) were added followed by addition of 1 ml of 1,2-dichloroethane via syringe. The reaction mixture was allowed to stir at room temperature for 12 h. Then, the mixture was cooled and diluted with CH_2Cl_2 (10 mL). The mixture was filtered through a Celite pad and washed with CH_2Cl_2 (3 × 10 mL). The filtrate was concentrated under reduced pressure. The residue was purified by silica gel column chromatography using hexane/ethyl acetate as eluent to afford the desired pure product as light yellow liquid **51** (29.2 mg**,** 60% yield).

(iii) Synthesis of 53:

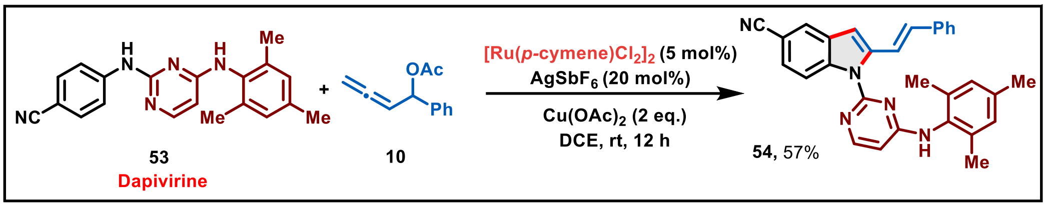

In an oven-dried reaction tube, charged with magnetic stir-bar, [*Ru(p-cymene)Cl*$_2$]$_2$ (5 mol%), and Cu(OAc)$_2$ (2 equiv.), $AgSbF_6$ (20 mol %)($AgSbF_6$ was taken inside the glove box), Dapivirine **53** (0.15 mmol,1.5 equiv) and allenyl carbinol acetates **10** (0.1 mmol, 1.equiv) were added followed by addition of 1 ml of 1,2-dichloroethane via syringe. The reaction mixture was allowed to stir at room temperature for 12 h. Then, the mixture was cooled and diluted with CH_2Cl_2 (10 mL). The mixture was filtered through a Celite pad and washed with CH_2Cl_2 (3 × 10 mL). The filtrate was concentrated under reduced pressure. The residue was purified by silica gel column chromatography using hexane/ethyl acetate as eluent to afford the desired pure product as white solid **54** (26 mg, 57% yield).

(c) General procedure for the synthesis of Bis-vinyl indole derivatives.

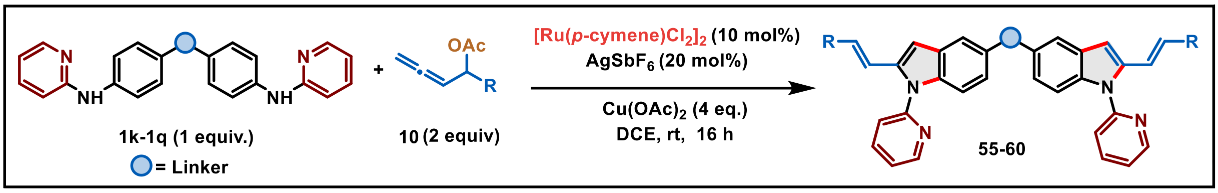

In an oven-dried reaction tube, charged with magnetic stir-bar, [*Ru(p-cymene)Cl*$_2$]$_2$ (10 mol%), and Cu(OAc)$_2$ (4 equiv.), AgSbF$_6$ (20 mol %)(AgSbF$_6$ was taken inside the glove box), Bis - N-aryl aminopyridines **1** (0.1 mmol,1 equiv.) and allenyl acetates **10** (0.2 mmol, 2 equiv) were added followed by addition of 1 ml of 1,2-dichloroethane via syringe. The reaction mixture was allowed to stir at room temperature for 16 h. Then, the mixture was cooled and diluted with CH_2Cl_2 (10 mL). The mixture was filtered through a Celite pad and washed with CH_2Cl_2 (3 × 10 mL). The filtrate was concentrated under reduced pressure. The residue was purified by silica gel column chromatography using hexane/ethyl acetate as eluent to afford the desired pure product.

5. Mechanistic studies

(a) Deuterium exchange experiment

In an oven-dried reaction tube, charged with magnetic stir-bar, [*Ru(p-cymene)Cl*$_2$]$_2$ (5 mol%), Cu(OAc)$_2$ (0.4 mmol), AgSbF$_6$ (20 mol %)(AgSbF$_6$ was taken inside the glove box), N-aryl aminopyridine **1** (0.2 mmol) were added followed by addition of DCE/D_2O (1.6/0.4 ml), via syringe. The reaction mixture was allowed to stir at room temperature for 12 h. Then, the mixture was cooled and diluted with CH_2Cl_2 (10 mL). The mixture was filtered through a Celite pad and washed with CH_2Cl_2 (3 × 10 mL). The filtrate was concentrated under reduced pressure. The residue was purified by silica gel column chromatography using hexane/ethyl acetate as eluent to afford recovered substrate **1** with incorporation of deuterium.

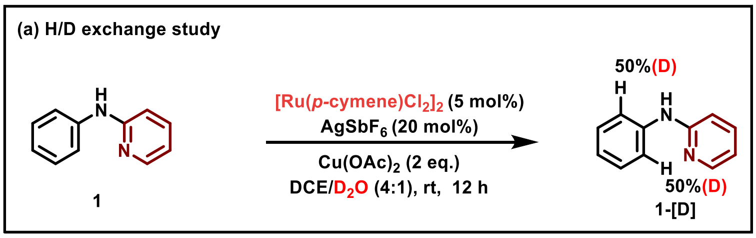

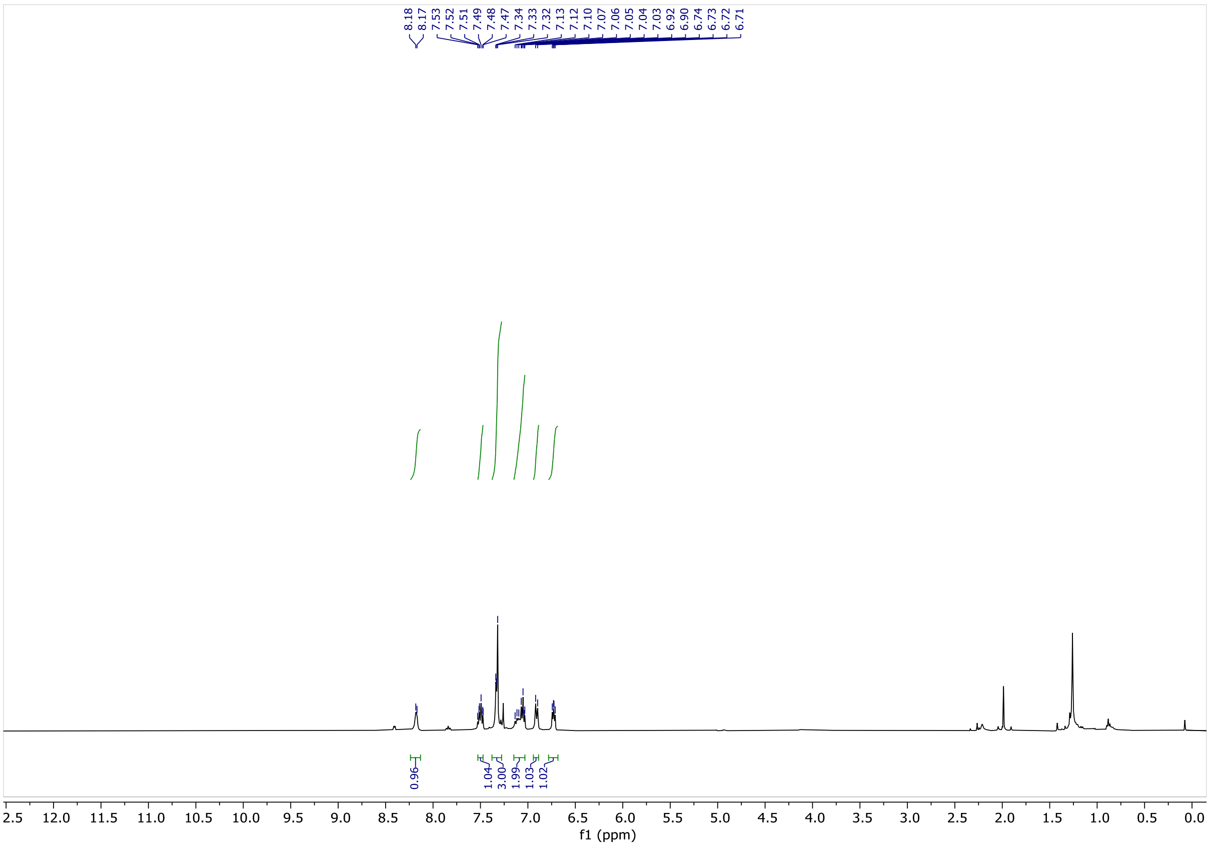

(b) Deuterium exchange experiment with coupling partner

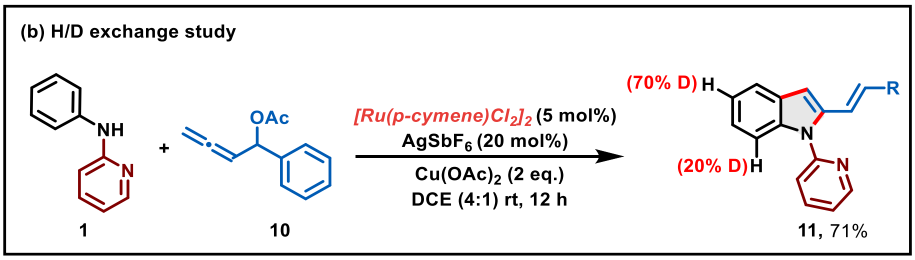

In an oven-dried reaction tube, charged with magnetic stir-bar, [*Ru(p-cymene)Cl*$_2$]$_2$ (5 mol%), and $Cu(OAc)_2$ (0.4 mmol, 2 equiv.), $AgSbF_6$ (20 mol %)($AgSbF_6$ was taken inside the glove box), N-aryl aminopyridine **1** (0.30 mmol,1.5 equiv) and allenyl acetate **10** (0.2 mmol, 1 equiv.) were added followed by addition of DCE/D_2O (1.6/0.4 ml) via syringe. The reaction mixture was allowed to stir at room temperature for 12 h. Then, the mixture was diluted with CH_2Cl_2 (10 mL). The mixture was filtered through a Celite pad and washed with CH_2Cl_2 (3 × 10 mL). The filtrate was concentrated under reduced pressure. The residue was purified by silica gel column chromatography using hexane/ethyl acetate as eluent to afford the desired pure product 3 in which 20% of deuterium incorporation occurred at ortho C-H proton and 70% deuterium incorporation at C-5 C-H proton.

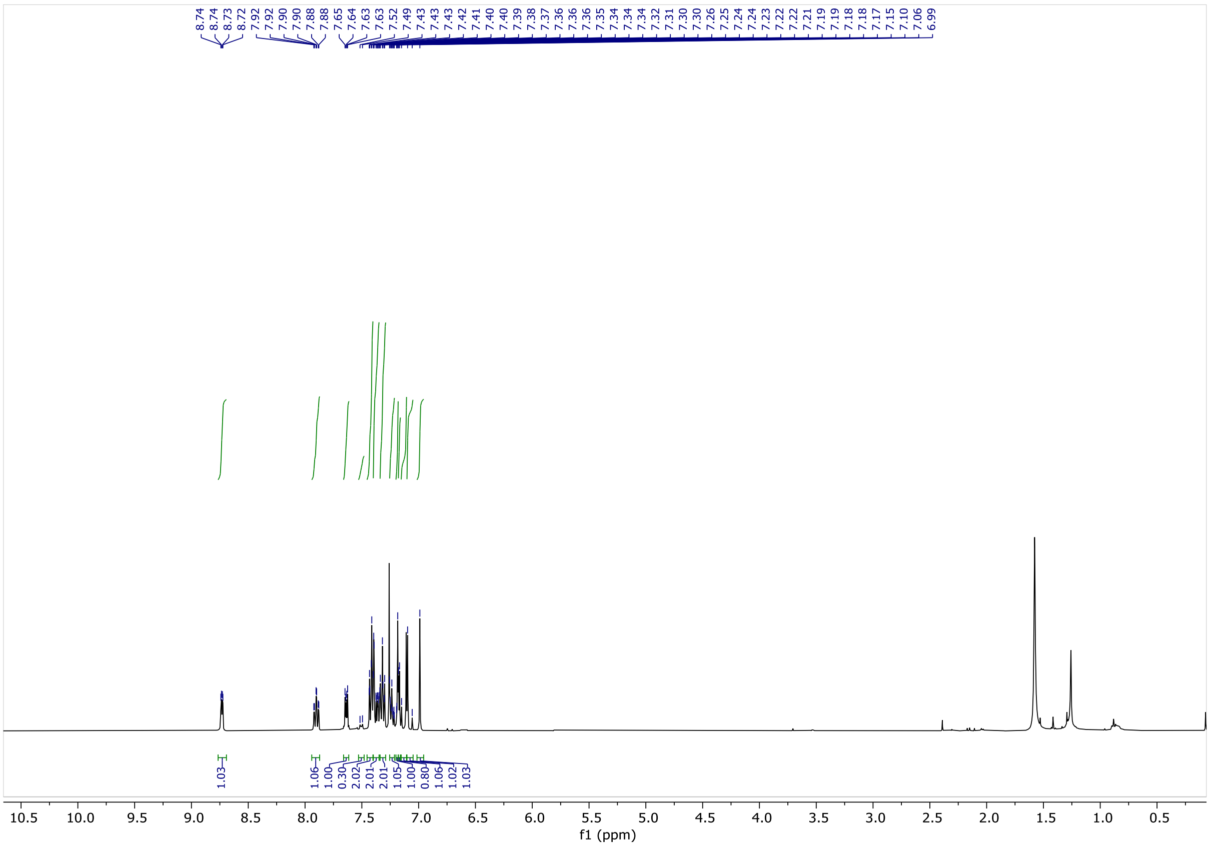

(c) Deuterium exchange experiment with product

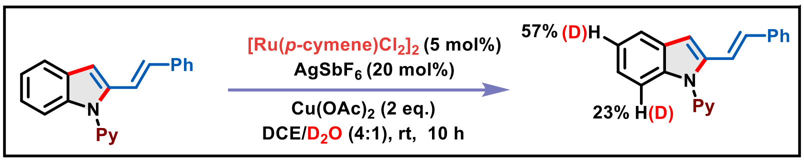

In an oven-dried reaction tube, charged with magnetic stir-bar, [*Ru(p-cymene)Cl*$_2$]$_2$ (5 mol%), and $Cu(OAc)_2$ (0.4 mmol, 2 equiv.), $AgSbF_6$ (20 mol %)($AgSbF_6$ was taken inside the glove box), 2-vinylindole **11** (0.2 mmol,1 equiv.) were added followed by addition of DCE/D_2O (1.6/0.4 ml) via syringe. The reaction mixture was allowed to stir at room temperature for 10 h. Then, the mixture was diluted with CH_2Cl_2 (10 mL). The mixture was filtered through a Celite pad and washed with CH_2Cl_2 (3 × 10 mL). The filtrate was concentrated under reduced pressure. The residue was purified by silica gel column chromatography using hexane/ethyl acetate as eluent to afford the desired pure product **11** in which 23% of deuterium incorporation occurred at ortho C-H proton and 57% deuterium incorporation at C-5 C-H proton.

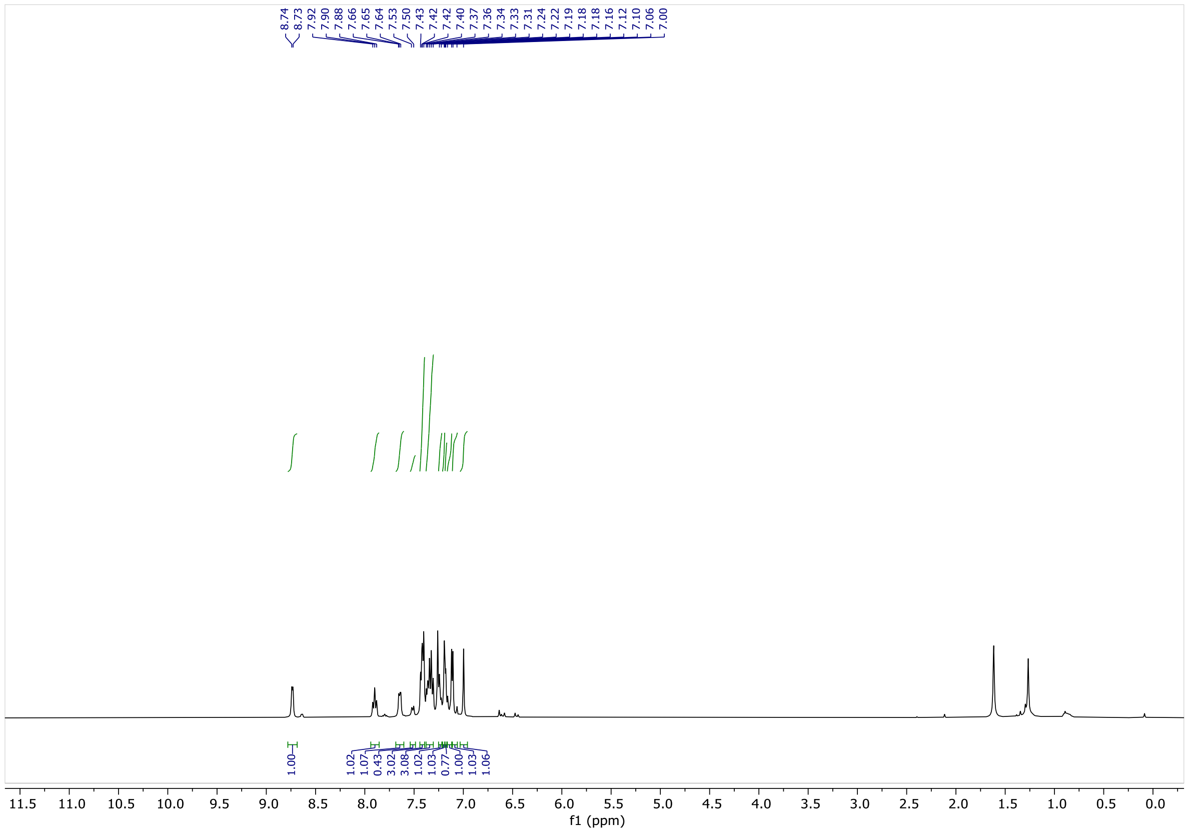

(d) Kinetic isotopic studies

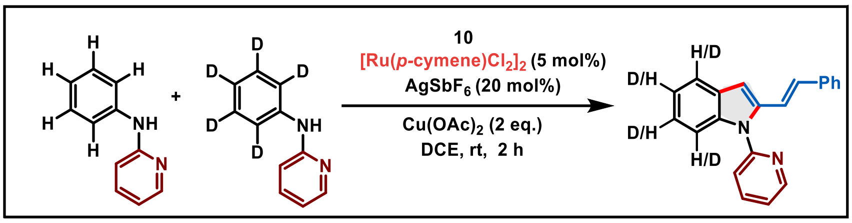

In an oven-dried reaction tube, charged with magnetic stir-bar, [*Ru(p-cymene)Cl$_2$*]$_2$ (5 mol%), and Cu(OAc)$_2$ (0.4 mmol), AgSbF$_6$ (20 mol %)(AgSbF$_6$ was taken inside the glove box). Then an equimolar ratio of H$_5$- N-aryl aminopyridine **1** (0.15 mmol,1.5 equiv) and D$_5$- N-aryl aminopyridine 1 (0.15 mmol,1.5 equiv) and allenyl acetate **10** (0.2 mmol, 1 equiv.), were added to an oven-dried screw cap reaction tube equipped with a stir bar. Then, DCE (2 mL) was added via syringe. The reaction mixture was allowed to stir at room temperature for 2 h. Then, the mixture was diluted with CH_2Cl_2 (10 mL). The mixture was filtered through a Celite pad and washed with CH_2Cl_2 (3 × 10 mL). The filtrate was concentrated under reduced pressure. The residue was purified by silica gel column chromatography using hexane/ethyl acetate as eluent to afford the desired pure product **11** in which k_h/k_d was found to be 1.63.

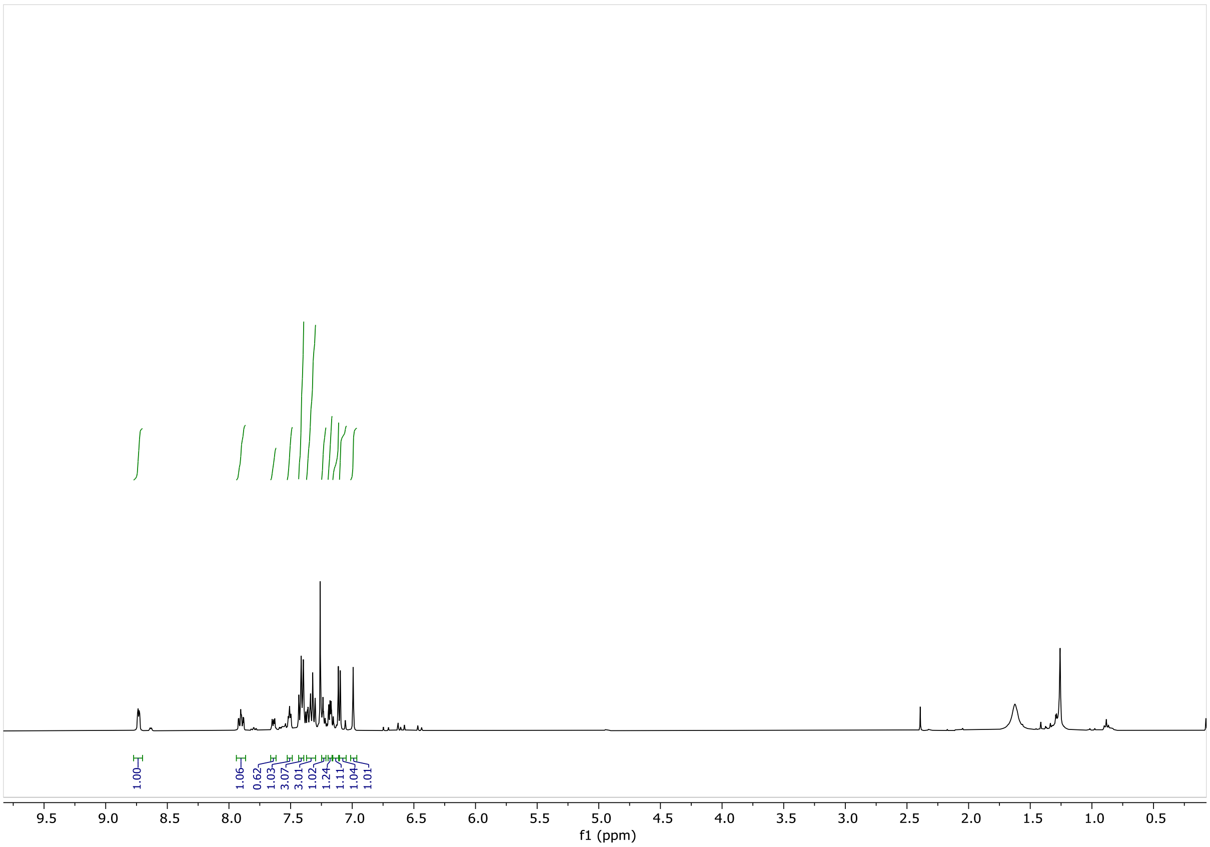

(e) Competitive Experiment:

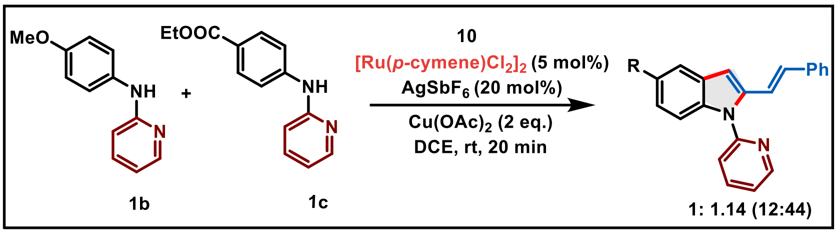

In an oven-dried reaction tube, charged with magnetic stir-bar, [*Ru(p-cymene)Cl*$_2$]$_2$ (5 mol%), and $Cu(OAc)_2$ (2 equiv.), $AgSbF_6$ (20 mol %) ($AgSbF_6$ was taken inside the glove box, An equimolar ratio of 4-methoxy N-phenyl aminopyridine **1b** and (0.15 mmol) and ethyl 4-(pyridin-2-ylamino)benzoate **1c** (0.15 mmol), **10** (0.2 mmol, 1 equiv.) followed by addition of DCE (2 mL) via syringe under nitrogen atmosphere The reaction mixture was allowed to stir at room temperature for 20 min. Then, the mixture was diluted with CH_2Cl_2 (10 mL). The mixture was filtered through a celite pad and the celite pad was washed with CH_2Cl_2 (3 × 5 mL).

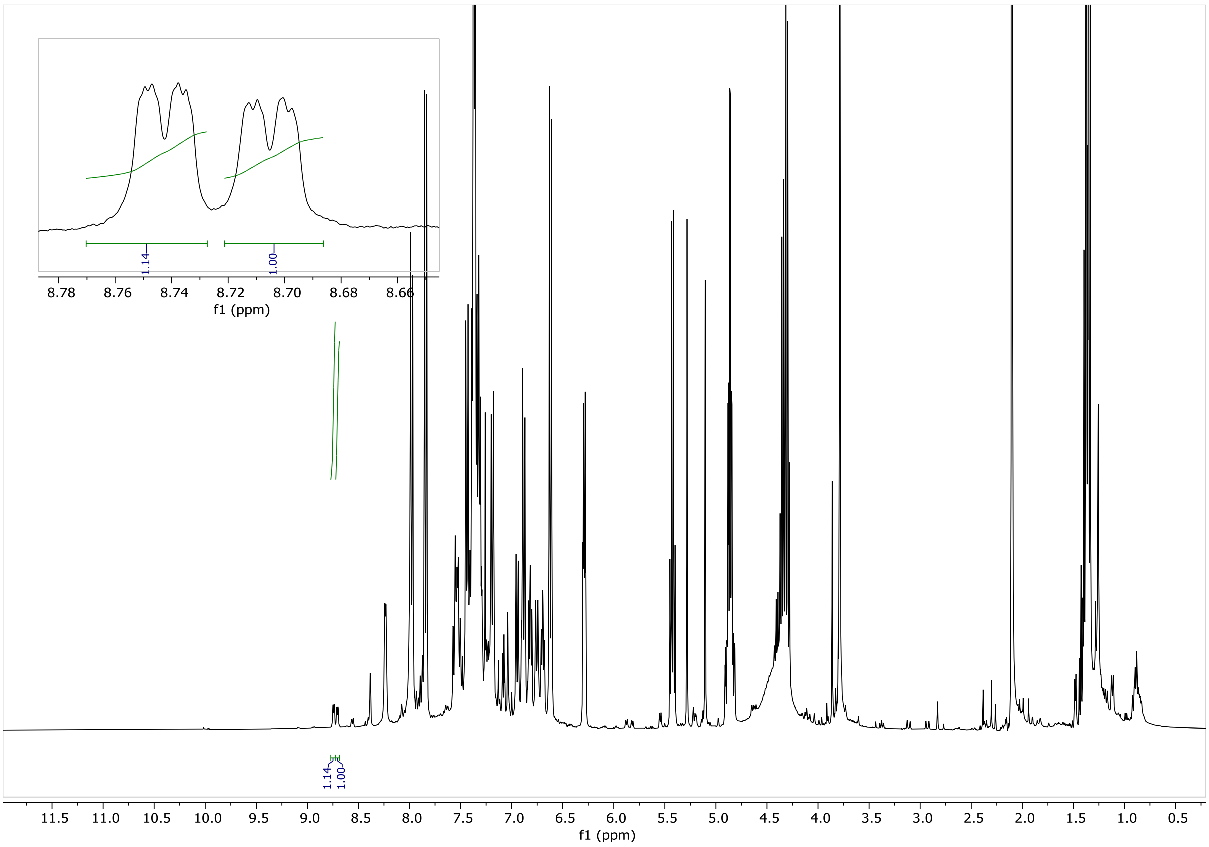

(f) Reaction with Co(III) catalyst

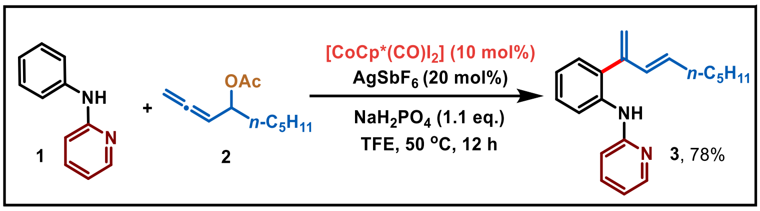

An oven dried Schlenk was charged with Teflon coated magnetic stir bar under argon atmosphere, Cp^*CoI_2 (10 mol%), and NaH_2PO_4 (1.1 equiv.), $AgSbF_6$ (20 mol %) ($AgSbF_6$ was taken inside the glove box), N-aryl aminopyridines **1** (0.2mmol, 1 equiv.) and allenyl acetates **2** (0.3 mmol, 1.5 equiv). were added followed by addition of 2 ml of TFE via syringe. The closed Schlenk tube containing reaction mixture were placed in preheated oil bath at 50 °C for 12 hours. The reaction mixture was allowed to cool to room temperature after above mentioned time. Removal of solvent followed by column chromatography on silica gel afforded dienylated product **3** in (46 mg, 78% yield).

(g) Reaction with 1,3-diene

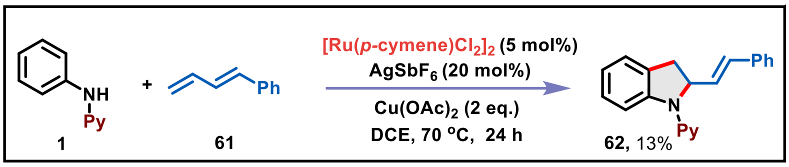

In an oven-dried reaction tube, charged with magnetic stir-bar, [*Ru(p-cymene)Cl*$_2$]$_2$ (5 mol%), and $Cu(OAc)_2$ (0.4 mmol), $AgSbF_6$ (20 mol %)($AgSbF_6$ was taken inside the glove box), *N*-aryl aminopyridines **1** (0.20 mmol, 1 equiv.) and 1,3-diene **61** (0.24 mmol, 1.2 equiv) were added followed by addition of 2 ml of 1,2-dichloroethane via syringe. The reaction mixture was allowed to stir at 70 °C for 24 h. Then, the mixture was cooled and diluted with CH_2Cl_2 (10 mL). The mixture was filtered through a Celite pad and washed with CH_2Cl_2 (3 × 10 mL). The filtrate was concentrated under reduced pressure. The residue was purified by silica gel column chromatography using hexane as eluent to afford the product **62** in 13% yield (8 mg). The yield was very low and rest of the starting material remains unreacted.

(f) Gram scale reaction.

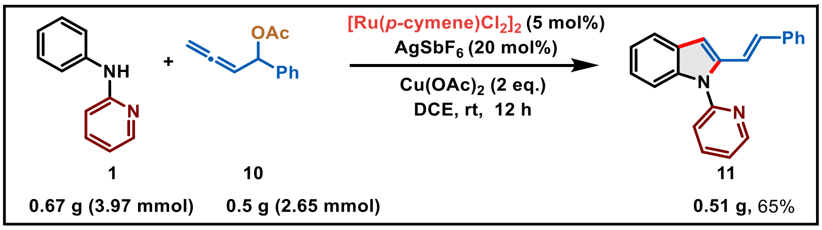

In an oven-dried reaction tube, charged with magnetic stir-bar, [*Ru(p-cymene)Cl*$_2$]$_2$ (83 mg, 5 mol%), and $Cu(OAc)_2$ (960 g mg, 5.3 mmol), $AgSbF_6$ (20 mol %) ($AgSbF_6$ was taken inside the glove box), N-aryl aminopyridines **1** (0.67 g, 3.97 mmol, 1.5 equiv) and allenyl acetates **10** (0.5 g, 2.65 mmol, 1 equiv) were added followed by addition of 30 ml of 1,2-dichloroethane via syringe. The reaction mixture was allowed to stir at room temperature for 12 h. Then, the mixture was cooled and diluted with CH_2Cl_2 (10 mL). The mixture was filtered through a celite pad and washed with CH_2Cl_2 (3 × 10 mL). The filtrate was concentrated under reduced pressure. The residue was purified by silica gel column chromatography using hexane/ethyl acetate as eluent to afford the desired pure product **11** in (0.51 g, 65% yield).

6. Mechanism

We attempted to isolate the intermediate **V**, which contains an exo-double bond and readily undergoes aromatization. As a result, we were unable to isolate it. However, this intermediate was detected by HRMS analysis.

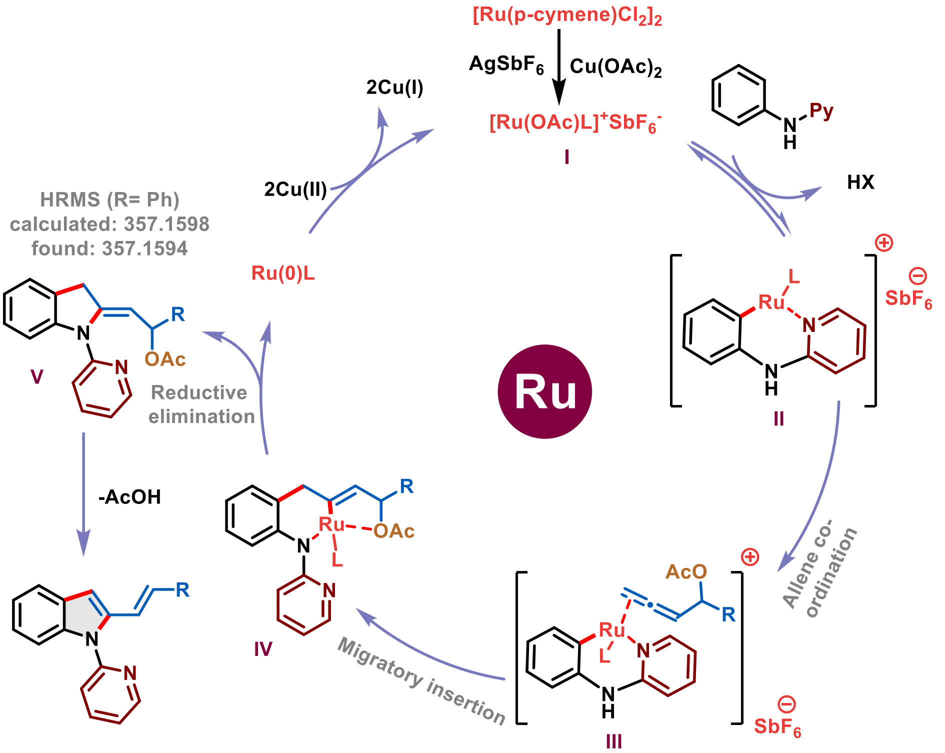

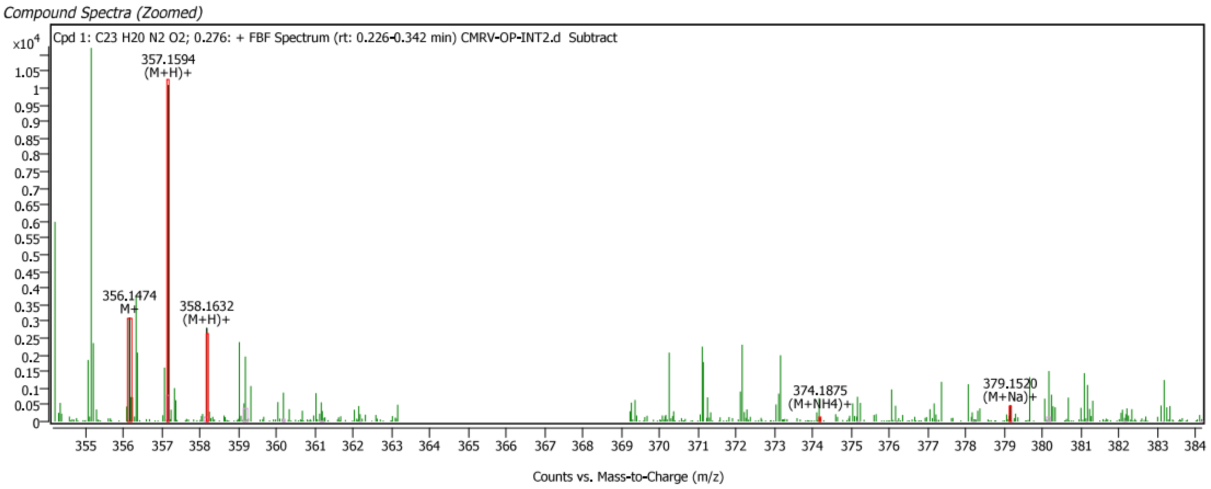

7. Control studies

(a) Role of pyridine as a directing group: -

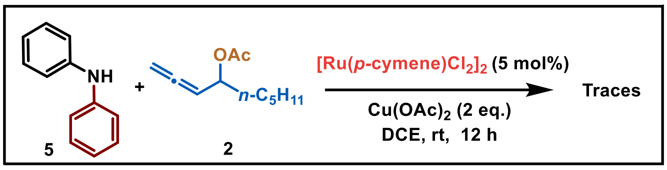

In an oven-dried reaction tube, charged with magnetic stir-bar, [*Ru(p-cymene)Cl*$_2$]$_2$ (5 mol%), and Cu(OAc)$_2$ (2 equiv.), diphenyl aniline **5** (0.3 mmol, 1.5 equiv.) and allenyl acetates **2** (0.2 mmol, 1equiv.) were added followed by addition of 2 ml of 1,2-dichloroethane via syringe. The reaction mixture was allowed to stir at room temperature for 12 h. No product formation was observed and starting materials remains unreactive.

(b) Role of N-H group: -

(i) Reaction with N-protected derivatives

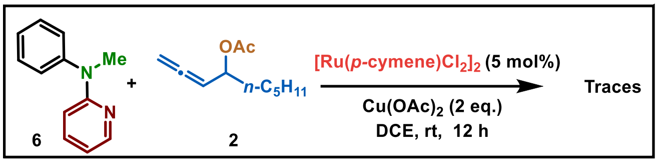

In an oven-dried reaction tube, charged with magnetic stir-bar, [*Ru(p-cymene)Cl*$_2$]$_2$ (5 mol%), and Cu(OAc)$_2$ (2 equiv.), *N*-methyl-*N*-phenylpyridin-2-amine **5** (0.3 mmol, 1.5 equiv) and allenyl acetates **2** (0.2 mmol, 1 equiv.) were added followed by addition of 2 ml of 1,2-dichloroethane via syringe. The reaction mixture was allowed to stir at room temperature for 12 h. No product formation was observed and starting materials remains unreactive.

(ii) Reaction with phenol derivatives

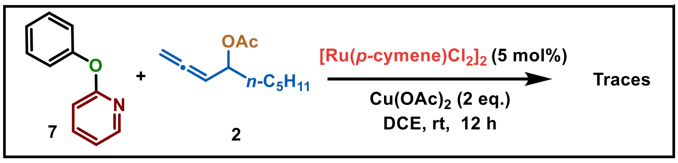

In an oven-dried reaction tube, charged with magnetic stir-bar, [*Ru(p-cymene)Cl*$_2$]$_2$ (5 mol%), and Cu(OAc)$_2$ (2 equiv.), AgSbF$_6$ (20 mol %) 2-phenoxy pyridine **7** (0.3 mmol,1.5 equiv) and allenyl carbinol acetates **2** (0.1 mmol, 1 equiv.) were added followed by addition of 2 ml of

1,2-dichloroethane via syringe. The reaction mixture was allowed to stir at room temperature for 12 h. No product formation was observed and starting materials remains unreactive.

(C) Reaction with other directing groups: -

In an oven-dried reaction tube, charged with magnetic stir-bar, [*Ru(p-cymene)Cl*$_2$]$_2$ (5 mol%), and Cu(OAc)$_2$ (2 equiv.), N-phenylacetamide **8** or methyl phenylcarbamate **9** (0.3 mmol,1.5 equiv) and allenyl acetates **2** (0.1 mmol, 1 equiv.) were added followed by addition of 2 ml of 1,2-dichloroethane via syringe. The reaction mixture was allowed to stir at room temperature for 12 h. No product formation was observed and starting materials remains unreactive.

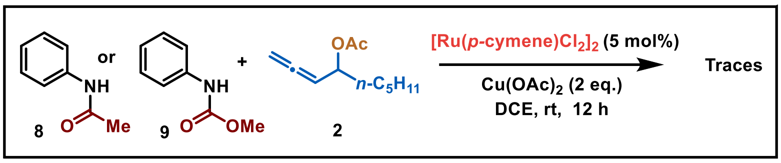

8. (a)X-ray Crystallography data for the compound of 34:

Crystal of the compound **34** was obtained after slow evaporation of chloroform solvent. Molecular structure of **34** with 50% ellipsoid probability.

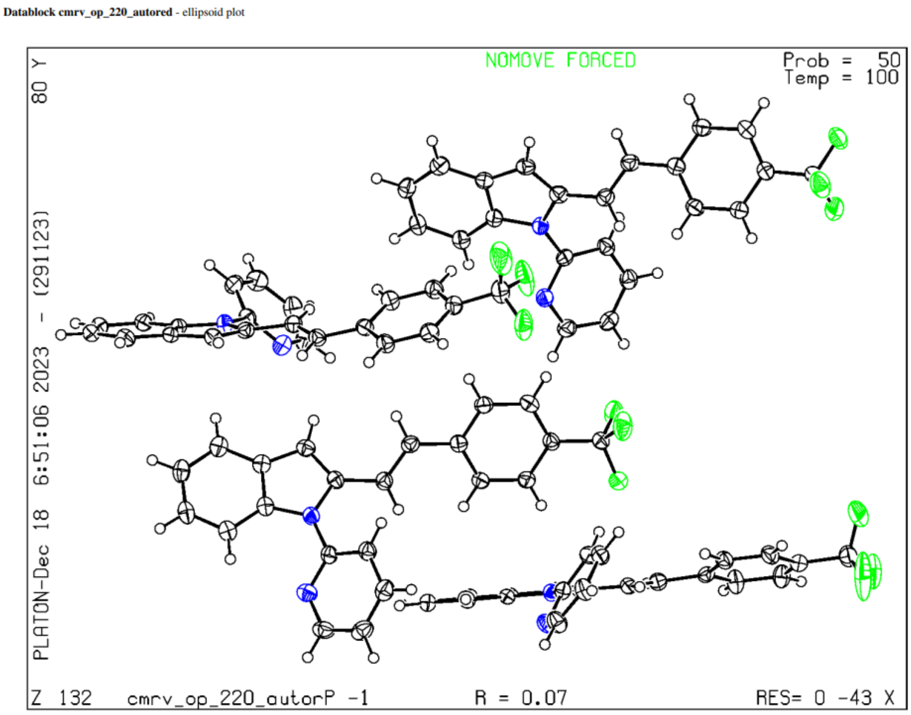

Figure S12. Molecular structure of **34** with 50% ellipsoid probability.

Table 1 Crystal data and structure refinement for CMRV_op_220_autored.	
Identification code	**CMRV_OP_220_autored**
Empirical formula	$C_{88}H_{60}F_{12}N_8$
Formula weight	1457.492
Temperature/K	100.00
Crystal system	triclinic
Space group	P-1
a/Å	13.1810(2)
b/Å	16.2119(4)
c/Å	16.3726(6)
α/°	94.325(2)
β/°	92.658(2)
γ/°	90.373(2)
Volume/Å^3	3484.75(16)
Z	2
ρ_{calc}g/cm^3	1.389
μ/mm^{-1}	0.105
F(000)	1505.1
Crystal size/mm^3	0.23 × 0.12 × 0.056
Radiation	Mo Kα (λ = 0.71073)
2Θ range for data collection/°	3.1 to 50
Index ranges	-20 ≤ h ≤ 20, -26 ≤ k ≤ 23, -24 ≤ l ≤ 25
Reflections collected	201080
Independent reflections	12287 [R_{int} = 0.1295, R_{sigma} = 0.1429]
Data/restraints/parameters	12287/0/973
Goodness-of-fit on F^2	1.070
Final R indexes [I>=2σ (I)]	R_1 = 0.0663, wR_2 = 0.1626
Final R indexes [all data]	R_1 = 0.0853, wR_2 = 0.1736
Largest diff. peak/hole / e Å^{-3}	0.65/-0.50

(b)X-ray Crystallography data for the compound of 54:

Crystal of the compound **54** was obtained after slow evaporation of chloroform solvent. Molecular structure of **54** with 50% ellipsoid probability.

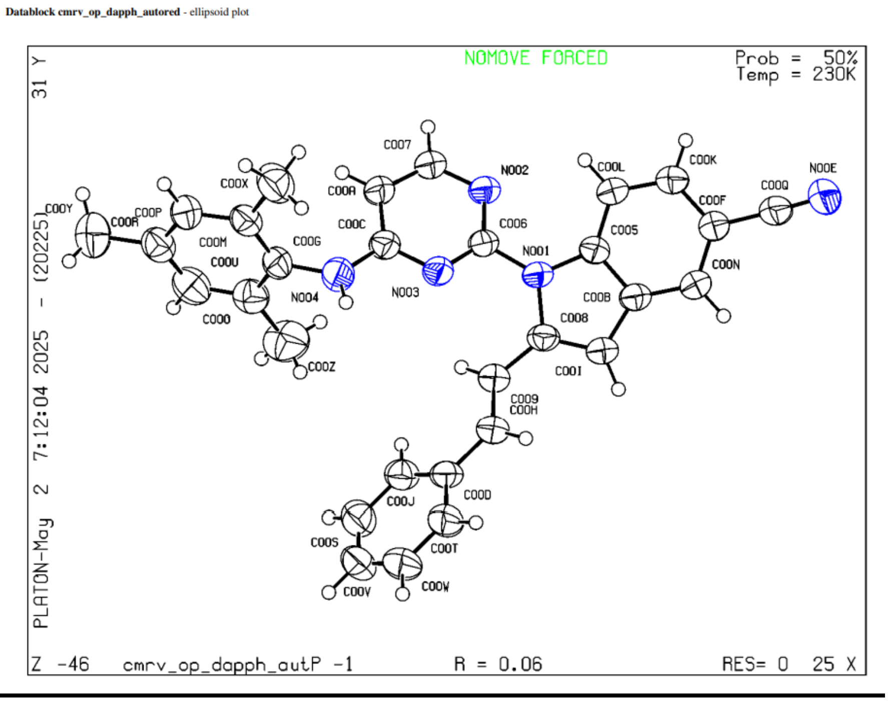

Table 2 Crystal data and structure refinement for CMRV_OP_DAPPH_autored.	
Identification code	CMRV_OP_DAPPH_autored
Empirical formula	$C_{30}H_{25}N_5$
Formula weight	455.55
Temperature/K	226(30)
Crystal system	triclinic
Space group	P-1
a/Å	7.9808(5)
b/Å	11.7665(9)
c/Å	13.3819(10)
α/°	89.340(6)
β/°	80.584(6)
γ/°	77.853(6)

Volume/Å^3	1211.61(15)
Z	2
ρ_{calc}g/cm^3	1.249
μ/mm^{-1}	0.076
F(000)	480.0
Crystal size/mm^3	0.0765 × 0.0657 × 0.0345
Radiation	Mo Kα (λ = 0.71073)
2Θ range for data collection/°	3.542 to 49.998
Index ranges	$-9 \le h \le 9$, $-13 \le k \le 13$, $-15 \le l \le 15$
Reflections collected	42095
Independent reflections	4261 [R_{int} = 0.1595, R_{sigma} = 0.0735]
Data/restraints/parameters	4261/0/319
Goodness-of-fit on F^2	0.989
Final R indexes [I>=2σ (I)]	R_1 = 0.0576, wR_2 = 0.1356
Final R indexes [all data]	R_1 = 0.1152, wR_2 = 0.1660
Largest diff. peak/hole / e Å^{-3}	0.17/-0.22

9. Functionalization

(a) Procedure for the hydrogenation reaction:

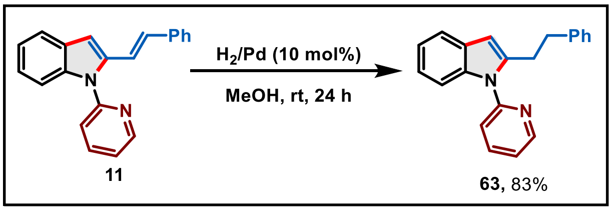

To a solution of 38 (E)-1-phenyl-2-styryl-1H-indole **11** (30 mg, 0.10 mmol) in MeOH (3 mL) was added Pd/C (10 wt.%, 0.0267mmol) and H_2 gas was bubbled from a balloon at room temperature. After 24 h, mixture was filtered through a pad of celite and washed with MeOH (10 mL) and diethyl ether (10 mL). The combined organic phase was dried with Na_2SO_4. After removal of the solvent under reduced pressure, the residue was purified by column chromatography (silica gel mesh100-200; (petroleum ether/EtOAc: 95/05) to give the desire product **63** as colourless liquid (24.7 mg, 83%yield).

(b) Procedure for C-7 functionalization of (E)-1-phenyl-2-styryl-1H-indole:

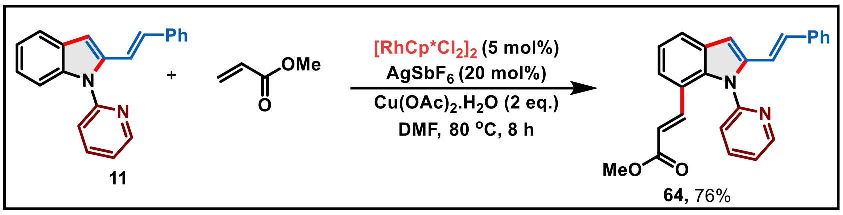

A mixture of **11** (30 mg, 0.1 mmol), [Cp*RhCl$_2$] (5 mol%), AgSbF$_6$ (20 mol%), Cu(OAc)$_2$ (0.2 mmol) and methyl acrylate (0.12 mmol,) was combined in a Schlenk tube followed by addition of DMF (1.0 mL) via syringe. Then the reaction mixture was heated to 80 ^{o}C with stirring for 8 h. It was allowed to cool to room temperature, diluted with EtOAc (10 mL) and washed brine. The combined organic phase was dried (Na_2SO_4). After evaporation of the solvents under reduced pressure, the crude product was purified on a silica gel column to give the desired product **64** (28.9 mg, 76%) as colourless liquid.

(c) Procedure for Removal of 2-pyridinyl group:

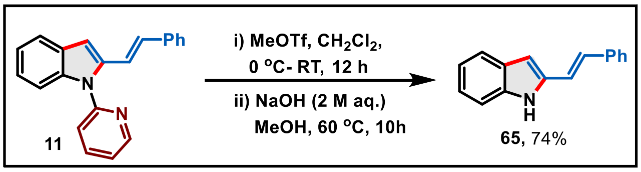

In an oven dried round bottom flask, (E)-1-phenyl-2-styryl-1H-indole **11** (60 mg, 0.20 mmol) was introduced and CH_2Cl_2 (5 mL) was added into it via syringe. Then Methyl trifluoromethanesulfonate (49 mg, 0.30 mmol) was added drop wise via a syringe to the reaction mixture at 0 ^{o}C and the resultant reaction mixture was stirred at room temperature for 10 h. Then the reaction mixture was removed under vacuum and the residue was further dissolved in MeOH (2 mL). To the resultant mixture, NaOH (2 mL, 2M aqueous) solution was added and the reaction mixture was stirred at 60 ^{o}C for 12h, then the reaction was allowed to cool to room temperature and the volatiles were evaporated under reduced pressure, and the resulting residue was extracted with EtOAc (15 mL x 3). The combined organic extract was washed with brine, dried over Na_2SO_4 and the volatiles were evaporated in vacuo. The remaining residue was purified by column chromatography on silica gel (silica gel mesh100-200; petroleum ether/EtOAc: 85/15) to yield **65** (32.4 mg, 74%) as white solid.

(d) Procedure for acid catalysed [4+2] Diels-alder reaction:

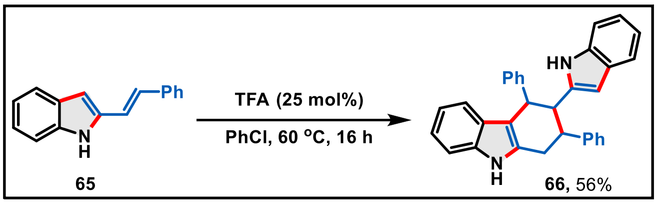

Trifluoroacetic acid (25 mol%) was added to the solution of (E)-1-phenyl-2-styryl-1H-indole **65** (0.10 mmol) in chlorobenzene (1mL), which was stirred at 60 ^{o}C for 16h. Then the reaction was allowed to cool to room temperature and the volatiles were evaporated under reduced pressure, and the resulting residue was extracted with EtOAc (15 mL x 3). The combined organic extract was washed with brine, dried over Na_2SO_4 and the volatiles were evaporated in vacuo, the remaining residue was purified by column chromatography on silica gel (silica gel mesh 100-200; petroleum ether/EtOAc: 80/20) to yield **66** (24.5 mg, 56%) as sticky solid.

(e) Procedure for Lewis acid catalysed annulation with propagyl alcohol:

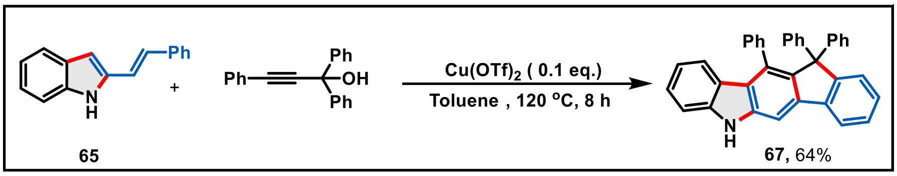

The reaction of propargylic alcohol (31 mg, 0.1 mmol), (E)-1-phenyl-2-styryl-1H-indole **65** (39 mg, 0.13 mmol), $Cu(OTf)_2$ (12 mol %) was combined in a reaction tube and toluene (2.0 mL) was added into it via syringe. Then the reaction tube was stirred at 120 °C under an air atmosphere for 8 h. The resulting mixture was cooled down to room temperature and evaporated under reduced pressure. The residue was further purified by chromatography on silica gel (petroleum ether/ethyl acetate, 10:1) (silica gel mesh 100-200; petroleum ether/EtOAc: 80/20) to yield **67** (31 mg, 64%) as yellow solid.

10. Photophysical Studies

(a) Absorption Spectrum of 3a, 3t and 3w: 5× 10^{-5} M solutions of **22**, **23**, **28**, **34** and **36** were prepared in CH_2Cl_2. And the UV-vis spectra were measured by using Perkin Elmer Lambda 950 spectrophotometer. The same concentration solutions were used for the measurement of Emission Spectrum of **22**, **23**, **28**, **34** and **36**.

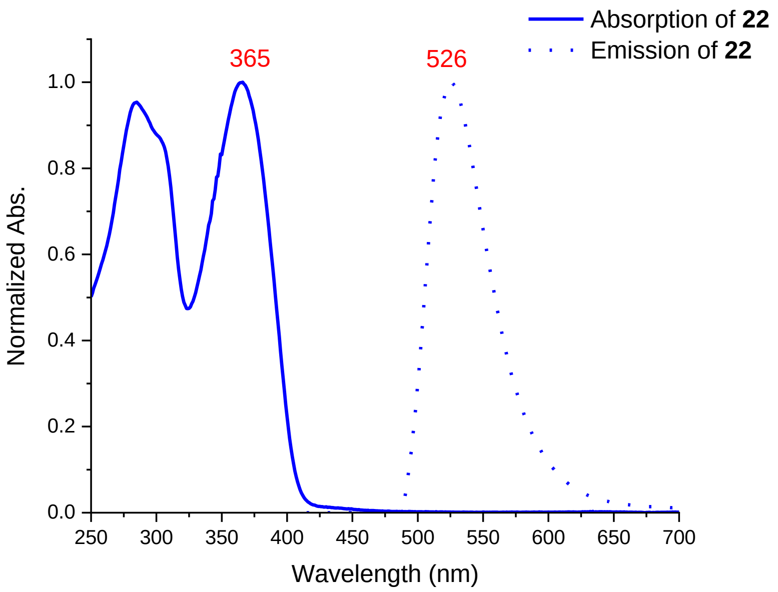
Absorption of 22
Emission of 22
365
526
Normalized Abs.
Wavelength (nm)

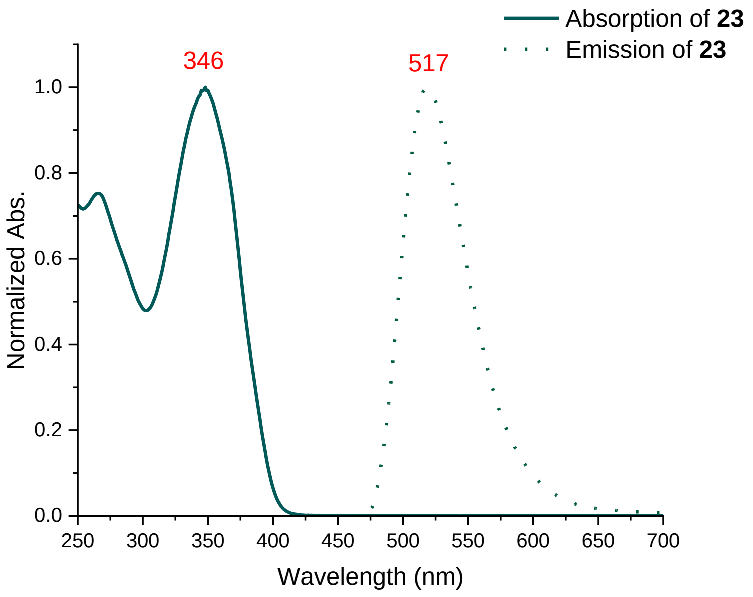
Absorption of 23
Emission of 23
346
517
Normalized Abs.
Wavelength (nm)

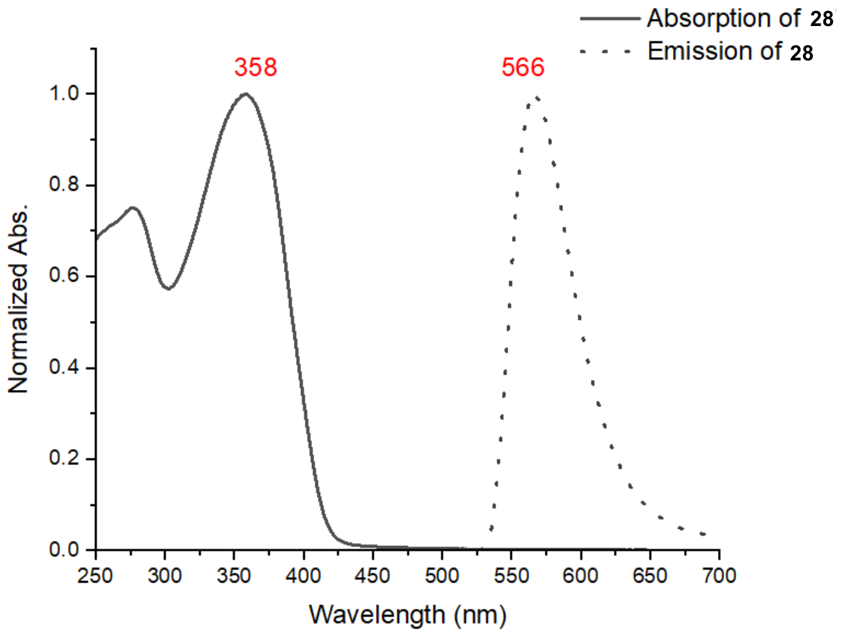
Absorption of 28
Emission of 28
358
566
Normalized Abs.
Wavelength (nm)

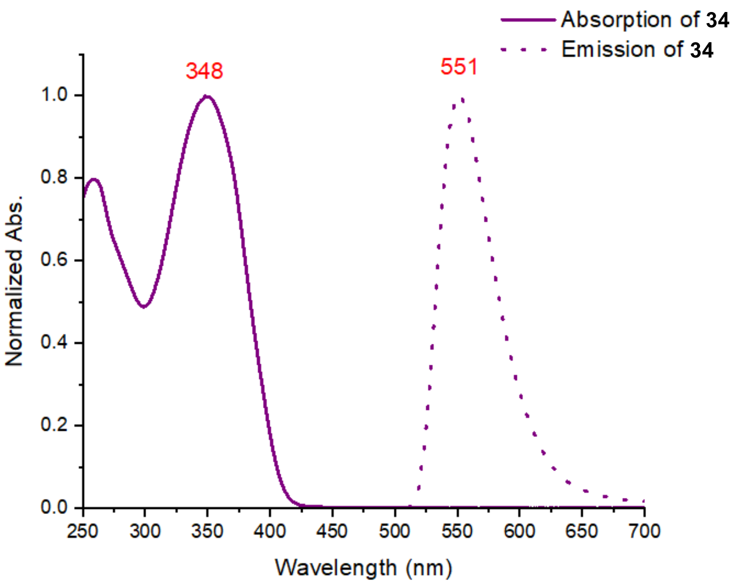
Absorption of 34
Emission of 34
348
551
Normalized Abs.
Wavelength (nm)

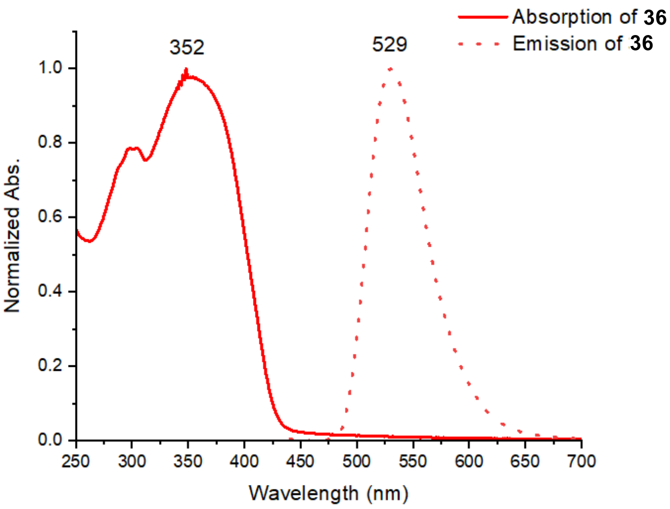
Absorption of 36
Emission of 36
352
529
Normalized Abs.
Wavelength (nm)

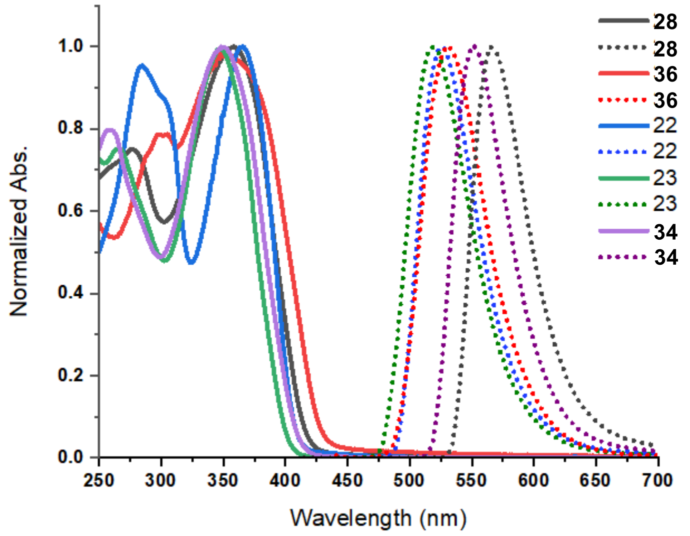

11. DFT studies:

The electronic properties of 28 are examined by using the Gaussian 09 program package

. The calculations are done in the ground state and gas phase.

Single point energy calculation:

E(B3LYP) = –1256.18687844 a.u.

Number of imaginary frequencies = 30

Optimization: B3LYP/6-31G*

E(B3LYP) = -1256.43309229 a.u.

Number of imaginary frequencies = 0

Computed Isodensity Surfaces of Molecular Orbitals of **34**:

**Molecular Orbital**	**Energy**	
	**Hartrees**	**eV**

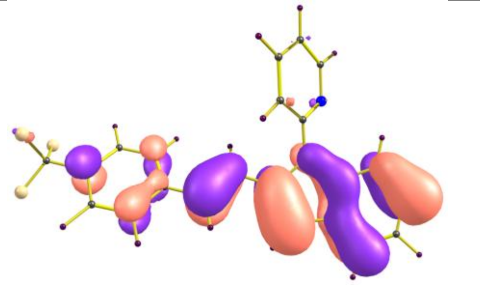	-0.196698449793	-5.35244
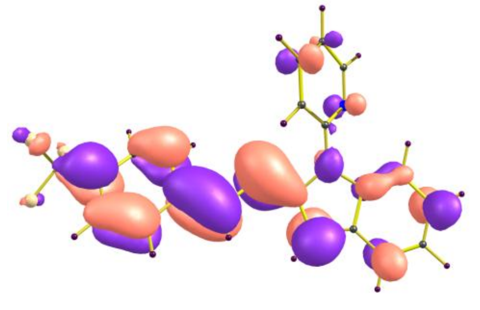	-0.06795460976	-1.84914

(a) Cartesian Coordinates for Optimized Structure of **34**:

Cartesian coordinates before optimization: - 34 crystal structure

	X	Y	Z
F	6.458000000	18.194000000	1.826000000
N	9.776000000	8.906000000	1.685000000
N	12.010000000	9.019000000	1.039000000
F	8.544000000	18.129000000	1.941000000
C	8.580000000	9.641000000	1.624000000
C	11.091000000	9.409000000	1.903000000
C	7.486000000	13.310000000	1.900000000
C	8.059000000	7.460000000	1.410000000
C	7.530000000	8.774000000	1.462000000
H	6.612000000	9.012000000	1.396000000

C	8.582000000	11.091000000	1.717000000
H	9.424000000	11.525000000	1.794000000
C	9.462000000	7.553000000	1.538000000
F	7.406000000	17.894000000	3.673000000
C	7.471000000	6.181000000	1.269000000
H	6.530000000	6.087000000	1.181000000
C	11.344000000	10.252000000	2.991000000
H	10.656000000	10.484000000	3.602000000
C	10.291000000	6.431000000	1.564000000
H	11.230000000	6.512000000	1.687000000
C	7.496000000	11.856000000	1.702000000
H	6.661000000	11.428000000	1.552000000
C	7.441000000	16.085000000	2.216000000
C	8.568000000	13.985000000	2.462000000
H	9.327000000	13.493000000	2.752000000
C	12.632000000	10.733000000	3.146000000
H	12.844000000	11.323000000	3.860000000
C	8.292000000	5.073000000	1.259000000
H	7.908000000	4.210000000	1.152000000
C	8.555000000	15.357000000	2.606000000

H	9.308000000	15.802000000	2.974000000
C	9.692000000	5.191000000	1.404000000
H	10.232000000	4.410000000	1.391000000
C	13.604000000	10.342000000	2.246000000
H	14.492000000	10.670000000	2.320000000
C	13.260000000	9.464000000	1.236000000
H	13.944000000	9.161000000	0.650000000
C	6.363000000	14.049000000	1.539000000
H	5.603000000	13.602000000	1.187000000
C	6.335000000	15.431000000	1.690000000
H	5.566000000	15.925000000	1.432000000
C	7.449000000	17.569000000	2.409000000

Cartesian coordinates after optimization: -

	X	Y	Z
F	-7.076122000	-1.061659000	0.060515000
N	2.845952000	0.055047000	-0.080075000
N	3.580157000	2.192720000	-0.680743000
F	-6.638893000	0.559557000	-1.331761000
C	1.776287000	-0.865381000	-0.043953000
C	2.744199000	1.459326000	0.061355000
C	-2.074917000	-0.873831000	0.118083000
C	3.719414000	-2.046225000	-0.063805000

C	2.295956000	-2.142029000	-0.036120000
H	1.709417000	-3.048562000	-0.082521000
C	0.390449000	-0.451163000	-0.139596000
H	0.208116000	0.524162000	-0.583207000
C	4.045787000	-0.663862000	-0.104246000
F	-6.640771000	0.931444000	0.807785000
C	4.751716000	-3.001817000	-0.063070000
H	4.515540000	-4.061712000	-0.038338000
C	1.826836000	2.024600000	0.962639000
H	1.204889000	1.392058000	1.583933000
C	5.373279000	-0.222284000	-0.123889000
H	5.608683000	0.832421000	-0.174204000
C	-0.657456000	-1.202066000	0.266435000
H	-0.449756000	-2.141455000	0.775407000
C	-4.833234000	-0.315657000	-0.077525000
C	-2.545963000	0.197430000	-0.668409000
H	-1.845402000	0.806976000	-1.229686000
C	1.758237000	3.410948000	1.049074000
H	1.056241000	3.877173000	1.733918000
C	6.067763000	-2.566238000	-0.086087000
H	6.876680000	-3.290713000	-0.085363000
C	-3.902813000	0.475936000	-0.760670000
H	-4.248189000	1.298144000	-1.378218000
C	6.371687000	-1.189892000	-0.113384000
H	7.410783000	-0.875111000	-0.136382000

C	2.616498000	4.186732000	0.268972000
H	2.599167000	5.270349000	0.311865000
C	3.516924000	3.523961000	-0.562413000
H	4.219783000	4.085668000	-1.174424000
C	-3.029519000	-1.663771000	0.787123000
H	-2.692732000	-2.503149000	1.389135000
C	-4.389839000	-1.390561000	0.695717000
H	-5.108744000	-2.013844000	1.216075000
C	-6.295484000	0.024032000	-0.138080000

12. Computational details

All the DFT calculations were performed in (CPCM:Dichloromethane)B3LYP-D3BJ/def2-SVP[17] level of theory using ORCA[18] 5.0.4 software. Fully optimized structures were characterized to be local minima as they feature zero imaginary frequencies. Energy values were derived from frequency analysis using the harmonic oscillator approach and include zero-point energy (ZPE) correction. Values of enthalpy (H) and Gibb's free energy (G) are unscaled and are reported for standard conditions if not stated otherwise.

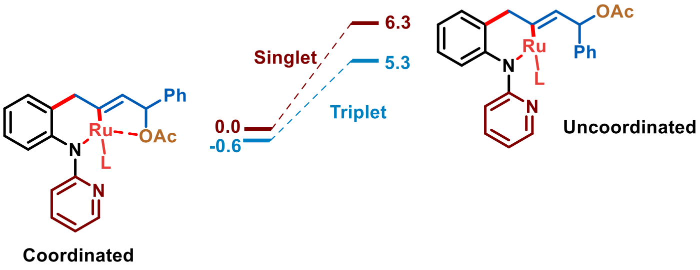

Optimized coordinates:

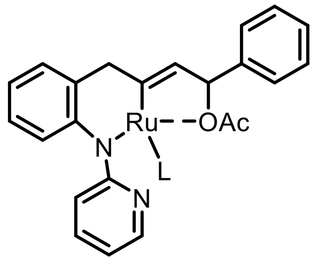	Singlet-Coordinated	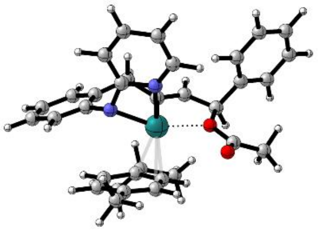

Charge:0

Multiplicity:1

G_{298K}: -1630.42506737

H_{298K}: -1630.32790294

XYZ

N	-1.23627156459247	1.69894135982155	-0.97241986793577
C	-0.19260182826690	0.76469586539594	-1.14309543994842
C	1.12955199799044	1.07852074279290	-0.80612552928783
C	-0.48819242158270	-0.47438095468768	-1.76133723291423
C	2.17274836818007	0.20051792634386	-1.11474939592761
H	1.32934789502770	2.03837783826828	-0.32595244007880
C	0.57044709478309	-1.33110936891678	-2.08359701270288
C	1.89549067662315	-1.00015686168774	-1.77374389942233
H	3.20193335302955	0.46316099002979	-0.85555698610067
H	0.35114165369730	-2.28114484110875	-2.58018342449745
H	2.70567799992783	-1.68403185336429	-2.03981355007247
C	-2.16924191450116	1.54282080475108	-0.00071503236632
C	-2.06006418762397	1.10571205060262	1.33431342983380
C	-4.48252467955619	1.92508309151915	0.24355630632738
C	-3.20615937594223	1.13427874055195	2.12389453777679
H	-1.09419192518649	0.77964627489147	1.72303779627756
C	-4.43283590517670	1.57114349798137	1.59070682121524
H	-5.41239106398862	2.24019410883221	-0.23632963332274
H	-3.14943244640989	0.82176817595992	3.17022078629166

H	-5.33448111384523	1.62019326553437	2.20271087746195
C	-1.92646318106895	-0.88175666281920	-2.00038357190181
H	-2.37990465109503	-1.05571829831912	-1.00480858741494
H	-1.94582435561283	-1.85933775559662	-2.51120383515667
C	-2.84740469143914	0.09934782522029	-2.71091196360510
Ru	-2.64919830608956	2.03505210938265	-2.45084164556984
C	-3.84171209171588	-0.36441355534997	-3.50099100081063
H	-3.96533899395459	-1.42972130351381	-3.73234973382798
C	-4.91857342599401	0.52222313294070	-4.05746174628415
H	-4.91525433982498	0.53045625123406	-5.15937106371695
C	-6.29342682695672	0.10215545211044	-3.57709126206845
C	-6.61926566596851	0.17415969661649	-2.21588458362636
C	-7.23084187005051	-0.41532481599213	-4.47877092340825
C	-7.87313285176283	-0.24132165344869	-1.76649561560607
H	-5.87930320626513	0.55220478222288	-1.51081076766112
C	-8.48284760846762	-0.84937482975276	-4.02866268204512
H	-6.98081696605089	-0.47362156037052	-5.54187598065894
C	-8.80870767213108	-0.75697861856200	-2.67279079058361
H	-8.11923768023085	-0.17216813014441	-0.70376686756947
H	-9.20634790474512	-1.25372257131524	-4.74112597092656
H	-9.78865670809149	-1.08886740909754	-2.32028471119234
O	-4.59319350211177	1.89412959303890	-3.60886596648480
C	-5.30195524158460	2.99785780341570	-3.99371304803069
O	-5.10235789779895	4.02635991605141	-3.39581926159326

C	-6.23774111137493	2.86117010884823	-5.15984839622263
H	-7.10064634946135	2.23537978656575	-4.89142597981835
H	-6.58492692833058	3.86223859277771	-5.44117880074245
H	-5.73245036740304	2.38215430234735	-6.01169169025573
N	-3.38948740242397	1.88132954164689	-0.52546947904231
C	-1.97056556263607	3.20834003563090	-4.24081856376026
C	-0.92782169078040	2.24095389017369	-4.25217102470477
C	0.26941213009460	2.58121773555271	-3.54698677322124
C	0.39143454758532	3.74087609420991	-2.81924010433769
C	-0.68683837111032	4.66709387422337	-2.73605467803146
C	-1.83034561063033	4.40913369471957	-3.47658871145870
H	-2.77522030503245	3.15708593722938	-4.97278627429554
H	1.11169978537789	1.89134144296200	-3.57846854501078
H	1.31767223766727	3.94809832639824	-2.27794259910143
H	-2.64628324334312	5.13445544125304	-3.49230260412363
C	-0.55743310014413	5.89542955753001	-1.87797076532942
H	-1.42652902037841	6.56092739945491	-1.98495328531101
H	-0.46924697077363	5.61441202349973	-0.81431035570278
H	0.35317819651415	6.46307532721460	-2.13260748042308
C	-0.91123671937307	1.04953774557801	-5.21686552874650
H	-1.03661584492488	0.13631030914556	-4.61192732723473
C	0.42990415307604	0.92109813604903	-5.95902903895910
H	1.26939722444080	0.72085940321165	-5.27795992019777
H	0.38364764121012	0.08543207947077	-6.67571850948236

H 0.66013987731230 1.84060123190428 -6.52352201094796

C -2.05584076082551 1.08173933112543 -6.23139752517686

H -2.04682161086975 0.16488220432351 -6.84110834592904

H -3.03483245008631 1.14508440965444 -5.74435247688082

H -1.95933734695119 1.94277678583636 -6.91403573641481

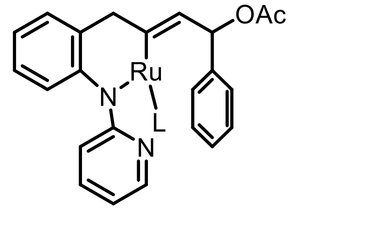	Singlet-uncoordinated	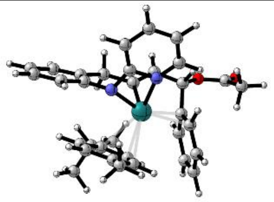

Charge:

Multiplicity:

G_{298K}: -1630.41498700

H_{298K}: -1630.31868067

XYZ

N -1.77990013680791 1.26723737543735 -1.53741936814976

C -0.65356256768889 0.44259007528964 -1.37666421908518

C 0.35965928111709 0.78157747081351 -0.46410670255529

C -0.52475177044669 -0.70969677674469 -2.17852856482906

C 1.51146084633886 0.00456943518096 -0.35160420818685

H 0.23315290988841 1.68402120373911 0.13745678883103

C 0.65443585729820 -1.46465426489124 -2.06753672918886

C 1.66644490312358 -1.12061947522100 -1.16998693138654

H 2.29360072341199 0.28460604622074 0.35890246193299

H 0.77159067589862 -2.35040753359532 -2.69964699992420

H 2.57198224861890 -1.72947628344423 -1.10674310852284

C	-2.87224706984030	1.09908277593248	-0.75699763561474
C	-2.99827289525270	0.67233155106596	0.58164306721793
C	-5.20027484658747	1.32934536974599	-0.97268543338085
C	-4.27349404664693	0.62280230713421	1.13067143601336
H	-2.10947456765394	0.40479765047108	1.15368213803879
C	-5.39685807424617	0.96922316465330	0.35701600585270
H	-6.04690876110696	1.57126016193357	-1.61935843029441
H	-4.40375327828542	0.31125327626474	2.17057810107735
H	-6.40510142668795	0.94591509246768	0.77225604075795
C	-1.59382696718433	-1.18700084045884	-3.14364481630698
H	-1.90534385900292	-2.19757410216367	-2.81999706556796
H	-1.09561739507471	-1.35246556837709	-4.11518798354834
C	-2.84512991484702	-0.37405097827080	-3.41705364765629
Ru	-2.85528679559896	1.62333983430933	-3.26292958479532
C	-3.97837424089343	-0.98711766119336	-3.80561592474454
H	-4.10243216989282	-2.07563458343439	-3.87177935810550
C	-5.14648376199369	-0.12227268695787	-4.18767174621865
H	-5.77177210234561	0.10759237290119	-3.31219159181269
C	-4.66341683853736	1.17997085806778	-4.82203704500241
C	-4.30563972812480	1.13812598535759	-6.20145105148364
C	-4.89464544533252	2.45484117446959	-4.23609494993910
C	-4.20025767807462	2.29035293218033	-6.95414430468432
H	-4.16241910107599	0.16432544173122	-6.67123358929981
C	-4.81081096915772	3.62921534072658	-5.03454713969407

H	-5.42106532632216	2.53279953183147	-3.28699046614590
C	-4.45946893256880	3.55375109884949	-6.36787632897101
H	-3.93347675284231	2.22688521676815	-8.01194329394800
H	-5.05872082829572	4.59249508915006	-4.58225271436995
H	-4.40075135775702	4.45995727993189	-6.97568303719418
O	-5.95518005839635	-0.88940076990759	-5.12081609082282
C	-7.22059518523734	-0.56249925732241	-5.44811527328451
O	-7.79050951270256	-1.24565754549461	-6.26941154272427
C	-7.85685235805879	0.62991399265716	-4.77662710589102
H	-7.33321270037889	1.55359193162713	-5.06458587961150
H	-8.90469661903344	0.68863457903155	-5.09373431875735
H	-7.80360854358191	0.55093992699018	-3.68039815023769
N	-3.97588734741718	1.38238121658534	-1.51153608108617
C	-1.58071060667762	2.89448518650507	-4.77040440755174
C	-0.50798396816215	2.01533186702136	-4.50873251611129
C	0.37245250571475	2.34912016564625	-3.43800589634212
C	0.15815642787053	3.44310321795020	-2.63329116787240
C	-0.95797963933039	4.29529562169807	-2.84802422157007
C	-1.78354764729160	4.03009720382102	-3.93299734577131
H	-2.13895598628701	2.81763487733522	-5.70160913479904
H	1.23423538222447	1.70403020570430	-3.25552670811896
H	0.83979642744054	3.65237663840851	-1.80524197719304
H	-2.60471341643354	4.71080898387027	-4.16063472390336
C	-1.21341437267572	5.45014226514211	-1.91945716313501

H	-2.04181254601667	6.08120808195872	-2.27291075526833
H	-1.46888897700301	5.08267203351826	-0.91058829962166
H	-0.31413600603595	6.07862080622366	-1.81098025335817
C	-0.02503147218610	0.94772269575499	-5.48067923211407
H	0.38417419812168	0.12931696505505	-4.86458458091648
C	1.14079840487845	1.51591093612689	-6.31383415394050
H	1.95405498859220	1.89246615663339	-5.67479984648658
H	1.55649424683575	0.73862250525835	-6.97541011707658
H	0.79172834924914	2.35047147688317	-6.94475966808456
C	-1.09622718482229	0.36707634617476	-6.39857745874059
H	-0.66881694658959	-0.44286209523484	-7.01027517044293
H	-1.94042033913243	-0.03457602211595	-5.82551842188914
H	-1.49310397719221	1.12931933864202	-7.08631396628068

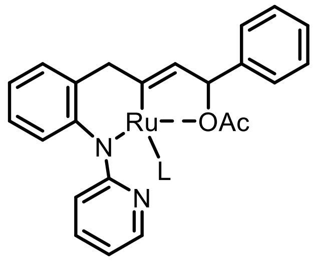	Triplet-coordinated	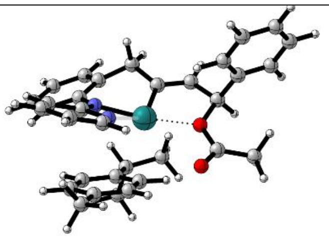

Charge:0

Multiplicity:3

G_{298K}: -1630.42602407

H_{298K}: -1630.32713400

XYZ

N	-1.67624183538689	1.02542317592536	-1.11172913606556
C	-0.61103763758850	0.18168685506634	-1.36816297817739

C	0.70709951827516	0.46098512447858	-0.95247120277806
C	-0.85130563060478	-0.97994432593398	-2.15624779420917
C	1.77137239027843	-0.36053529903877	-1.32552854146207
H	0.90228085427538	1.36429145530889	-0.37558994327854
C	0.23757060983732	-1.77120083064979	-2.53691675547906
C	1.54620794335879	-1.47602124642312	-2.13677002585368
H	2.78468317856431	-0.10979851781253	-0.99921820692519
H	0.04926605180631	-2.65680231056142	-3.15164389441554
H	2.37576026670939	-2.11430036516477	-2.45098743189035
C	-1.93911269295907	1.82645826786841	-0.06305669035497
C	-1.26420156494992	2.02359861679995	1.16869164876208
C	-3.64418845681431	3.31438031414774	0.58637925111377
C	-1.82987481896772	2.88801318956016	2.09557060104346
H	-0.33840665552769	1.49381426912025	1.38715619110202
C	-3.03631533838500	3.55892793264252	1.81488405689448
H	-4.58424261173968	3.80288187352185	0.30979015143278
H	-1.32929076300504	3.04677037329676	3.05512676447241
H	-3.48809184062670	4.24636821457363	2.53104144222306
C	-2.25969703850523	-1.43930546892572	-2.48661945099819
H	-2.69316695373828	-1.84153238065664	-1.54887753322887
H	-2.20069916120809	-2.29681172100743	-3.17945322999061
C	-3.24378392421871	-0.40727399473218	-2.98859589875110
Ru	-3.24063737133016	1.45848026881135	-2.34169206976775
C	-4.21130452106314	-0.72107890735073	-3.88449856751415

H	-4.31196354436839	-1.71878444630215	-4.33451508716508
C	-5.28795782443325	0.24239948495317	-4.30373249119904
H	-5.32927186300983	0.32431463426459	-5.40007709224270
C	-6.64551747031807	-0.16488602852575	-3.76411136871900
C	-6.94499651053212	0.00709167971338	-2.40455062973644
C	-7.59045380530271	-0.75913891131929	-4.60820670715606
C	-8.18120594108912	-0.40046234918891	-1.90127320172831
H	-6.20659723877441	0.46606516781180	-1.74160272256251
C	-8.82449736938044	-1.18081053822691	-4.10057508422739
H	-7.36115791196585	-0.88720985682514	-5.66966363810373
C	-9.12303426957971	-0.99940138687357	-2.74761149094055
H	-8.41026901119219	-0.25534003019546	-0.84264256867748
H	-9.55631016682540	-1.64397855377338	-4.76716527808226
H	-10.08911192190959	-1.32208114385575	-2.35097321788343
O	-4.92060489692178	1.56649888475076	-3.76719542117303
C	-5.56633575595338	2.72552956396406	-4.12306239792083
O	-5.33562685197009	3.70710569911451	-3.46450006361855
C	-6.46721079705210	2.67765330304574	-5.32090309192538
H	-7.30634576619558	1.98768517356575	-5.14987408115746
H	-6.85068929491579	3.68788998441497	-5.50432387011231
H	-5.91432008951927	2.32126373646709	-6.20337292699160
N	-3.12176241688593	2.48029719811647	-0.31605391571257
C	-1.76554233742133	3.01916630551426	-4.09458087185145
C	-0.50940406578351	2.39191074298671	-4.12377912749812

C	0.42405753670024	2.77543777865427	-3.14838855812812
C	0.12427090065663	3.73613312795286	-2.18579985518896
C	-1.12557731699668	4.37492080940838	-2.16083169023636
C	-2.06135399293664	4.00408209113986	-3.13224971293372
H	-2.50529106919946	2.79825005827023	-4.86438777069499
H	1.40151845232801	2.28887919890168	-3.12956855987197
H	0.87206798505504	3.99113399846004	-1.42895888147158
H	-3.04243067003953	4.48202202920539	-3.14984357393043
C	-1.44654805904937	5.40169028884586	-1.10804220560872
H	-2.48366721666851	5.75513273907163	-1.19635682737627
H	-1.31965323208524	4.98070593143332	-0.09804048421575
H	-0.77770676705475	6.27575402883318	-1.18278435274084
C	-0.12314493932571	1.33807167626614	-5.15092428062996
H	0.29705634615858	0.49246103649932	-4.57738159677562
C	0.98467521806261	1.86377681774881	-6.08130407489115
H	1.86261524845034	2.21057497889757	-5.51507484690525
H	1.31629484205677	1.07451481450806	-6.77564653423884
H	0.61330659181294	2.71180699854972	-6.68128005839253
C	-1.29894864937452	0.80183213581763	-5.96987067700601
H	-0.95822091455643	-0.01002063857138	-6.63177070333813
H	-2.09653425526982	0.40469526853109	-5.32519464248987
H	-1.73479091390952	1.58677695511414	-6.61072752245222

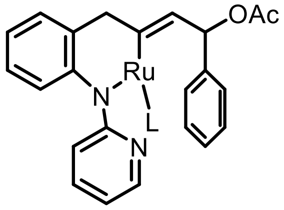	Triplet-uncoordinated	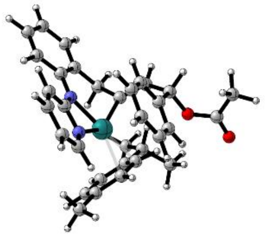

Charge:0

Multiplicity:3

G_{298K}: -1630.41850703

H_{298K}: -1630.31926134

XYZ

N	-1.87670227819562	1.01385899772817	-1.14954041690436
C	-1.30905825178296	-0.16018867995435	-0.69030629125936
C	-1.16850189654360	-0.47565227383420	0.68293004767711
C	-0.78072263956539	-1.08597545133799	-1.64198870347761
C	-0.59617130034584	-1.67580056681027	1.10483628079637
H	-1.47754390958539	0.24106323247523	1.43867609534935
C	-0.22509706952934	-2.28690999546974	-1.18787054983321
C	-0.13335513121188	-2.60416986465539	0.17099934536754
H	-0.51105566483055	-1.87727767389890	2.17637291317299
H	0.15637682400711	-2.99265641919818	-1.93161197078343
H	0.30481525598279	-3.55278527772941	0.49031857115808
C	-2.61835628717114	1.94926215820136	-0.50161121158542
C	-3.18667938287214	2.00589872433528	0.79431367556014
C	-3.64155043944801	4.00536693646358	-1.06251765171340
C	-3.95598566317679	3.11410777100371	1.13335758960361
H	-3.06629509640234	1.19399449821956	1.50521678100977

C	-4.18423457021667	4.14677623518593	0.21093327909792
H	-3.82257239307797	4.74485552110542	-1.84595361965700
H	-4.40016960445551	3.16647132365450	2.13118284858775
H	-4.78647851401567	5.02028392312066	0.46342624017253
C	-0.74836242521062	-0.79518525847293	-3.13364997065594
H	-0.56216308907145	-1.74456194196504	-3.66496451710532
H	0.12584862139750	-0.14817845846856	-3.34568593724083
C	-1.99721210245818	-0.12425226223653	-3.65398547922111
Ru	-2.06653778538296	1.81623857088467	-3.05850400689702
C	-2.94323284055886	-0.85402332655222	-4.27516287610613
H	-2.76778829154651	-1.92079993762957	-4.47412464530993
C	-4.26269545424276	-0.32659244300461	-4.76827017015458
H	-4.97210100425565	-1.16690971007011	-4.79312928798717
C	-4.87472696474198	0.76852833146960	-3.90892415824474
C	-5.29203737055957	2.00025167732821	-4.43003914322230
C	-5.12507862363885	0.49054214567254	-2.55296621530312
C	-5.95505160860026	2.92817566636215	-3.61875886634638
H	-5.10148746670169	2.24646562955779	-5.47522225739508
C	-5.79397469832365	1.41007002676732	-1.74811778222767
H	-4.78952600924959	-0.45988104647246	-2.13251765926681
C	-6.21724692936756	2.63299016447237	-2.28064820030629
H	-6.26775795377150	3.88589878173360	-4.04228021090056
H	-5.97418476473741	1.17919757111638	-0.69605999772555
H	-6.73465064273569	3.35686684592350	-1.64769863418671

O	-4.07158256258034	0.13313996814528	-6.13649916490729
C	-5.02349797589105	0.08740687999752	-7.08997445121974
O	-4.76248572691025	0.56578378389435	-8.17174995753002
C	-6.34379985415549	-0.56293675687682	-6.76061570185041
H	-6.80705438387368	-0.09596562352596	-5.87862488366344
H	-7.00771488505429	-0.45862167008879	-7.62669197447178
H	-6.20230474753343	-1.63226455159831	-6.53749167764799
N	-2.88118010369135	2.96166359502963	-1.39368324281777
C	-1.90861648913817	2.39718466512449	-5.31572476106174
C	-0.56520556253264	2.03667309586130	-5.64935117390693
C	0.46700783548675	2.83590939740362	-5.17380989088881
C	0.19799395323360	3.98709575787473	-4.39994401120610
C	-1.09999504457932	4.37480558600470	-4.08555376514186
C	-2.18092382927185	3.55739972874927	-4.55105702464129
H	-2.72014310118355	1.86898922716853	-5.81237928353045
H	1.50456010512548	2.58863718617983	-5.40130424246356
H	1.03411296160488	4.60131485623036	-4.05398476462194
H	-3.20208543807099	3.93840293354043	-4.48222733558025
C	-1.37499433926005	5.61809607387446	-3.28425884972927
H	-2.31697317117563	6.09471098802372	-3.59762707999504
H	-1.47365293694975	5.37959800397210	-2.21223031546036
H	-0.55903730594401	6.34884305200765	-3.38647138120423
C	-0.34833824869727	0.82516299811970	-6.53662038346677
H	-0.99712353259780	0.03214030167530	-6.12908014655716

C	1.08906607458376	0.30149145855805	-6.53524178743882
H	1.44278355231472	0.09193868318999	-5.51358214682741
H	1.14939983587210	-0.63292101752891	-7.11490944703295
H	1.78582871325769	1.02089240666826	-6.99634742407015
C	-0.83018507814013	1.11211590830394	-7.97128734923796
H	-0.68906900736424	0.22188931233668	-8.60571496302418
H	-1.89877420657707	1.37249423476304	-7.99729005078581
H	-0.25831672430590	1.94315928192083	-8.41690416444366

13. References

1) Huang, X.; Xu, S.; Tan, Q.; Gao, M.; Li, M.; Xu, B. *Chem. Commun.* **2014**, *50*, 1465–1468.

2) Ackermann, L.; Lygin, *Org. Lett.* **2012**, *14*, 764–767.

3) Lv, S.; Han, X.; Wang, J.-Y.; Zhou, M.; Wu, Y.; Ma, L.; Niu, L.; Gao, W.; Zhou, J.; Hu, W.; Cui, Y.; Chen, J. *Angew. Chem. Int. Ed.* **2020**, *59*, 11583–11590.

4) Wang, Y.; Jia, D.; Zeng, J.; Liu, Y.; Bu, X.; Yang, X. *Org. Lett.* **2021**, *23*, 7740–7745.

5) Yan, X.; Ye, R.; Sun, H.; Zhong, J.; Xiang, H.; Zhou, X *Org. Lett.* **2019**, *21*, 7455–7459.

6) Petrone, D. A.; Isomura, M.; Franzoni, I.; Rössler, S. L.; Carreira, E. M *J. Am. Chem. Soc.* **2018**, *140*, 4697–4704.

7) Zhai, Y.; Zhang, X.; Ma, S.; *Chem. Sci*. **2021**, *12*, 11330-11337.

8) Zhao, Z.; Murphy G. K. Beilstein *J. Org. Chem*. **2018**, *14*, 796–802.

9) Singh, A.; Shukla, R. K.; Volla, C. M. R. *Chem. Sci.* **2022**, *13*, 2043–2049.

10) Shukla, R. K.; Nair, A. M.; Khan, S.; Volla, C. M. R. *Angew. Chem. Int. Ed*. **2020**, *59*, 17042-17048.

11) Jagtap, R. A.; Vinod, C. P.; Punji, B. *ACS Catal.* **2019**, *9*, 431–441.

12) Kim, Y. L.; Park, S.-A.; Kim, J. H. *Eur. J. Org. Chem.* **2020**, *2020*, 4026–4030.

13) Yin, L.; Wang, Y.; Sun, M.; Shi, F. *Adv. Synth. Catal.* **2016**, *358*, 1093–1102.

14) Li, X.-S.; Han, Y.-P.; Zhu, X.-Y.; Xia, Y.; Wei, W.-X.; Li, M.; Liang, Y.-M. *Adv. Synth. Catal*. **2018**, *360*, 4441–4445.

15) (a) Venkatraj, M.; Ariën, K. K.; Heeres, J.; Joossens, J.; Dirié, B.; Lyssens, S.; Michiels, J.; Cos, P.; Lewi, P. J.; Vanham, G.; Maes, L.; Van der Veken, P.; Augustyns, K. *Bioorg. Med.*

Chem. **2014**, *22*, 5241–5248. (b) Ma, J.; Qiao, A.; Wang, B.; Li, Z.; Zhao, F.; Wu, J. *ACS Catal.* **2025**, *15*, 2896–2903.

16) Kristensen, S. K.; Eikeland, E. Z.; Taarning, E.; Lindhardt, A. T.; Skrydstrup, T. *Chem. Sci.* **2017**, *8*, 8094–8105.

17) (a) A. D. Becke, "Density-functional thermochemistry. III. The role of exact exchange," *J. Chem. Phys.*, **98** (1993) 5648-52. (b) S. Grimme, S. Ehrlich and L. Goerigk, "Effect of the damping function in dispersion corrected density functional theory," *J. Comp. Chem.* **32** (2011) 1456-65. (c) F. Weigend, "Accurate Coulomb-fitting basis sets for H to Rn," *Phys. Chem. Chem. Phys.*, **8** (2006) 1057-65. (d) F. Weigend and R. Ahlrichs, "Balanced basis sets of split valence, triple zeta valence and quadruple zeta valence quality for H to Rn: Design and assessment of accuracy," *Phys. Chem. Chem. Phys.*, **7** (2005) 3297-305. (e) V. Barone and M. Cossi, "Quantum calculation of molecular energies and energy gradients in solution by a conductor solvent model," *J. Phys. Chem. A*, **102** (1998) 1995-2001.

18) (a) F. Neese, "The ORCA Program System," *WIREs Comput. Mol. Sci.* 2012, 2, 73-78. (b) F. Neese, "Software update: The ORCA program system—Version 5.0" *WIREs Comput. Mol. Sci.* 2022, 12.

14. Spectroscopic data of the new starting materials

1k

N-(4-(methylsulfonyl)phenyl)pyridin-2-amine

Purified by (petroleum ether/EtOAc: 70/30), 90%, white solid.

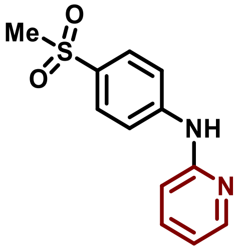

^{1}H NMR (500 MHz, CDCl$_3$): δ 8.26 (dd, *J* = 5.0, 2.7 Hz, 1H), 7.78 (d, *J* = 8.9 Hz, 2H), 7.63 (d, *J* = 8.9 Hz, 2H), 7.55 (td, *J* = 7.8, 2.0 Hz, 1H), 6.90 – 6.82 (m, 2H), 3.02 (s, 3H).

^{13}C NMR (126 MHz, CDCl$_3$): δ 154.3, 148.0, 146.3, 138.0, 131.4, 128.9, 117.6, 116.8, 111.4, 44.9.

HRMS (ESI-TOF) m/z: $[M + H]^+$ Calcd. for $C_{12}H_{13}N_2O_2S$ 249.0692; Found 249.0706

1m

N-(4-tritylphenyl)pyridin-2-amine

Purified by (petroleum ether/EtOAc: 80/20), 86%, purple solid.

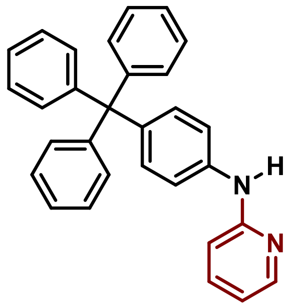

^{1}H NMR (500 MHz, $CDCl_3$): δ 8.19 (dd, *J* = 5.1, 2.5 Hz, 1H), 7.48 (ddd, *J* = 8.7, 7.2, 2.0 Hz, 1H), 7.28 – 7.16 (m, 19H), 6.88 (d, *J* = 8.4 Hz, 1H), 6.72 (dd, *J* = 7.3, 5.8 Hz, 1H), 6.62 (s, 1H).

^{13}C NMR (126 MHz, $CDCl_3$): δ 155.9, 148.4, 147.0, 141.1, 138.4, 137.8, 132.1, 131.3, 127.6, 126.0, 118.8, 115.2, 108.7, 64.6.

HRMS (ESI-TOF) m/z: [M + H]+ Calcd. for $C_{30}H_{25}N_2$ 413.2012; Found 413.2003

1o

(E)-3-ethyl-3-(1-(pyridin-2-yl)-2-styryl-1H-indol-5-yl)piperidine-2,6-dione

Purified by (petroleum ether/EtOAc: 60/40), 80%, white solid.

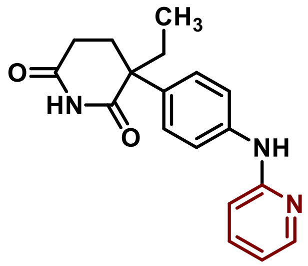

^{1}H NMR (400 MHz, $CDCl_3$) δ 9.39 (s, 1H), 8.27 (s, 1H), 7.50 (t, *J* = 8.0 Hz, 1H), 7.39 – 7.17 (m, 5H), 7.00 – 6.71 (m, 2H), 2.65 – 2.42 (m, 2H), 2.38 – 2.17 (m, 2H), 2.12 – 1.90 (m, 2H), 0.88 (t, *J* = 7.4 Hz, 3H).

^{13}C NMR (126 MHz, $CDCl_3$) δ 176.0, 173.2, 155.7, 148.2, 140.1, 138.1, 132.5, 127.3, 120.1, 115.4, 108.7, 50.6, 33.0, 29.5, 27.2, 9.2.

HRMS (ESI-TOF) m/z: [M + H]+ Calcd. for $C_{18}H_{20}N_3O_2$ 310.1550; Found 310.1547

1p

***N,N'*-((9H-fluorene-9,9-diyl)bis(4,1-phenylene))bis(pyridin-2-amine)**

Purified by (petroleum ether/EtOAc: 50/50), 72%, white solid.

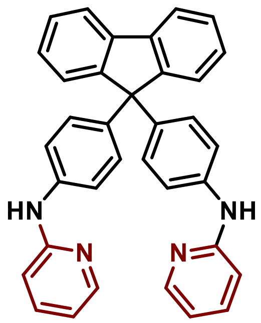

^{1}H NMR (500 MHz, $CDCl_3$): δ 8.16 (dd, *J* = 5.1, 2.1 Hz, 2H), 7.77 (d, *J* = 7.5 Hz, 2H), 7.48 – 7.40 (m, 4H), 7.36 (t, *J* = 7.1 Hz, 2H), 7.28 (d, *J* = 7.5 Hz, 2H), 7.21 – 7.14 (m, 8H), 6.82 (d, *J* = 8.4 Hz, 2H), 6.73 – 6.67 (m, 2H), 6.63 (s, 2H).

^{13}C NMR (126 MHz, $CDCl_3$): δ 156.0, 151.6, 148.5, 140.2, 140.2, 139.1, 137.8, 129.1, 127.8, 127.6, 126.2, 120.3, 120.0, 115.1, 108.5, 64.7.

HRMS (ESI-TOF) m/z: [M + H]+ Calcd. for $C_{35}H_{27}N_4$ 503.2230; Found 503.2221

1q

N,N'-((1,3-phenylenebis(oxy))bis(4,1-phenylene))bis(pyridin-2-amine)

Purified by (petroleum ether/EtOAc: 50/50), 78%, white solid.

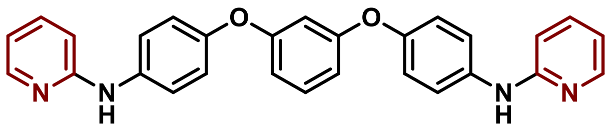

^{1}H NMR (400 MHz, $CDCl_3$) δ 8.17 (dd, *J* = 5.1, 2.7 Hz, 2H), 7.47 (ddd, *J* = 8.7, 7.2, 1.9 Hz, 2H), 7.32 (d, *J* = 8.9 Hz, 4H), 7.23 (m, 1H), 7.11 (s, 2H), 7.02 (d, *J* = 8.9 Hz, 4H), 6.79 (d, *J* = 8.5 Hz, 2H), 6.75 – 6.63 (m, 5H).

^{13}C NMR (101 MHz, $CDCl_3$) δ 159.3, 156.5, 152.1, 148.2, 138.0, 136.4, 130.4, 122.7, 120.5, 114.8, 112.3, 108.3, 108.1.

HRMS (ESI-TOF) m/z: [M + H]+ Calcd. for $C_{28}H_{23}N_2O_2$ 447.1816; Found 447.1811

1s

N,N'-(((propane-2,2-diylbis(4,1-phenylene))bis(oxy))bis(4,1-phenylene))bis(pyridin-2-amine)

Purified by (petroleum ether/EtOAc: 50/50), 62%, white solid.

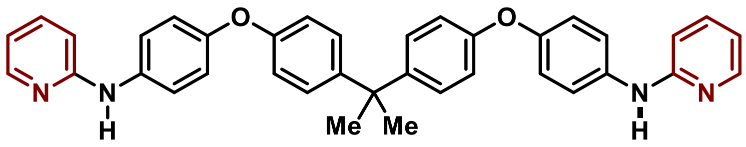

^{1}H NMR (400 MHz, CDCl$_3$) δ 8.18 (dd, *J* = 5.1, 1.0 Hz, 2H), 7.51 – 7.42 (m, 2H), 7.30 (d, *J* = 8.9 Hz, 4H), 7.19 (d, *J* = 8.8 Hz, 4H), 7.01 (d, *J* = 8.9 Hz, 4H), 6.91 (d, *J* = 8.8 Hz, 4H), 6.83 (s, 2H), 6.77 (d, *J* = 8.4 Hz, 2H), 6.70 (ddd, *J* = 7.2, 5.0, 1.0 Hz, 2H), 1.68 (s, 6H).

^{13}C NMR (126 MHz, CDCl$_3$) δ 156.7, 155.7, 152.9, 148.4, 145.4, 137.8, 136.0, 128.1, 122.9, 120.1, 117.8, 114.8, 107.9, 42.2, 31.2.

HRMS (ESI-TOF) m/z: [M + H]$^+$ Calcd. for $C_{37}H_{33}N_4O_2$ 565.2598; Found 565.2594.

15. Spectroscopic data of the final Products:

(E)-N-(2-(nona-1,3-dien-2-yl)phenyl)pyridin-2-amine

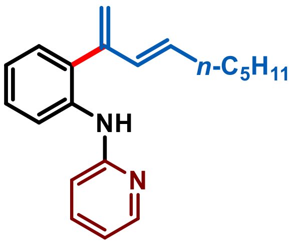

3, 78%

Purified by (petroleum ether/EtOAc:95/5), 45.6 mg, 78%, colourless oil.

^{1}H NMR (400 MHz, CDCl$_3$) δ 8.18 (dd, *J* = 5.0, 2.8 Hz, 1H), 7.78 (d, *J* = 7.0 Hz, 1H), 7.45 (td, *J* = 7.9, 1.9 Hz, 1H), 7.31 (td, *J* = 7.6, 1.8 Hz, 1H), 7.15 (dd, *J* = 7.6, 1.8 Hz, 1H), 7.04 (t, *J* = 8.0 Hz, 1H), 6.82 (d, *J* = 8.4 Hz, 1H), 6.70 (dd, *J* = 7.2, 5.0 Hz, 1H), 6.47 (s, 1H), 6.29 (d, *J* = 15.6 Hz, 1H), 5.34 (s, 1H), 5.05 (s, 1H), 2.00 – 1.94 (m, 2H), 1.21 – 1.11 (m, 6H), 0.80 (t, *J* = 7.0 Hz, 3H).

^{13}C NMR (101 MHz, CDCl$_3$) δ 156.2, 148.5, 145.8, 137.8, 137.6, 135.6, 131.8, 130.9, 130.5, 128.2, 122.4, 120.1, 117.5, 115.1, 108.5, 32.8, 31.5, 28.8, 22.6, 14.1.

HRMS **(ESI-TOF)** m/z: [M + H]$^+$ calcd for $C_{20}H_{25}N_2$ 293.2012, found 293.2009.

(E)-2-(hept-1-en-1-yl)-1-(pyridin-2-yl)-1H-indole

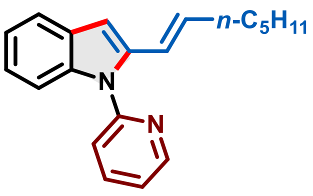

4, 66%

Purified by (petroleum ether/EtOAc: 95/5), 38.3 mg, 66%, light yellow liquid.

^{1}H NMR (500 MHz, CDCl$_3$) δ 8.69 (dd, *J* = 4.8, 1.8 Hz, 1H), 7.87 (td, *J* = 7.7, 1.9 Hz, 1H), 7.61 – 7.52 (m, 1H), 7.48 – 7.43 (m, 1H), 7.37 (d, *J* = 8.0 Hz, 1H), 7.32 (dd, *J* = 7.4, 4.9 Hz, 1H), 7.18 – 7.07 (m, 1H), 6.75 (s, 1H), 6.33 (d, *J* = 15.9 Hz, 1H), 6.24 (dt, *J* = 15.9, 6.7 Hz,

1H), 2.24 – 2.05 (m, 2H), 1.43 (dt, J = 14.4, 7.1 Hz, 2H), 1.36 – 1.27 (m, 4H), 0.89 (t, J = 6.9 Hz, 3H).

^{13}C NMR (126 MHz, CDCl$_3$) δ 151.6, 149.6, 138.5, 138.2, 137.5, 134.3, 128.9, 122.4, 122.0, 121.7, 121.2, 120.3, 119.9, 110.9, 101.3, 33.3, 31.5, 28.9, 22.6, 14.2.

HRMS (ESI-TOF) m/z: [M + H]$^+$ calcd for $C_{20}H_{23}N_2$ 291.1856, found 291.1856

***(E)-1-(pyridin-2-yl)-2-styryl-1H-indole*[11]**

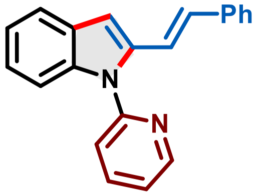

11, 72%

Purified by (petroleum ether/EtOAc: 95/5), 42.6 mg, 72%, light yellow liquid.

^{1}H NMR (400 MHz, CDCl$_3$) δ 8.74 (ddd, J = 4.9, 2.0, 0.9 Hz, 1H), 7.90 (td, J = 7.8, 2.0 Hz, 1H), 7.69 – 7.62 (m, 1H), 7.57 – 7.48 (m, 1H), 7.44 – 7.39 (m, 3H), 7.37 – 7.29 (m, 3H), 7.25 – 7.22 (m, 1H), 7.21 – 7.17 (m, 2H), 7.14 (d, J = 16.3 Hz, 1H), 7.11 (d, J = 16.3 Hz, 1H), 7.00 (s, 1H).

^{13}C NMR (100 MHz, CDCl$_3$) δ 151.5, 149.8, 138.4, 138.2, 138.0, 137.3, 130.7, 128.8, 128.8, 127.9, 126.6, 123.0, 122.2, 121.7, 121.5, 120.6, 118.5, 111.0, 102.5.

HRMS (ESI-TOF) m/z: [M + H]$^+$ calcd for $C_{21}H_{17}N_2$ 297.1386, found 297.1388.

***(E)-5-methoxy-1-(pyridin-2-yl)-2-styryl-1H-indole*[11]**

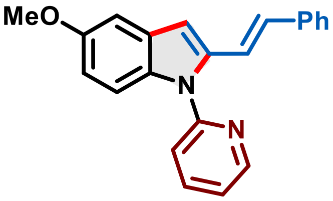

12, 68%

Purified by (petroleum ether/EtOAc: 90/10), 44.4 mg, 68%, light brown liquid.

^{1}H NMR (400 MHz, CDCl$_3$) δ 8.71 (dd, J = 5.8, 2.0 Hz, 1H), 7.88 (td, J = 7.8, 1.9 Hz, 1H), 7.47 – 7.36 (m, 4H), 7.37 – 7.28 (m, 3H), 7.27 – 7.23 (m, 1H), 7.16 – 7.07 (m, 3H), 6.91 (s, 1H), 6.84 (dd, J = 9.0, 2.5 Hz, 1H), 3.88 (s, 3H).

^{13}C NMR (101 MHz, CDCl$_3$) δ 155.4, 151.6, 149.8, 138.7, 138.4, 137.3, 133.2, 130.5, 129.4, 128.8, 127.9, 126.6, 122.0, 121.4, 118.6, 113.1, 112.0, 102.3, 102.2, 56.0.

HRMS (ESI-TOF) m/z: [M + H]$^+$ calcd for $C_{22}H_{19}N_2O$ 327.1492, found 327.1502.

(E)-5-(methylthio)-1-(pyridin-2-yl)-2-styryl-1H-indole

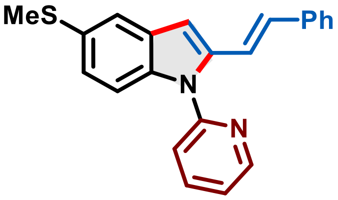

13, 73%

Purified by (petroleum ether/EtOAc: 90/10), 50 mg, 73%, light brown liquid

^{1}H NMR (400 MHz, CDCl$_3$) δ 8.72 (ddd, *J* = 4.9, 1.9, 0.8 Hz, 1H), 7.95 – 7.82 (m, 1H), 7.60 (d, *J* = 1.7 Hz, 1H), 7.46 – 7.43 (m, 1H), 7.42 – 7.38 (m, 3H), 7.37 – 7.34 (m, 1H), 7.34 – 7.29 (m, 2H), 7.23 (dt, *J* = 6.2, 1.8 Hz, 1H), 7.19 (dd, *J* = 8.6, 1.9 Hz, 1H), 7.13 (d, *J* = 16.8 Hz, 1H), 7.05 (d, *J* = 16.8 Hz, 1H), 6.92 (s, 1H), 2.52 (d, *J* = 10.0 Hz, 3H).

^{13}C NMR (101 MHz, CDCl$_3$) δ 151.2, 149.8, 138.7, 138.5, 137.1, 136.6, 131.1, 130.0, 129.6, 128.8, 128.0, 126.7, 124.5, 122.3, 121.5, 120.5, 118.2, 111.6, 101.8, 18.5.

HRMS (ESI-TOF) m/z: $[M + H]^+$ calcd for $C_{22}H_{19}N_2S$ 343.1263, found 343.1276.

(E)-5-chloro-1-(pyridin-2-yl)-2-styryl-1H-indole[11]

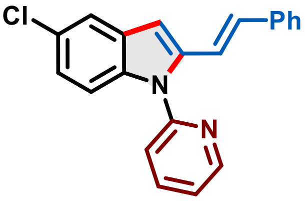

14, 70%

Purified by (petroleum ether/EtOAc: 95/5), 46.3 mg, 70%, light brown liquid.

^{1}H NMR (500 MHz, CDCl$_3$) δ 8.73 (dd, *J* = 4.9, 2.1 Hz, 1H), 7.91 (td, *J* = 7.8, 2.1 Hz, 1H), 7.59 (d, *J* = 2.3 Hz, 1H), 7.44 – 7.36 (m, 5H), 7.32 (t, *J* = 7.6 Hz, 2H), 7.26 – 7.23 (m, 1H), 7.17 – 7.10 (m, 2H), 7.03 (d, *J* = 16.3 Hz, 1H), 6.91 (s, 1H).

^{13}C NMR (126 MHz, CDCl$_3$) δ 151.1, 149.9, 139.5, 138.6, 137.0, 136.3, 131.6, 129.9, 128.9, 128.2, 127.0, 126.7, 123.1, 122.5, 121.6, 119.9, 118.0, 112.2, 101.7.

HRMS (ESI-TOF) m/z: $[M + H]^+$ calcd for $C_{21}H_{16}ClN_2$ 331.0997, found 331.1003.

(E)-5-bromo-1-(pyridin-2-yl)-2-styryl-1H-indole[11]

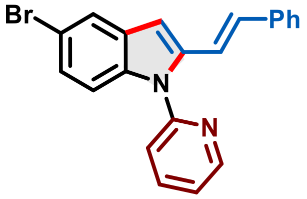

15, 68%

Purified by (petroleum ether/EtOAc: 95/5), 51 mg, 68%, light brown liquid.

^{1}H NMR (400 MHz, CDCl$_3$) δ 8.73 (dd, *J* = 5.5, 1.9 Hz, 1H), 7.90 (td, *J* = 7.8, 2.0 Hz, 1H), 7.75 (d, *J* = 1.8 Hz, 1H), 7.42 – 7.37 (m, 5H), 7.34 (d, *J* = 7.1 Hz, 2H), 7.27 – 7.24 (m, 2H), 7.14 (d, *J* = 16.3 Hz, 1H), 7.03 (d, *J* = 16.3 Hz, 1H), 6.90 (s, 1H).

^{13}C NMR (101 MHz, CDCl$_3$) δ 151.0, 149.9, 139.3, 138.6, 137.0, 136.6, 131.7, 130.5, 128.8, 128.2, 126.7, 125.7, 123.0, 122.5, 121.6, 117.9, 114.6, 112.6, 101.5.

HRMS (ESI-TOF) m/z: [M + H]$^+$ calcd for $C_{21}H_{16}BrN_2$ 375.0491, found 375. 0507

(E)-6-chloro-1-(pyridin-2-yl)-2-styryl-1H-indole

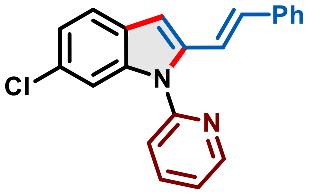

16, 74%

Purified by (petroleum ether/EtOAc: 95/5), 49 mg, 74%, light brown liquid.

^{1}H NMR (500 MHz, CDCl$_3$) δ 8.74 (dd, *J* = 5.6, 2.2 Hz, 1H), 7.92 (td, *J* = 7.8, 2.2 Hz, 1H), 7.67 (s, 1H), 7.49 (d, *J* = 8.4 Hz, 2H), 7.44 – 7.36 (m, 4H), 7.33 (t, *J* = 7.7 Hz, 1H), 7.29 – 7.21 (m, 2H), 7.13 (d, *J* = 16.2 Hz, 1H), 7.01 (d, *J* = 16.2 Hz, 1H), 6.94 (s, 1H).

^{13}C NMR (126 MHz, CDCl$_3$) δ 150.9, 150.0, 138.8, 138.7, 138.6, 137.0, 131.4, 128.8, 128.1, 127.6, 126.7, 124.8, 122.6, 121.7, 121.6, 118.0, 116.5, 114.1, 102.3.

HRMS (ESI-TOF) m/z: [M + H]$^+$ calcd for $C_{21}H_{16}ClN_2$ 331.0997, found 331.1005.

(E)-6-bromo-1-(pyridin-2-yl)-2-styryl-1H-indole

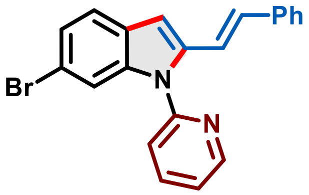

17, 71%

Purified by (petroleum ether/EtOAc: 95/5), 53.3 mg, 71%, light brown liquid.

^{1}H NMR (400 MHz, CDCl$_3$) δ 8.74 (d, *J* = 3.7 Hz, 1H), 7.99 – 7.85 (m, 1H), 7.66 (d, *J* = 20.1 Hz, 2H), 7.42 – 7.38 (m, 4H), 7.36 – 7.30 (m, 3H), 7.28 – 7.24 (m, 1H), 7.14 (d, *J* = 16.3 Hz, 1H), 6.98 (d, *J* = 16.3 Hz, 1H), 6.88 (s, 1H).

^{13}C NMR (101 MHz, CDCl$_3$) δ 150.7, 150.0, 140.0, 138.8, 136.8, 136.6, 132.1, 128.9, 128.4, 128.3, 126.8, 126.6, 125.4, 122.8, 121.4, 121.3, 117.6, 112.8, 101.5.

HRMS (ESI-TOF) m/z: [M + H]$^+$ calcd for $C_{21}H_{16}BrN_2$ 375.0491, found 375. 0489.

(E)-5,6-dichloro-1-(pyridin-2-yl)-2-styryl-1H-indole

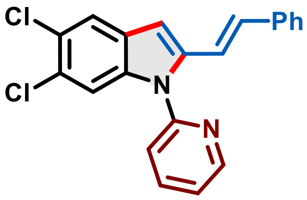

18, 76%

Purified by (petroleum ether/EtOAc: 95/5), 55.5 mg, 76%, light brown liquid.

^{1}H NMR (400 MHz, CDCl$_3$) δ 8.74 (dd, *J* = 4.7, 1.4 Hz, 1H), 7.93 (td, *J* = 7.8, 1.9 Hz, 1H), 7.69 (s, 1H), 7.63 (s, 1H), 7.42 – 7.37 (m, 4H), 7.37 – 7.28 (m, 3H), 7.14 (d, *J* = 16.3 Hz, 1H), 6.98 (d, *J* = 16.3 Hz, 1H), 6.87 (s, 1H).

^{13}C NMR (101 MHz, CDCl$_3$) δ 150.7, 150.0, 140.1, 138.8, 136.8, 136.7, 132.2, 128.9, 128.4, 128.3, 126.8, 126.7, 125.4, 122.8, 121.5, 121.3, 117.6, 112.8, 101.5.

HRMS (ESI-TOF) m/z: [M + H]$^+$ calcd for $C_{21}H_{15}Cl_2N_2$ 365.0607, found 365.0620.

(E)-6-chloro-5-fluoro-1-(pyridin-2-yl)-2-styryl-1H-indole

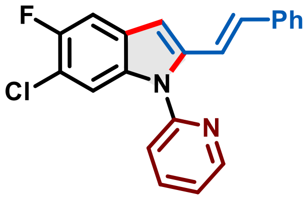

19, 67%

Purified by (petroleum ether/EtOAc: 95/5), 46.7 mg, 67%, light brown liquid.

^{1}H NMR (500 MHz, CDCl$_3$) δ 8.74 (dd, *J* = 4.9, 2.1 Hz, 1H), 7.92 (td, *J* = 7.7, 2.1 Hz, 1H), 7.57 (d, *J* = 6.4 Hz, 1H), 7.42 – 7.38 (m, 3H), 7.36 (dd, 2H), 7.34 – 7.30 (m, 2H), 7.28 – 7.24 (m, 1H), 7.14 (d, *J* = 16.2 Hz, 1H), 6.99 (d, *J* = 16.2 Hz, 1H), 6.90 (s, 1H).

^{13}C NMR (126 MHz, CDCl$_3$) δ 154.1 (d, *J* = 239.4 Hz), 150.8, 150.0, 140.0, 138.8, 136.8, 134.2, 131.9, 128.9, 128.3, 127.7 (d, *J* = 9.2 Hz), 126.7, 122.7, 121.4, 117.7, 116.4 (d, *J* = 21.2 Hz), 112.6, 106.3 (d, *J* = 23.5 Hz), 101.9 (d, *J* = 4.5 Hz).

^{19}F NMR (377 MHz, CDCl$_3$) δ -124.92.

HRMS (ESI-TOF) m/z: $[M + H]^+$ calcd for $C_{21}H_{15}ClFN_2$ 349.0902, found 349.0914.

(E)-5-(methylsulfonyl)-1-(pyridin-2-yl)-2-styryl-1H-indole

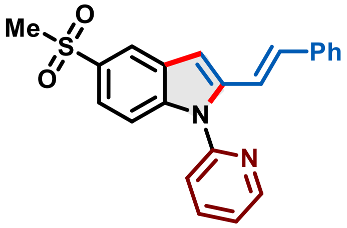

20, 60%

Purified by (petroleum ether/EtOAc: 80/20), 44.9 mg, 60%, light brown liquid.

^{1}H NMR (400 MHz, CDCl$_3$) δ 8.80 (d, *J* = 4.9 Hz, 1H), 8.31 (s, 1H), 8.00 (t, *J* = 7.9 Hz, 1H), 7.75 (d, *J* = 8.5 Hz, 1H), 7.63 (d, *J* = 8.8 Hz, 1H), 7.53 – 7.43 (m, 4H), 7.38 (t, *J* = 7.5 Hz, 2H), 7.32 (d, *J* = 6.4 Hz, 1H), 7.24 (d, *J* = 16.3 Hz, 1H), 7.11 (s, 1H), 7.04 (d, *J* = 16.3 Hz, 1H), 3.13 (s, 3H).

^{13}C NMR (126 MHz, CDCl$_3$) δ 150.5, 150.1, 141.0, 140.0, 138.9, 136.6, 133.3, 132.9, 129.2, 128.9, 128.5, 126.8, 123.2, 121.9, 121.2, 121.1, 117.2, 111.8, 102.7, 45.3.

HRMS (ESI-TOF) m/z: $[M + H]^+$ calcd for $C_{21}H_{19}N_2O_2S$ 375.1162, found 375.1171.

(E)-1-(1-(pyridin-2-yl)-2-styryl-1H-indol-5-yl)ethan-1-one

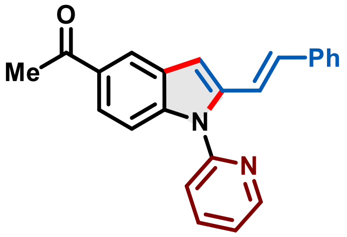

21, 62%

Purified by (petroleum ether/EtOAc: 85/15), 42 mg, 62%, light brown liquid.

^{1}H NMR (400 MHz, CDCl$_3$) δ 8.75 (dd, *J* = 4.8, 1.0 Hz, 1H), 8.29 (d, *J* = 1.5 Hz, 1H), 7.94 (td, *J* = 7.7, 1.9 Hz, 1H), 7.84 (dd, *J* = 8.8, 1.7 Hz, 1H), 7.49 (d, *J* = 8.7 Hz, 1H), 7.42 (t, *J* = 7.6 Hz, 4H), 7.33 (dd, *J* = 8.2, 6.8 Hz, 2H), 7.28 (dd, *J* = 5.7, 4.5 Hz, 1H), 7.16 (d, *J* = 16.2 Hz, 1H), 7.06 (s, 1H), 7.02 (d, *J* = 16.3 Hz, 1H), 2.68 (s, 3H).

^{13}C NMR (101 MHz, CDCl$_3$) δ 198.3, 150.8, 150.0, 140.4, 139.9, 138.7, 136.9, 131.9, 131.3, 128.9, 128.3, 128.2, 126.7, 123.2, 122.9, 122.6, 121.8, 117.7, 110.9, 103.2, 26.8.

HRMS (ESI-TOF) m/z: $[M + Na]^+$ calcd for $C_{23}H_{18}N_2ONa$ 361.1311, found 361.1329.

(E)-1-(pyridin-2-yl)-2-styryl-1,9-dihydroindeno[1,2-f]indole

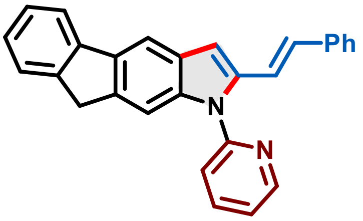

22, 75%

Purified by (petroleum ether/EtOAc: 95/5), 57.6 mg, 77%, light brown liquid.

^{1}H NMR (500 MHz, CDCl$_3$) δ 8.77 (dd, *J* = 4.8, 1.1 Hz, 1H), 8.00 (s, 1H), 7.92 (td, *J* = 7.8, 1.8 Hz, 1H), 7.84 (d, *J* = 7.5 Hz, 1H), 7.68 (s, 1H), 7.51 (d, *J* = 7.4 Hz, 1H), 7.46 – 7.35 (m, 5H), 7.33 (t, *J* = 7.6 Hz, 2H), 7.28 – 7.23 (m, 2H), 7.16 – 7.08 (m, 2H), 7.06 (s, 1H), 3.96 (s, 2H).

^{13}C NMR (126 MHz, CDCl$_3$) δ 151.6, 149.8, 143.3, 142.4, 139.4, 138.5, 138.2, 138.2, 137.3, 136.3, 130.4, 128.8, 128.3, 127.8, 126.8, 126.6, 126.0, 125.1, 122.2, 121.8, 119.5, 118.5, 111.1, 107.5, 102.9, 36.9.

HRMS (ESI-TOF) m/z: $[M + H]^+$ calcd for $C_{28}H_{21}N_2$ 385.1699, found 385.1717.

(E)-1-(pyridin-2-yl)-2-styryl-5-trityl-1H-indole

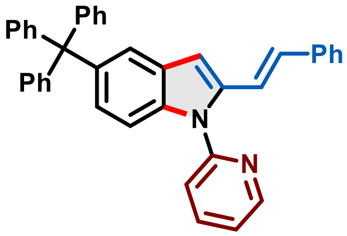

23, 77%

Purified by (petroleum ether/EtOAc: 90/10), 83 mg, 77%, white solid.

^{1}H NMR (400 MHz, CDCl$_3$) δ 8.70 (dd, *J* = 4.9, 2.1 Hz, 1H), 7.87 (td, *J* = 7.8, 2.0 Hz, 1H), 7.52 (d, *J* = 2.0 Hz, 1H), 7.44 – 7.35 (m, 5H), 7.31 – 7.24 (m, 13H), 7.23 – 7.17 (m, 5H), 7.09 – 7.03 (m, 2H), 6.97 (dd, *J* = 8.8, 2.0 Hz, 1H), 6.89 (s, 1H).

^{13}C NMR (101 MHz, CDCl$_3$) δ 151.4, 149.7, 147.4, 140.3, 138.4, 138.1, 137.3, 136.0, 131.5, 130.5, 128.8, 128.3, 127.9, 127.5, 127.5, 126.6, 125.9, 122.1, 122.1, 121.4, 118.5, 110.1, 103.1, 65.1.

HRMS (ESI-TOF) m/z: $[M + H]^+$ calcd for $C_{40}H_{31}N_2$ 539.2482, found 539.2472.

(E)-6-chloro-2-(hept-1-en-1-yl)-7-methyl-1-(pyridin-2-yl)-1H-indole

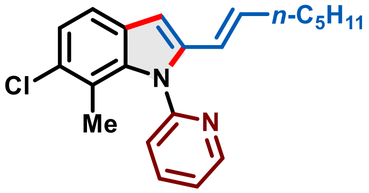

24, 51%

Purified by (petroleum ether/EtOAc: 95/5), 34.5 mg, 51%, light yellow liquid.

^{1}H NMR (400 MHz, CDCl$_3$) δ 8.68 (dd, *J* = 5.3, 2.4 Hz, 1H), 7.87 (td, *J* = 7.6, 2.0 Hz, 1H), 7.47 – 7.42 (m, 1H), 7.34 (d, *J* = 8.4 Hz, 1H), 7.29 (d, *J* = 7.9 Hz, 1H), 7.14 (d, *J* = 8.4 Hz, 1H), 6.66 (s, 1H), 6.20 (dt, *J* = 15.8, 7.1 Hz, 1H), 5.93 (d, *J* = 15.8 Hz, 1H), 2.11 – 2.03 (m, 2H), 1.85 (s, 3H), 1.37 – 1.33 (m, 2H), 1.27 – 1.23 (m, 4H), 0.86 (t, *J* = 7.0 Hz, 3H).

^{13}C NMR (101 MHz, CDCl$_3$) δ 153.0, 149.4, 140.8, 138.0, 137.5, 134.9, 128.9, 128.2, 124.6, 123.7, 122.5, 119.4, 119.0, 118.6, 99.9, 33.3, 31.4, 28.8, 22.6, 15.6, 14.2.

HRMS (ESI-TOF) m/z: $[M + H]^+$ calcd for $C_{21}H_{24}ClN_2$ 339.1623, found 339.1619.

(E)-2-(pent-1-en-1-yl)-1-(pyridin-2-yl)-1H-indole

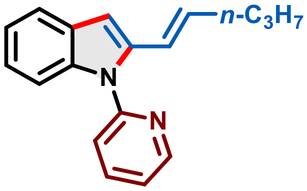

25, 71%

Purified by (petroleum ether/EtOAc: 95/5), 37.2 mg, 71%, light yellow liquid.

^{1}H NMR (400 MHz, CDCl$_3$) δ 8.69 (ddd, *J* = 4.9, 1.9, 0.8 Hz, 1H), 7.90 – 7.85 (td, *J* = 7.8, 2.0 Hz, 1H), 7.61 – 7.55 (m, 1H), 7.49 – 7.44 (m, 1H), 7.39 – 7.35 (m, 1H), 7.34 – 7.29 (m, 1H), 7.17 – 7.10 (m, 2H), 6.76 (s, 1H), 6.34 (d, *J* = 15.9 Hz, 1H), 6.24 (dt, *J* = 15.9, 6.6 Hz, 1H), 2.15 (td, *J* = 7.7, 1.0 Hz, 2H), 1.53 – 1.37 (m, 2H), 0.92 (t, *J* = 7.4 Hz, 3H).

^{13}C NMR (101 MHz, CDCl$_3$) δ 151.6, 149.6, 138.5, 138.2, 137.6, 134.0, 128.9, 122.4, 122.0, 121.7, 121.2, 120.3, 120.1, 110.9, 101.3, 35.4, 22.5, 13.8.

HRMS (ESI-TOF) m/z: $[M + H]^+$ calcd for $C_{18}H_{19}N_2$ 263.1543, found 263.1569.

(E)-2-(2-cyclohexylvinyl)-1-(pyridin-2-yl)-1H-indole

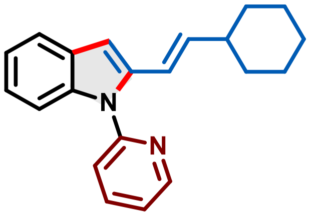

26, 70%

Purified by (petroleum ether/EtOAc:95/5), 42.3 mg, 70%, colourless oil.

^{1}H NMR (500 MHz, $CDCl_3$) δ 8.70 (d, *J* = 3.2 Hz, 1H), 7.86 (td, *J* = 7.7, 2.1 Hz, 1H), 7.59 (dd, *J* = 5.9, 3.3 Hz, 1H), 7.53 – 7.44 (m, 1H), 7.36 (d, *J* = 8.1 Hz, 1H), 7.33 – 7.28 (m, 1H), 7.19 – 7.10 (m, 2H), 6.77 (s, 1H), 6.32 (d, *J* = 16.2 Hz, 1H), 6.21 (dd, *J* = 16.2, 6.8 Hz, 1H), 2.13 – 2.05 (m, 1H), 1.80 – 1.64 (m, 6H), 1.31 – 1.26 (m, 2H), 1.18 – 1.12 (m, 2H).

^{13}C NMR (126 MHz, $CDCl_3$) δ 151.6, 149.5, 139.7, 138.6, 138.2, 137.6, 128.8, 122.3, 121.9, 121.6, 121.1, 120.2, 117.5, 110.9, 101.2, 41.3, 32.8, 26.2, 26.0.

HRMS (ESI-TOF) m/z: $[M + H]^+$ calcd for $C_{21}H_{23}N_2$ 303.1856, found 303.1865.

***(E)-2-(4-fluorostyryl)-1-(pyridin-2-yl)-1H-indole*[11]**

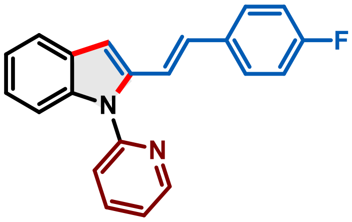

27, 67%

Purified by (petroleum ether/EtOAc: 95/5), 42.1 mg, 67%, light brown liquid.

^{1}H NMR (400 MHz, $CDCl_3$) δ 8.73 (dd, *J* = 4.9, 2.1 Hz, 1H), 7.91 (td, *J* = 7.7, 2.0 Hz, 1H), 7.64 – 7.57 (m, 1H), 7.54 – 7.48 (m, 1H), 7.43 (d, *J* = 7.9 Hz, 1H), 7.39 – 7.34 (m, 3H), 7.22 – 7.16 (m, 2H), 7.09 (d, *J* = 16.2 Hz, 1H), 7.05 – 6.96 (m, 4H).

^{13}C NMR (101 MHz, $CDCl_3$) δ 162.5 (d, *J* = 247.6 Hz), 151.4, 149.8, 138.5, 138.1, 133.5 (d, *J* = 3.3 Hz), 129.4, 128.8, 128.1 (d, *J* = 8.0 Hz), 123.0, 122.2, 121.6, 121.5, 120.7, 118.3, 118.2, 115.7 (d, *J* = 21.9 Hz), 110.9, 102.4.

^{19}F NMR (377 MHz, $CDCl_3$) δ -124.92.

HRMS (ESI-TOF) m/z: $[M + H]^+$ calcd for $C_{21}H_{16}FN_2$ 315.1292, found 315.1288

***(E)-2-(2-([1,1'-biphenyl]-4-yl)vinyl)-1-(pyridin-2-yl)-1H-indole*[11]**

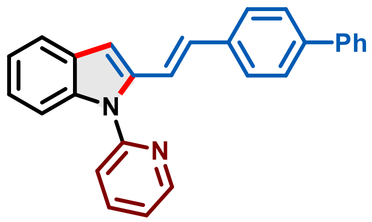

28, 73%

Purified by (petroleum ether/EtOAc: 90/10), 54.3 mg, 76%, white solid.

^{1}H NMR (400 MHz, CDCl$_3$) δ 8.75 (dd, *J* = 4.6, 1.4 Hz, 1H), 7.92 (td, *J* = 7.8, 1.9 Hz, 1H), 7.71 – 7.63 (m, 1H), 7.62 – 7.55 (m, 4H), 7.54 – 7.48 (m, 2H), 7.47 – 7.41 (m, 4H), 7.40 – 7.31 (m, 2H), 7.22 – 7.16 (m, 2H), 7.15 – 7.09 (m, 2H), 7.02 (s, 1H).

^{13}C NMR (101 MHz, CDCl$_3$) δ 151.4, 149.7, 140.7, 140.6, 138.6, 138.2, 138.0, 136.3, 130.2, 128.9, 128.8, 127.5, 127.5, 127.1, 127.0, 123.1, 122.2, 121.7, 121.6, 120.7, 118.5, 111.0, 102.6.

HRMS (ESI-TOF) m/z: $[M + H]^+$ calcd for $C_{27}H_{21}N_2$ 373.1699, found 373.1714.

***(E)-2-(4-chlorostyryl)-1-(pyridin-2-yl)-1H-indole*[11]**

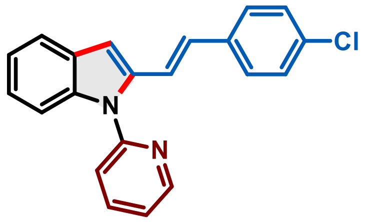

29, 74%

Purified by (petroleum ether/EtOAc: 95/5), 49 mg, 74%, light yellow liquid.

^{1}H NMR (400 MHz, CDCl$_3$) δ 8.73 (dd, *J* = 4.9, 2.8 Hz, 1H), 7.91 (td, *J* = 7.7, 2.0 Hz, 1H), 7.68 – 7.60 (m, 1H), 7.53 – 7.46 (m, 1H), 7.45 – 7.40 (m, 1H), 7.37 (dd, *J* = 7.4, 4.9 Hz, 1H), 7.34 – 7.26 (m, 4H), 7.22 – 7.05 (m, 4H), 6.99 (s, 1H).

^{13}C NMR (101 MHz, CDCl$_3$) δ 151.3, 149.8, 138.5, 138.0, 137.9, 135.8, 133.4, 129.2, 128.9, 128.7, 127.7, 123.2, 122.3, 121.6, 121.6, 120.7, 119.1, 111.0, 102.7.

HRMS (ESI-TOF) m/z: $[M + H]^+$ calcd for $C_{21}H_{16}ClN_2$ 331.0997, found 331.0993

(E)-2-(2-chloro-6-fluorostyryl)-1-(pyridin-2-yl)-1H-indole

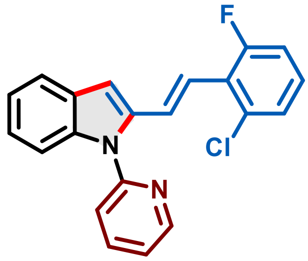

30, 77%

Purified by (petroleum ether/EtOAc: 90/10), 53.7 mg, 77%, white solid.

^{1}H NMR (400 MHz, CDCl$_3$) δ 8.72 (dd, *J* = 4.8, 1.8 Hz, 1H), 7.91 (td, *J* = 7.7, 1.9 Hz, 1H), 7.66 (dd, *J* = 6.5, 2.0 Hz, 1H), 7.59 – 7.47 (m, 1H), 7.48 – 7.31 (m, 3H), 7.26 (d, *J* = 16.6 Hz, 1H), 7.23 – 7.16 (m, 3H), 7.12 – 7.05 (m, 2H), 7.00 – 6.93 (m, 1H).

^{13}C NMR (101 MHz, CDCl$_3$) δ 161.3 (d, *J* = 253.0 Hz), 151.2, 149.7, 138.3, 138.0 (d, *J* = 9.3 Hz), 134.4 (d, *J* = 5.9 Hz), 128.6, 128.0 (d, *J* = 10.2 Hz), 125.8 (d, *J* = 2.9 Hz), 125.7, 125.6, 124.0 (d, *J* = 14.0 Hz), 123.3, 122.1, 121.5, 121.4, 120.8, 120.8, 114.6 (d, *J* = 23.3 Hz).111.0, 103.4.

^{19}F NMR (377 MHz, CDCl$_3$) δ -124.92.

HRMS **(ESI-TOF)** m/z: $[M + H]^+$ calcd for $C_{21}H_{15}ClFN_2$ 349.0902, found 349.0918.

(E)-2-(2,4-dichlorostyryl)-1-(pyridin-2-yl)-1H-indole

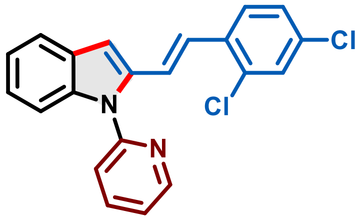

31, 74%

Purified by (petroleum ether/EtOAc: 95/5), 54 mg, 74%, light yellow liquid.

^{1}H NMR (500 MHz, CDCl$_3$) δ 8.66 (dd, *J* = 4.8, 1.3 Hz, 1H), 7.85 (td, *J* = 7.8, 1.9 Hz, 1H), 7.59 (dt, *J* = 8.1, 4.2 Hz, 1H), 7.41 (d, *J* = 7.7 Hz, 1H), 7.39 (d, *J* = 3.3 Hz, 1H), 7.37 – 7.35 (m, 2H), 7.31 – 7.29 (m, 1H), 7.27 (dd, *J* = 7.9, 1.3 Hz, 1H), 7.16 – 7.10 (m, 2H), 7.07 (d, *J* = 7.9 Hz, 1H), 7.04 (d, *J* = 11.7 Hz, 1H), 7.01 (s, 1H).

^{13}C NMR (126 MHz, CDCl$_3$) δ 151.4, 150.0, 138.7, 138.4, 137.8, 137.5, 133.8, 131.6, 129.3, 128.8, 127.3, 126.4, 124.7, 123.6, 122.5, 122.2, 121.8, 121.8, 121.1 111.1, 104.2.

HRMS **(ESI-TOF)** m/z: [M + H]+ calcd for $C_{21}H_{15}Cl_2N_2$ 365.0607, found 365.0633.

(E)-2-(2-(naphthalen-1-yl)vinyl)-1-(pyridin-2-yl)-1H-indole

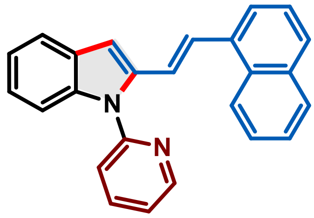

32, 68%

Purified by (petroleum ether/EtOAc: 90/10), 47.1 mg, 68%, yellow liquid.

^{1}H NMR (500 MHz, $CDCl_3$) δ 8.74 (d, *J* = 2.1 Hz, 1H), 8.17 (d, *J* = 8.2 Hz, 1H), 7.92 – 7.85 (m, 3H), 7.79 (d, *J* = 8.2 Hz, 1H), 7.71 – 7.67 (m, 1H), 7.64 (d, *J* = 7.2 Hz, 1H), 7.57 – 7.50 (m, 3H), 7.48 – 7.41 (m, 2H), 7.38 – 7.32 (m, 1H), 7.24 – 7.18 (m, 2H), 7.15 (d, *J* = 15.9 Hz, 1H), 7.10 (s, 1H).

^{13}C NMR (126 MHz, $CDCl_3$) δ 151.5, 149.8, 138.5, 138.4, 138.1, 134.7, 133.8, 131.3, 128.8, 128.7, 128.3, 127.6, 126.3, 126.0, 125.7, 123.8, 123.6, 123.2, 122.2, 121.7, 121.5, 121.3, 120.7, 111.0, 102.9.

HRMS **(ESI-TOF)** m/z: $[M + H]^+$ calcd for $C_{25}H_{19}N_2$ 347.1543, found 347.1541.

(E)-4-(2-(1-(pyridin-2-yl)-1H-indol-2-yl)vinyl)benzonitrile

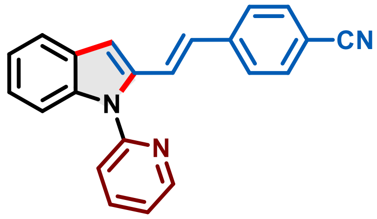

33, 69%

Purified by (petroleum ether/EtOAc: 85/15), 44.3 mg, 69%, white solid.

^{1}H NMR (500 MHz, $CDCl_3$) δ 8.74 (dd, *J* = 4.8, 1.1 Hz, 1H), 7.94 (td, *J* = 7.8, 1.9 Hz, 1H), 7.70 – 7.61 (m, 1H), 7.58 (d, *J* = 8.3 Hz, 2H), 7.51 – 7.41 (m, 4H), 7.41 – 7.35 (m, 1H), 7.24 – 7.16 (m, 3H), 7.10 – 7.05 (m, 2H).

^{13}C NMR (126 MHz, $CDCl_3$) δ 151.3, 150.0, 141.9, 138.7, 138.3, 137.3, 132.7, 128.7, 128.3, 127.0, 123.8, 122.6, 122.2, 121.9, 121.7, 121.1, 119.3, 111.1, 110.7, 103.9.

HRMS **(ESI-TOF)** m/z: $[M + H]^+$ calcd for $C_{22}H_{16}N_3$ 322.1339, found 322.1354.

(E)-1-(pyridin-2-yl)-2-(4-(trifluoromethyl)styryl)-1H-indole

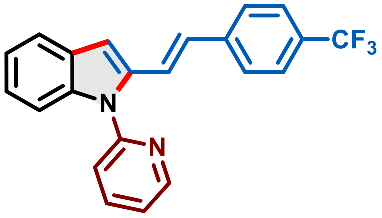

34, 63%

Purified by (petroleum ether/EtOAc: 90/10), 45.9 mg, 63%, white solid.

^{1}H NMR (400 MHz, CDCl$_3$) δ 8.75 (d, *J* = 3.7 Hz, 1H), 7.91 (td, *J* = 7.7, 1.8 Hz, 1H), 7.67 (d, *J* = 7.0 Hz, 1H), 7.57 (d, *J* = 8.3 Hz, 2H), 7.53 – 7.43 (m, 4H), 7.40 – 7.34 (m, 1H), 7.25 – 7.19 (m, 3H), 7.12 (d, *J* = 16.2 Hz, 1H), 7.05 (s, 1H).

^{13}C NMR (101 MHz, CDCl$_3$) δ 151.2, 149.8, 140.7, 138.6, 138.1, 137.5, 129.2 (q, *J* = 32.3 Hz), 128.7, 128.6, 126.6, 125.6 (q, *J* = 3.7 Hz), 123.4, 122.9, 122.3, 121.6, 121.5, 121.0, 120.9, 111.0, 103.3.

^{19}F NMR (376 MHz, CDCl$_3$) δ -62.46.

HRMS **(ESI-TOF)** m/z: $[M + Na]^+$ calcd for $C_{22}H_{16}F_3N_2Na$ 387.1080, found 387.1088.

(E)-2-(2-(phenylethynyl)styryl)-1-(pyridin-2-yl)-1H-indole

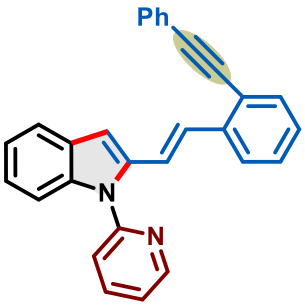

35, 70%

Purified by (petroleum ether/EtOAc: 95/5), 55.5 mg, 70%, light brown liquid.

^{1}H NMR (500 MHz, CDCl$_3$) δ 8.66 (dd, *J* = 4.9, 2.1 Hz, 1H), 7.83 (td, *J* = 7.7, 2.0 Hz, 1H), 7.71 – 7.60 (m, 2H), 7.58 – 7.46 (m, 5H), 7.42 (d, *J* = 8.0 Hz, 1H), 7.41 – 7.34 (m, 3H), 7.32 – 7.27 (m, 2H), 7.24 – 7.14 (m, 4H), 7.05 (s, 1H).

^{13}C NMR (126 MHz, CDCl$_3$) δ 151.4, 149.8, 138.5, 138.4, 138.3, 138.2, 133.0, 131.7, 128.8, 128.6, 128.6, 128.6, 128.6, 127.4, 125.0, 123.4, 123.2, 122.2, 122.2, 121.7, 121.5, 120.8, 120.0, 111.1, 103.2, 94.7, 87.9.

HRMS (ESI-TOF) *m/z* $[M + H]^+$ calcd for $C_{29}H_{21}N_2$ 397.1699; found 397.1706.

(E)-2-(4-(phenylethynyl)styryl)-1-(pyridin-2-yl)-1H-indole

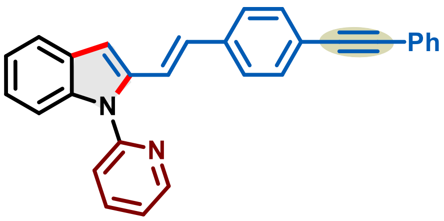

36, 75%

Purified by (petroleum ether/EtOAc: 90/10), 59.4 mg, 75%, white solid.

^{1}H NMR (500 MHz, CDCl$_3$) δ 8.75 (d, *J* = 3.6 Hz, 1H), 7.92 (td, *J* = 7.8, 1.7 Hz, 1H), 7.64 (dd, *J* = 6.0, 2.5 Hz, 1H), 7.55 – 7.52 (m, 2H), 7.49 – 7.42 (m, 4H), 7.40 – 7.32 (m, 6H), 7.23 – 7.16 (m, 2H), 7.11 (d, *J* = 3.9 Hz, 2H), 7.02 (s, 1H).

^{13}C NMR (126 MHz, CDCl$_3$) δ 151.4, 149.8, 138.6, 138.1, 138.0, 137.2, 132.0, 131.7, 129.8, 128.8, 128.5, 128.4, 126.5, 123.4, 123.2, 122.5, 122.3, 121.7, 121.6, 120.8, 119.3, 111.0, 102.9, 90.6, 89.7.

HRMS (ESI-TOF) *m/z* [M + H]$^+$ calcd for $C_{29}H_{21}N_2$ 397.1699; found 397.1705.

(E)-3-propyl-1-(pyridin-2-yl)-2-styryl-1H-indole

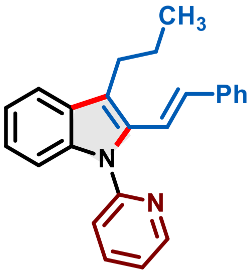

37, 73%

Purified by (petroleum ether/EtOAc: 95/5), 49.4 mg, 73%, light yellow oil.

^{1}H NMR (400 MHz, CDCl$_3$) δ 8.70 (ddd, *J* = 4.9, 2.0, 0.8 Hz, 1H), 7.86 – 7.79 (m, 1H), 7.67 – 7.61 (m, 1H), 7.55 – 7.47 (m, 1H), 7.37 – 7.28 (m, 6H), 7.25 – 7.22 (m, 1H), 7.21 – 7.15 (m, 2H), 7.10 (d, *J* = 16.6 Hz, 1H), 6.52 (d, *J* = 16.6 Hz, 1H), 2.99 – 2.91 (m, 2H), 1.82 (h, *J* = 7.3 Hz, 2H), 1.08 (t, *J* = 7.3 Hz, 3H).

^{13}C NMR (101 MHz, CDCl$_3$) δ 152.4, 149.6, 138.3, 138.0, 137.6, 133.4, 131.5, 129.4, 128.8, 127.7, 126.4, 123.5, 121.9, 121.9, 120.8, 119.5, 119.3, 118.2, 111.0, 27.2, 24.3, 14.6.

HRMS **(ESI-TOF)** m/z: [M + H]$^+$ calcd for $C_{24}H_{23}N_2$ 339.1856, found 339.1846.

(E)-2-(4-(4-(tert-butyl)phenyl)-3-methylbut-1-en-1-yl)-1-(pyridin-2-yl)-1H-indole

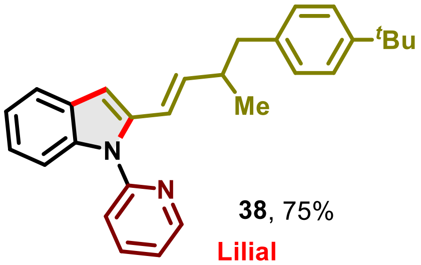

38, 75%

Lilial

Purified by (petroleum ether/EtOAc:95/5), 59.1 mg, 75%, colourless oil.

^{1}H NMR (500 MHz, CDCl$_3$) δ 8.68 (d, *J* = 4.9 Hz, 1H), 7.78 (td, *J* = 7.8, 2.0 Hz, 1H), 7.62 (dd, *J* = 6.3, 2.8 Hz, 1H), 7.57 – 7.51 (m, 1H), 7.34 – 7.28 (m, 3H), 7.22 – 7.14 (m, 3H), 7.11 (d, *J* = 8.2 Hz, 2H), 6.78 (s, 1H), 6.26 (d, *J* = 9.2 Hz, 2H), 2.75 – 2.69 (m, 1H), 2.61 (d, *J* = 15.1 Hz, 2H), 1.35 (s, 9H), 1.09 (d, *J* = 6.3 Hz, 3H).

^{13}C NMR (126 MHz, CDCl$_3$) δ 151.5, 149.5, 148.7, 138.9, 138.3, 138.1, 137.5, 137.4, 129.0, 128.8, 125.1, 122.5, 121.8, 121.5, 121.2, 120.8, 118.6, 111.1, 101.5, 42.9, 39.2, 34.5, 31.6, 19.9.

HRMS **(ESI-TOF)** m/z: [M + H]$^+$ calcd for $C_{28}H_{31}N_2$ 395.2482, found 395.2490.

3,7-dimethyloct-6-en-1-yl (E)-4-(2-(1-(pyridin-2-yl)-1H-indol-2-yl)vinyl)benzoate

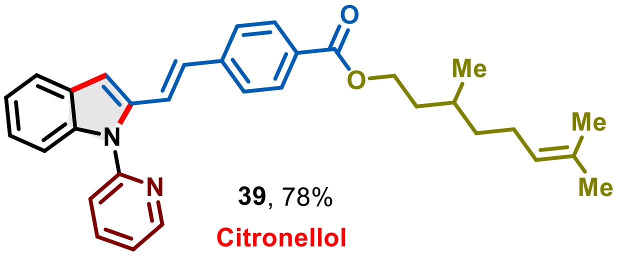

39, 78%

Citronellol

Purified by (petroleum ether/EtOAc: 85/15), 74.6 mg, 78%, light yellow oil.

^{1}H NMR (400 MHz, CDCl$_3$) δ 8.74 (dd, *J* = 4.8, 1.2 Hz, 1H), 7.97 (d, *J* = 8.3 Hz, 2H), 7.92 (td, *J* = 7.8, 1.9 Hz, 1H), 7.65 (dd, *J* = 6.1, 2.5 Hz, 1H), 7.52 – 7.42 (m, 4H), 7.38 (dd, *J* = 7.3, 4.9 Hz, 1H), 7.25 – 7.16 (m, 3H), 7.13 (d, *J* = 16.3 Hz, 1H), 7.05 (s, 1H), 5.10 (t, *J* = 7.1 Hz, 1H), 4.46 – 4.06 (m, 2H), 2.19 – 1.92 (m, 2H), 1.81 (dt, *J* = 12.4, 4.7 Hz, 1H), 1.68 (s, 3H), 1.62 – 1.53 (m, 5H), 1.46 – 1.35 (m, 1H), 1.25 – 1.20 (m, 1H), 0.97 (d, *J* = 6.5 Hz, 3H).

^{13}C NMR (101 MHz, CDCl$_3$) δ 166.5, 151.3, 149.9, 141.6, 138.6, 138.1, 137.7, 131.5, 130.1, 129.42, 129.3, 128.7, 126.3, 124.7, 123.4, 122.4, 121.6, 120.9, 120.9, 111.0, 103.3, 63.6, 37.1, 35.6, 29.7, 25.8, 25.5, 19.6, 17.8.

HRMS **(ESI-TOF)** m/z: [M + H]$^+$ calcd for $C_{32}H_{35}N_2O_2$ 479.2693, found 479.2684.

(Z)-3,7-dimethylocta-2,6-dien-1-yl 4-((E)-2-(5-methoxy-1-(pyridin-2-yl)-1H-indol-2-yl)vinyl)benzoate

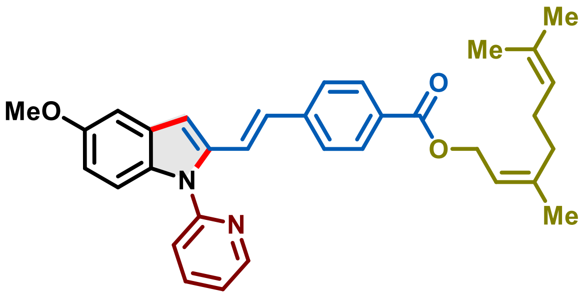

40, 74%
Geraniol

Purified by (petroleum ether/EtOAc: 85/15), 75 mg, 78%, light yellow oil.

^{1}H NMR (500 MHz, CDCl$_3$) δ 8.72 (dd, *J* = 3.9, 0.6 Hz, 1H), 7.98 (d, *J* = 8.2 Hz, 2H), 7.90 (td, *J* = 7.7, 1.6 Hz, 1H), 7.47 – 7.39 (m, 4H), 7.38 – 7.32 (m, 1H), 7.20 (d, *J* = 16.2 Hz, 1H), 7.13 – 7.08 (m, 2H), 6.96 (s, 1H), 6.88 – 6.82 (m, 1H), 5.47 (t, *J* = 6.9 Hz, 1H), 5.10 (t, *J* = 6.5 Hz, 1H), 4.83 (d, *J* = 7.0 Hz, 2H), 3.88 (s, 3H), 2.19 – 2.03 (m, 4H), 1.77 (s, 3H), 1.68 (s, 3H), 1.61 (s, 3H).

^{13}C NMR (126 MHz, CDCl$_3$) δ 166.5, 155.5, 151.4, 149.8, 142.5, 141.7, 138.5, 138.1, 133.4, 132.0, 130.1, 129.4, 129.3, 129.1, 126.3, 123.9, 122.2, 121.3, 120.9, 118.6, 113.6, 112.0, 103.0, 102.2, 62.0, 55.9, 39.7, 26.5, 25.8, 17.9, 16.7.

HRMS **(ESI-TOF)** m/z: [M + H]$^+$ calcd for $C_{33}H_{35}N_2O_3$ 507.2642, found 507.2642

(1S,2R,5S)-2-isopropyl-5-methylcyclohexyl 4-((E)-2-(1-(pyridin-2-yl)-1H-indol-2-yl)vinyl)benzoate

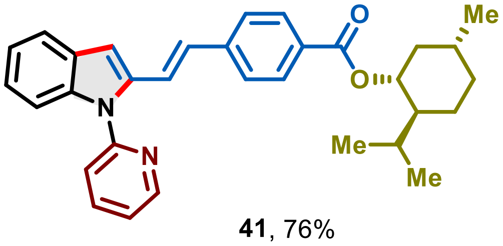

41, 76%
L-Menthol

Purified by (petroleum ether/EtOAc: 85/15), 72.7 mg, 76%, light yellow oil.

^{1}H NMR (500 MHz, CDCl$_3$) δ 8.74 (dd, *J* = 4.8, 1.2 Hz, 1H), 7.99 (d, *J* = 8.3 Hz, 2H), 7.92 (td, *J* = 7.8, 1.9 Hz, 1H), 7.65 (dd, *J* = 6.4, 2.0 Hz, 1H), 7.50 (d, *J* = 7.5 Hz, 1H), 7.46 – 7.42 (m, 3H), 7.38 (dd, *J* = 7.0, 5.3 Hz, 1H), 7.24 – 7.17 (m, 3H), 7.13 (d, *J* = 16.3 Hz, 1H), 7.05 (s, 1H), 4.92 (td, *J* = 10.8, 4.4 Hz, 1H), 2.18 – 2.04 (m, 1H), 1.96 (m, 1H), 1.79 – 1.69 (m, 2H), 1.59 – 1.52 (m, 2H), 1.18 – 1.04 (m, 2H), 0.96 – 0.89 (m, 7H), 0.80 (d, *J* = 6.9 Hz, 3H).

^{13}C NMR (126 MHz, CDCl$_3$) δ 165.9, 151.3, 149.9, 141.5, 138.5, 138.1, 137.7, 130.1, 129.8, 129.3, 128.7, 126.3, 123.4, 122.3, 121.6, 121.6, 120.9, 120.8, 111.0, 103.2, 74.9, 47.4, 41.1, 34.5, 31.6, 26.7, 23.8, 22.2, 20.9, 16.7.

HRMS **(ESI-TOF)** m/z: [M + H]$^+$ calcd for $C_{31}H_{34}N_2O_2$ 479.2693, found 479.2706.

(R)-2,5,7,8-tetramethyl-2-((4R,8R)-4,8,12-trimethyltridecyl)chroman-6-yl 4-((E)-2-(5-(methylsulfonyl)-1-(pyridin-2-yl)-1H-indol-2-yl)vinyl)benzoate

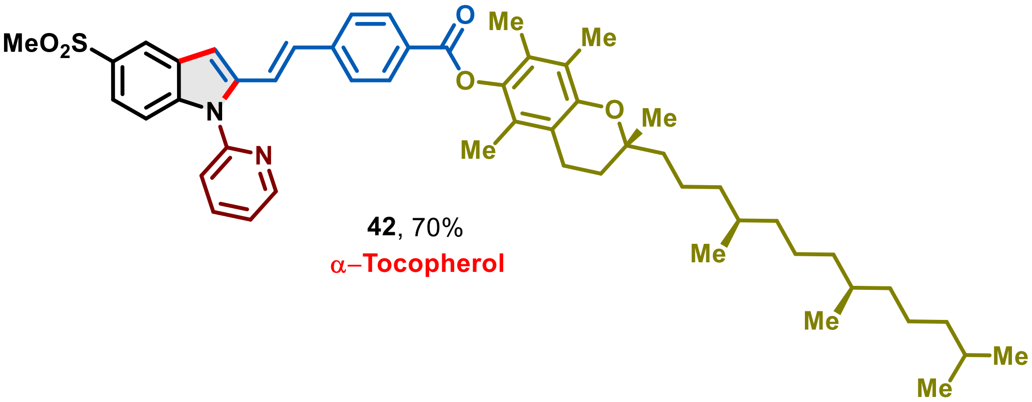

42, 70%
α−Tocopherol

Purified by (petroleum ether/EtOAc: 80/20), 116.3 mg, 70%, light brown liquid.

^{1}H NMR (400 MHz, CDCl$_3$) δ 8.78 (dd, *J* = 4.9, 2.8 Hz, 1H), 8.30 (d, *J* = 1.9 Hz, 1H), 8.20 (d, *J* = 8.5 Hz, 2H), 8.00 (td, *J* = 7.8, 1.9 Hz, 1H), 7.73 (dd, *J* = 8.7, 1.9 Hz, 1H), 7.59 (d, *J* = 8.8 Hz, 1H), 7.54 (d, *J* = 8.4 Hz, 2H), 7.50 – 7.43 (m, 2H), 7.25 (d, *J* = 5.3 Hz, 1H), 7.19 – 7.13 (m, 2H), 3.10 (s, 3H), 2.62 (t, *J* = 6.8 Hz, 2H), 2.12 (s, 3H), 2.05 (s, 3H), 2.01 (s, 3H), 1.88 – 1.74 (m, 3H), 1.56 – 1.50 (m, 2H), 1.43 – 1.37 (m, 3H), 1.29 – 1.24 (m, 10H), 1.17 – 1.07 (m, 6H), 0.89 – 0.84 (m, 14H).

^{13}C NMR (101 MHz, CDCl$_3$) δ 164.9, 150.3, 150.2, 149.6, 141.5, 140.7, 140.3, 140.1, 139.0, 133.5, 131.3, 130.8, 129.1, 128.3, 127.0, 126.8, 125.2, 123.4, 123.3, 121.8, 121.6, 121.4, 119.9, 117.6, 111.9, 103.6, 75.2, 45.3, 39.5, 37.6, 37.5, 37.4, 32.9, 32.9, 32.8, 28.1, 24.9, 24.9, 24.6, 22.8, 22.8, 21.2, 20.8, 19.9, 19.8, 19.8, 19.7, 13.2, 12.3, 12.0.

HRMS **(ESI-TOF)** m/z: [M + H]$^+$ calcd for $C_{52}H_{67}N_2O_5S$ 830.4765, found 830.4728.

(8S,10R,13R,14S)-10,13-dimethyl-17-((R)-6-methylheptan-2-yl)-2,3,4,7,8,9,10,11,12,13,14,15,16,17-tetradecahydro-1H-cyclopenta[a]phenanthren-3-yl 4-((E)-2-(5-(methylsulfonyl)-1-(pyridin-2-yl)-1H-indol-2-yl)vinyl)benzoate

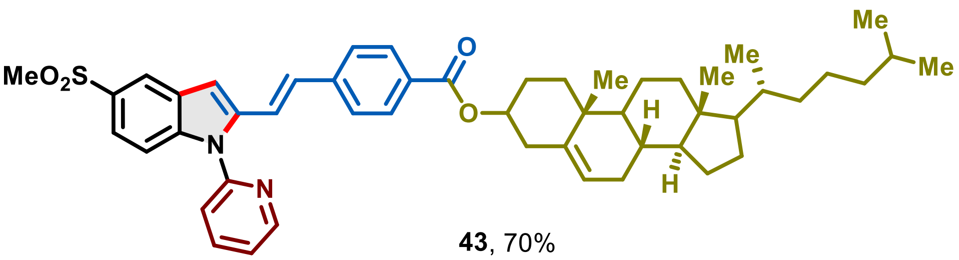

43, 70%
Cholesterol

Purified by (petroleum ether/EtOAc: 85/15), 110.1 mg, 70%, light yellow oil.

^{1}H NMR (400 MHz, CDCl$_3$) δ 8.77 (dd, *J* = 4.9, 1.9 Hz, 1H), 8.28 (d, *J* = 1.8 Hz, 1H), 8.10 – 7.86 (m, 3H), 7.72 (dd, *J* = 8.7, 1.8 Hz, 1H), 7.58 (d, *J* = 8.8 Hz, 1H), 7.53 – 7.37 (m, 3H), 7.22 (d, *J* = 16.2 Hz, 1H), 7.17 – 6.98 (m, 2H), 5.41 (dd, *J* = 5.0, 1.9 Hz, 1H), 5.10 – 4.36 (m, 1H), 3.09 (s, 3H), 2.45 (d, *J* = 8.2 Hz, 2H), 2.07 – 1.64 (m, 1H), 1.62 – 1.39 (m, 1H), 1.29 – 1.11 (m, 1H), 1.06 (s, 3H), 1.03 – 0.97 (m, 1H), 0.92 (d, *J* = 6.5 Hz, 3H), 0.87 (d, *J* = 1.9 Hz, 3H), 0.86 (d, *J* = 1.8 Hz, 3H), 0.68 (s, 3H).

^{13}C NMR (101 MHz, CDCl$_3$) δ 165.7, 150.3, 150.2, 140.8, 140.4, 140.1, 139.7, 139.0, 133.5, 131.5, 130.3, 130.1, 128.3, 126.5, 123.4, 123.0, 121.8, 121.5, 121.4, 119.5, 111.8, 103.4, 74.8, 56.8, 56.2, 50.1, 45.3, 42.4, 39.8, 39.6, 38.3, 37.1, 36.8, 36.3, 35.9, 32.1, 32.0, 28.4, 28.1, 28.0, 24.4, 24.0, 23.0, 22.7, 21.2, 19.5, 18.8, 12.0.

HRMS **(ESI-TOF)** m/z: [M + H]$^+$ calcd for $C_{50}H_{63}N_2O_4S$ 787.4503, found 787.4551.

ethyl (E)-1-(pyridin-2-yl)-2-styryl-1H-indole-5-carboxylate

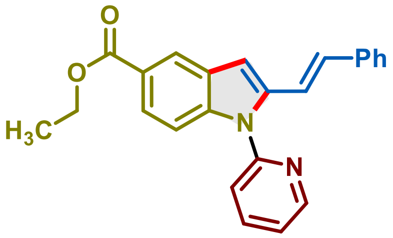

44, 63%
Benzocaine

Purified by (petroleum ether/EtOAc: 85/15), 46.4 mg, 63%, light yellow oil.

^{1}H NMR (400 MHz, CDCl$_3$) δ 8.75 (dd, *J* = 5.8, 2.0 Hz, 1H), 8.39 (s, 1H), 7.94 (td, *J* = 7.7, 1.9 Hz, 1H), 7.89 (dd, *J* = 8.8, 1.6 Hz, 1H), 7.48 (d, *J* = 8.7 Hz, 1H), 7.45 – 7.36 (m, 4H), 7.36 – 7.30 (m, 2H), 7.27 (d, *J* = 1.4 Hz, 1H), 7.16 (d, *J* = 16.4 Hz, 1H), 7.03 (d, *J* = 15.7 Hz, 2H), 4.41 (q, *J* = 7.2 Hz, 2H), 1.43 (t, *J* = 7.1 Hz, 3H).

^{13}C NMR (101 MHz, CDCl$_3$) δ 167.6, 150.9, 150.0, 140.3, 139.7, 138.7, 137.0, 131.7, 128.8, 128.3, 128.2, 126.7, 124.3, 123.8, 123.3, 122.8, 121.8, 117.9, 110.6, 103.0, 60.8, 14.6.

HRMS **(ESI-TOF)** m/z: [M + H]$^+$ calcd for $C_{24}H_{21}N_2O_2$ 369.1598, found 369.1582

(E)-3-ethyl-3-(1-(pyridin-2-yl)-2-styryl-1H-indol-5-yl)piperidine-2,6-dione

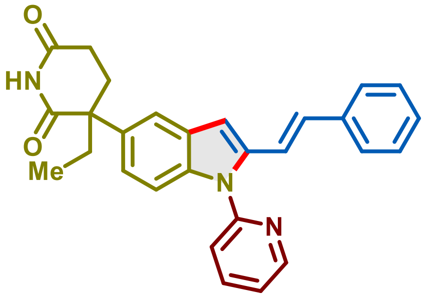

45, 77%

Aminogluthemide

Purified by (petroleum ether/EtOAc: 85/15), 67 mg, 77%, light yellow oil.

^{1}H NMR (500 MHz, CDCl$_3$) δ 8.73 (d, *J* = 5.4 Hz, 1H), 7.97 (s, 1H), 7.90 (td, 1H), 7.54 – 7.48 (m, 2H), 7.44 – 7.35 (m, 4H), 7.32 (t, *J* = 7.6 Hz, 2H), 7.25 – 7.22 (m, 1H), 7.18 – 7.09 (m, 2H), 7.05 (d, *J* = 16.2 Hz, 1H), 6.93 (s, 1H), 2.64 – 2.56 (m, 1H), 2.54 – 2.40 (m, 2H), 2.26 (td, *J* = 14.2, 4.7 Hz, 1H), 2.15 – 2.06 (m, 1H), 2.04 – 1.92 (m, 1H), 0.90 (t, *J* = 7.4 Hz, 3H).

^{13}C NMR (126 MHz, CDCl$_3$) δ 175.9, 172.6, 151.2, 149.8, 139.1, 138.5, 137.1, 137.0, 131.7, 131.3, 129.1, 128.8, 128.1, 126.7, 122.4, 121.5, 120.9, 118.4, 118.1, 111.7, 102.3, 51.3, 33.3, 29.6, 27.7, 9.3.

HRMS **(ESI-TOF)** m/z: [M + H]$^+$ calcd for $C_{28}H_{26}N_3O_2$ 436.2020, found 436.2030.

(R)-2,5,7,8-tetramethyl-2-((4R,8R)-4,8,12-trimethyltridecyl)chroman-6-yl 4-((E)-2-(5-(3-ethyl-2,6-dioxopiperidin-3-yl)-1-(pyridin-2-yl)-1H-indol-2-yl)vinyl)benzoate

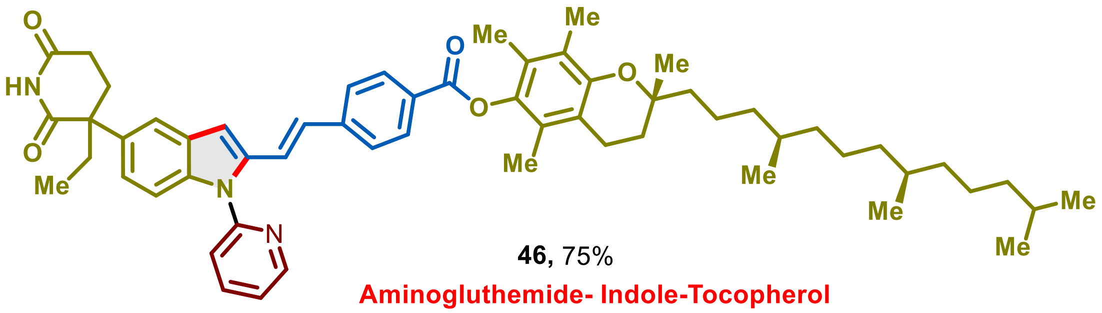

46, 75%

Aminogluthemide- Indole-Tocopherol

Purified by (petroleum ether/EtOAc: 85/15), 133.8 mg, 75%, light yellow oil.

^{1}H NMR (400 MHz, CDCl$_3$) δ 8.75 (dd, *J* = 4.9, 2.8 Hz, 1H), 8.19 (d, *J* = 8.4 Hz, 2H), 8.01 (s, 1H), 7.94 (td, *J* = 7.7, 1.9 Hz, 1H), 7.56 – 7.50 (m, 4H), 7.44 – 7.38 (m, 2H), 7.23 (d, *J* = 16.2 Hz, 1H), 7.17 (d, *J* = 16.2 Hz, 1H), 7.16 – 7.10 (m, 1H), 7.02 (s, 1H), 2.65 – 2.58 (m, 3H), 2.53 – 2.43 (m, 2H), 2.32 – 2.25 (m, 1H), 2.12 (s, 3H), 2.06 (s, 3H), 2.01 (s, 3H), 1.93 – 1.71

(m, 3H), 1.60 – 1.50 (m, 3H), 1.45 – 1.36 (m, 4H), 1.31 – 1.24 (m, 10H), 1.18 – 1.07 (m, 6H), 0.92 – 0.80 (m, 17H).

^{13}C NMR (101 MHz, CDCl$_3$) δ 175.8, 172.6, 165.0, 151.0, 150.0, 149.6, 142.0, 140.7, 138.7, 138.4, 137.2, 131.9, 130.7, 129.8, 129.0, 128.7, 127.0, 126.6, 125.2, 123.3, 122.6, 121.4, 121.4, 120.9, 118.7, 117.6, 111.7, 103.3, 75.2, 51.3, 39.5, 37.7, 37.6, 37.5, 37.5, 37.4, 33.4, 32.9, 32.9, 32.9, 29.6, 28.1, 27.7, 24.9, 24.6, 22.9, 22.8, 21.2, 20.8, 19.9, 19.8, 19.7, 13.2, 12.3, 12.0, 9.3.

HRMS **(ESI-TOF)** m/z: $[M + H]^+$ calcd for $C_{58}H_{74}N_3O_5$ 892.5623, found 892.5674.

1-(4-((4-((E)-2-cyanovinyl)-2,6-dimethylphenyl)amino)pyrimidin-2-yl)-2-((E)-styryl)-1H-indole-5-carbonitrile

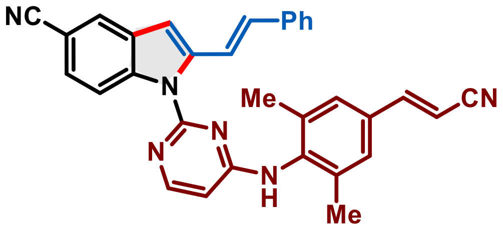

50, 63%

Purified by (petroleum ether/EtOAc: 60/40), 31 mg, 63%, sticky solid. (0.1 mmol scale)

^{1}H NMR (400 MHz, CDCl$_3$) δ 8.35 (d, J = 5.9 Hz, 1H), 8.13 (d, J = 8.8 Hz, 1H), 7.83 (s, 1H), 7.55 (d, J = 17.4 Hz, 1H), 7.51 – 7.41 (m, 2H), 7.39 – 7.28 (m, 5H), 7.25 – 7.01 (m, 4H), 6.89 (s, 1H), 5.85 (d, J = 17.4 Hz, 2H), 2.20 (s, 6H).

^{13}C NMR (101 MHz, CDCl$_3$) δ 162.8, 158.0, 157.0, 149.7, 140.9, 138.8, 137.6, 137.4, 137.1, 133.1, 131.2, 129.0, 128.9, 128.8, 128.3, 127.7, 126.8, 125.9, 125.3, 120.4, 119.4, 118.1, 114.5, 104.9, 103.7, 97.2, 18.5.

HRMS **(ESI-TOF)** m/z: $[M + H]^+$ calcd for $C_{32}H_{25}N_6$ 493.2135, found 493.2160.

1-(4-((4-((E)-2-cyanovinyl)-2,6-dimethylphenyl)amino)pyrimidin-2-yl)-2-((E)-hept-1-en-1-yl)-1H-indole-5-carbonitrile

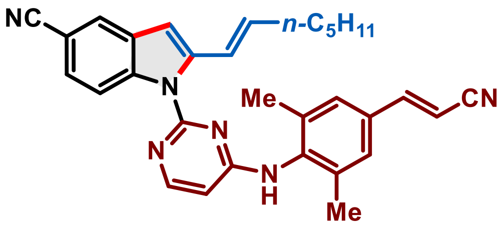

51, 60%

Purified by (petroleum ether/EtOAc: 60/40), 29.2 mg, 60%, light yellow oil. (0.1 mmol scale)

^{1}H NMR (500 MHz, CDCl$_3$) δ 8.32 (d, *J* = 6.0 Hz, 1H), 8.10 (d, *J* = 7.8 Hz, 1H), 7.82 (s, 1H), 7.36 (d, *J* = 16.6 Hz, 2H), 7.24 (s, 2H), 6.82 (d, *J* = 15.9 Hz, 2H), 6.73 (s, 1H), 6.39 – 6.27 (m, 1H), 5.90 (d, *J* = 16.6 Hz, 2H), 2.26 (s, 6H), 2.24 – 2.18 (m, 2H), 1.51 – 1.46 (m, 2H), 1.36 – 1.32 (m, 4H), 0.90 (t, 2H).

^{13}C NMR (126 MHz, CDCl$_3$) δ 162.8, 158.0, 157.2, 149.7, 141.4, 138.6, 137.6, 135.0, 133.2, 129.1, 129.0, 127.8, 125.8, 125.5, 125.0, 120.8, 120.6, 118.1, 114.5, 104.6, 102.9, 97.3, 33.3, 31.6, 28.8, 22.7, 18.5, 14.2.

HRMS **(ESI-TOF)** m/z: [M + H]$^+$ calcd for $C_{31}H_{31}N_6$ 487.2605, found 487.2647 .

(E)-1-(4-(mesitylamino)pyrimidin-2-yl)-2-styryl-1H-indole-5-carbonitrile

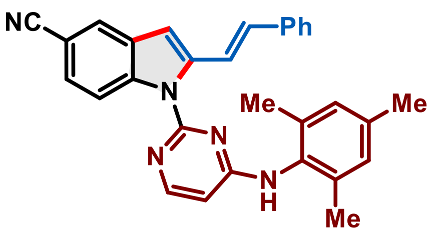

54, 57%

Purified by (petroleum ether/EtOAc: 60/40), 26 mg, 57%, white solid. (0.1 mmol scale)

^{1}H NMR (500 MHz, CDCl$_3$) δ 8.28 (d, *J* = 5.8 Hz, 1H), 8.15 (d, *J* = 6.7 Hz, 1H), 7.86 (s, 1H), 7.61 (d, *J* = 16.2 Hz, 1H), 7.50 (d, *J* = 6.6 Hz, 2H), 7.40 – 7.34 (m, 3H), 7.29 (t, *J* = 7.4 Hz, 1H), 7.16 (d, *J* = 17.1 Hz, 1H), 7.05 (s, 1H), 6.92 (s, 3H), 5.87 (s, 1H), 2.32 (s, 3H), 2.15 (s, 6H).

^{13}C NMR (126 MHz, CDCl$_3$) δ 163.56, 157.80, 156.91, 140.76, 138.83, 138.19, 137.21, 136.31, 131.14, 130.84, 129.58, 128.96, 128.90, 128.22, 126.76, 125.91, 125.34, 120.62, 119.45, 114.29, 104.79, 103.38, 100.11, 21.15, 18.17.

HRMS **(ESI-TOF)** m/z: [M + H]$^+$ calcd for $C_{30}H_{26}N_5$ 456.2183, found 456.2198.

1,4-bis(2-(1-(pyridin-2-yl)-2-((E)-styryl)-1H-indol-5-yl)propan-2-yl)benzene

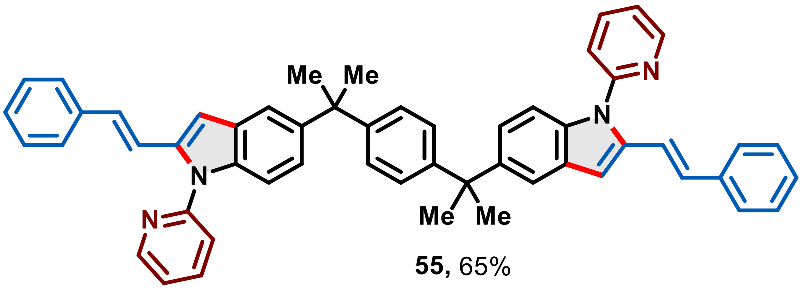

55, 65%

Purified by (petroleum ether/EtOAc:90/10), 48.8 mg, 65%, light yellow oil. (0.1 mmole scale)

^{1}H NMR (500 MHz, CDCl$_3$) δ 8.69 (dd, *J* = 5.3, 2.4 Hz, 2H), 7.86 (td, *J* = 7.6, 2.0 Hz, 2H), 7.59 (d, *J* = 2.0 Hz, 2H), 7.43 – 7.36 (m, 8H), 7.31 (t, *J* = 7.6 Hz, 6H), 7.24 – 7.20 (m, 2H), 7.17 – 7.05 (m, 8H), 6.99 (dd, *J* = 8.7, 2.0 Hz, 2H), 6.94 (s, 2H), 1.73 (s, 12H).

^{13}C NMR (126 MHz, CDCl$_3$) δ 151.6, 149.7, 148.3, 144.2, 138.3, 138.2, 137.4, 136.1, 130.3, 128.8, 128.4, 127.8, 126.6, 126.4, 123.6, 121.9, 121.4, 118.7, 117.7, 110.6, 102.8, 42.6, 31.3.

HRMS **(ESI-TOF)** m/z: [M + H]$^+$ calcd for $C_{54}H_{47}N_4$ 751.3795, found 751.3794.

5,5'-((propane-2,2-diylbis(4,1-phenylene))bis(oxy))bis(1-(pyridin-2-yl)-2-((E)-styryl)-1H-indole)

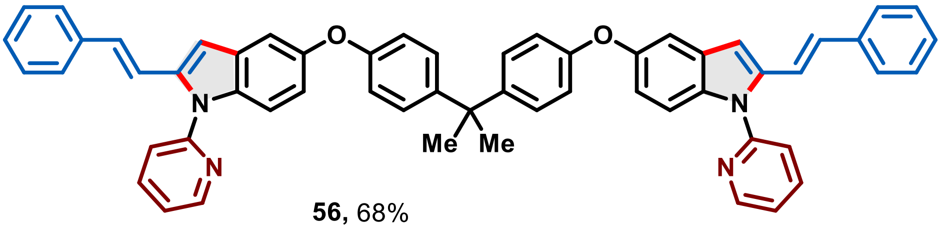

56, 68%

Purified by (petroleum ether/EtOAc:90/10), 55.5 mg, 68%, light yellow oil. (0.1 mmole scale)

^{1}H NMR (400 MHz, CDCl$_3$) δ 8.73 (dd, *J* = 4.9, 2.0 Hz, 2H), 7.90 (td, *J* = 7.8, 2.0 Hz, 2H), 7.49 (d, *J* = 8.8 Hz, 2H), 7.43 – 7.39 (m, 6H), 7.37 – 7.30 (m, 6H), 7.27 (d, *J* = 2.2 Hz, 2H), 7.25 – 7.22 (m, 2H), 7.20 – 7.16 (m, 4H), 7.10 (d, *J* = 16.4 Hz, 2H), 7.05 (d, *J* = 16.4 Hz, 2H), 6.96 – 6.88 (m, 8H), 1.67 (s, 6H).

^{13}C NMR (101 MHz, CDCl$_3$) δ 157.0, 151.6, 151.4, 149.8, 144.7, 139.1, 138.5, 137.2, 134.8, 131.1, 129.5, 128.8, 128.0, 128.0, 126.7, 122.2, 121.5, 118.3, 117.0, 116.5, 112.1, 110.8, 102.3, 42.1, 31.2.

HRMS **(ESI-TOF)** m/z: [M + H]$^+$ calcd for $C_{57}H_{45}N_4O_2$ 817.3537, found 817.3513

5,5'-(9H-fluorene-9,9-diyl)bis(1-(pyridin-2-yl)-2-((E)-styryl)-1H-indole)

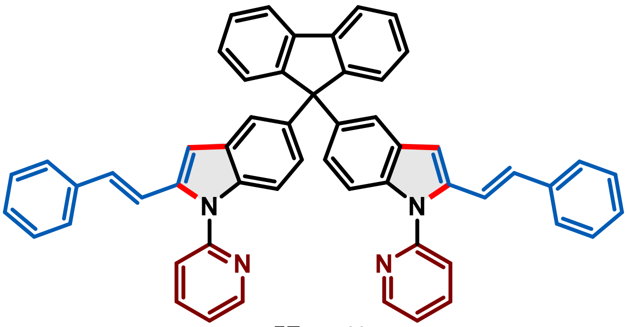

57, 69%

Purified by (petroleum ether/EtOAc:90/10), 52 mg, 69%, sticky solid. (0.1 mmole scale)

^{1}H NMR (400 MHz, CDCl$_3$) δ 8.69 (dd, *J* = 5.4, 2.0 Hz, 2H), 7.85 (td, *J* = 7.6, 1.9 Hz, 2H), 7.79 (d, *J* = 7.5 Hz, 2H), 7.52 (d, *J* = 7.7 Hz, 2H), 7.48 – 7.35 (m, 10H), 7.34 – 7.26 (m, 8H), 7.24 – 7.17 (m, 6H), 7.09 – 6.98 (m, 4H), 6.81 (s, 2H).

^{13}C NMR (101 MHz, CDCl$_3$) δ 152.6, 151.4, 149.8, 140.2, 139.7, 138.4, 138.3, 137.3, 136.8, 130.5, 128.8, 128.5, 127.8, 127.7, 127.2, 126.6, 124.5, 122.1, 121.4, 120.2, 119.7, 118.5, 110.8, 102.7, 65.7.

HRMS **(ESI-TOF)** m/z: $[M + H]^+$ calcd for $C_{55}H_{39}N_4$ 755.3169 found 755.3166

1,4-bis(1-(pyridin-2-yl)-2-((E)-styryl)-1H-indol-5-yl)benzene

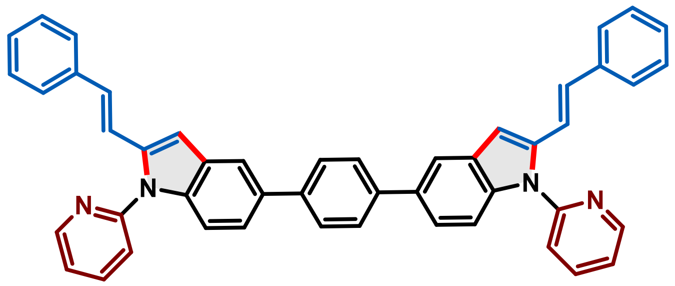

58, 63%

Purified by (petroleum ether/EtOAc:80/20), 42 mg, 63%, sticky solid. (0.1 mmole scale)

^{1}H NMR (500 MHz, CDCl$_3$) δ 8.76 (dd, *J* = 5.0, 1.8 Hz, 2H), 7.92 (m, *J* = 5.2 Hz, 4H), 7.76 (m, 4H), 7.60 (m, *J* = 8.5 Hz, 2H), 7.51 (d, *J* = 8.7 Hz, 2H), 7.45 (dd, *J* = 15.7, 7.8 Hz, 6H), 7.40 – 7.36 (m, 2H), 7.34 (t, *J* = 7.6 Hz, 4H), 7.25 (d, *J* = 7.3 Hz, 2H), 7.18 (d, *J* = 16.2 Hz, 2H), 7.11 (d, *J* = 16.2 Hz, 2H), 7.06 (s, 2H).

^{13}C NMR (126 MHz, CDCl$_3$) δ 151.4, 149.9, 140.5, 138.9, 138.5, 137.5, 137.2, 134.6, 130.9, 129.4, 128.8, 127.9, 127.7, 126.7, 122.8, 122.2, 121.6, 118.9, 118.4, 111.4, 102.7.

HRMS **(ESI-TOF)** m/z: $[M + H]^+$ calcd for $C_{48}H_{35}N_4$ 667.2856, found 667.2848.

1,3-bis((1-(pyridin-2-yl)-2-((E)-styryl)-1H-indol-5-yl)oxy)benzene

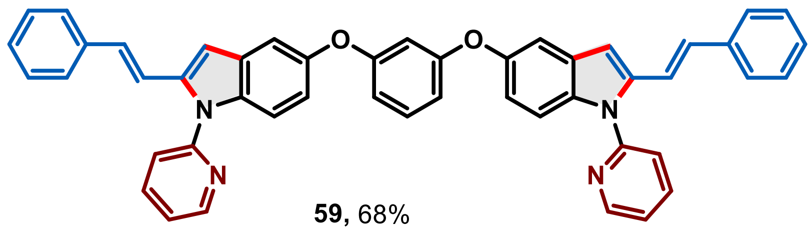

59, 68%

Purified by (petroleum ether/EtOAc:85/15), 47.5 mg, 68%, light yellow oil. (0.1 mmole scale)

^{1}H NMR (400 MHz, CDCl$_3$) δ 8.73 (dd, *J* = 4.9, 2.0 Hz, 2H), 7.89 (td, *J* = 7.8, 2.0 Hz, 2H), 7.49 (d, *J* = 8.8 Hz, 2H), 7.42 – 7.38 (m, 6H), 7.37 – 7.29 (m, 8H), 7.24 (q, *J* = 3.0 Hz, 2H),

7.19 (d, *J* = 8.2 Hz, 1H), 7.13 (d, *J* = 16.2 Hz, 2H), 7.05 (d, *J* = 16.2 Hz, 2H), 6.95 (dd, *J* = 8.9, 2.3 Hz, 2H), 6.93 (s, 2H), 6.72 (t, *J* = 2.4 Hz, 1H), 6.63 (dd, *J* = 8.2, 2.4 Hz, 2H).

^{13}C NMR (101 MHz, CDCl$_3$) δ 160.5, 151.3, 151.1, 149.8, 139.2, 138.5, 137.1, 135.0, 131.1, 130.1, 129.5, 128.8, 128.0, 126.7, 122.3, 121.6, 118.3, 116.6, 112.1, 111.1, 111.1, 107.4, 102.3.

HRMS **(ESI-TOF)** m/z: [M + H]$^+$ calcd for $C_{48}H_{35}N_4O_2$ 699.2755, found 699.2745. (0.1 mmole scale)

5,5'-((propane-2,2-diylbis(4,1-phenylene))bis(oxy))bis(2-((E)-hept-1-en-1-yl)-1-(pyridin-2-yl)-1H-indole)

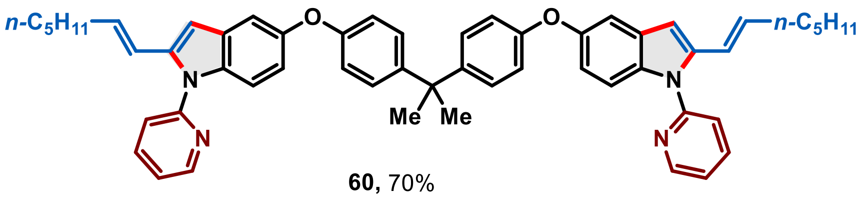

60, 70%

Purified by (petroleum ether/EtOAc:85/15), 56.3 mg, 70%, light yellow oil. (0.1 mmole scale)

^{1}H NMR (500 MHz, CDCl$_3$) δ 8.69 (dd, *J* = 5.3, 2.4 Hz, 1H), 7.87 (td, *J* = 7.6, 2.0 Hz, 1H), 7.45 (d, *J* = 8.9 Hz, 1H), 7.38 – 7.31 (m, 2H), 7.22 (d, *J* = 2.3 Hz, 1H), 7.14 (d, *J* = 8.9 Hz, 2H), 6.91 – 6.85 (m, 3H), 6.69 (s, 1H), 6.31 (d, *J* = 16.0 Hz, 1H), 6.28 – 6.21 (m, 1H), 2.17 (q, *J* = 7.2 Hz, 2H), 1.65 (s, 3H), 1.46 – 1.40 (m, 2H), 1.32 – 1.28 (m, 4H), 0.89 (t, *J* = 7.0 Hz, 3H).

^{13}C NMR (126 MHz, CDCl$_3$) δ 157.1, 151.5, 151.3, 149.6, 144.5, 139.5, 138.3, 134.7, 134.4, 129.6, 127.9, 122.0, 121.5, 119.8, 116.9, 115.9, 111.9, 110.7, 101.2, 42.0, 33.3, 31.5, 31.2, 28.9, 22.6, 14.2.

HRMS **(ESI-TOF)** m/z: [M + H]$^+$ calcd for $C_{55}H_{57}N_4O_2$ 805.4476, found 805.4469.

(E)-1-(pyridin-2-yl)-2-styrylindoline

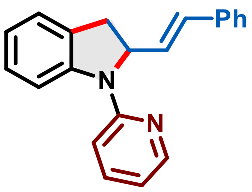

62, 13%

Purified by petroleum ether, 8 mg, 33%, colourless oil. (0.2 mmole scale)

^{1}H NMR (500 MHz, CDCl$_3$) δ 8.34 (d, *J* = 5.2 Hz, 1H), 8.21 (d, *J* = 8.4 Hz, 1H), 7.52 (t, *J* = 7.9 Hz, 1H), 7.34 – 7.30 (m, 2H), 7.23 – 7.14 (m, 5H), 6.95 – 6.82 (m, 2H), 6.80 – 6.72 (m,

1H), 6.56 (d, J = 15.9 Hz, 1H), 6.28 (dd, J = 15.9, 7.0 Hz, 1H), 5.13 – 5.04 (m, 1H), 3.62 (dd, J = 15.6, 10.0 Hz, 1H), 2.99 (dd, J = 15.6, 3.3 Hz, 1H).

^{13}C NMR (126 MHz, CDCl$_3$) δ 155.5, 148.0, 144.7, 137.3, 136.6, 129.8, 128.7, 128.5, 127.79, 127.5, 126.6, 126.2, 124.9, 121.0, 114.9, 114.5, 109.9, 62.8, 36.4.

HRMS **(ESI-TOF)** m/z: [M + H]$^+$ calcd for $C_{21}H_{19}N_2$ 299.1543, found 299.1539.

***2-phenethyl-1-(pyridin-2-yl)-1H-indole*[12]**

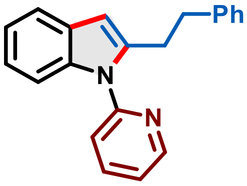

63, 83%

Purified by (petroleum ether/EtOAc:95/5), 25 mg, 83%, colourless oil. (0.1 mmole scale)

^{1}H NMR (400 MHz, CDCl$_3$) δ 8.67 (dd, J = 5.1, 2.2 Hz, 1H), 7.88 (td, J = 7.7, 2.0 Hz, 1H), 7.63 – 7.55 (m, 1H), 7.41 (d, J = 8.0 Hz, 1H), 7.35 – 7.31 (m, 2H), 7.26 – 7.21 (m, 2H), 7.20 – 7.13 (m, 3H), 7.13 – 7.10 (m, 2H), 6.50 (s, 1H), 3.20 – 3.10 (m, 2H), 2.95 – 2.89 (m, 2H).

^{13}C NMR (101 MHz, CDCl$_3$) δ 151.4, 149.6, 141.5, 140.8, 138.4, 137.2, 128.6, 128.5, 128.4, 128.3, 126.0, 122.1, 121.8, 121.1, 120.7, 120.1, 110.1, 102.5, 35.3, 29.6.

HRMS **(ESI-TOF)** m/z: [M + H]$^+$ calcd for $C_{21}H_{19}N_2$ 299.1543, found 299.1549.

methyl (E)-3-(1-(pyridin-2-yl)-2-((E)-styryl)-1H-indol-7-yl)acrylate

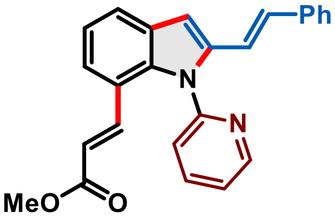

64, 76%

Purified by (petroleum ether/EtOAc:75/25), 29 mg, 76%, colourless oil.

^{1}H NMR (400 MHz, CDCl$_3$) δ 8.78 (dd, J = 4.9, 2.0 Hz, 1H), 7.93 (td, J = 7.8, 2.0 Hz, 1H), 7.69 (d, J = 7.9 Hz, 1H), 7.54 (dd, J = 7.6, 4.9 Hz, 1H), 7.36 – 7.27 (m, 6H), 7.25 – 7.21 (m, 1H), 7.16 (t, J = 7.7 Hz, 1H), 7.14 – 7.05 (m, 2H), 6.99 (s, 1H), 6.75 (d, J = 15.7 Hz, 1H), 6.17 (d, J = 15.7 Hz, 1H), 3.66 (s, 3H).

^{13}C NMR (101 MHz, CDCl$_3$) δ 167.1, 152.4, 150.2, 141.9, 140.2, 138.9, 137.0, 136.4, 131.5, 130.0, 128.8, 128.1, 126.6, 124.3, 124.1, 122.8, 122.2, 121.2, 119.8, 117.9, 117.3, 101.1, 51.4.

HRMS **(ESI-TOF)** m/z: [M + H]$^+$ calcd for $C_{25}H_{21}N_2O_2$ 381.1598, found 381.1593.

***(E)-2-styryl-1H-indole*[12]**

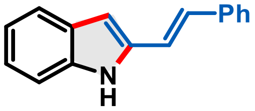

65, 74%

Purified by (petroleum ether/EtOAc:95/5), 32.4 mg, 74%, white solid. (0.1 mmole scale)

^{1}H NMR (500 MHz, CDCl$_3$) δ 8.23 (s, 1H), 7.60 (d, *J* = 7.9 Hz, 1H), 7.51 (d, *J* = 7.6 Hz, 2H), 7.42 – 7.33 (m, 3H), 7.29 (t, *J* = 7.3 Hz, 1H), 7.21 (t, *J* = 7.2 Hz, 1H), 7.16 – 7.08 (m, 2H), 6.91 (d, *J* = 16.5 Hz, 1H), 6.63 (s, 1H).

^{13}C NMR (126 MHz, CDCl$_3$) δ 137.1, 136.9, 136.4, 129.1, 128.9, 127.9, 127.3, 126.4, 123.0, 120.8, 120.3, 119.1, 110.7, 104.0.

HRMS **(ESI-TOF)** m/z: [M + H]$^+$ calcd for $C_{16}H_{14}N$ 220.1121, found 220.1119.

***3-(1H-indol-2-yl)-2,4-diphenyl-2,3,4,9-tetrahydro-1H-carbazole*[13]**

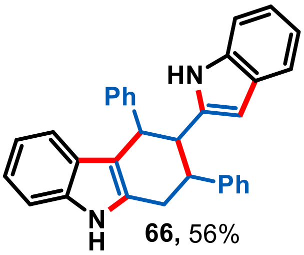

66, 56%

Purified by (petroleum ether/EtOAc:80/20), 25 mg, 56%, sticky solid. (0.1 mmole scale)

^{1}H NMR (400 MHz, CDCl$_3$) δ 8.01 (s, 1H), 7.46 – 7.39 (m, 3H), 7.35 – 7.28 (m, 2H), 7.25 – 7.17 (m, 6H), 7.12 – 6.97 (m, 4H), 6.85 (dd, *J* = 7.5, 2.1 Hz, 2H), 5.88 (d, *J* = 2.0 Hz, 1H), 4.79 (s, 1H), 4.07 – 3.49 (m, 2H), 3.27 – 2.92 (m, 2H).

^{13}C NMR (101 MHz, CDCl$_3$) δ 144.7, 142.8, 139.0, 136.8, 135.4, 128.5, 128.5, 128.4, 128.3, 128.2, 127.4, 126.9, 126.6, 121.9, 121.2, 120.0, 119.8, 119.6, 119.2, 110.8, 110.6, 110.1, 101.8, 50.4, 43.9, 39.5, 25.3.

HRMS **(ESI-TOF)** m/z: [M + H]$^+$ calcd for $C_{32}H_{27}N_2$ 439.2169, found 439.2165.

***11,11,12-triphenyl-5,11-dihydroindeno[1,2-b]carbazole*[14]**

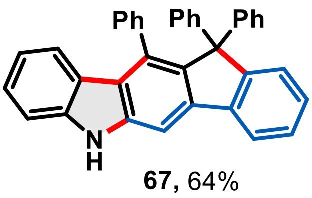

67, 64%

Purified by (petroleum ether/EtOAc:85/15), 31 mg, 64%, yellow solid. (0.1 mmole scale)

^{1}H NMR (500 MHz, CDCl$_3$) δ 8.14 (s, 1H), 7.83 (s, 1H), 7.79 (d, *J* = 7.6 Hz, 1H), 7.35 – 7.27 (m, 3H), 7.25 – 7.20 (m, 2H), 7.19 – 7.15 (m, 1H), 7.11 – 7.06 (m, 4H), 7.03 – 6.97 (m, 8H), 6.79 (t, *J* = 7.6 Hz, 1H), 6.70 (d, *J* = 7.5 Hz, 2H), 6.29 (d, *J* = 8.1 Hz, 1H).

^{13}C NMR (126 MHz, CDCl$_3$) δ 155.7, 144.3, 141.1, 140.3, 139.9, 139.6, 139.6, 138.3, 135.5, 129.8, 129.1, 128.1, 128.1, 127.5, 127.2, 126.9, 126.1, 125.6, 125.4, 123.8, 123.2, 122.2, 119.7, 119.4, 110.3, 101.3, 65.6.

HRMS **(ESI-TOF)** m/z: $[M + H]^+$ calcd for $C_{37}H_{26}N$ 484.2060, found 484.2013.

16. ^{1}H and ^{13}C Spectra of the compounds

1k

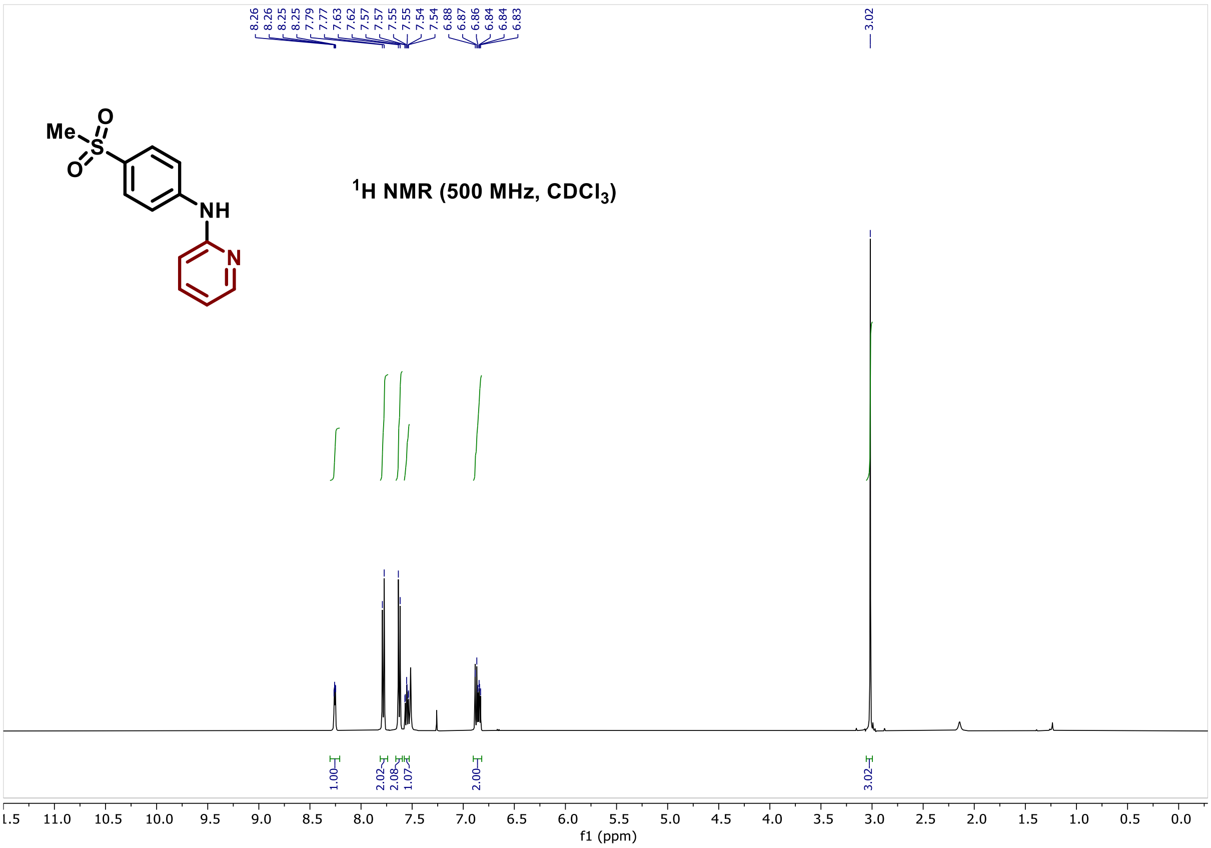

8.26 8.26 8.25 8.25 7.79 7.77 7.63 7.62 7.57 7.57 7.55 7.55 7.54 7.54 6.88 6.87 6.86 6.84 6.84 6.83
3.02
Me
S
O
O
NH
N
¹H NMR (500 MHz, CDCl₃)
1.00
2.02
2.08
1.07
2.00
3.02
f1 (ppm)

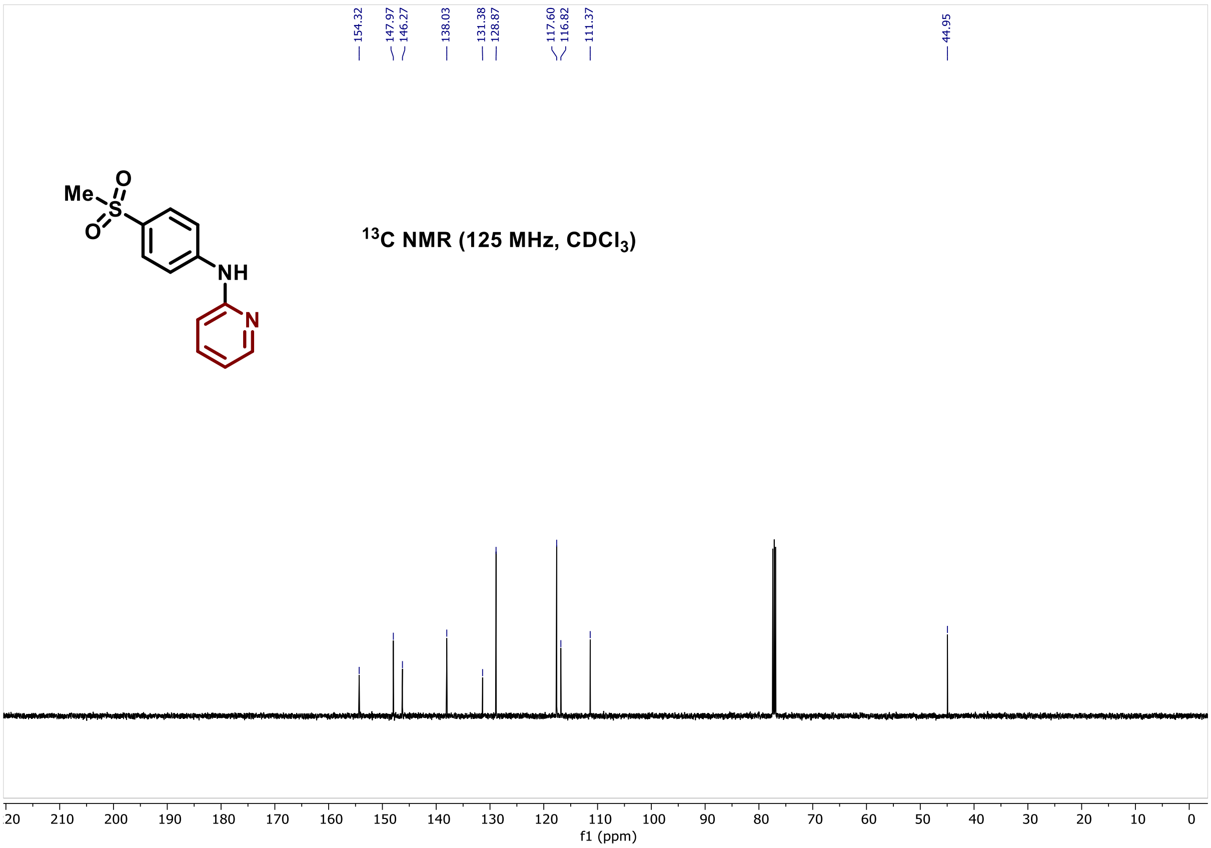

154.32
147.97
146.27
138.03
131.38
128.87
117.60
116.82
111.37
44.95
Me
S
O
O
NH
N
¹³C NMR (125 MHz, CDCl₃)
f1 (ppm)

1l

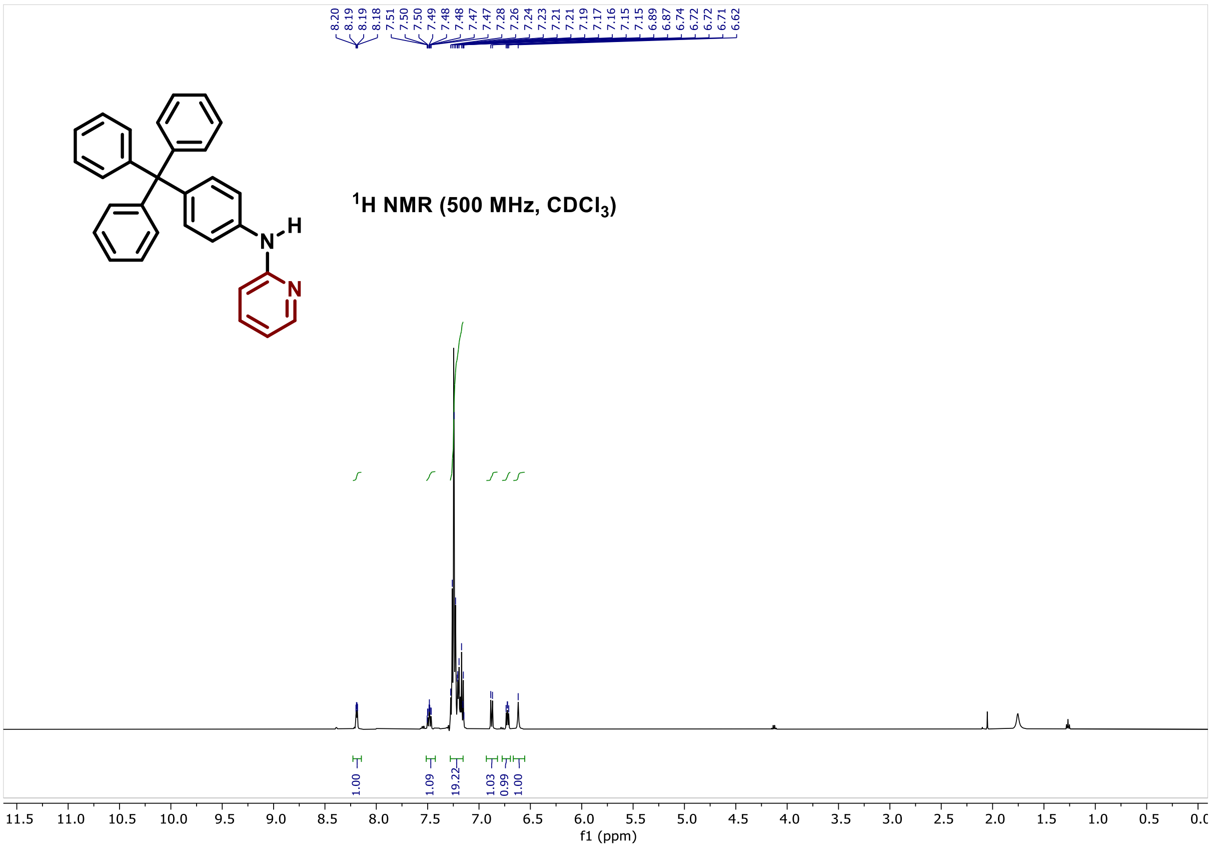

8.20
8.19
8.19
8.18
7.51
7.50
7.50
7.49
7.48
7.48
7.47
7.47
7.28
7.26
7.24
7.23
7.21
7.21
7.19
7.17
7.16
7.15
7.15
6.89
6.87
6.74
6.72
6.72
6.71
6.62
^{1}H NMR (500 MHz, CDCl$_3$)
1.00
1.09
19.22
1.03
0.99
1.00
11.5 11.0 10.5 10.0 9.5 9.0 8.5 8.0 7.5 7.0 6.5 6.0 5.5 5.0 4.5 4.0 3.5 3.0 2.5 2.0 1.5 1.0 0.5 0.0
f1 (ppm)

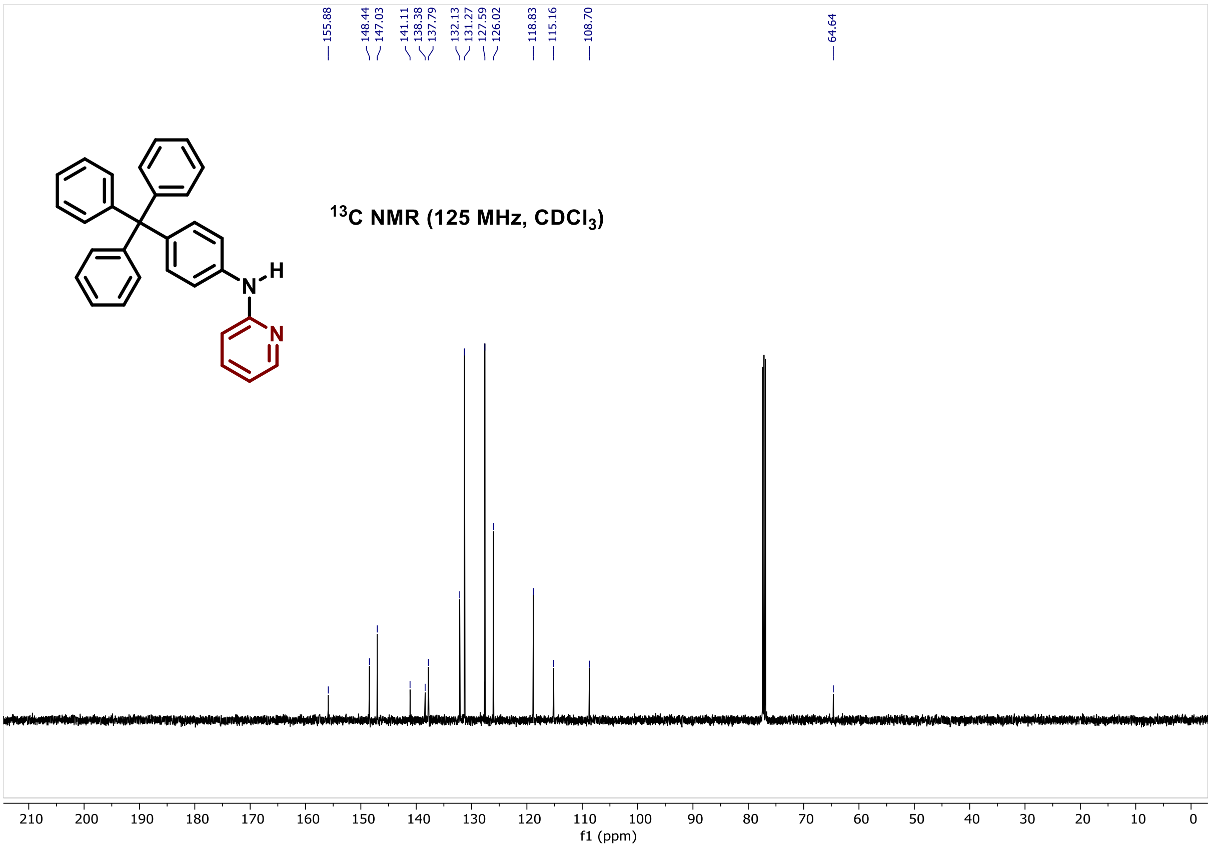

155.88
148.44
147.03
141.11
138.38
137.79
132.13
131.27
127.59
126.02
118.83
115.16
108.70
64.64
^{13}C NMR (125 MHz, CDCl$_3$)
210 200 190 180 170 160 150 140 130 120 110 100 90 80 70 60 50 40 30 20 10 0
f1 (ppm)

1o

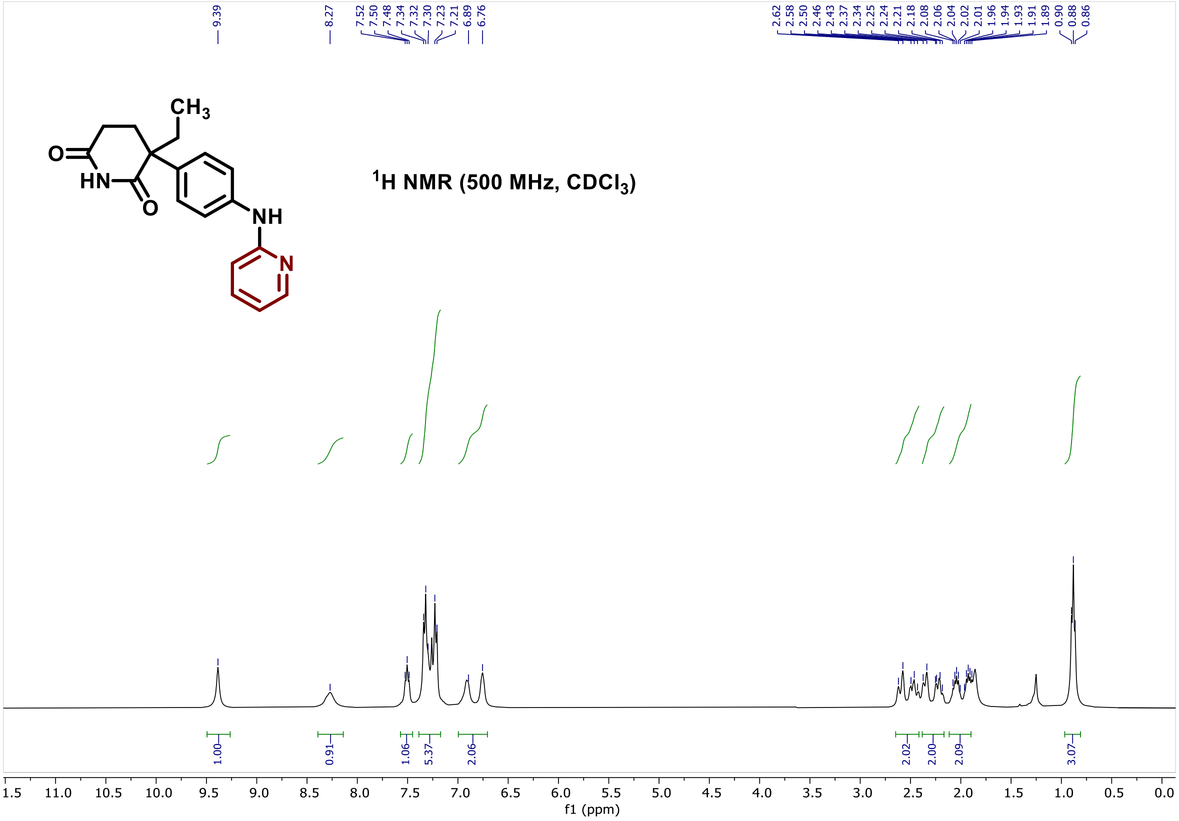

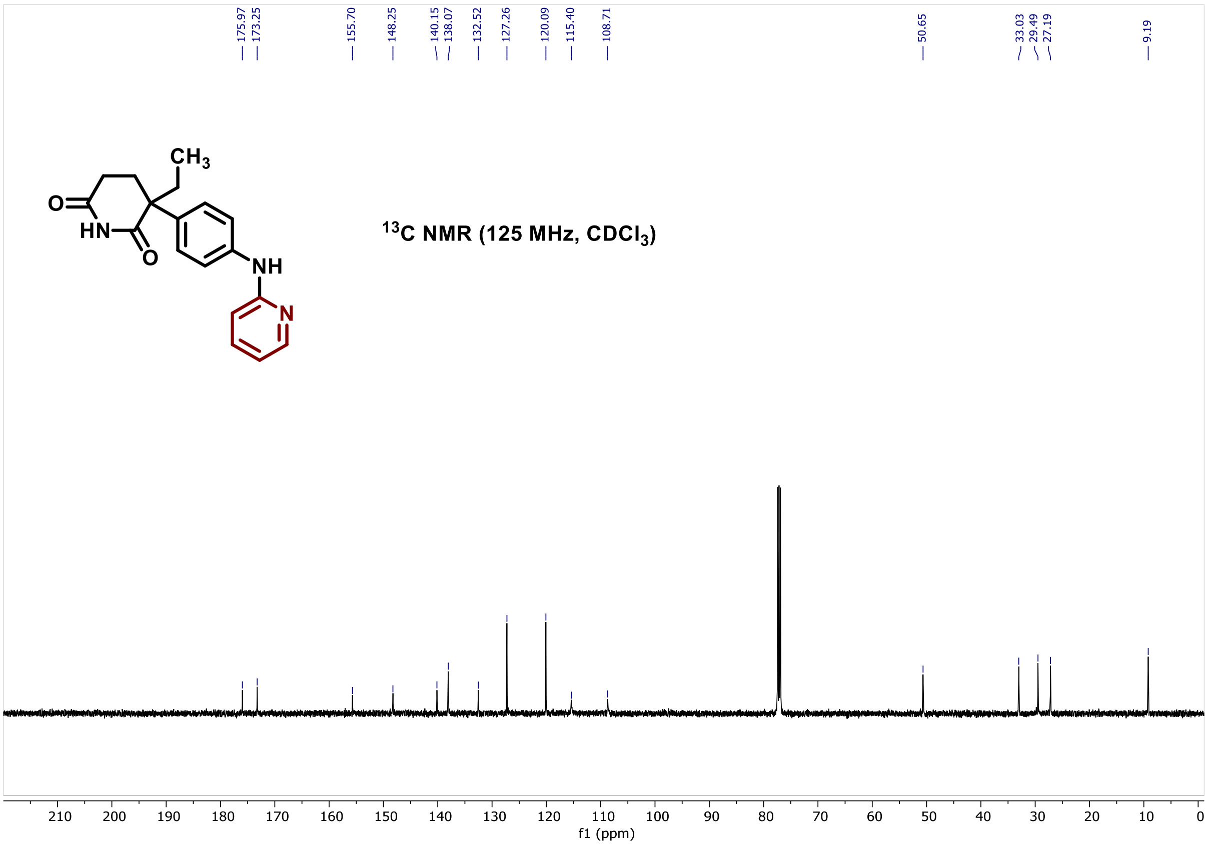

1p

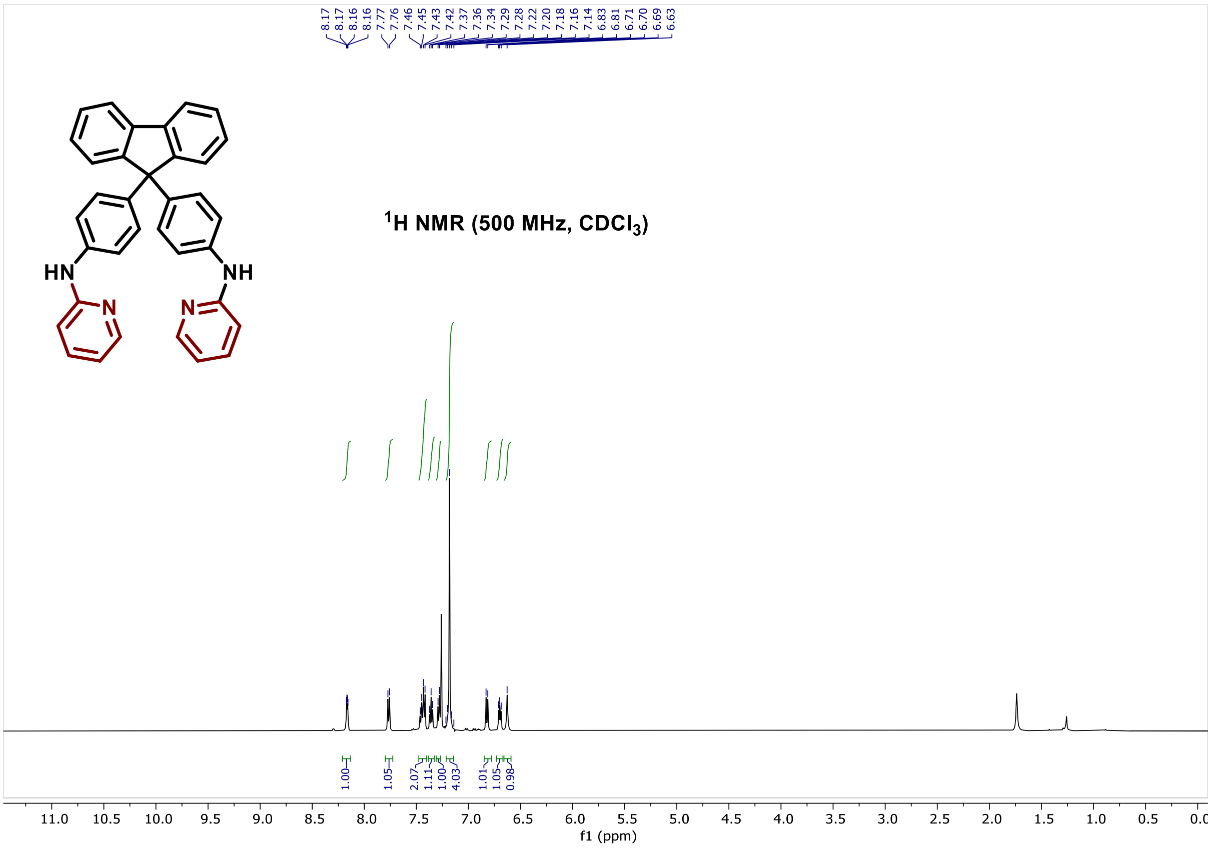

8.17
8.17
8.16
8.16
7.77
7.76
7.46
7.45
7.43
7.42
7.37
7.36
7.34
7.29
7.28
7.22
7.20
7.18
7.16
7.14
6.83
6.81
6.71
6.70
6.69
6.63
HN
NH
N
N
1H NMR (500 MHz, CDCl3)
1.00
1.05
2.07
1.11
1.00
1.00
4.03
1.01
1.05
0.98
11.0
10.5
10.0
9.5
9.0
8.5
8.0
7.5
7.0
6.5
6.0
5.5
5.0
4.5
4.0
3.5
3.0
2.5
2.0
1.5
1.0
0.5
0.0
f1 (ppm)

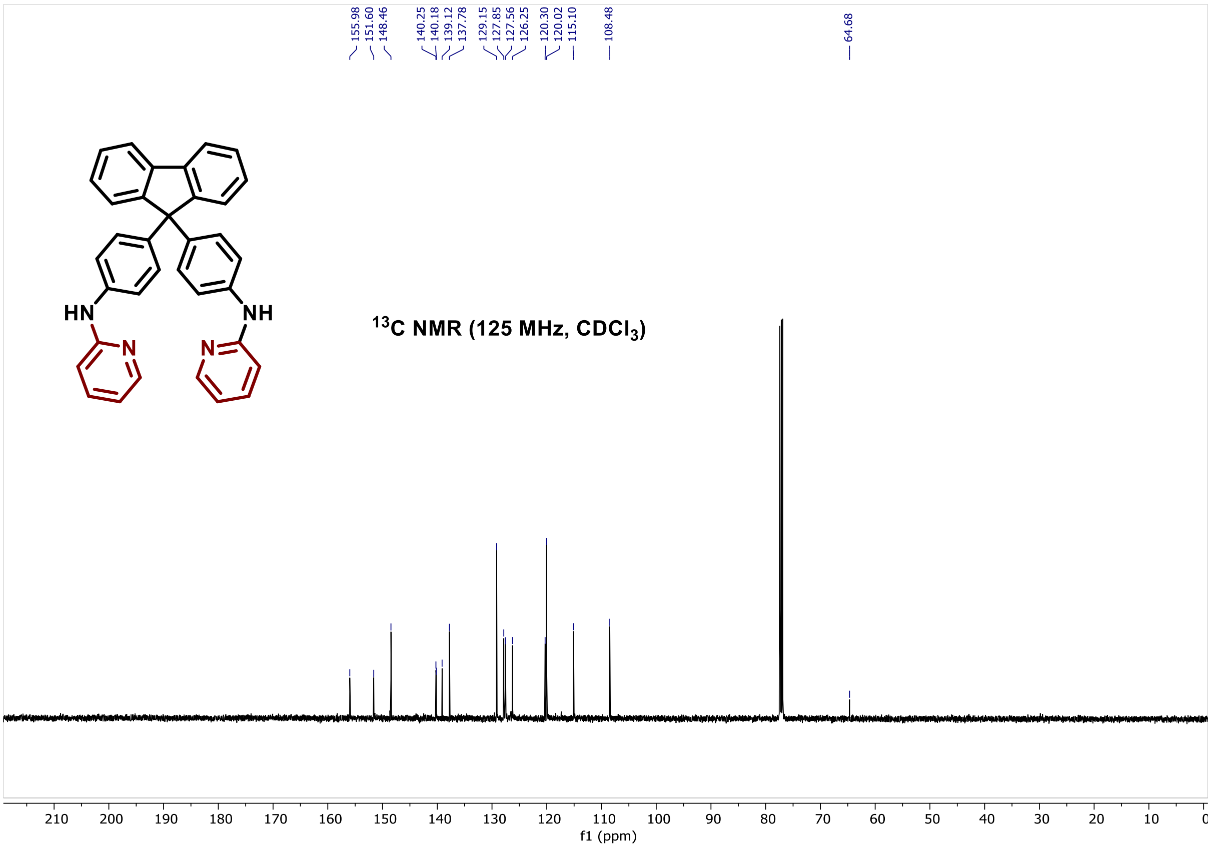

155.98
151.60
148.46
140.25
140.18
139.12
137.78
129.15
127.85
127.56
126.25
120.30
120.02
115.10
108.48
64.68
HN
NH
N
N
13C NMR (125 MHz, CDCl3)
210
200
190
180
170
160
150
140
130
120
110
100
90
80
70
60
50
40
30
20
10
0
f1 (ppm)

1q

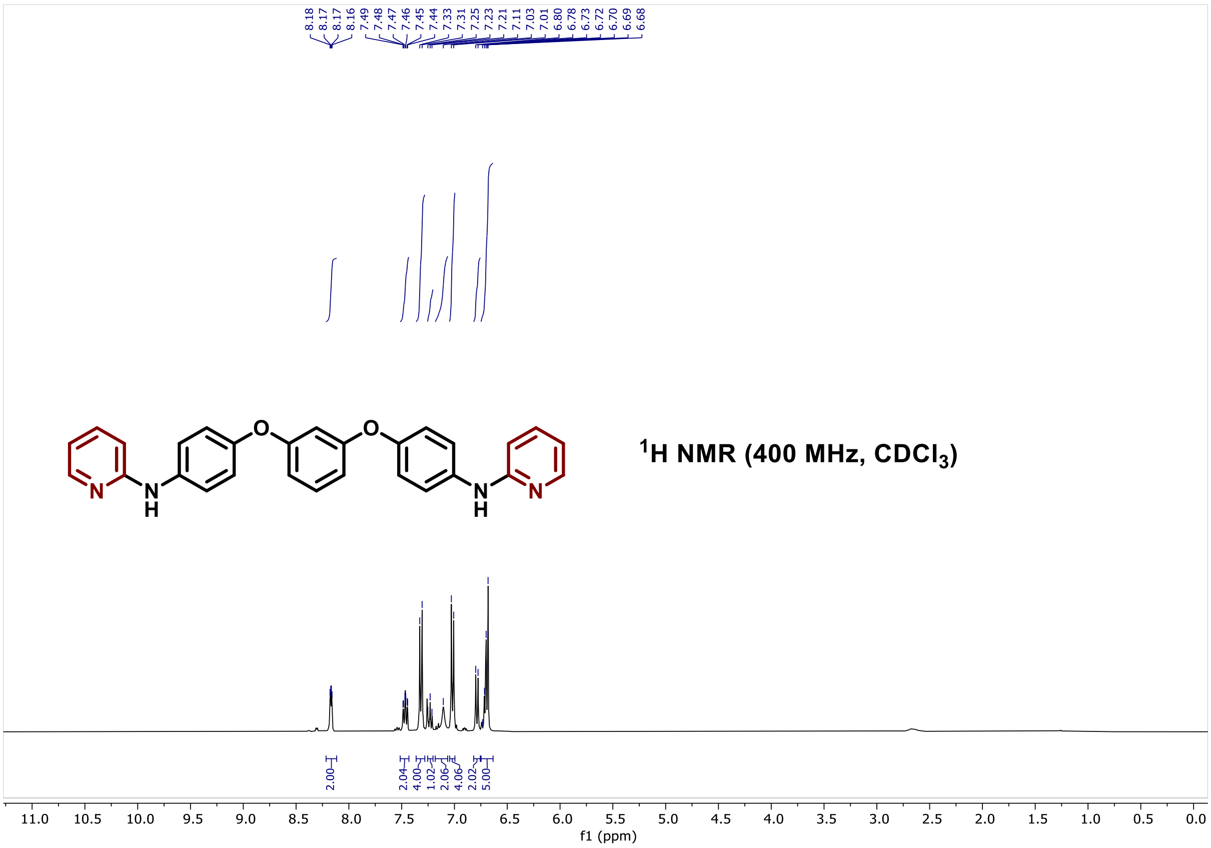

8.18 8.17 8.17 8.16 7.49 7.48 7.47 7.46 7.45 7.44 7.33 7.31 7.25 7.23 7.21 7.11 7.03 7.01 6.80 6.78 6.73 6.72 6.70 6.69 6.68
^{1}H NMR (400 MHz, CDCl$_3$)
2.00 2.04 4.00 1.02 2.06 4.06 2.02 5.00
f1 (ppm)

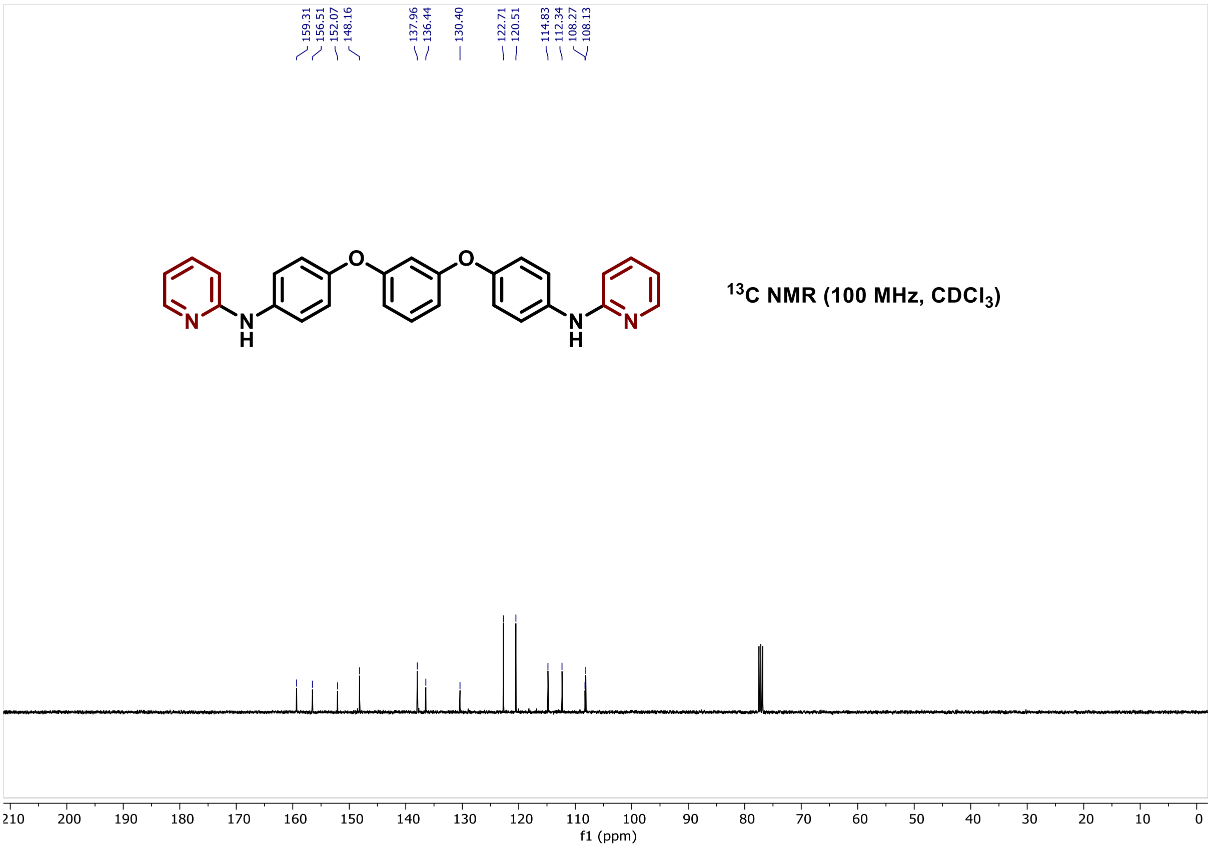

159.31 156.51 152.07 148.16 137.96 136.44 130.40 122.71 120.51 114.83 112.34 108.27 108.13
^{13}C NMR (100 MHz, CDCl$_3$)
f1 (ppm)

1s

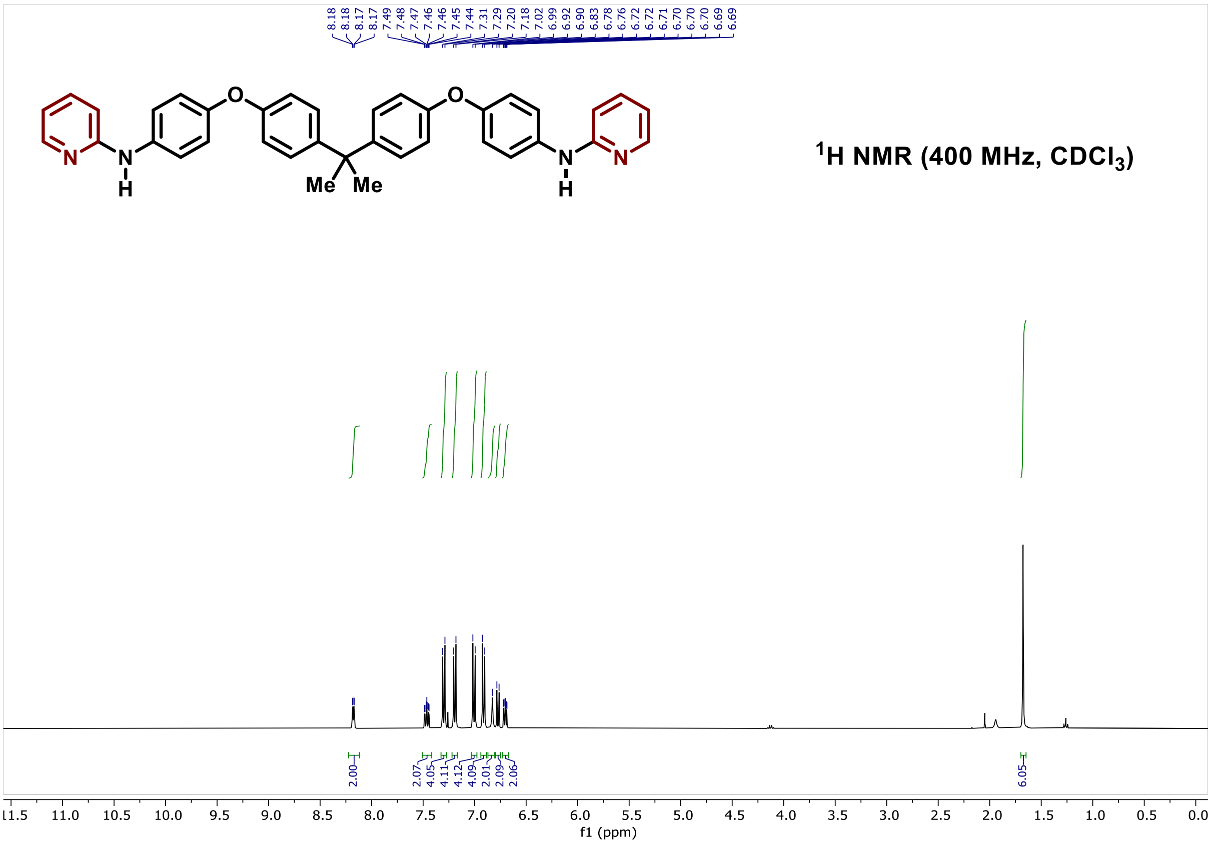

^{1}H NMR (400 MHz, CDCl$_3$)
Me Me
f1 (ppm)

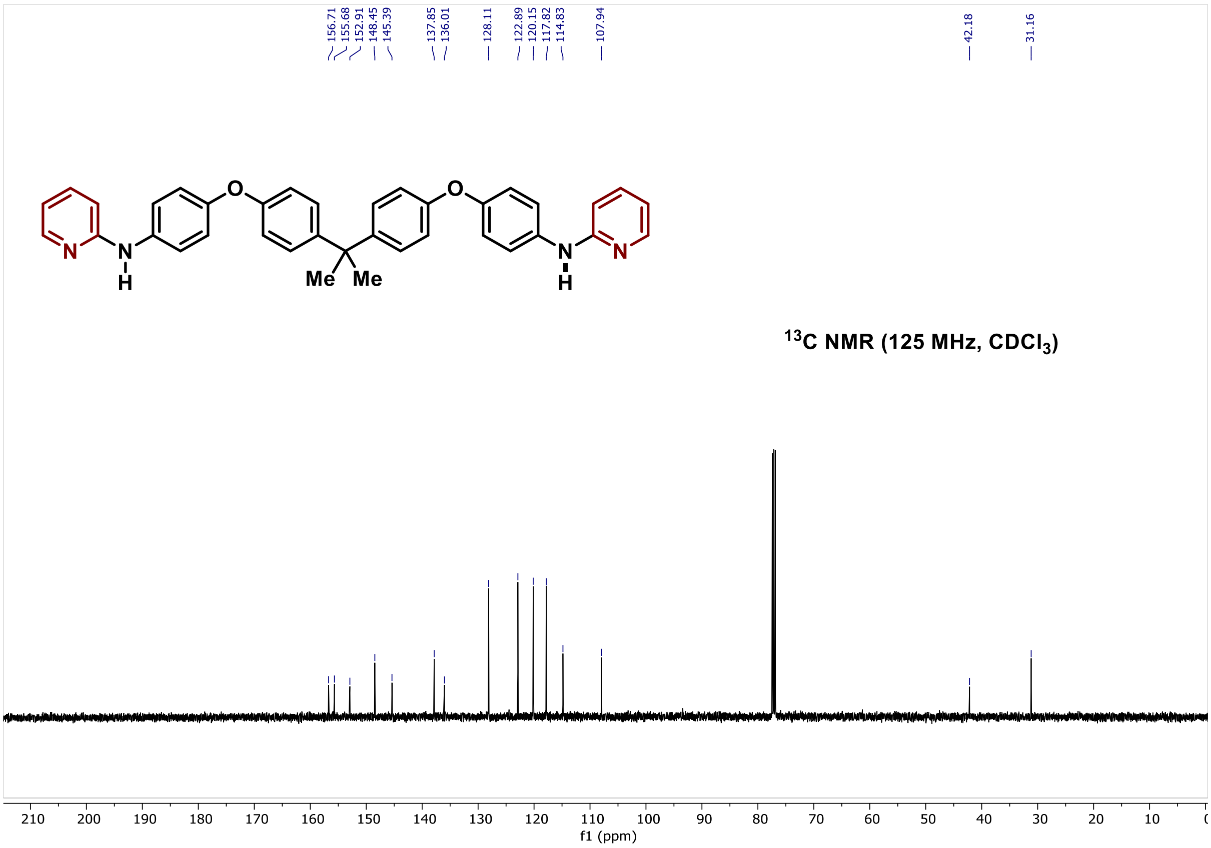

^{13}C NMR (125 MHz, CDCl$_3$)
156.71 155.68 152.91 148.45 145.39 137.85 136.01 128.11 122.89 120.15 117.82 114.83 107.94 42.18 31.16
f1 (ppm)

3

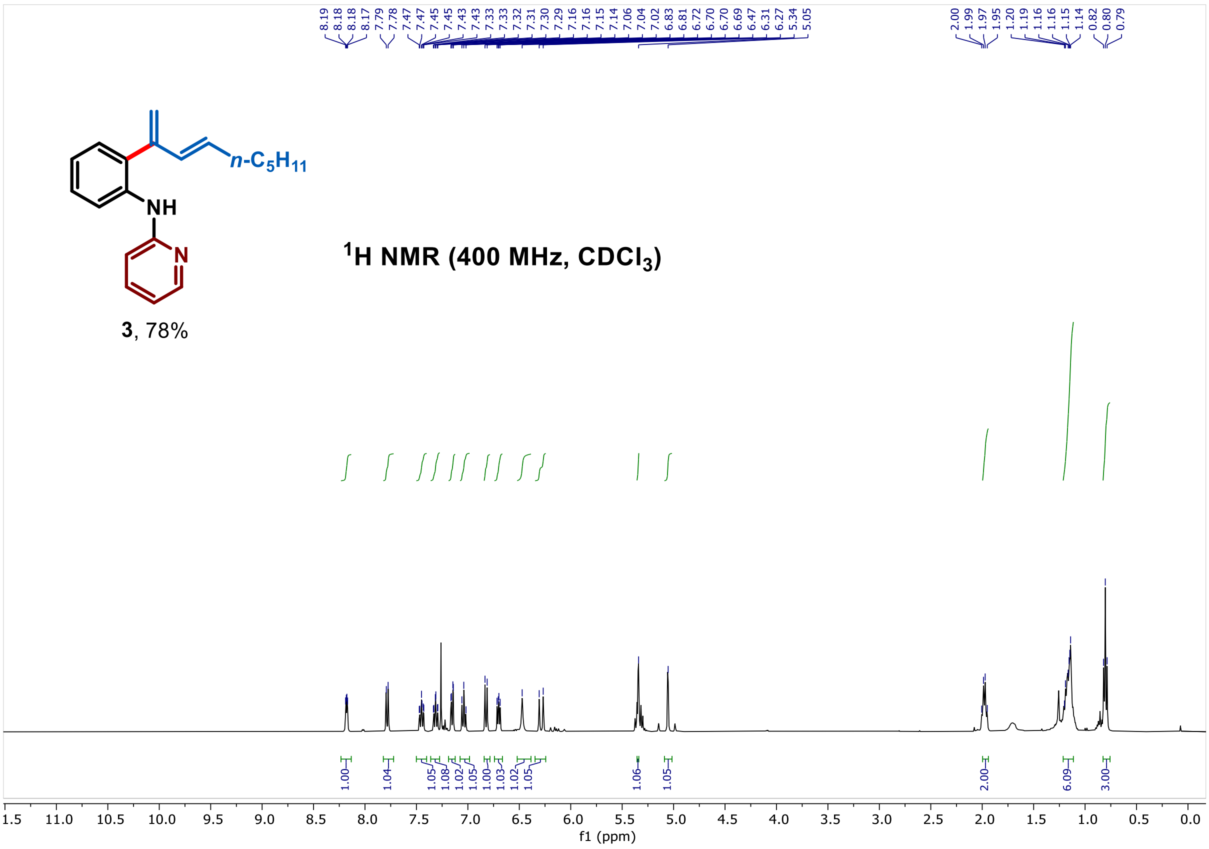

n-C5H11
NH
N
3, 78%
1H NMR (400 MHz, CDCl3)
f1 (ppm)

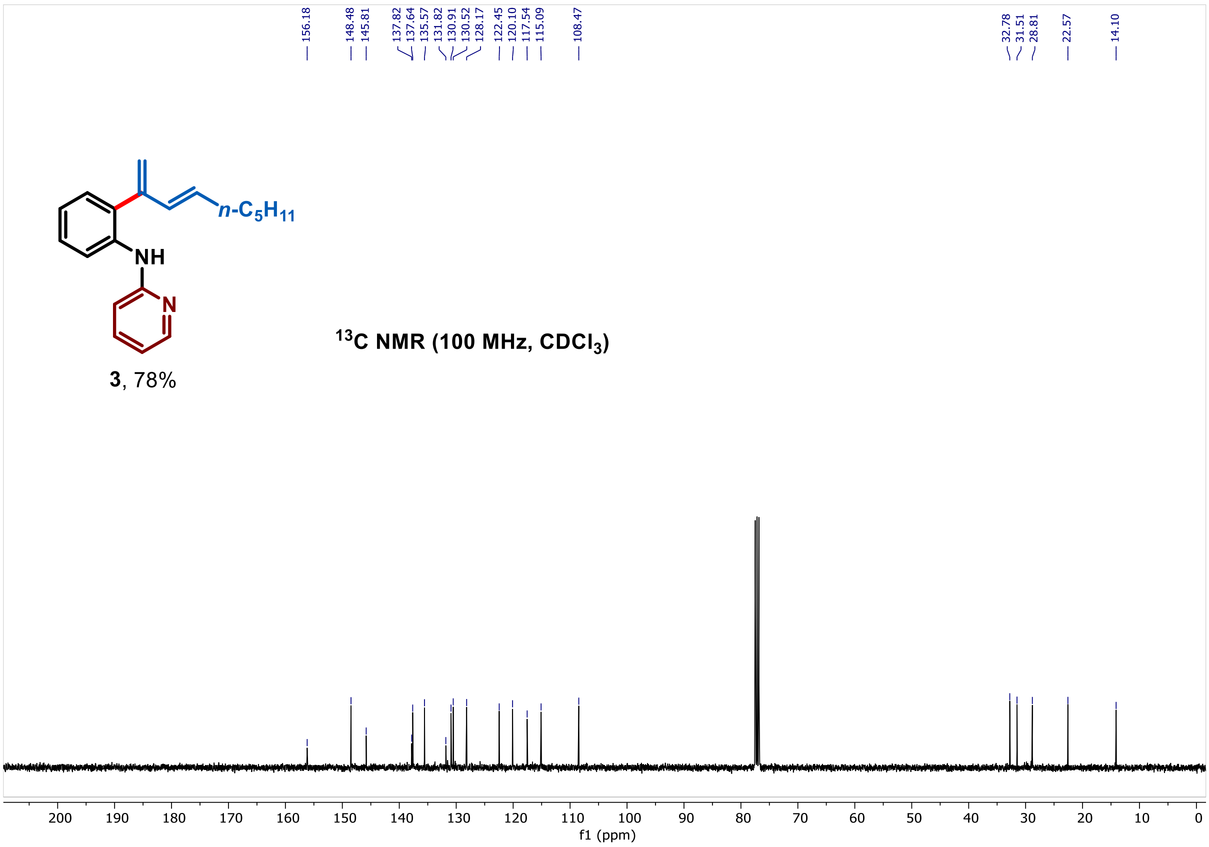

n-C5H11
NH
N
3, 78%
13C NMR (100 MHz, CDCl3)
156.18
148.48
145.81
137.82
137.64
135.57
131.82
130.91
130.52
128.17
122.45
120.10
117.54
115.09
108.47
32.78
31.51
28.81
22.57
14.10
f1 (ppm)

4

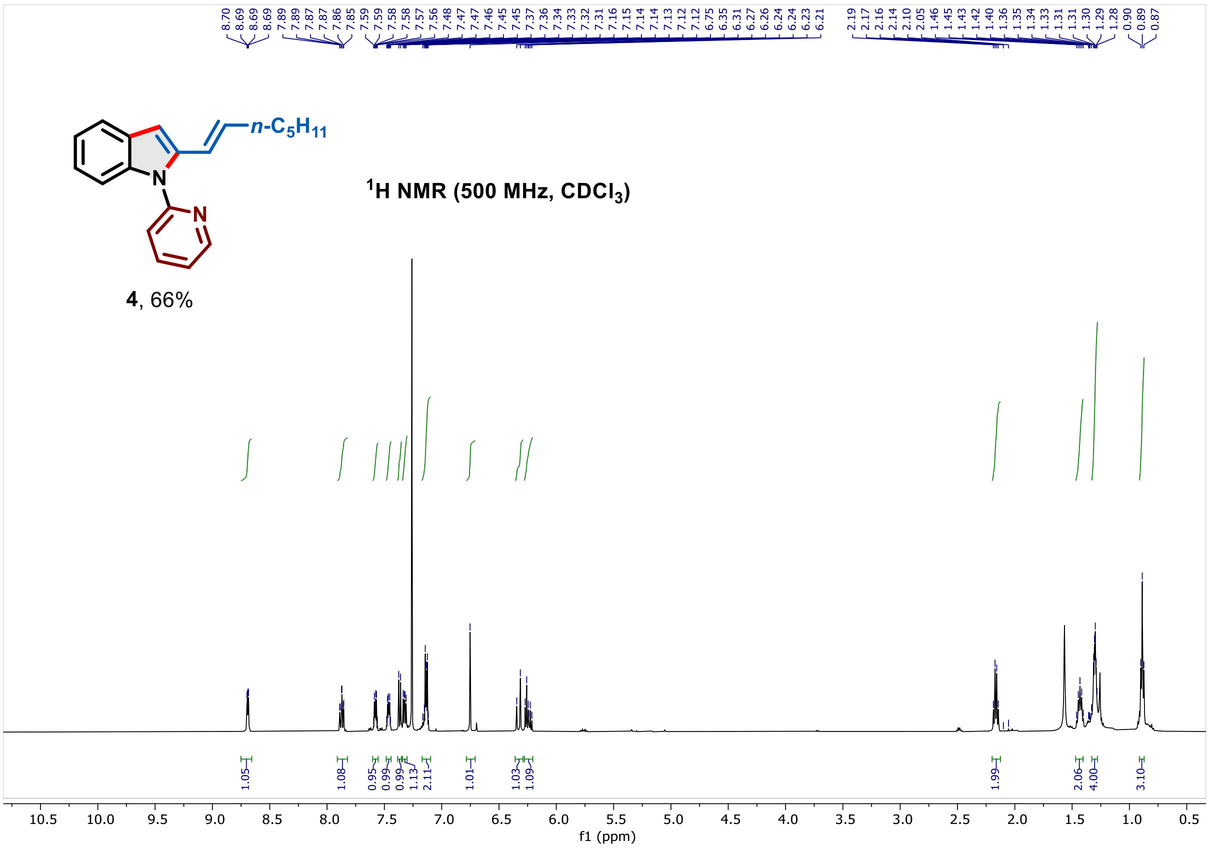
^{1}H NMR (500 MHz, CDCl$_3$)
n-C$_5$H$_{11}$
N
N
4, 66%
8.70 8.69 8.69 8.69 7.89 7.89 7.87 7.87 7.86 7.85 7.59 7.59 7.58 7.58 7.57 7.56 7.48 7.47 7.47 7.46 7.45 7.45 7.37 7.36 7.34 7.33 7.32 7.31 7.16 7.15 7.14 7.14 7.13 7.13 7.12 7.12 6.75 6.35 6.31 6.27 6.26 6.24 6.24 6.23 6.21 2.19 2.17 2.16 2.14 2.10 2.05 1.46 1.45 1.43 1.42 1.40 1.36 1.35 1.34 1.33 1.31 1.31 1.30 1.29 1.28 0.90 0.89 0.87
1.05 1.08 0.95 0.99 0.99 1.13 2.11 1.01 1.03 1.09 1.99 2.06 4.00 3.10
10.5 10.0 9.5 9.0 8.5 8.0 7.5 7.0 6.5 6.0 5.5 5.0 4.5 4.0 3.5 3.0 2.5 2.0 1.5 1.0 0.5
f1 (ppm)

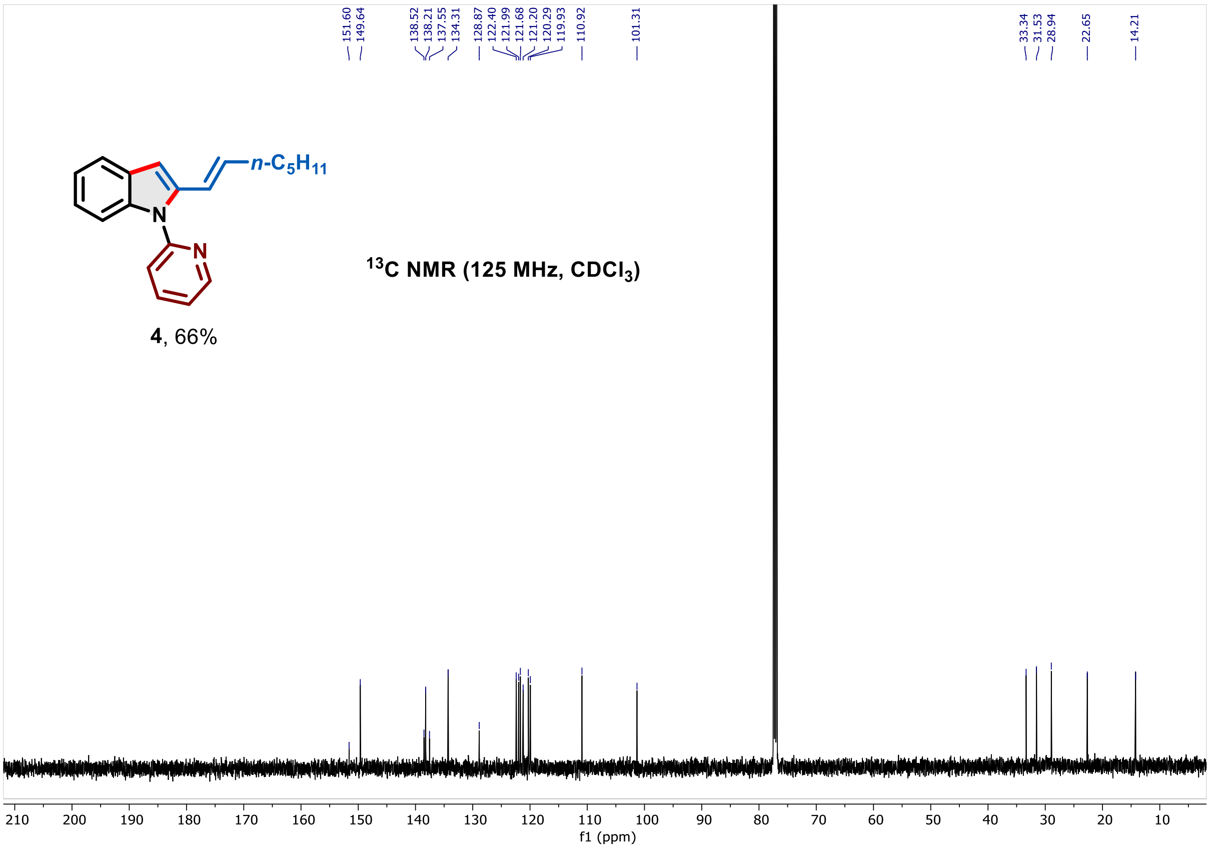
^{13}C NMR (125 MHz, CDCl$_3$)
n-C$_5$H$_{11}$
N
N
4, 66%
151.60 149.64 138.52 138.21 137.55 134.31 128.87 122.40 121.99 121.68 121.20 120.29 119.93 110.92 101.31 33.34 31.53 28.94 22.65 14.21
210 200 190 180 170 160 150 140 130 120 110 100 90 80 70 60 50 40 30 20 10
f1 (ppm)

11

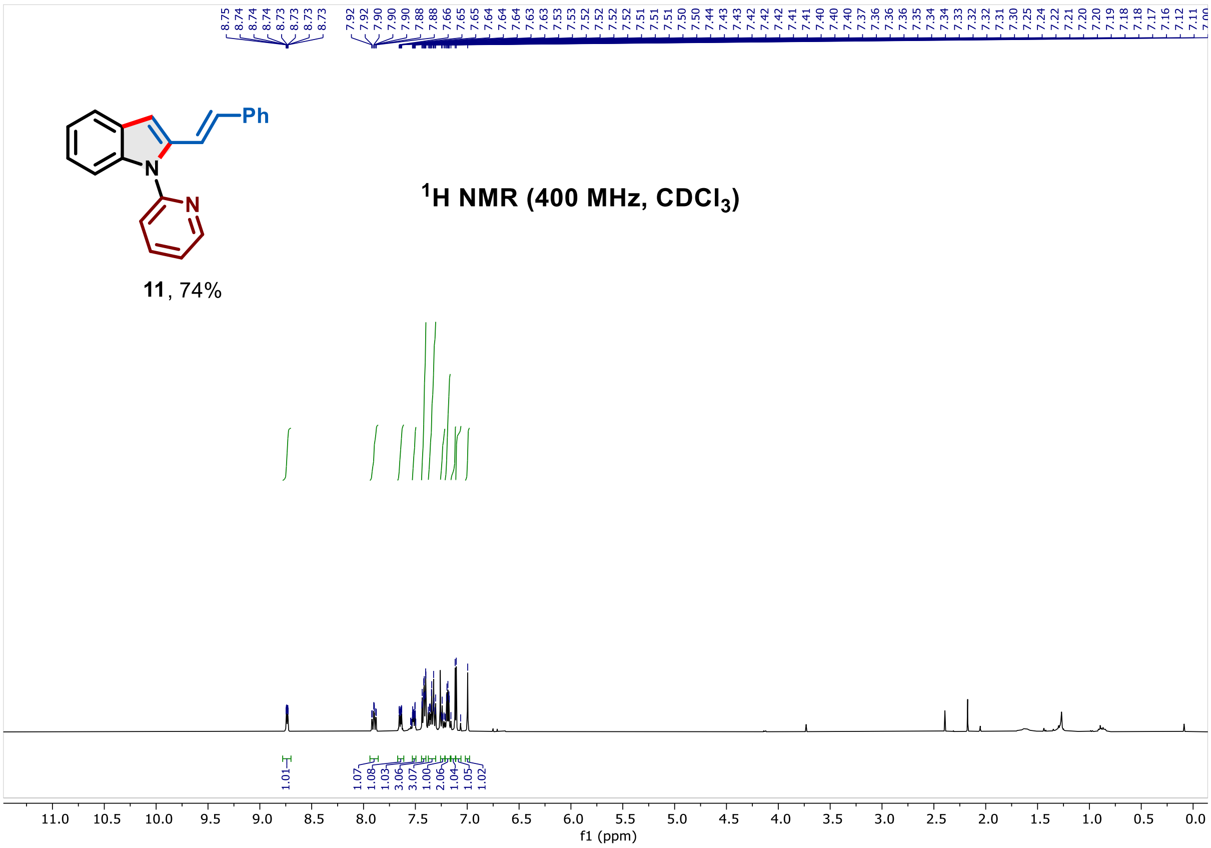
Ph
N
N
11, 74%
1H NMR (400 MHz, CDCl3)
f1 (ppm)

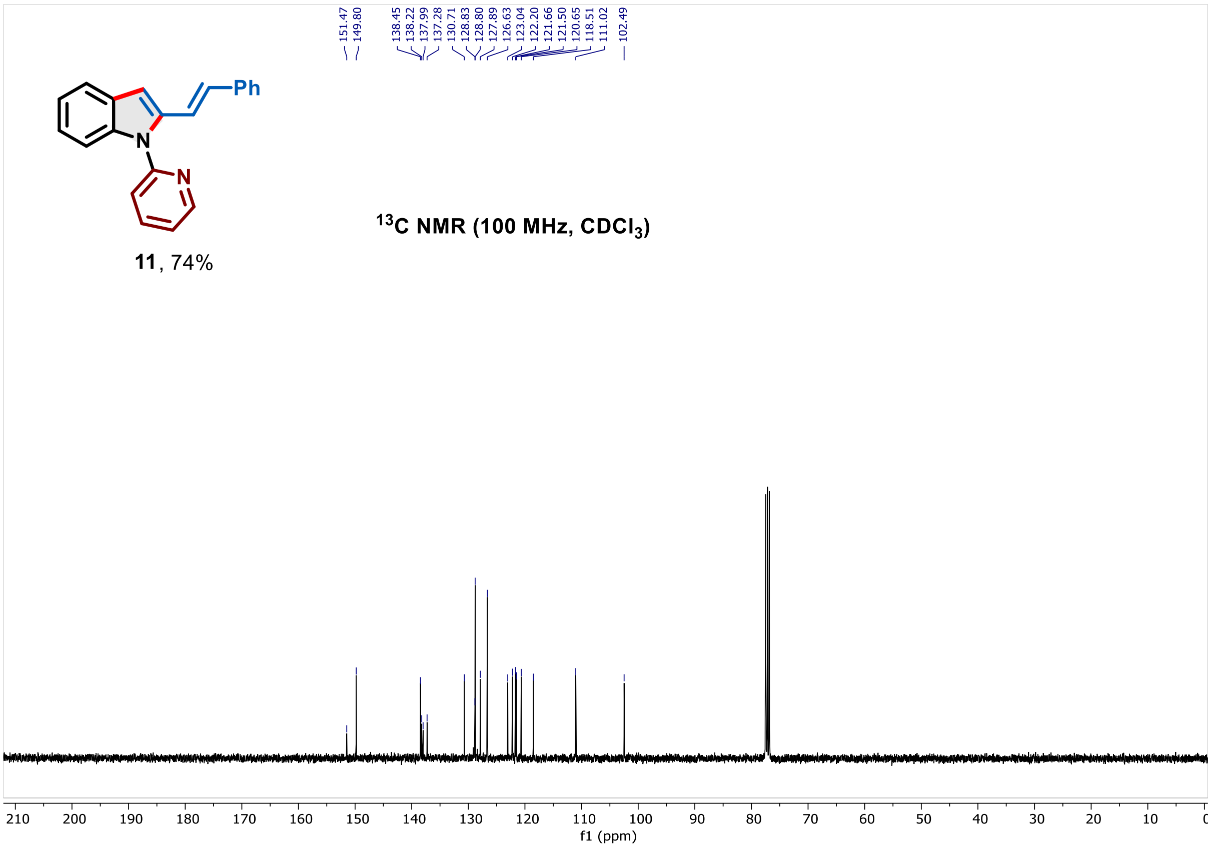
151.47
149.80
138.45
138.22
137.99
137.28
130.71
128.83
128.80
127.89
126.63
123.04
122.20
121.66
121.50
120.65
118.51
111.02
102.49
Ph
N
N
11, 74%
13C NMR (100 MHz, CDCl3)
f1 (ppm)

12

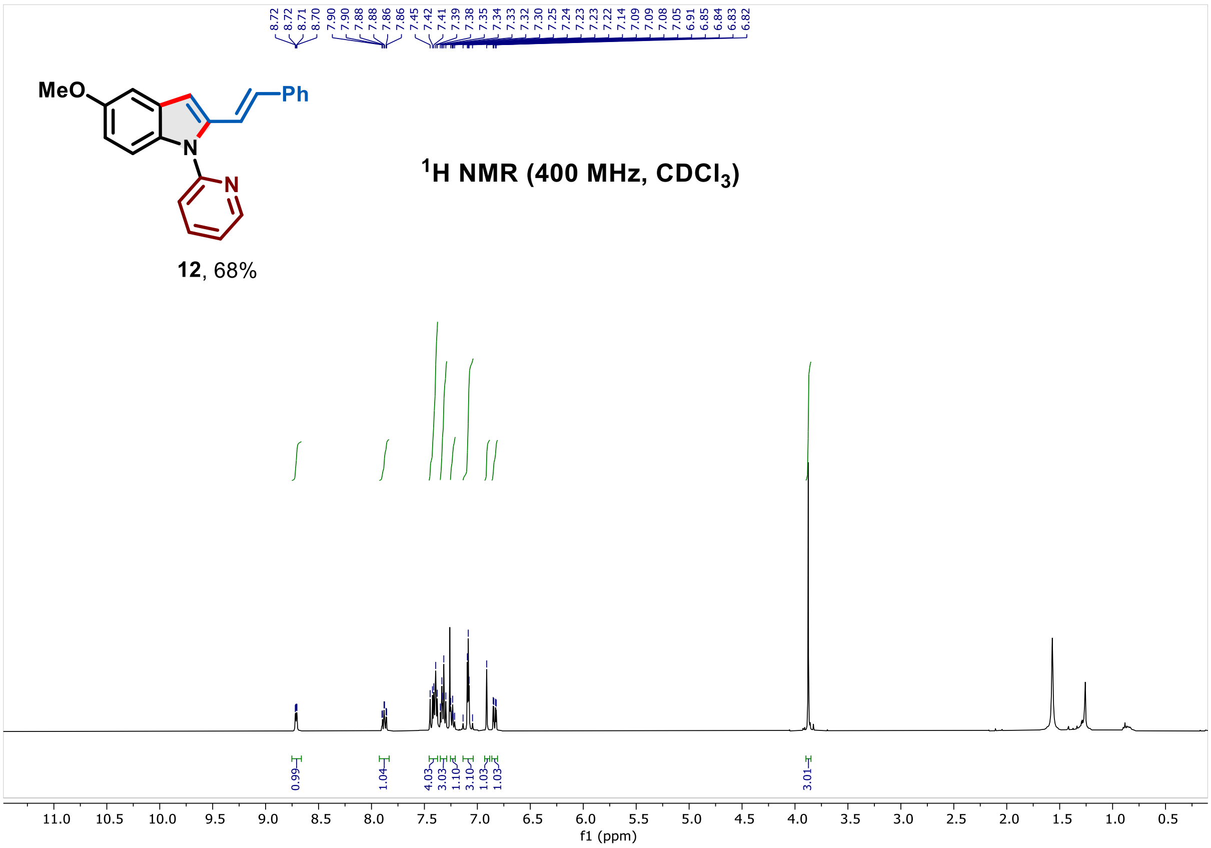
MeO
Ph
N
N
12, 68%
1H NMR (400 MHz, CDCl3)
f1 (ppm)

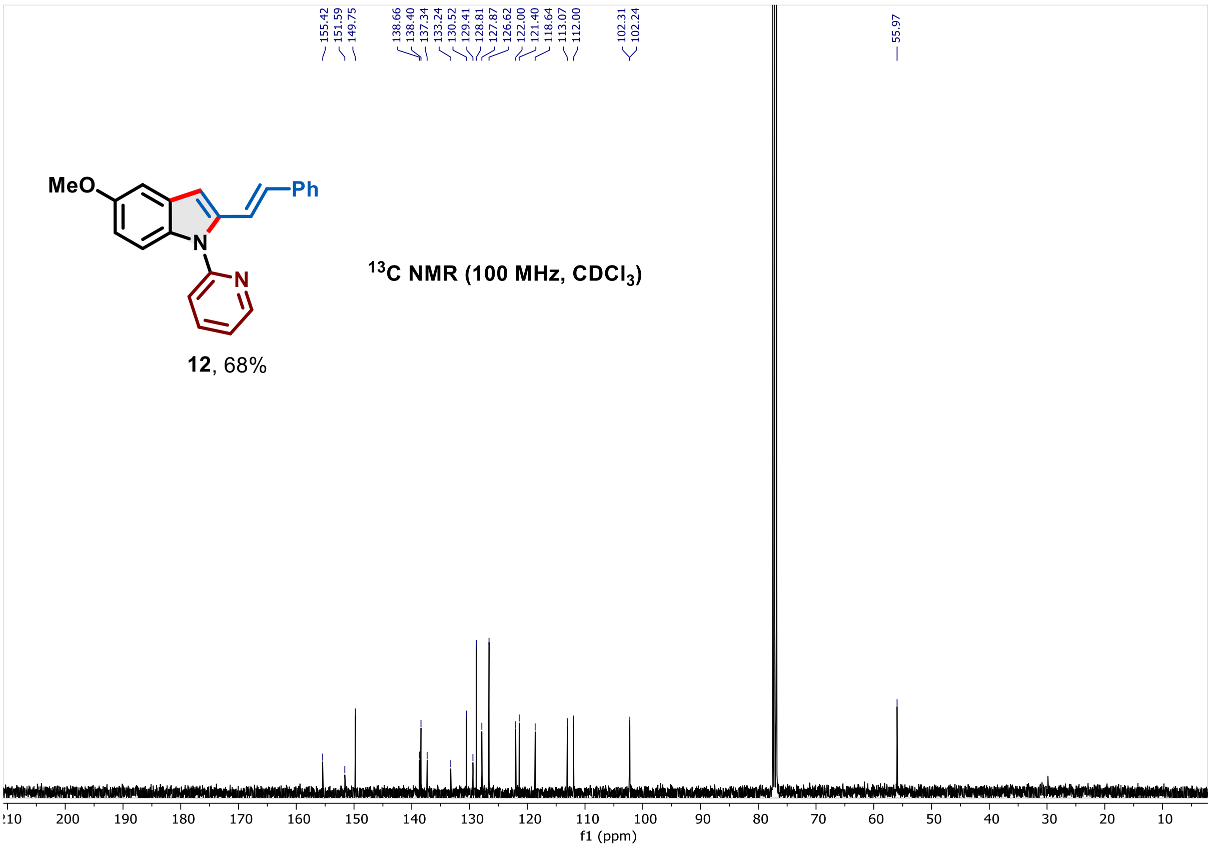
MeO
Ph
N
N
12, 68%
13C NMR (100 MHz, CDCl3)
f1 (ppm)

13

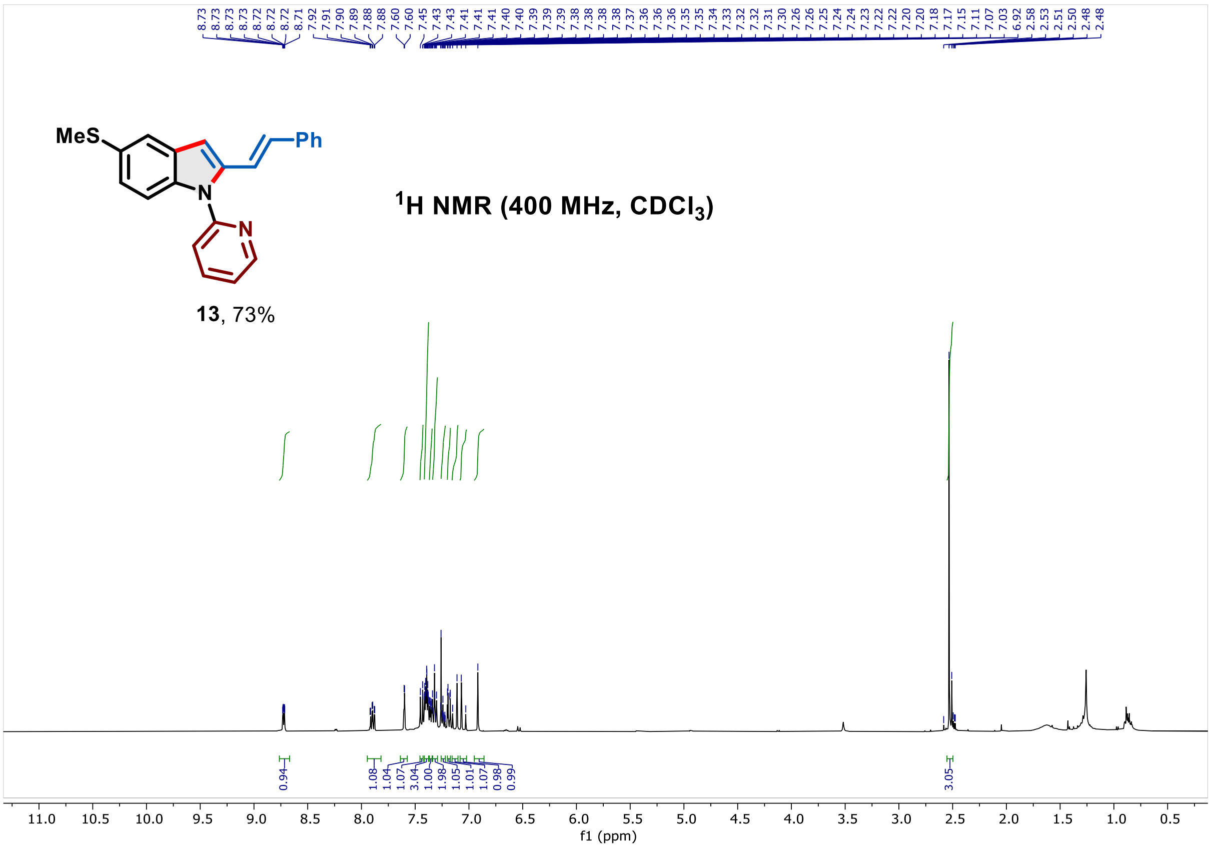
8.73 8.73 8.73 8.72 8.72 8.71 7.92 7.91 7.90 7.89 7.88 7.88 7.60 7.60 7.45 7.43 7.43 7.41 7.41 7.40 7.40 7.39 7.39 7.39 7.38 7.38 7.38 7.38 7.37 7.36 7.36 7.36 7.35 7.35 7.34 7.33 7.32 7.32 7.31 7.30 7.26 7.26 7.25 7.24 7.24 7.23 7.22 7.22 7.20 7.20 7.18 7.17 7.15 7.11 7.07 7.03 6.92 2.58 2.53 2.51 2.50 2.48 2.48
MeS
Ph
N
N
13, 73%
^{1}H NMR (400 MHz, CDCl$_3$)
0.94 1.08 1.04 1.07 3.04 1.00 1.98 1.05 1.01 1.07 0.98 0.99 3.05
11.0 10.5 10.0 9.5 9.0 8.5 8.0 7.5 7.0 6.5 6.0 5.5 5.0 4.5 4.0 3.5 3.0 2.5 2.0 1.5 1.0 0.5
f1 (ppm)

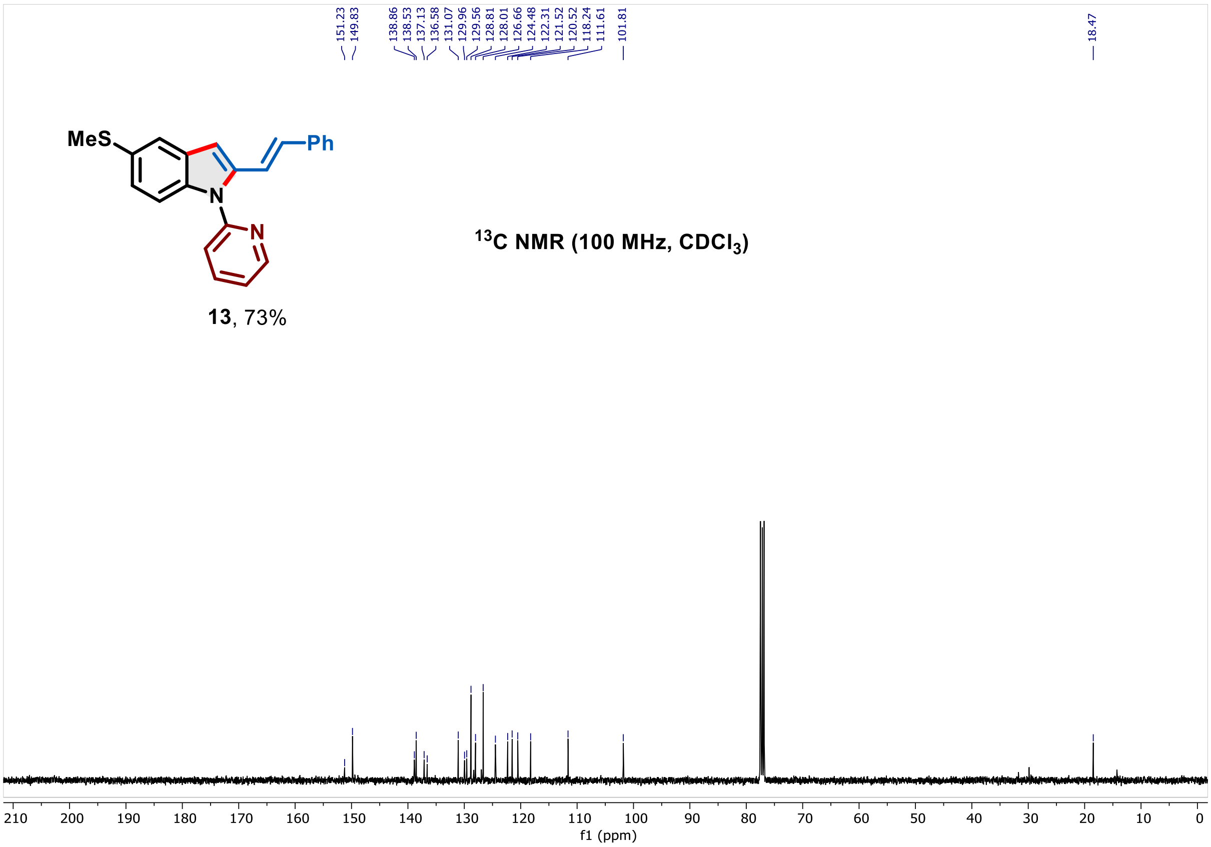
151.23 149.83 138.86 138.53 137.13 136.58 131.07 129.96 129.56 128.81 128.01 126.66 124.48 122.31 121.52 120.52 118.24 111.61 101.81 18.47
MeS
Ph
N
N
13, 73%
^{13}C NMR (100 MHz, CDCl$_3$)
210 200 190 180 170 160 150 140 130 120 110 100 90 80 70 60 50 40 30 20 10 0
f1 (ppm)

14

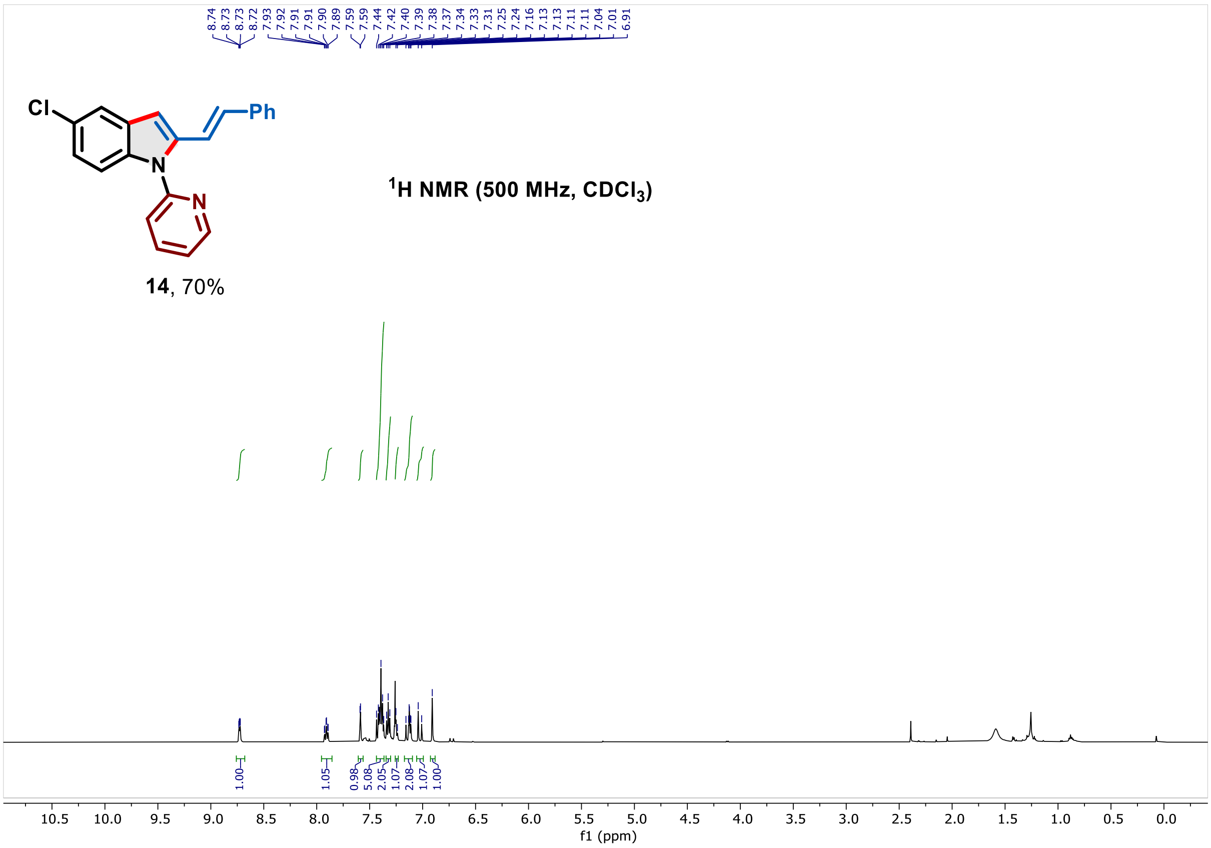
Cl
Ph
N
N
14, 70%
1H NMR (500 MHz, CDCl3)
f1 (ppm)

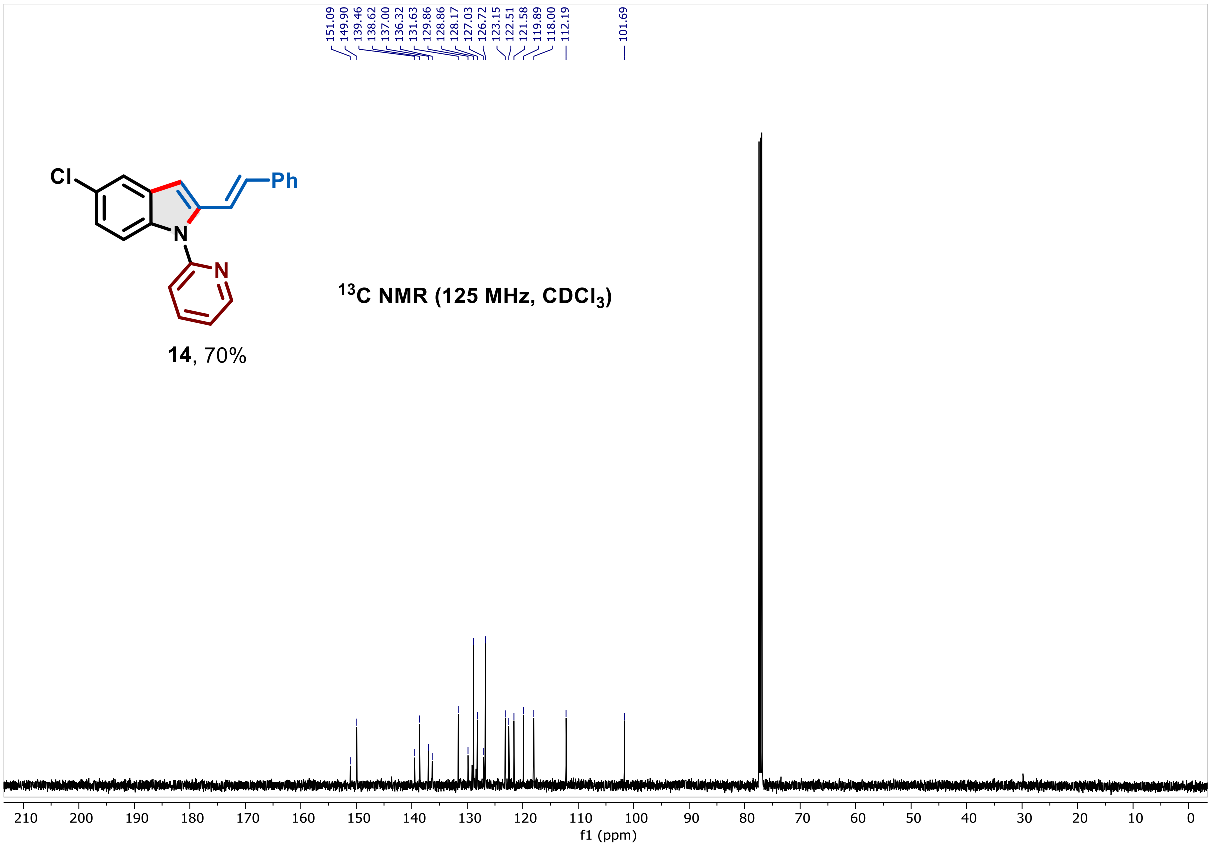
Cl
Ph
N
N
14, 70%
13C NMR (125 MHz, CDCl3)
f1 (ppm)

15

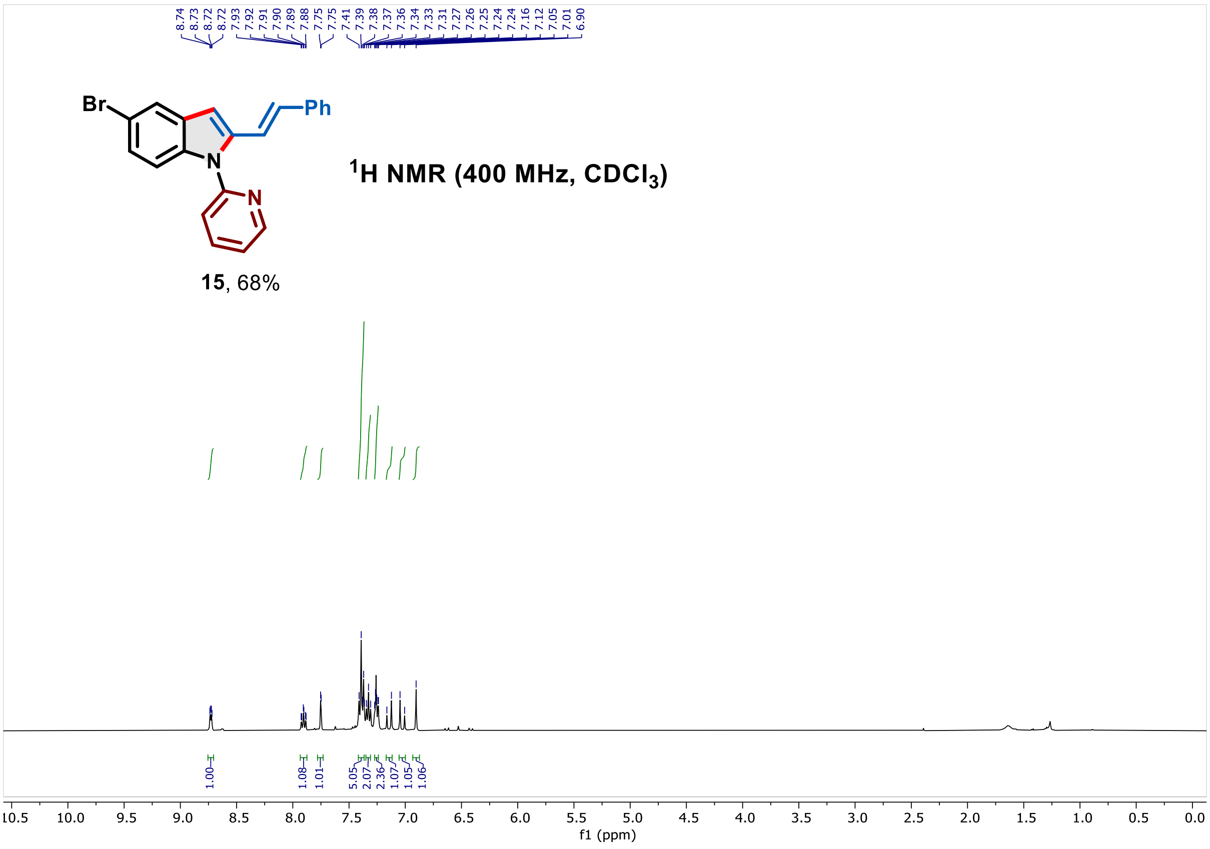

Br
Ph
N
N
15, 68%
^{1}H NMR (400 MHz, CDCl$_3$)

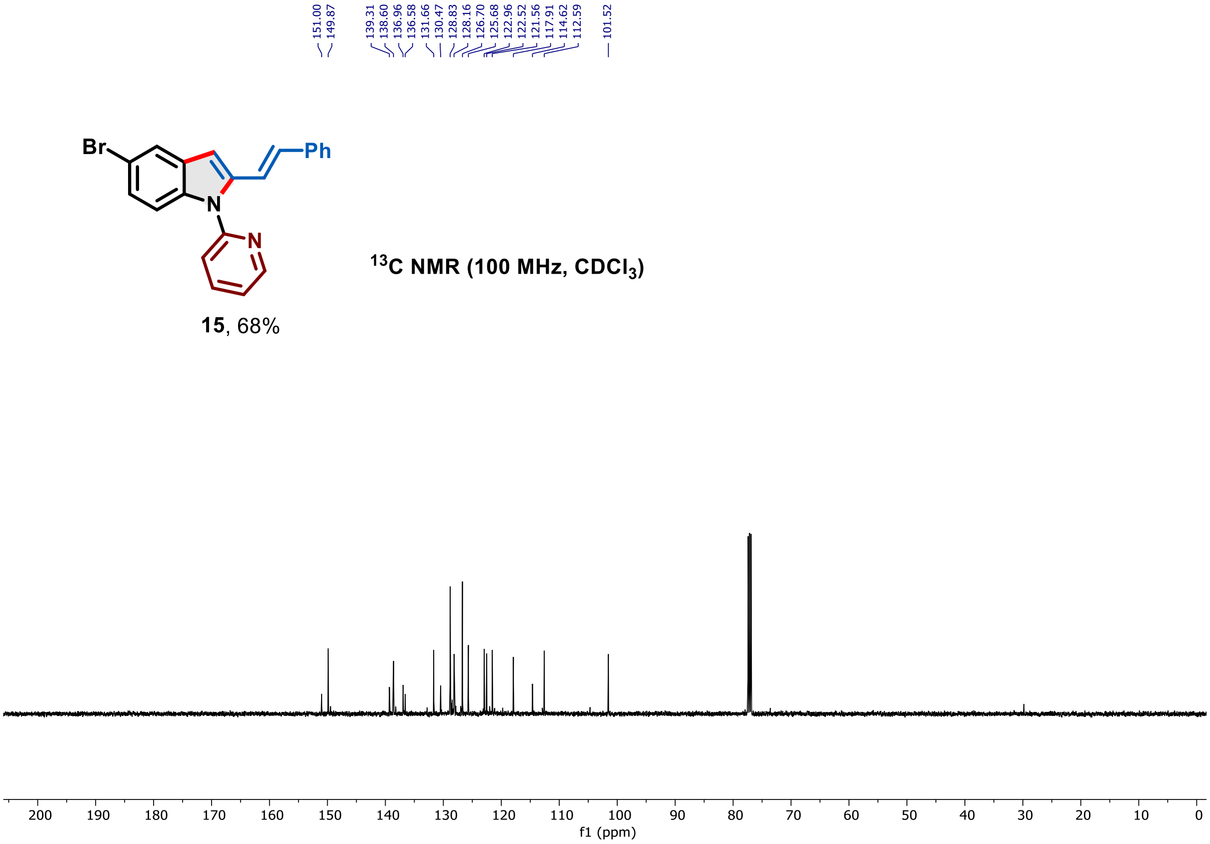

Br
Ph
N
N
15, 68%
^{13}C NMR (100 MHz, CDCl$_3$)

16

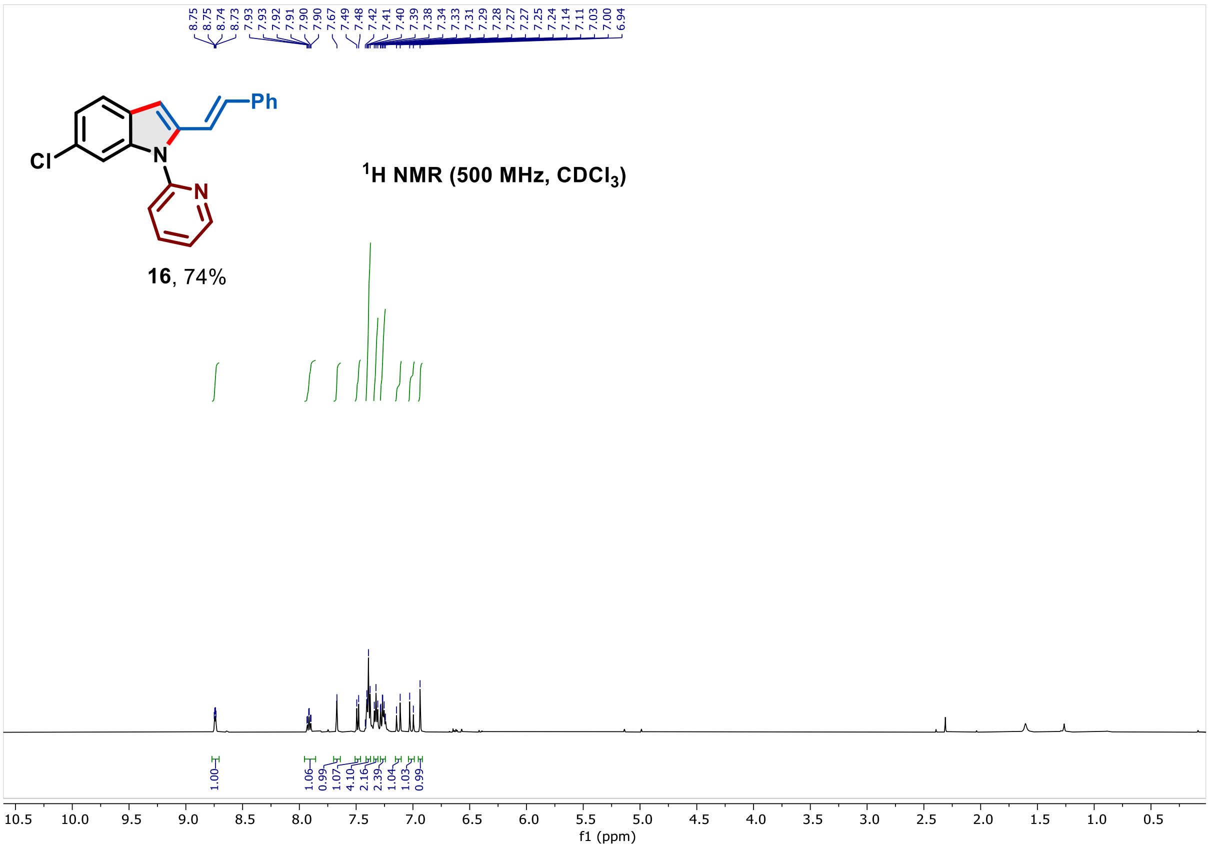
Cl
N
N
Ph
16, 74%
1H NMR (500 MHz, CDCl3)
f1 (ppm)

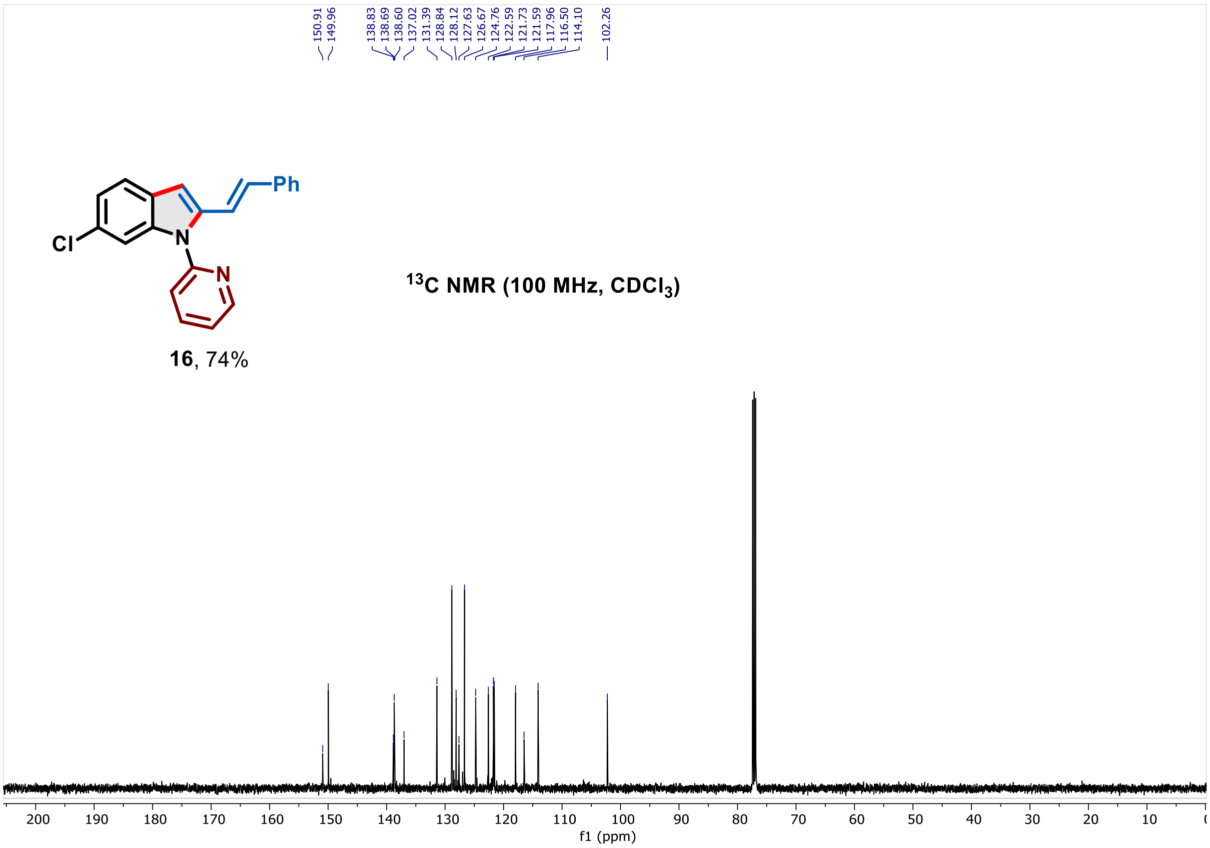
Cl
N
N
Ph
16, 74%
13C NMR (100 MHz, CDCl3)
f1 (ppm)

17

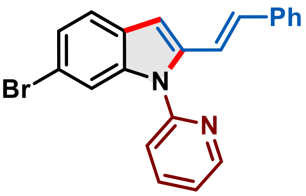

9, 71%

^{1}H NMR (400 MHz, $CDCl_3$)

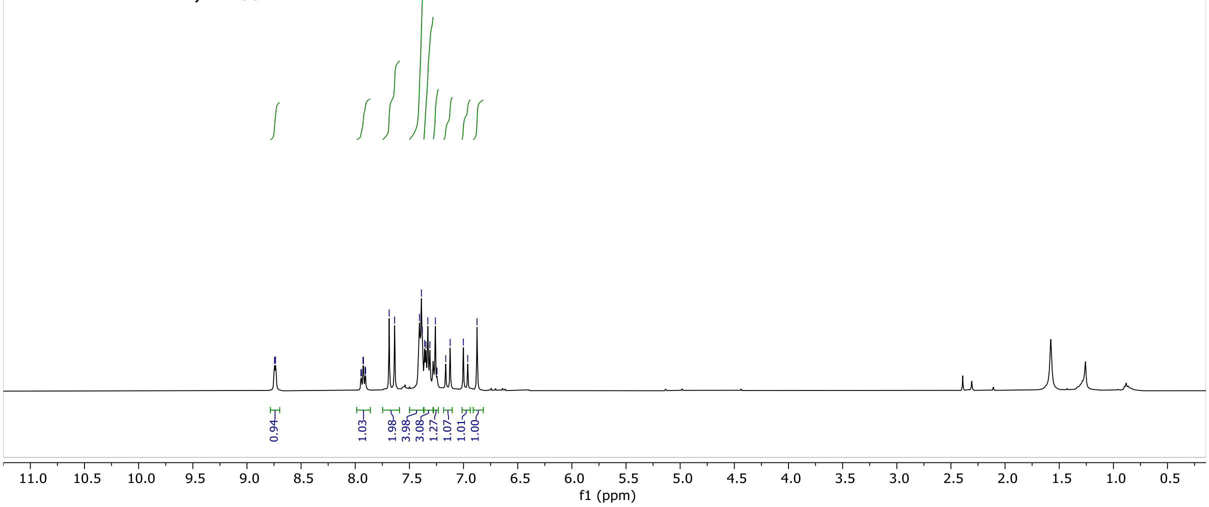

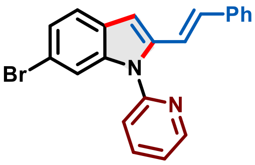

17, 71%

^{13}C NMR (100 MHz, $CDCl_3$)

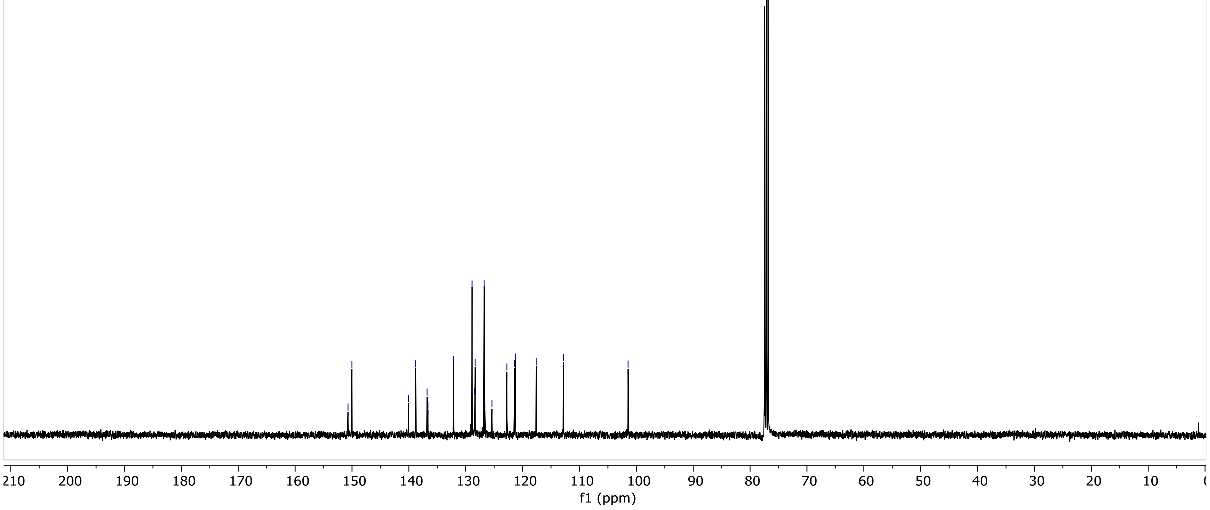

18

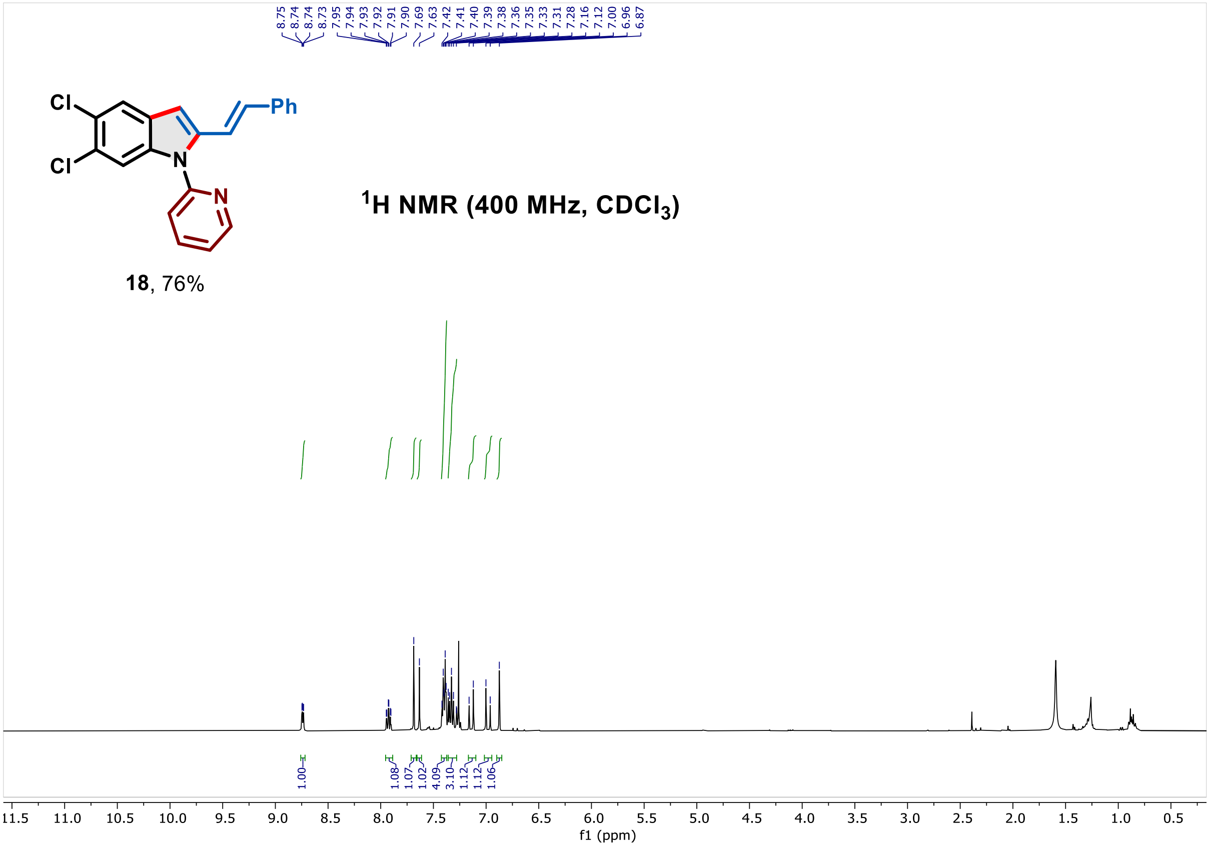
8.75
8.74
8.74
8.73
7.95
7.94
7.93
7.92
7.91
7.90
7.69
7.63
7.42
7.41
7.40
7.39
7.38
7.36
7.35
7.33
7.31
7.28
7.16
7.12
7.00
6.96
6.87
Cl
Cl
N
N
Ph
18, 76%
1H NMR (400 MHz, CDCl3)
1.00
1.08
1.07
1.02
4.09
3.10
1.12
1.12
1.06
f1 (ppm)

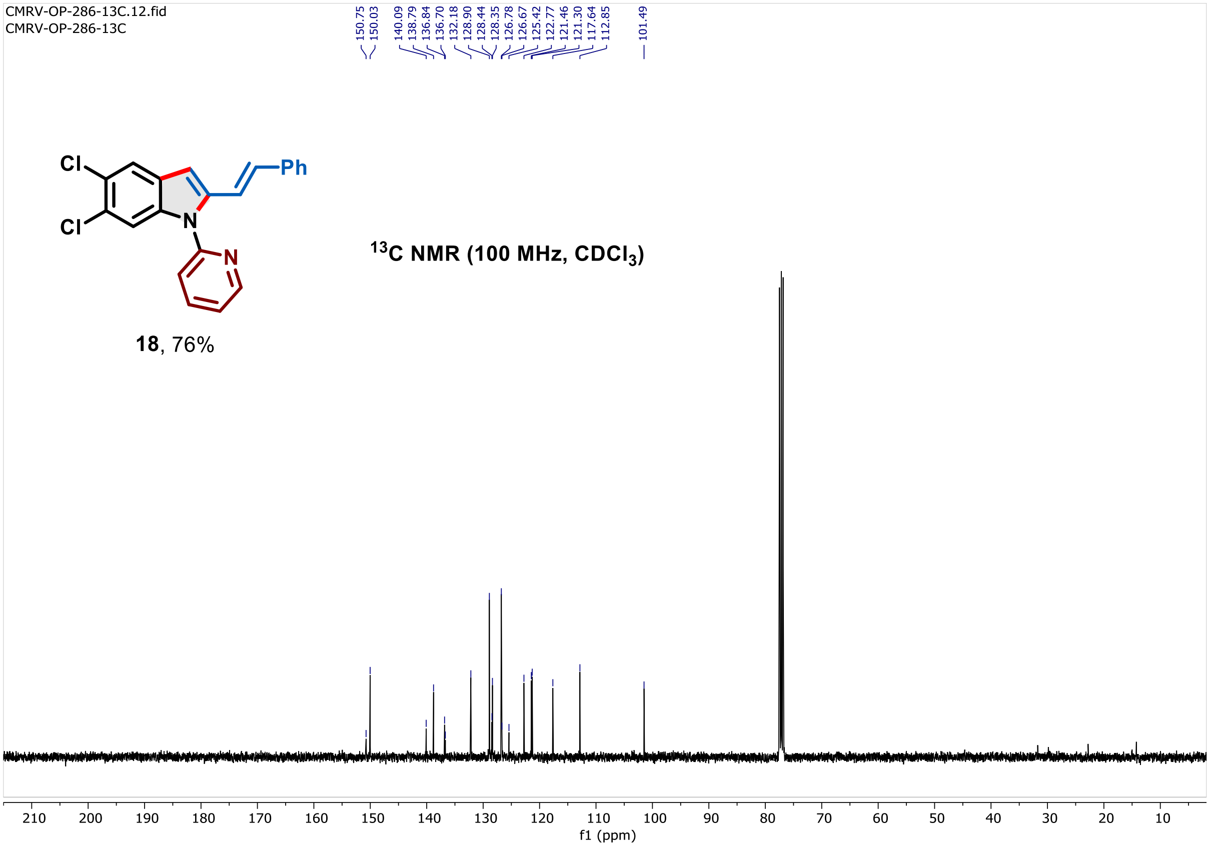
CMRV-OP-286-13C.12.fid
CMRV-OP-286-13C
150.75
150.03
140.09
138.79
136.84
136.70
132.18
128.90
128.44
128.35
126.78
126.67
125.42
122.77
121.46
121.30
117.64
112.85
101.49
Cl
Cl
N
N
Ph
18, 76%
13C NMR (100 MHz, CDCl3)
f1 (ppm)

19

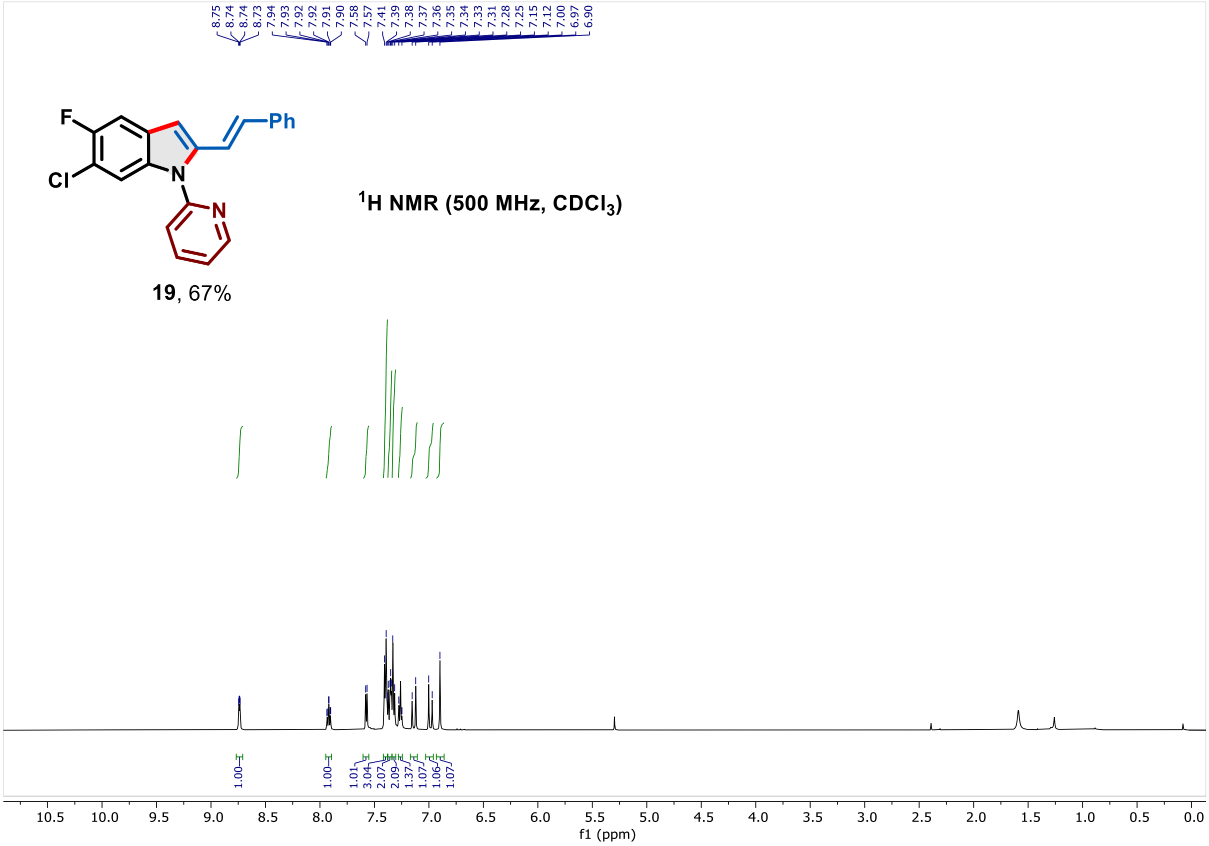
8.75
8.74
8.74
8.73
7.94
7.93
7.92
7.92
7.91
7.90
7.58
7.57
7.41
7.39
7.38
7.37
7.36
7.35
7.34
7.33
7.31
7.28
7.25
7.15
7.12
7.00
6.97
6.90
F
Cl
N
N
Ph
19, 67%
1H NMR (500 MHz, CDCl3)
1.00
1.00
1.01
3.04
2.07
2.09
1.37
1.07
1.06
1.07
10.5
10.0
9.5
9.0
8.5
8.0
7.5
7.0
6.5
6.0
5.5
5.0
4.5
4.0
3.5
3.0
2.5
2.0
1.5
1.0
0.5
0.0
f1 (ppm)

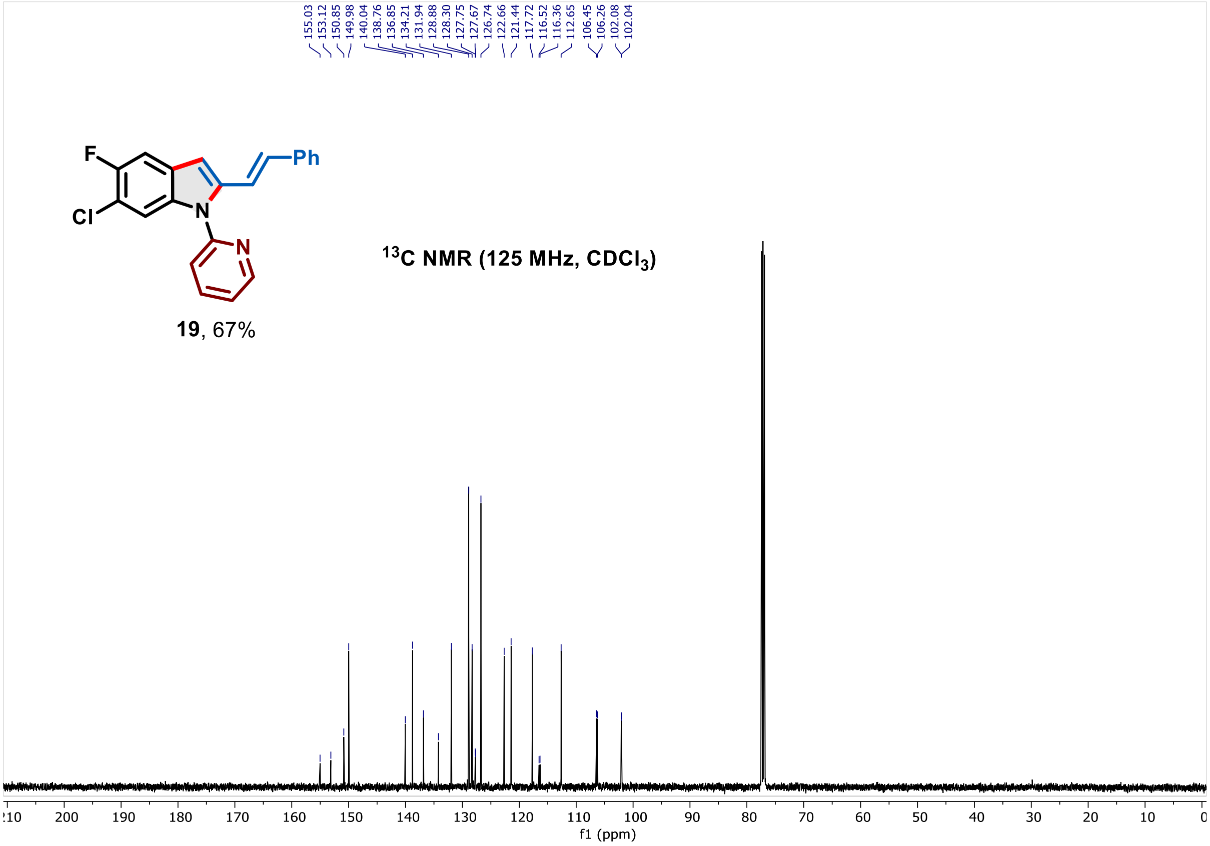
155.03
153.12
150.85
149.98
140.04
138.76
136.85
134.21
131.94
128.88
128.30
127.75
127.67
126.74
122.66
121.44
117.72
116.52
116.36
112.65
106.45
106.26
102.08
102.04
F
Cl
N
N
Ph
19, 67%
13C NMR (125 MHz, CDCl3)
210
200
190
180
170
160
150
140
130
120
110
100
90
80
70
60
50
40
30
20
10
0
f1 (ppm)

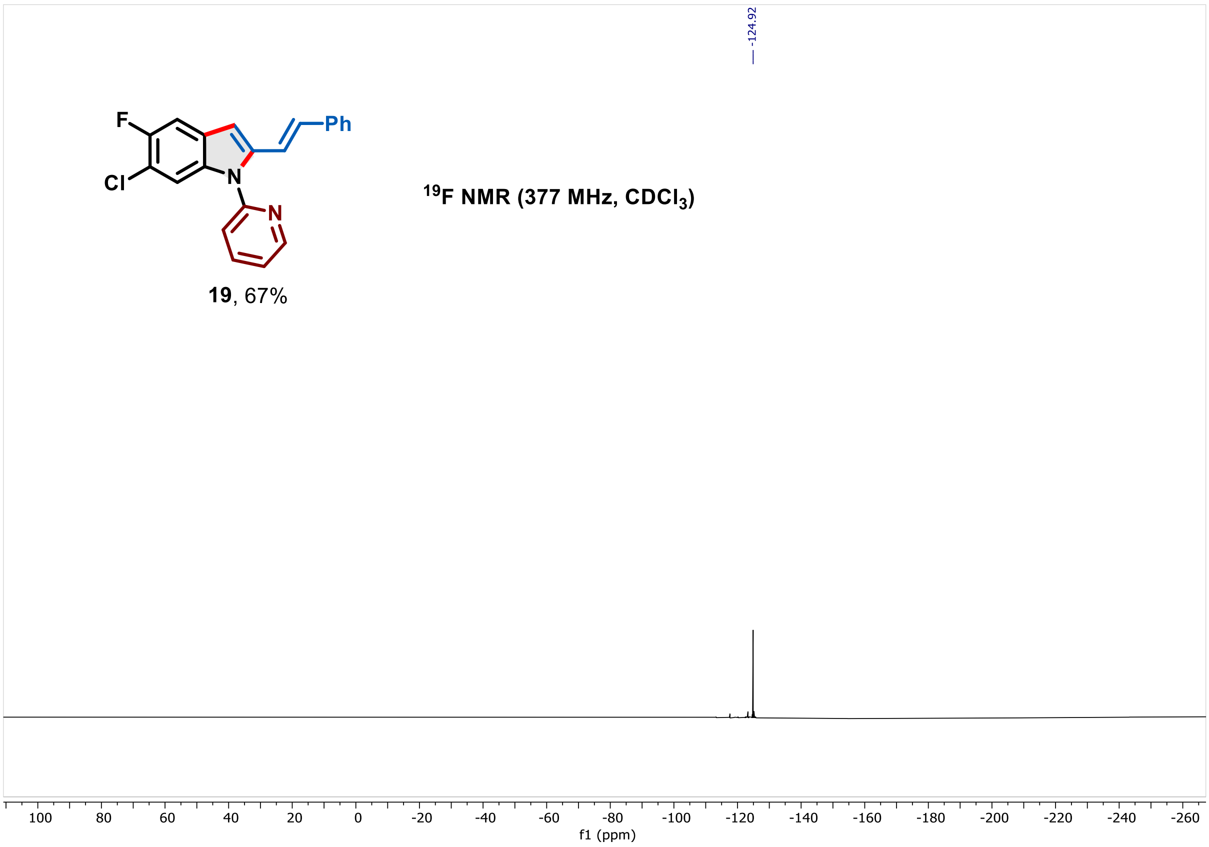
-124.92
19, 67%
19F NMR (377 MHz, CDCl3)
f1 (ppm)

20

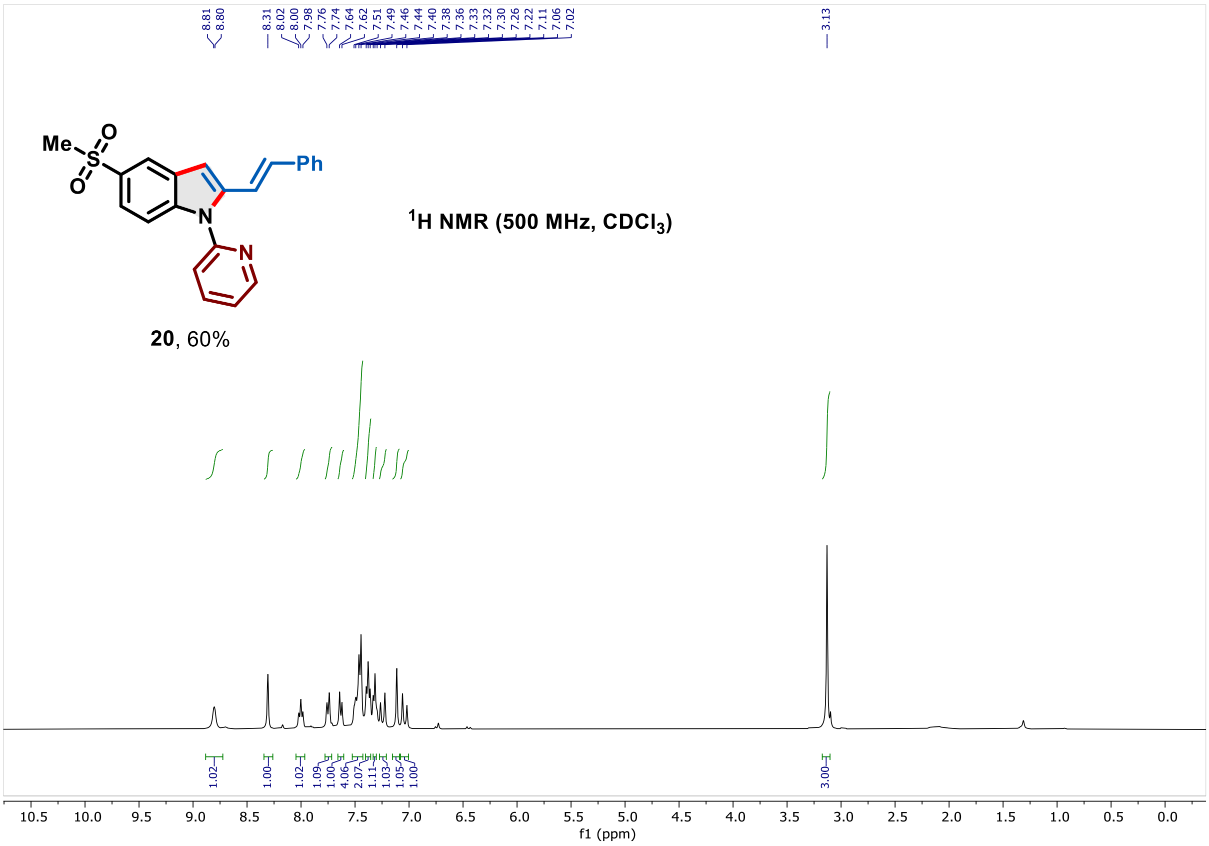
8.81
8.80
8.31
8.02
8.00
7.98
7.76
7.74
7.64
7.62
7.51
7.49
7.46
7.44
7.40
7.38
7.36
7.33
7.32
7.30
7.26
7.22
7.11
7.06
7.02
3.13
20, 60%
1H NMR (500 MHz, CDCl3)
f1 (ppm)

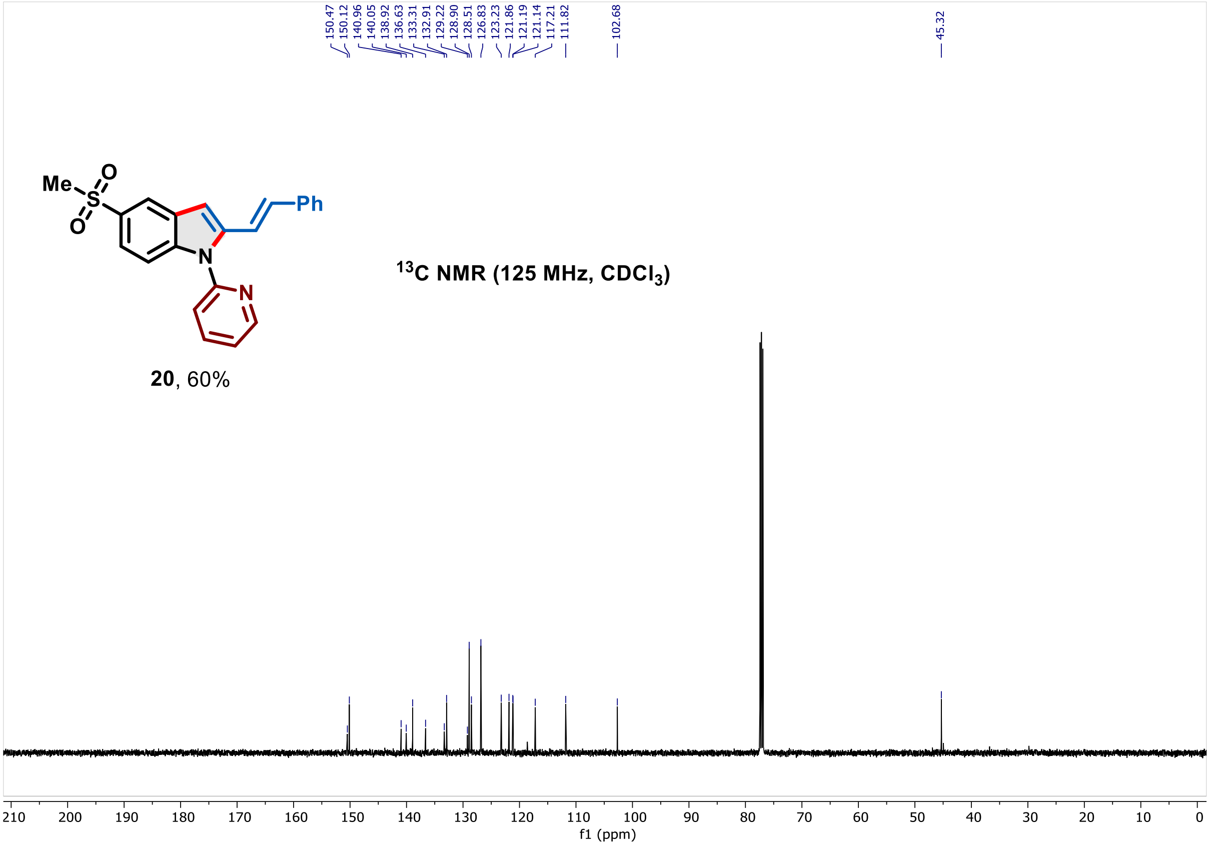
150.47
150.12
140.96
140.05
138.92
136.63
133.31
132.91
129.22
128.90
128.51
126.83
123.23
121.86
121.19
121.14
117.21
111.82
102.68
45.32
Me
S
O
O
Ph
N
N
20, 60%
13C NMR (125 MHz, CDCl3)
210 200 190 180 170 160 150 140 130 120 110 100 90 80 70 60 50 40 30 20 10 0
f1 (ppm)

21

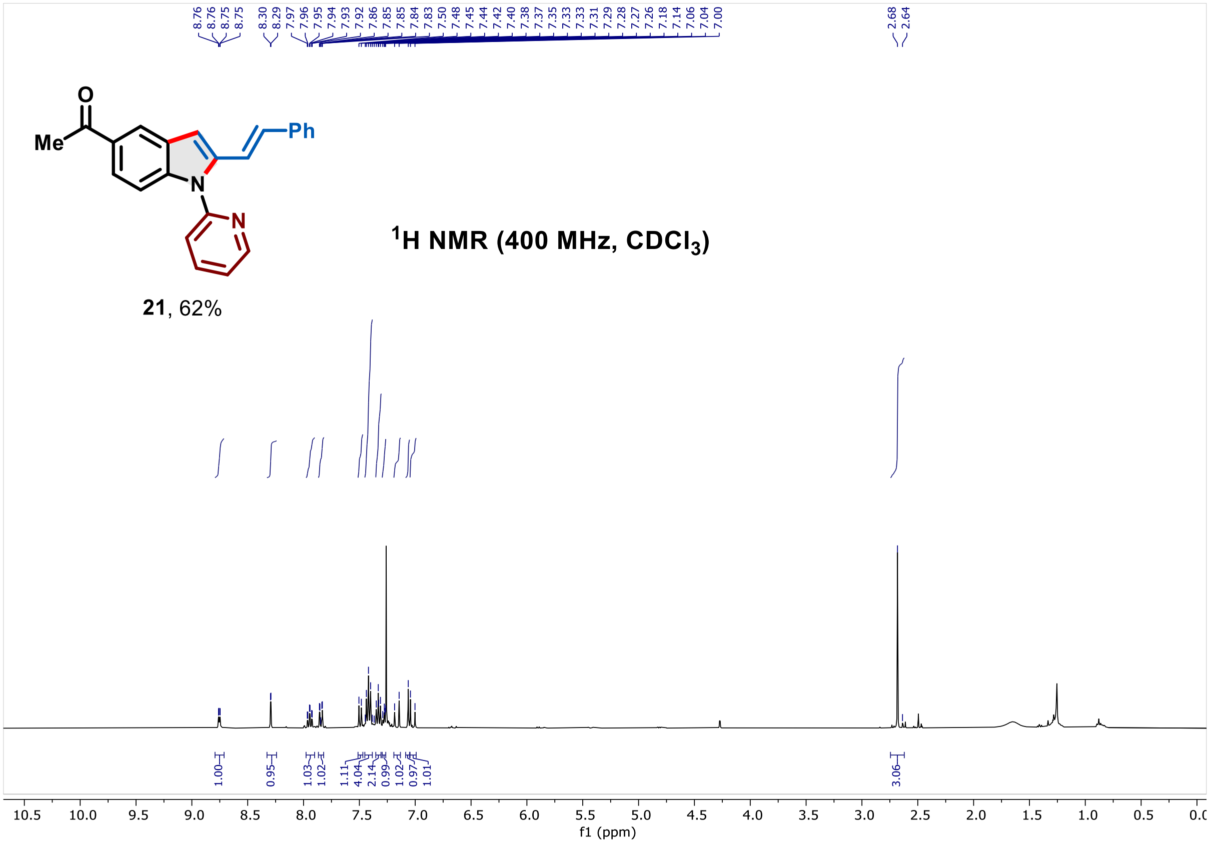
8.76
8.76
8.75
8.75
8.30
8.29
7.97
7.96
7.95
7.94
7.93
7.92
7.86
7.85
7.84
7.83
7.50
7.48
7.45
7.44
7.42
7.40
7.38
7.37
7.35
7.33
7.31
7.29
7.28
7.27
7.26
7.18
7.14
7.06
7.04
7.00
2.68
2.64
O
Me
Ph
N
N
21, 62%
1H NMR (400 MHz, CDCl3)
1.00
0.95
1.03
1.02
1.11
4.04
2.14
0.99
1.02
0.97
1.01
3.06
10.5 10.0 9.5 9.0 8.5 8.0 7.5 7.0 6.5 6.0 5.5 5.0 4.5 4.0 3.5 3.0 2.5 2.0 1.5 1.0 0.5 0.0
f1 (ppm)

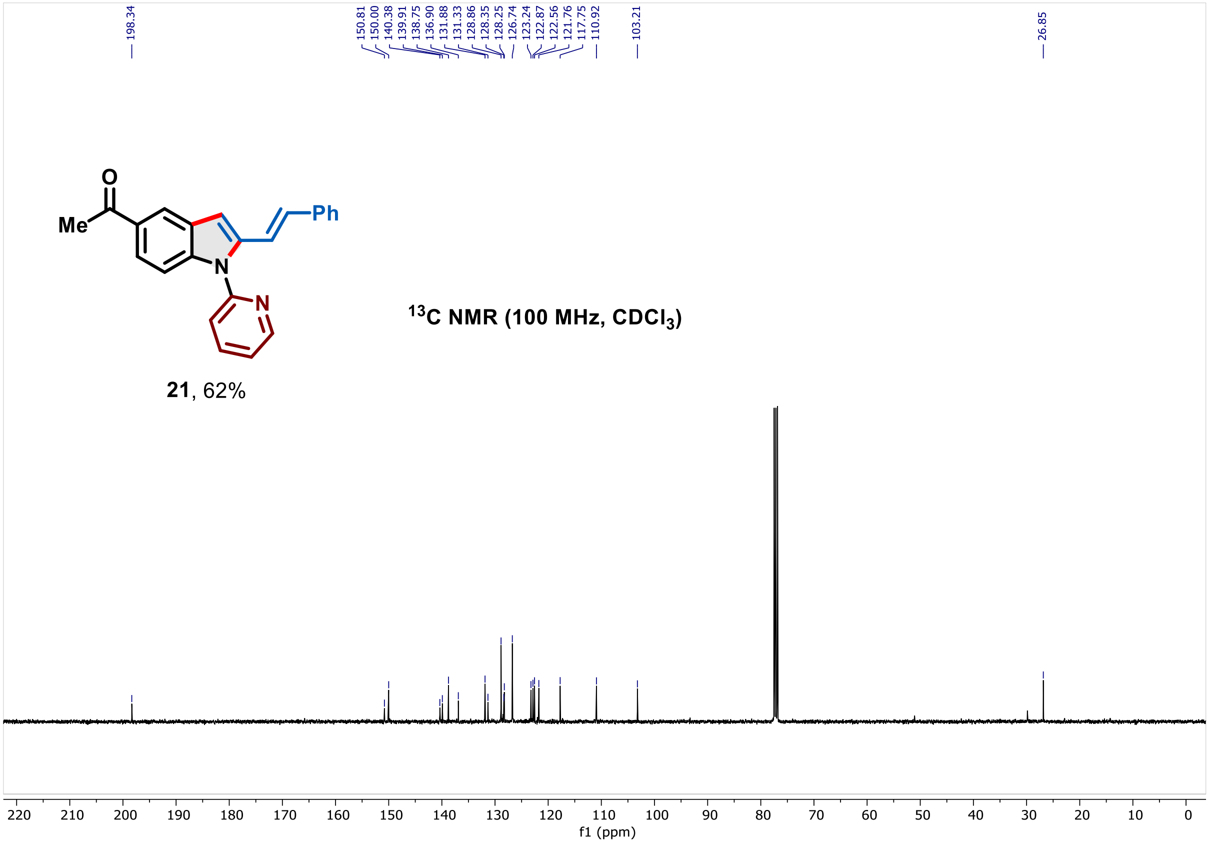
198.34
150.81
150.00
140.38
139.91
138.75
136.90
131.88
131.33
128.86
128.35
128.25
126.74
123.24
122.87
122.56
121.76
117.75
110.92
103.21
26.85
O
Me
N
N
Ph
21, 62%
13C NMR (100 MHz, CDCl3)
220 210 200 190 180 170 160 150 140 130 120 110 100 90 80 70 60 50 40 30 20 10 0
f1 (ppm)

22

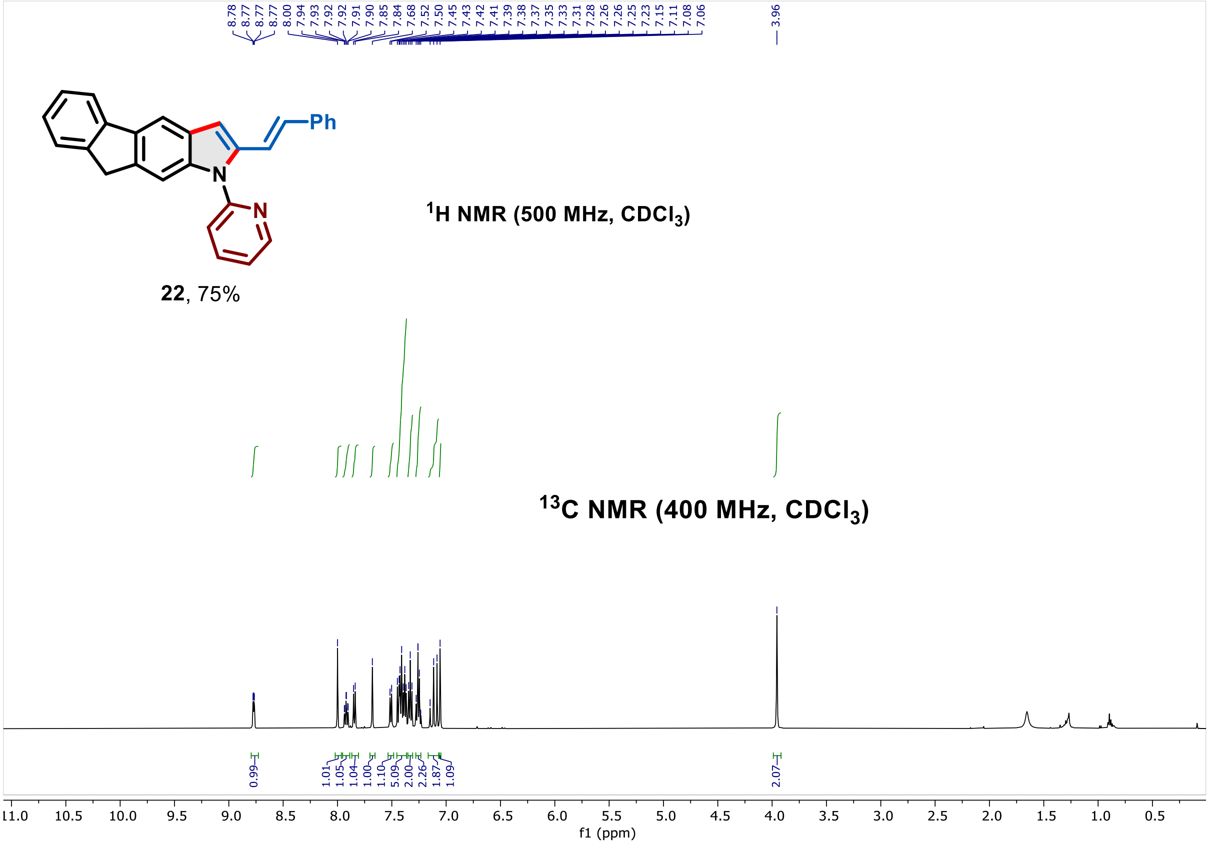
8.78
8.77
8.77
8.77
8.00
7.94
7.93
7.92
7.92
7.91
7.90
7.85
7.84
7.68
7.52
7.50
7.45
7.43
7.42
7.41
7.39
7.38
7.37
7.35
7.33
7.31
7.28
7.26
7.25
7.23
7.15
7.11
7.08
7.06
3.96
N
N
Ph
22, 75%
1H NMR (500 MHz, CDCl3)
13C NMR (400 MHz, CDCl3)
0.99
1.01
1.05
1.04
1.00
1.10
5.09
2.00
2.26
1.87
1.09
2.07
11.0 10.5 10.0 9.5 9.0 8.5 8.0 7.5 7.0 6.5 6.0 5.5 5.0 4.5 4.0 3.5 3.0 2.5 2.0 1.5 1.0 0.5
f1 (ppm)

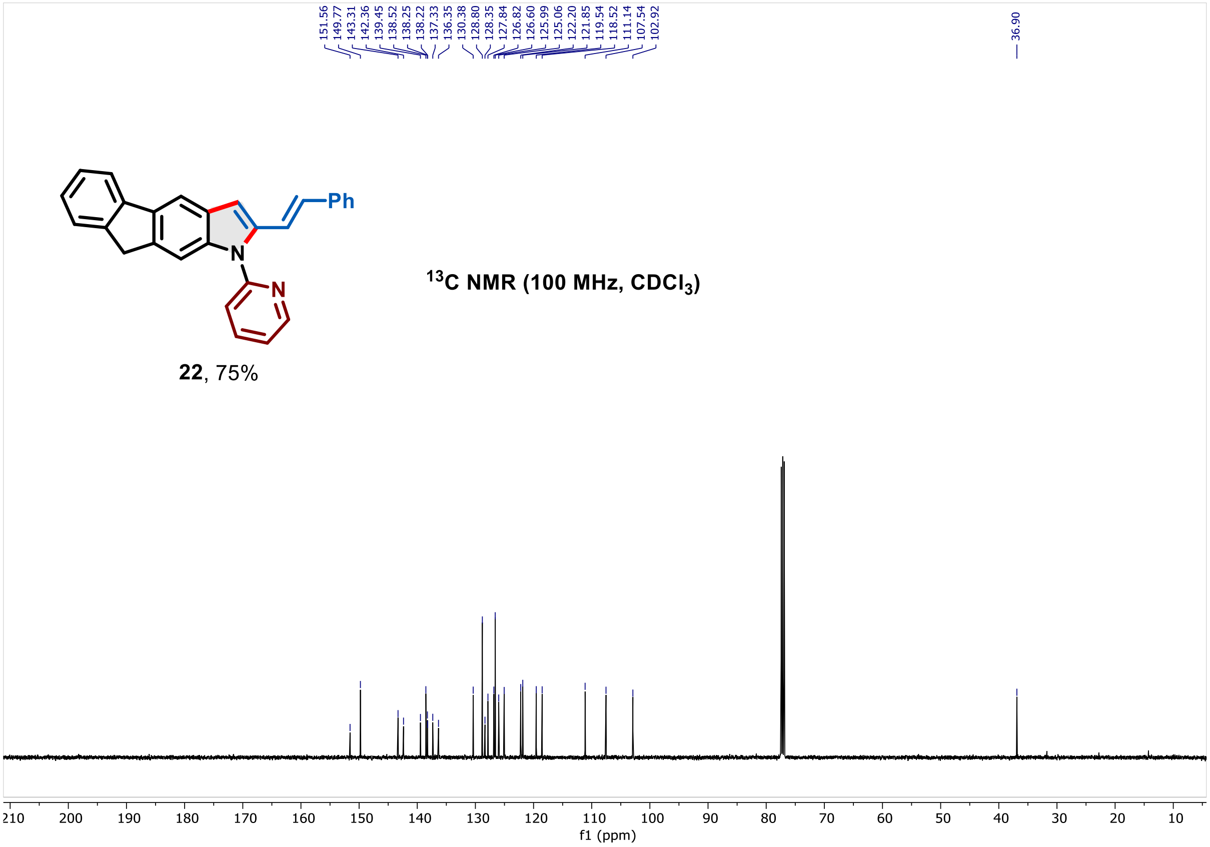
151.56 149.77 143.31 142.36 139.45 138.52 138.25 138.22 137.33 136.35 130.38 128.80 128.35 127.84 126.82 126.60 125.99 125.06 122.20 121.85 119.54 118.52 111.14 107.54 102.92
36.90
Ph
N
N
22, 75%
13C NMR (100 MHz, CDCl3)
f1 (ppm)

23

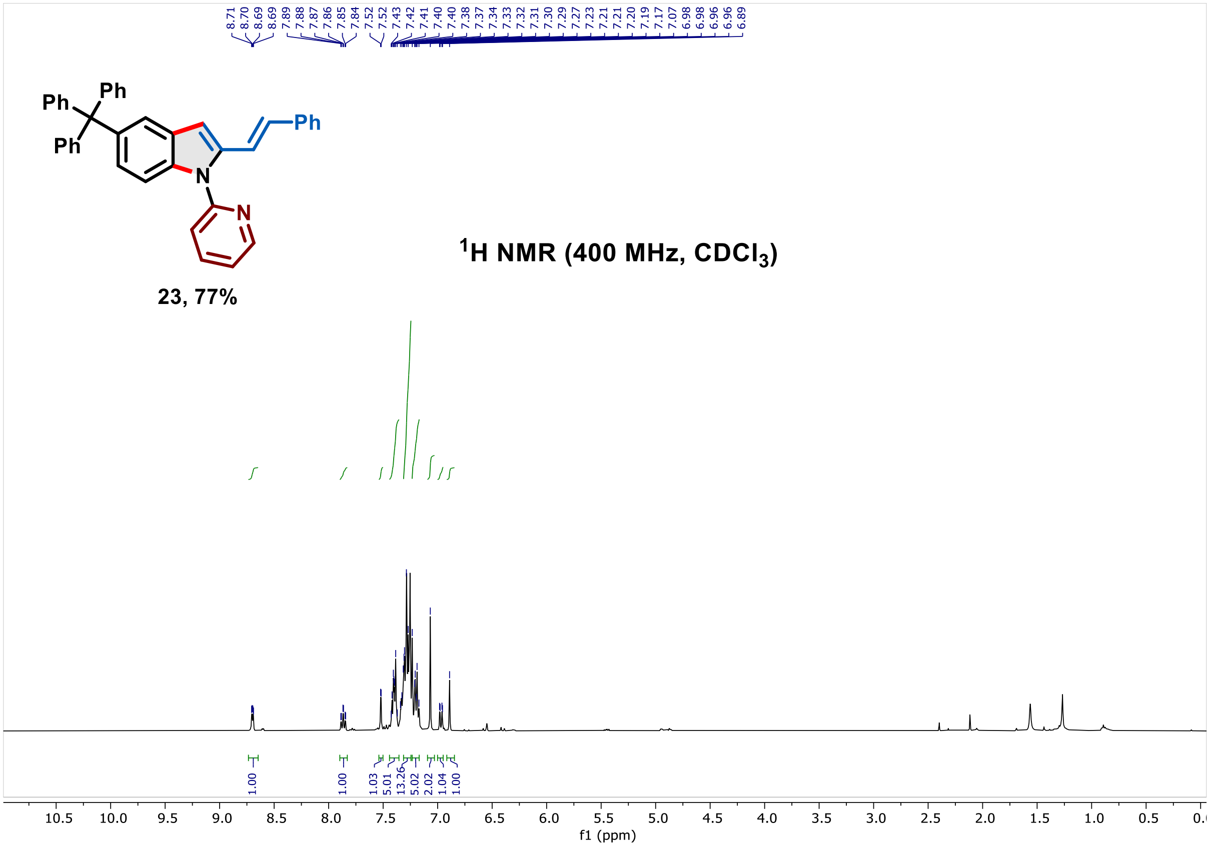
8.71 8.70 8.69 8.69 7.89 7.88 7.87 7.86 7.85 7.84 7.52 7.52 7.43 7.42 7.41 7.40 7.40 7.38 7.37 7.34 7.33 7.32 7.31 7.30 7.29 7.27 7.23 7.21 7.21 7.20 7.19 7.17 7.07 6.98 6.98 6.96 6.96 6.89
Ph
Ph
Ph
Ph
N
N
23, 77%
1H NMR (400 MHz, CDCl3)
1.00 1.00 1.03 5.01 13.26 5.02 2.02 1.04 1.00
f1 (ppm)

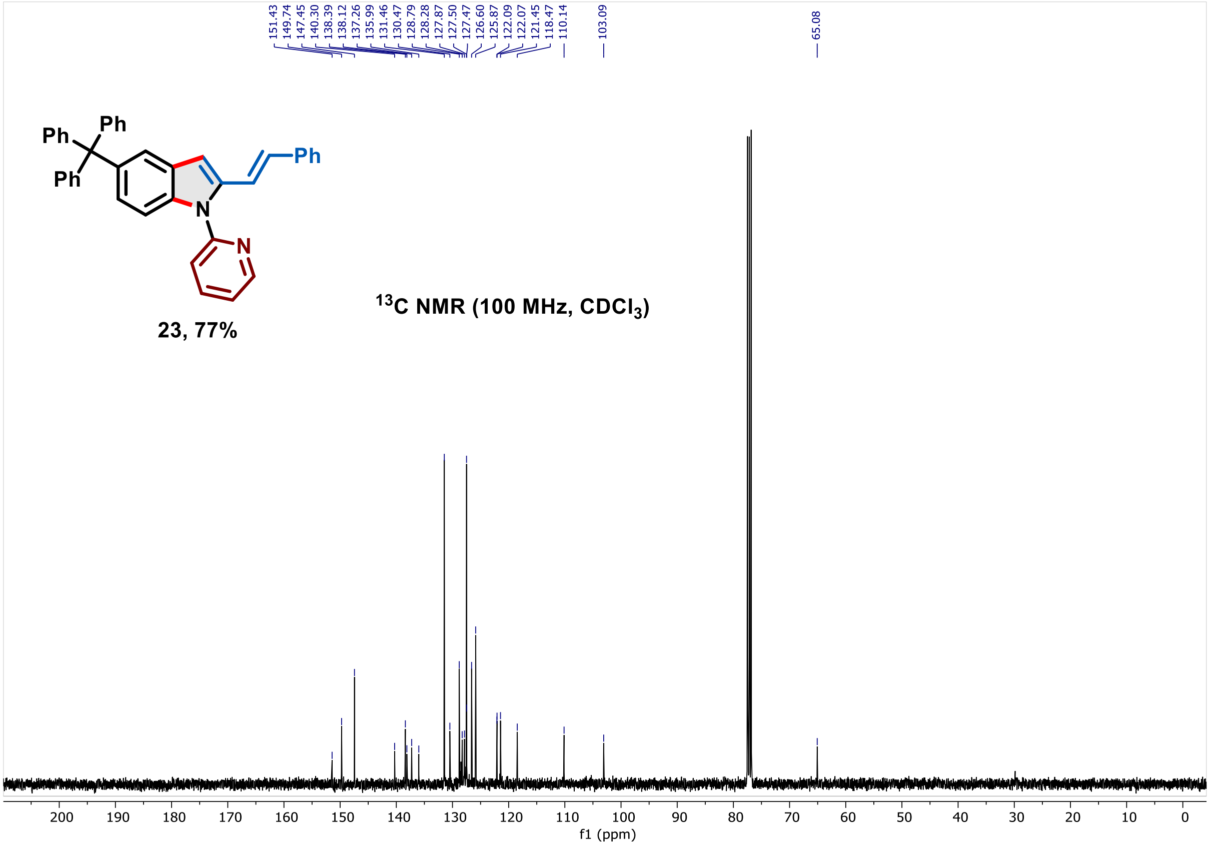
151.43 149.74 147.45 140.30 138.39 138.12 137.26 135.99 131.46 130.47 128.79 128.28 127.87 127.50 127.47 126.60 125.87 122.09 122.07 121.45 118.47 110.14
103.09
65.08
Ph
Ph
Ph
Ph
N
N
23, 77%
^{13}C NMR (100 MHz, CDCl$_3$)
f1 (ppm)

24

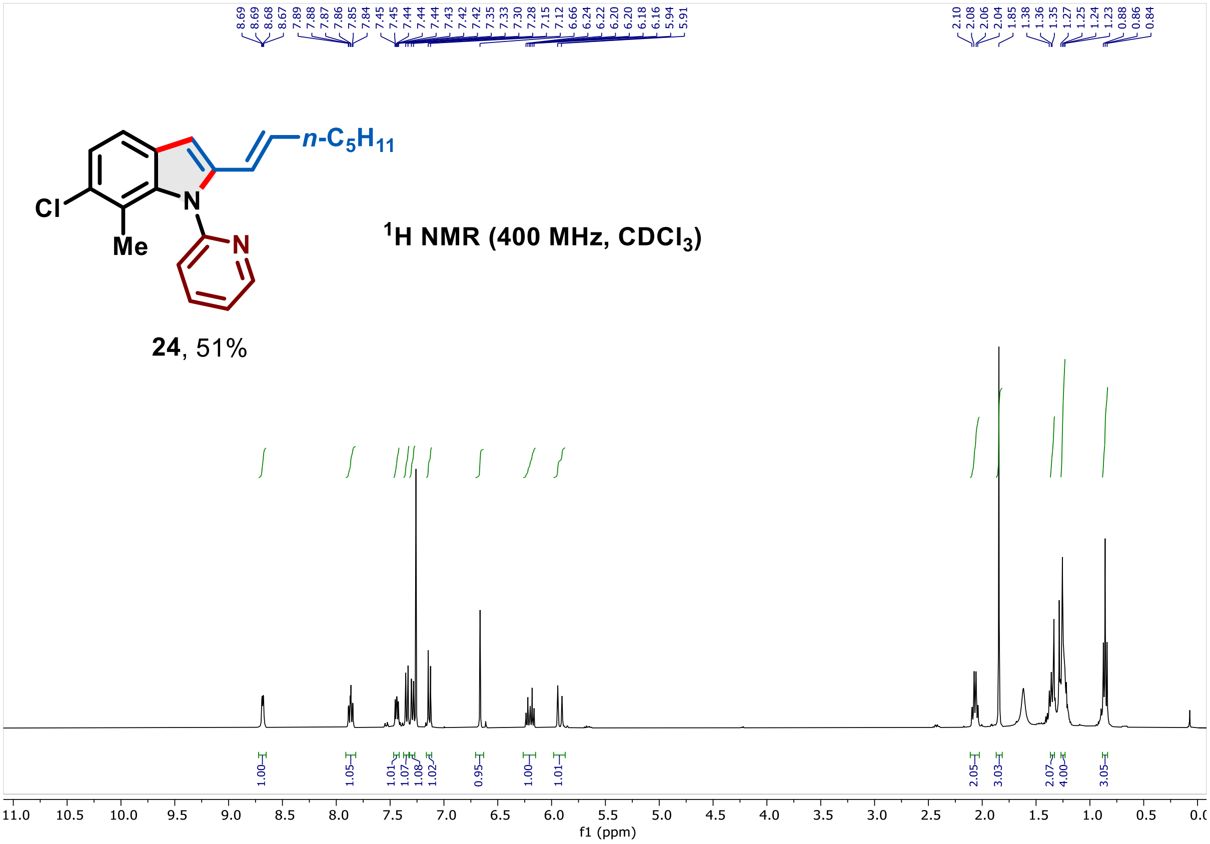
8.69 8.69 8.68 8.67 7.89 7.88 7.87 7.86 7.85 7.84 7.45 7.44 7.44 7.43 7.42 7.42 7.35 7.33 7.30 7.28 7.15 7.12 6.66 6.24 6.22 6.20 6.20 6.18 6.16 5.94 5.91
2.10 2.08 2.06 2.04 1.85 1.38 1.36 1.35 1.27 1.25 1.24 1.23 0.88 0.86 0.84
n-C$_5$H$_{11}$
Cl
Me
N
N
24, 51%
^{1}H NMR (400 MHz, CDCl$_3$)
1.00 1.05 1.01 1.07 1.08 1.02 0.95 1.00 1.01 2.05 3.03 2.07 4.00 3.05
f1 (ppm)

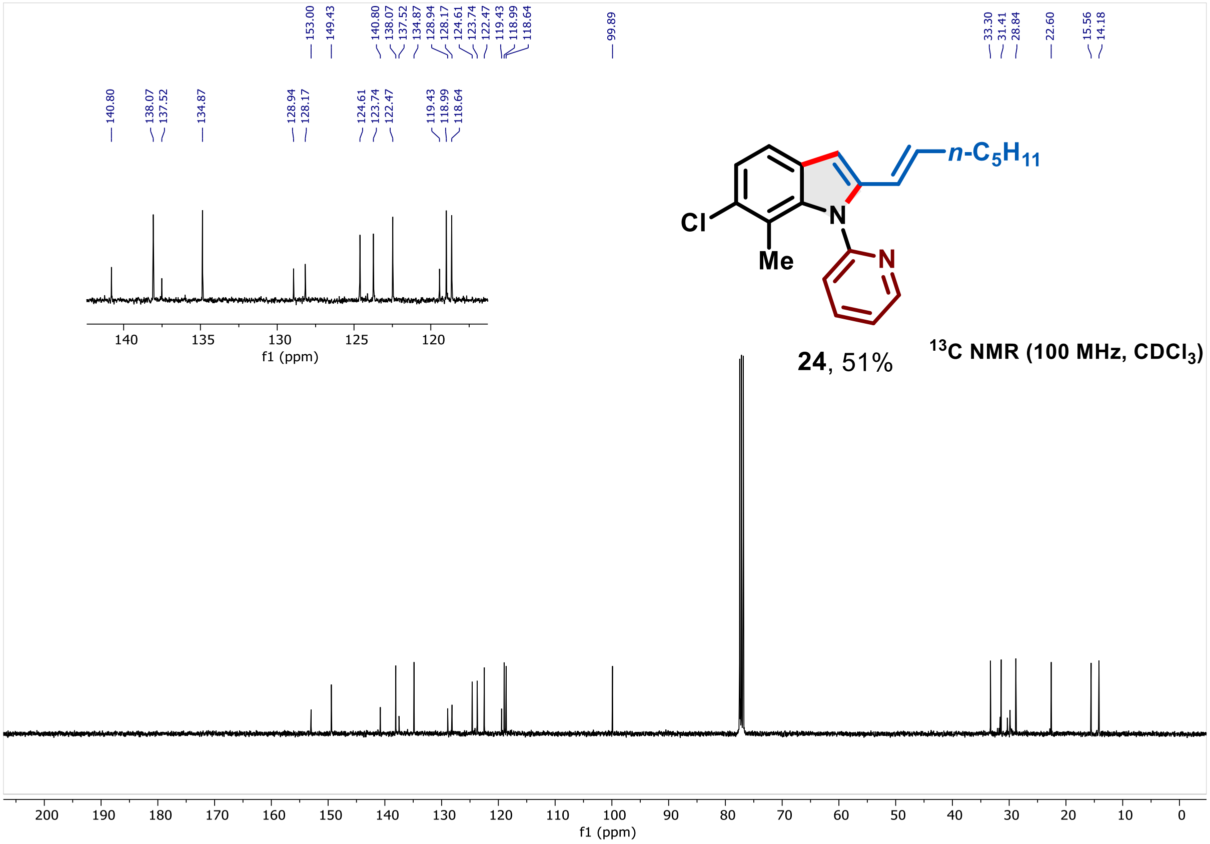
153.00
149.43
140.80
138.07
137.52
134.87
128.94
128.17
124.61
123.74
122.47
119.43
118.99
118.64
99.89
33.30
31.41
28.84
22.60
15.56
14.18
Cl
Me
N
N
n-C5H11
24, 51%
13C NMR (100 MHz, CDCl3)
f1 (ppm)

25

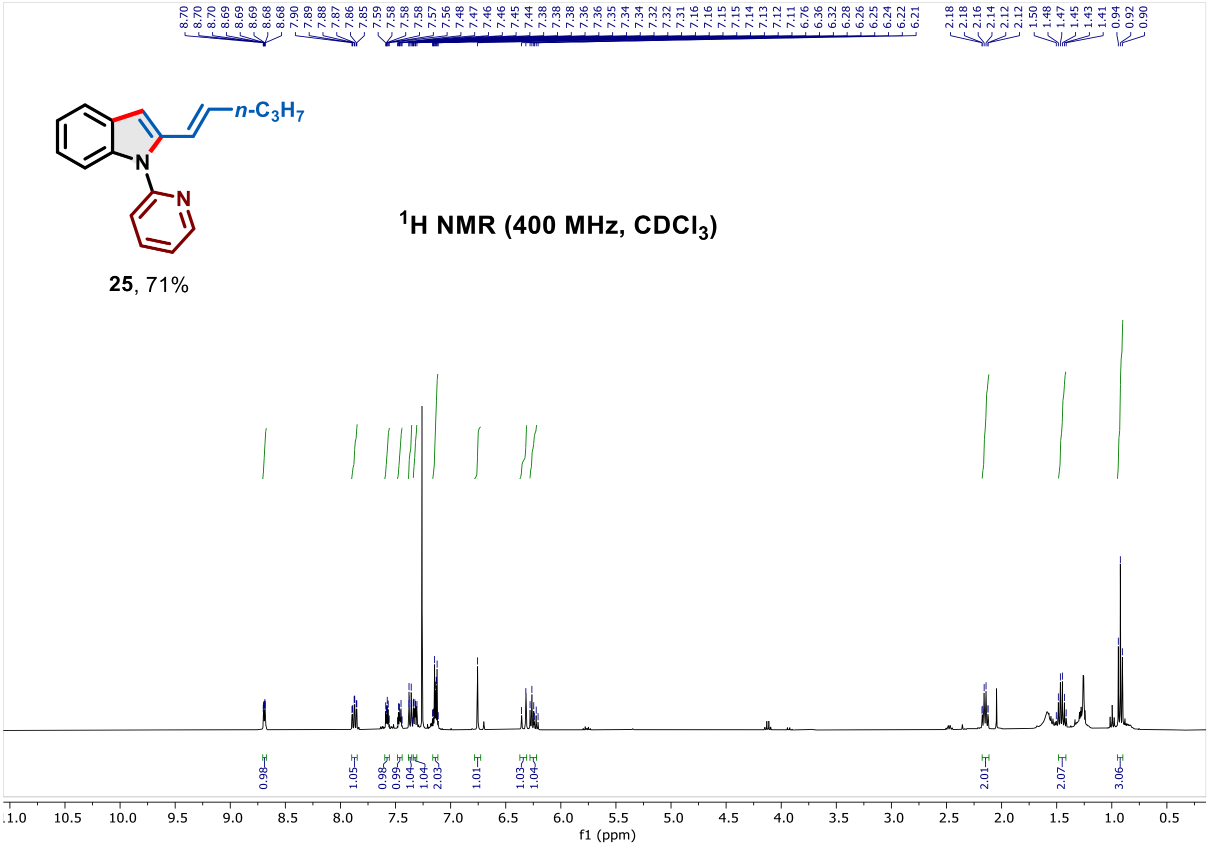
N
N
n-C3H7
25, 71%
1H NMR (400 MHz, CDCl3)
f1 (ppm)

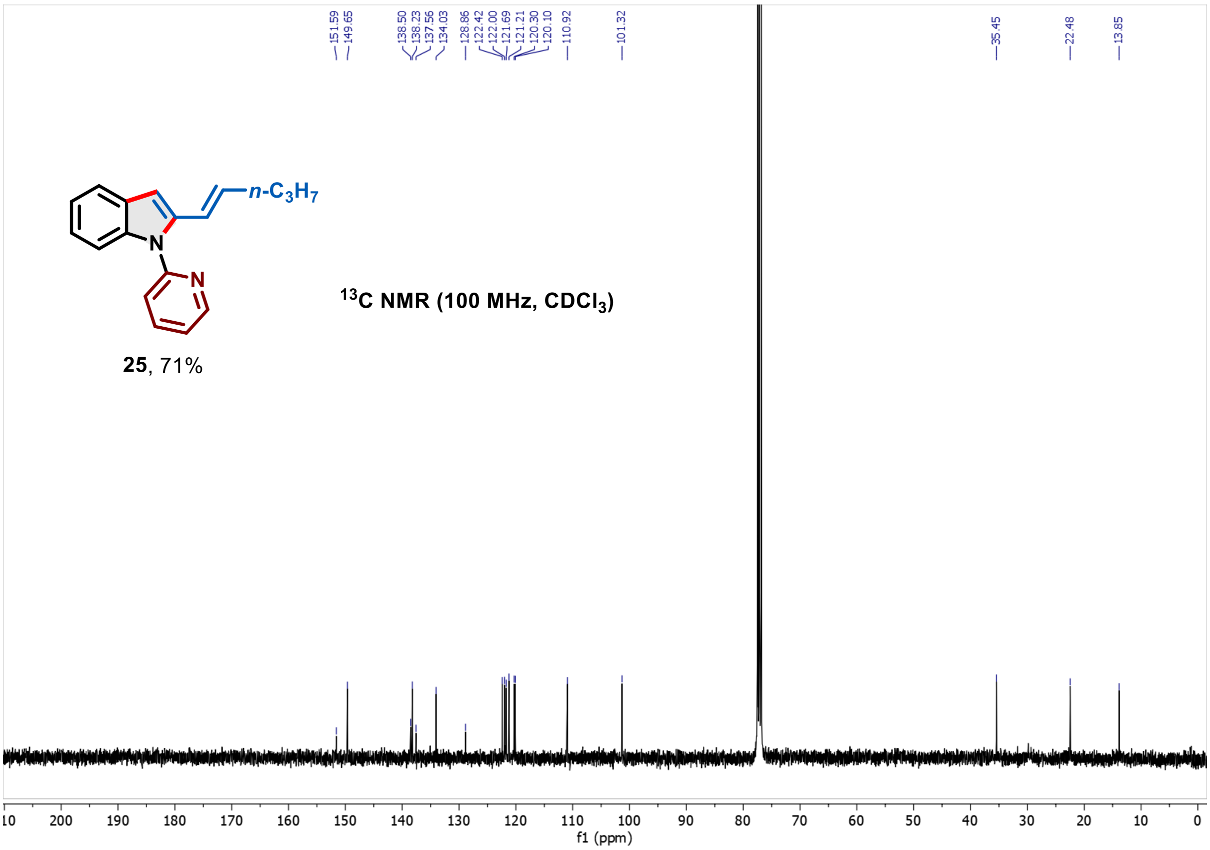
151.59
149.65
138.50
138.23
137.56
134.03
128.86
122.42
122.00
121.69
121.21
120.30
120.10
110.92
101.32
35.45
22.48
13.85
N
N
n-C$_3$H$_7$
25, 71%
^{13}C NMR (100 MHz, CDCl$_3$)
f1 (ppm)

26

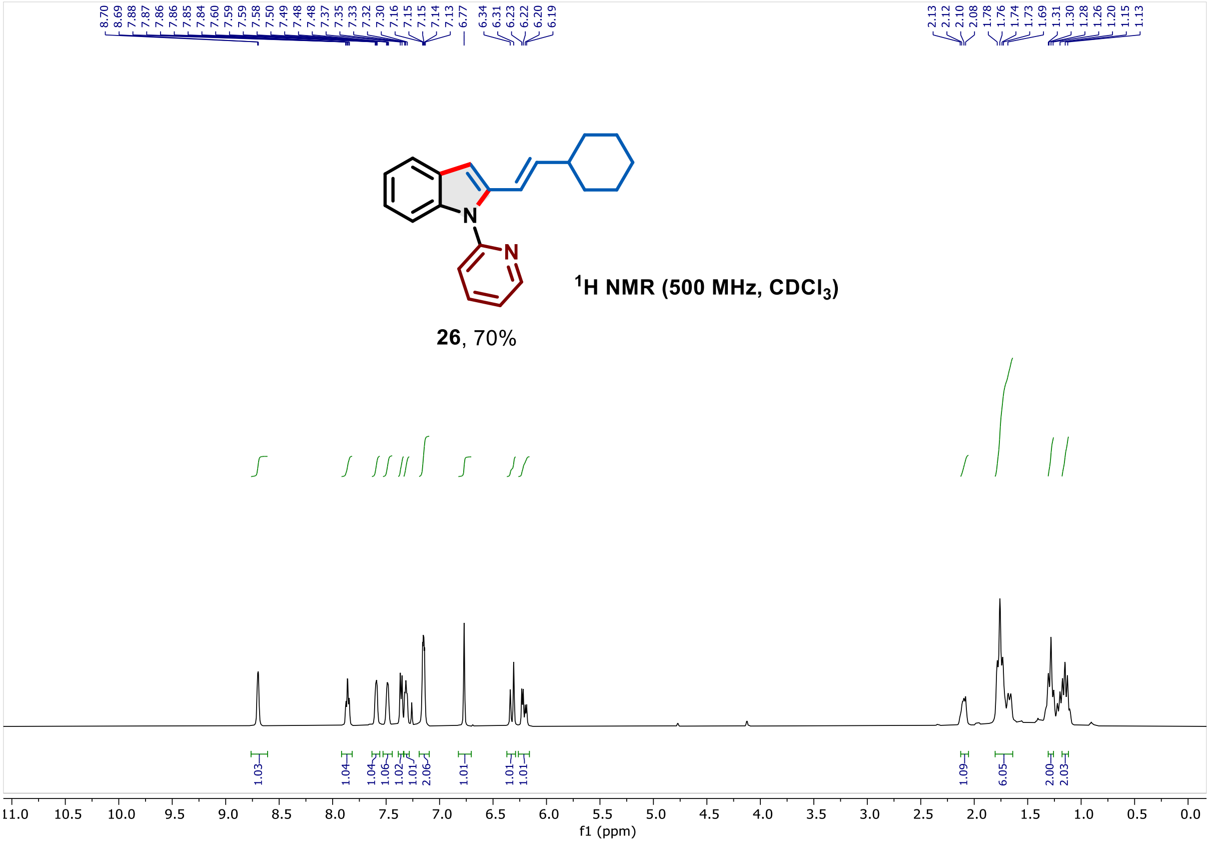
8.70
8.69
7.88
7.87
7.86
7.86
7.85
7.84
7.60
7.59
7.59
7.58
7.50
7.49
7.48
7.48
7.37
7.35
7.33
7.32
7.30
7.16
7.15
7.15
7.14
7.13
6.77
6.34
6.31
6.23
6.22
6.20
6.19
2.13
2.12
2.10
2.08
1.78
1.76
1.74
1.73
1.69
1.31
1.30
1.28
1.26
1.20
1.15
1.13
N
N
^{1}H NMR (500 MHz, CDCl$_3$)
26, 70%
1.03
1.04
1.04
1.06
1.02
1.01
2.06
1.01
1.01
1.01
1.09
6.05
2.00
2.03
f1 (ppm)

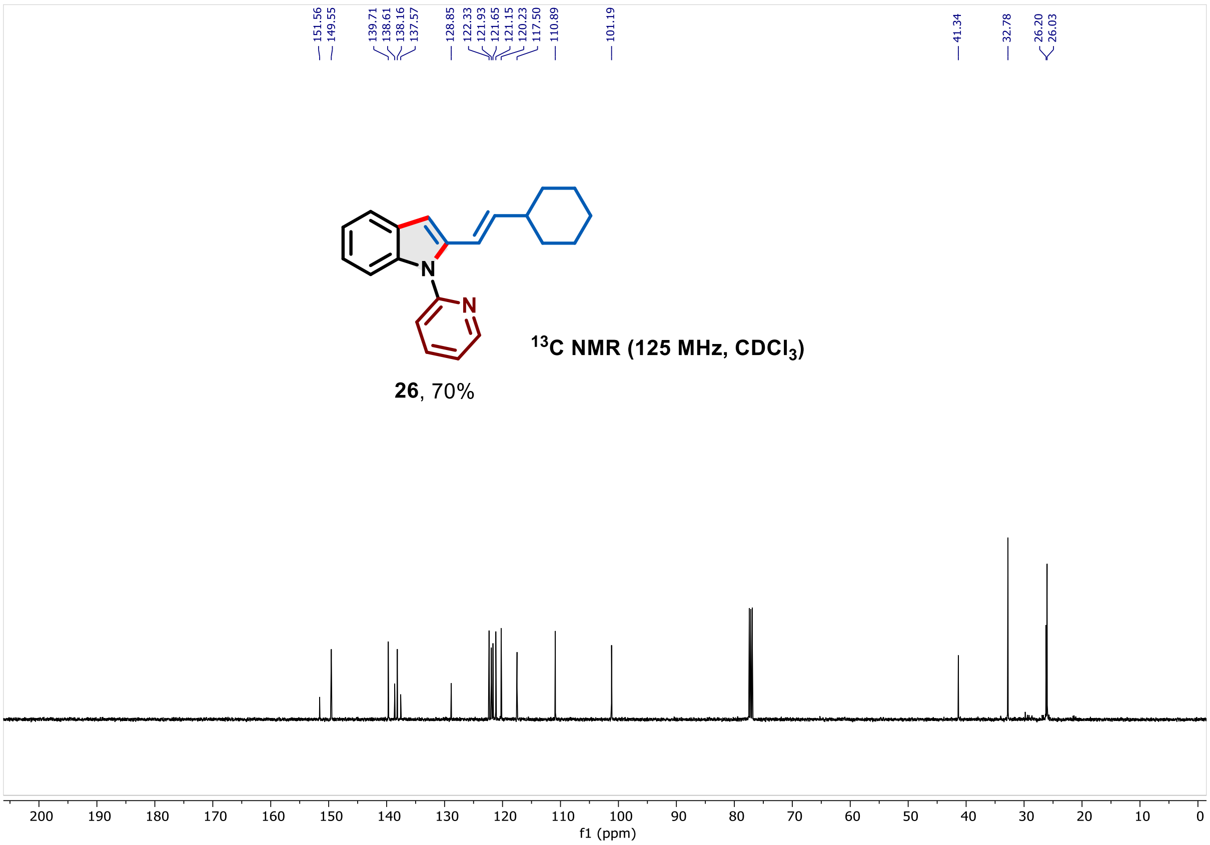
151.56
149.55
139.71
138.61
138.16
137.57
128.85
122.33
121.93
121.65
121.15
120.23
117.50
110.89
101.19
41.34
32.78
26.20
26.03
N
N
13C NMR (125 MHz, CDCl3)
26, 70%
f1 (ppm)

27

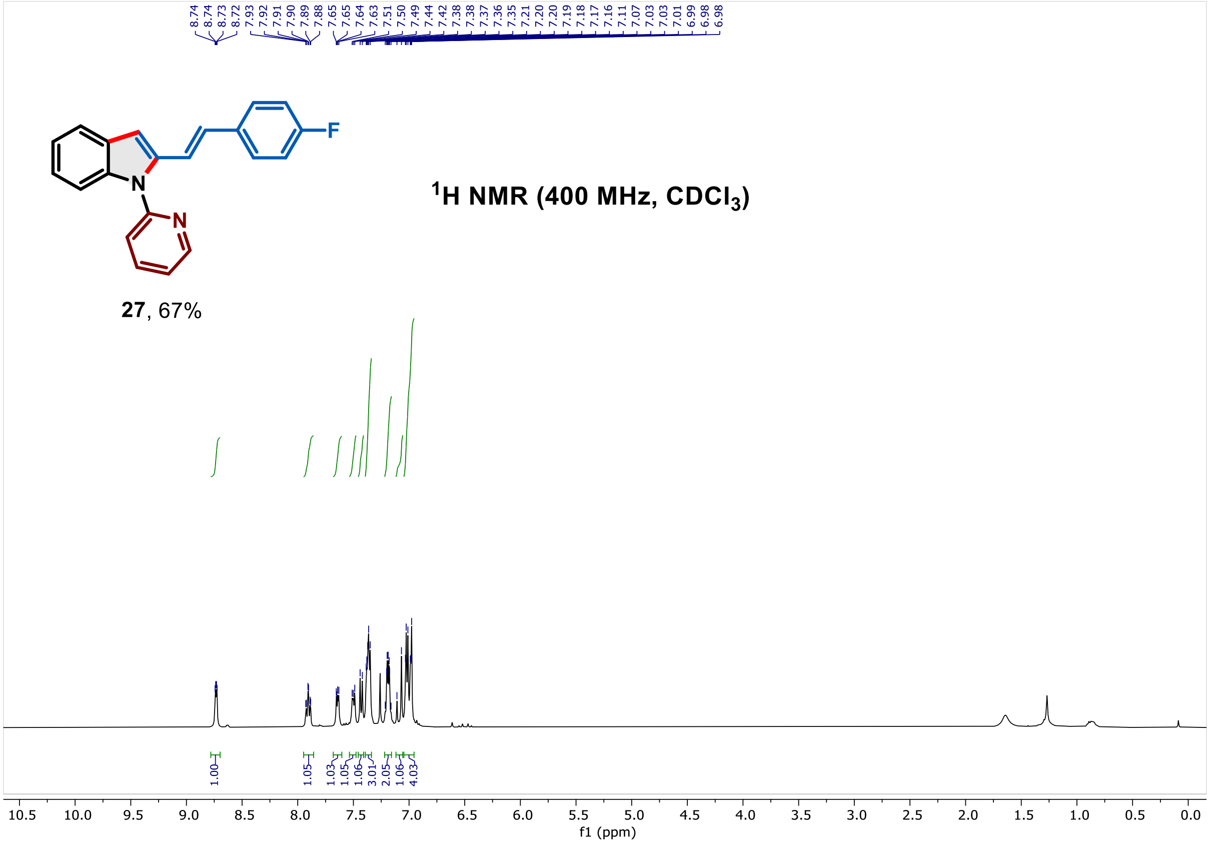
8.74
8.74
8.73
8.72
7.93
7.92
7.91
7.90
7.89
7.88
7.65
7.65
7.64
7.63
7.51
7.50
7.49
7.44
7.42
7.38
7.38
7.37
7.36
7.35
7.21
7.20
7.20
7.19
7.18
7.17
7.16
7.11
7.07
7.03
7.03
7.01
6.99
6.98
6.98
F
N
N
1H NMR (400 MHz, CDCl3)
27, 67%
1.00
1.05
1.03
1.05
1.06
3.01
2.05
1.06
4.03
f1 (ppm)

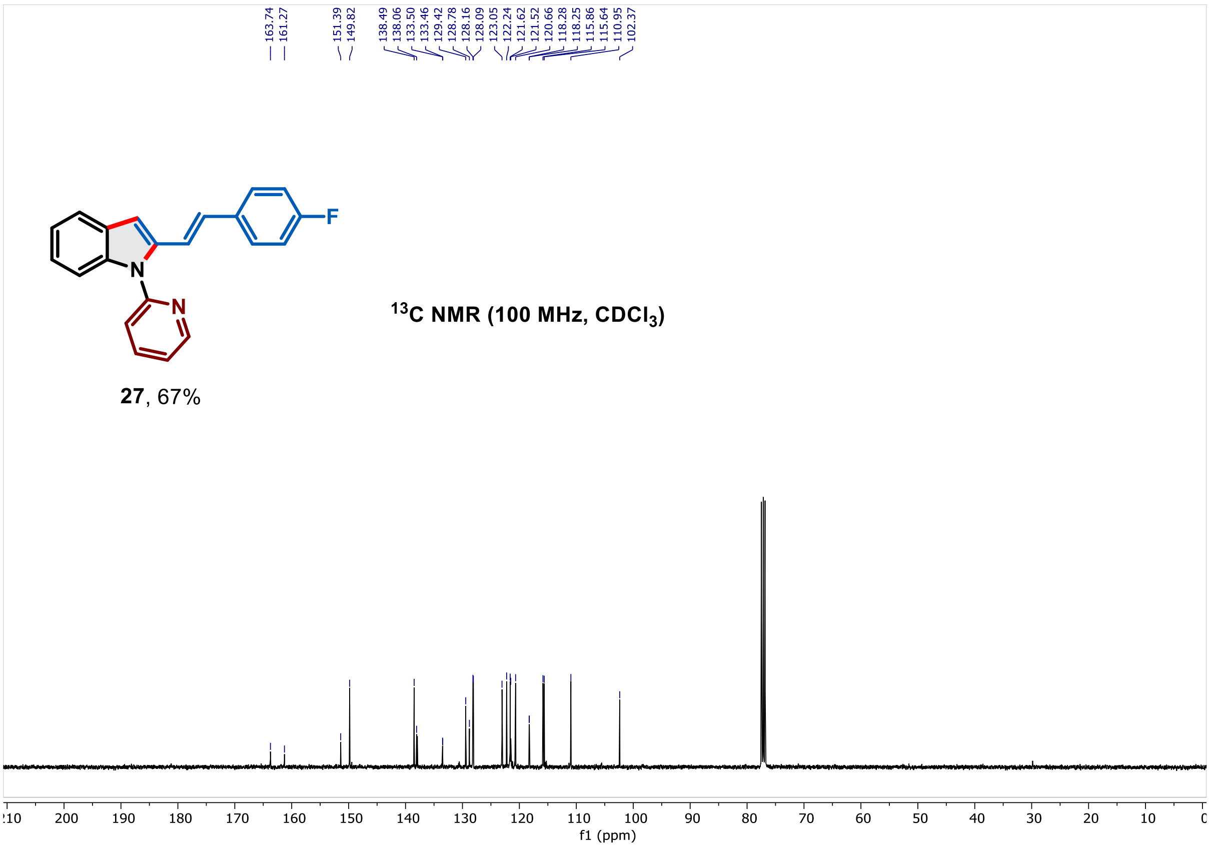
163.74
161.27
151.39
149.82
138.49
138.06
133.50
133.46
129.42
128.78
128.16
128.09
123.05
122.24
121.62
121.52
120.66
118.28
118.25
115.86
115.64
110.95
102.37
F
N
N
27, 67%
^{13}C NMR (100 MHz, CDCl$_3$)
f1 (ppm)

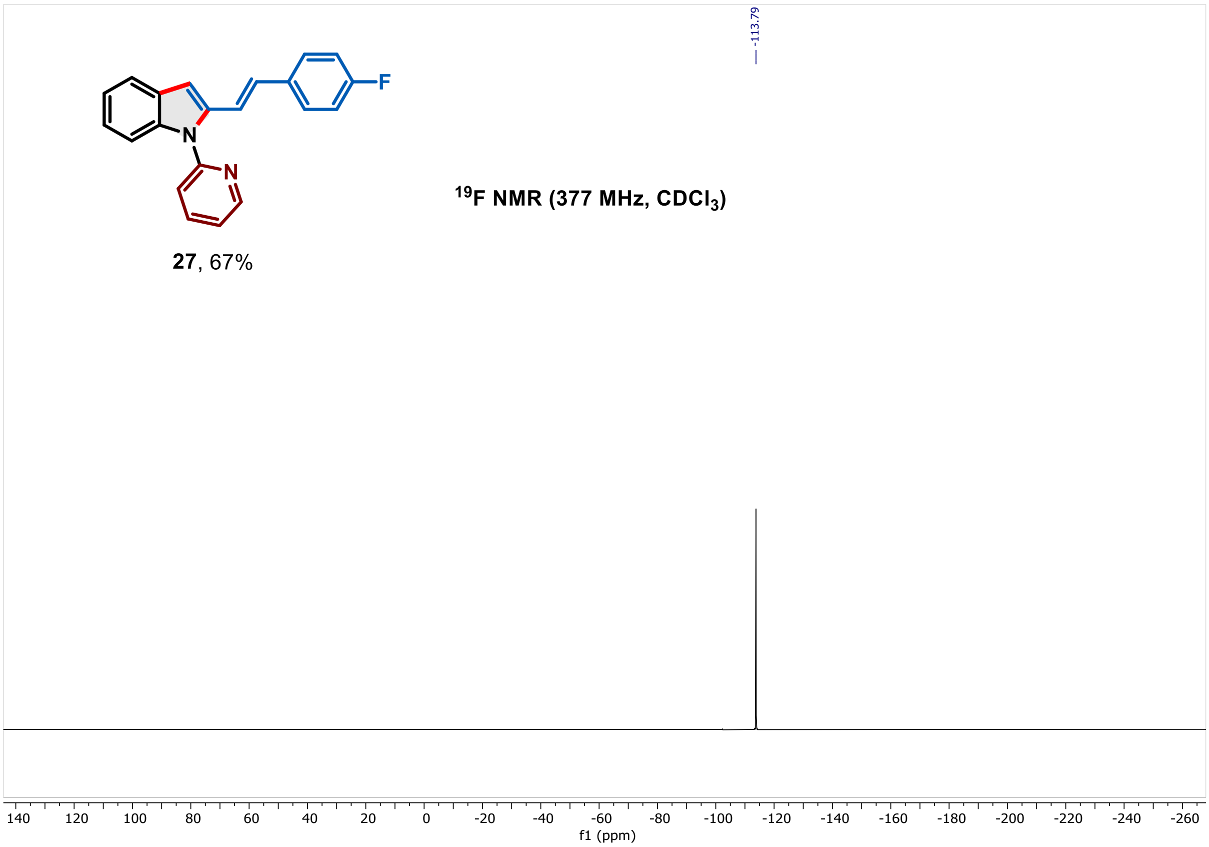
-113.79
F
N
N
27, 67%
^{19}F NMR (377 MHz, CDCl$_3$)
f1 (ppm)

28

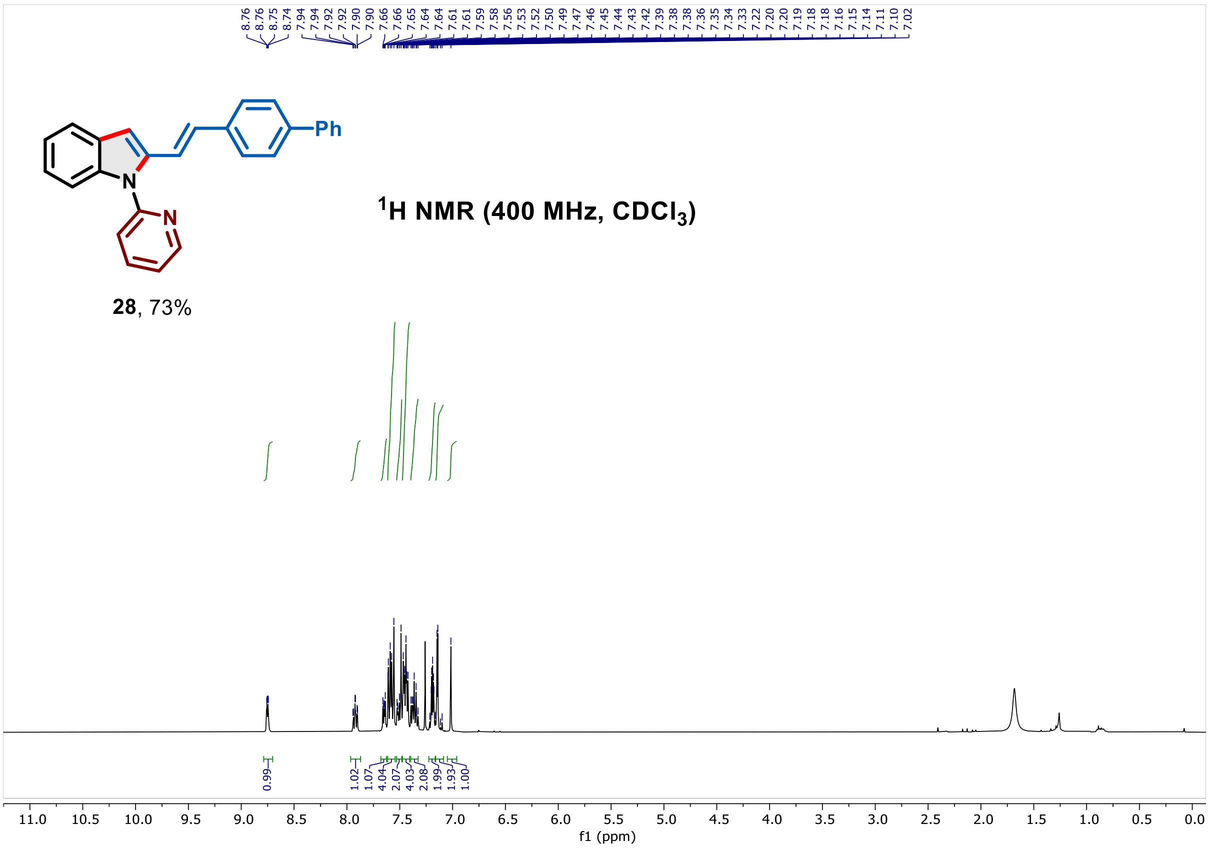
8.76
8.76
8.75
8.74
7.94
7.94
7.92
7.92
7.90
7.90
7.66
7.66
7.65
7.64
7.64
7.61
7.59
7.58
7.56
7.53
7.52
7.50
7.49
7.47
7.46
7.45
7.44
7.43
7.42
7.39
7.38
7.38
7.36
7.35
7.34
7.33
7.22
7.20
7.20
7.19
7.18
7.18
7.16
7.15
7.14
7.11
7.10
7.02
Ph
N
N
28, 73%
1H NMR (400 MHz, CDCl3)
0.99
1.02
1.07
4.04
2.07
4.03
2.08
1.99
1.93
1.00
11.0 10.5 10.0 9.5 9.0 8.5 8.0 7.5 7.0 6.5 6.0 5.5 5.0 4.5 4.0 3.5 3.0 2.5 2.0 1.5 1.0 0.5 0.0
f1 (ppm)

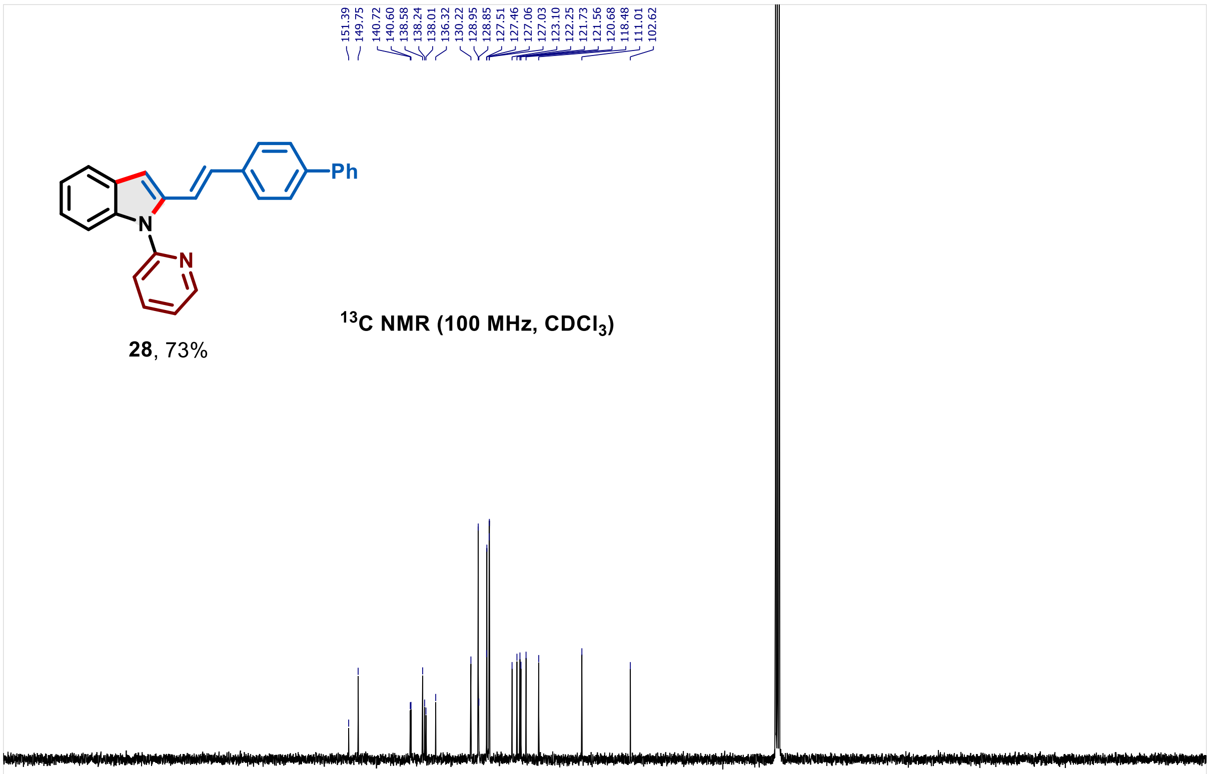
151.39
149.75
140.72
140.60
138.58
138.24
138.01
136.32
130.22
128.95
128.85
127.51
127.46
127.06
127.03
123.10
122.25
121.73
121.56
120.68
118.48
111.01
102.62
Ph
N
N
28, 73%
13C NMR (100 MHz, CDCl3)
210 200 190 180 170 160 150 140 130 120 110 100 90 80 70 60 50 40 30 20 10
f1 (ppm)

29

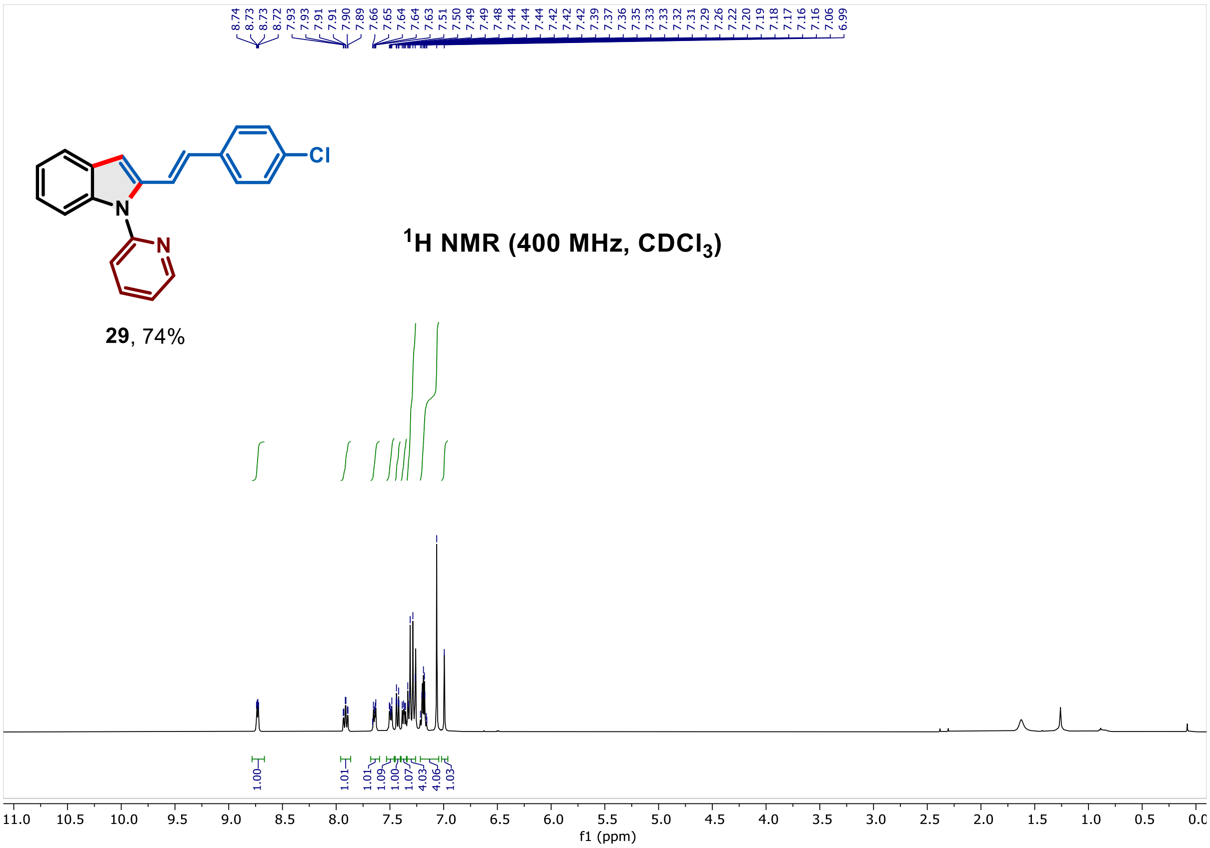
8.74 8.73 8.73 8.72 7.93 7.93 7.91 7.91 7.90 7.89 7.66 7.65 7.64 7.64 7.63 7.51 7.50 7.49 7.49 7.48 7.44 7.44 7.42 7.42 7.42 7.39 7.37 7.36 7.35 7.33 7.33 7.32 7.31 7.29 7.26 7.22 7.20 7.19 7.18 7.17 7.16 7.16 7.06 6.99
Cl
N
N
29, 74%
^{1}H NMR (400 MHz, CDCl$_3$)
1.00 1.01 1.01 1.09 1.00 1.07 4.03 4.06 1.03
11.0 10.5 10.0 9.5 9.0 8.5 8.0 7.5 7.0 6.5 6.0 5.5 5.0 4.5 4.0 3.5 3.0 2.5 2.0 1.5 1.0 0.5 0.0
f1 (ppm)

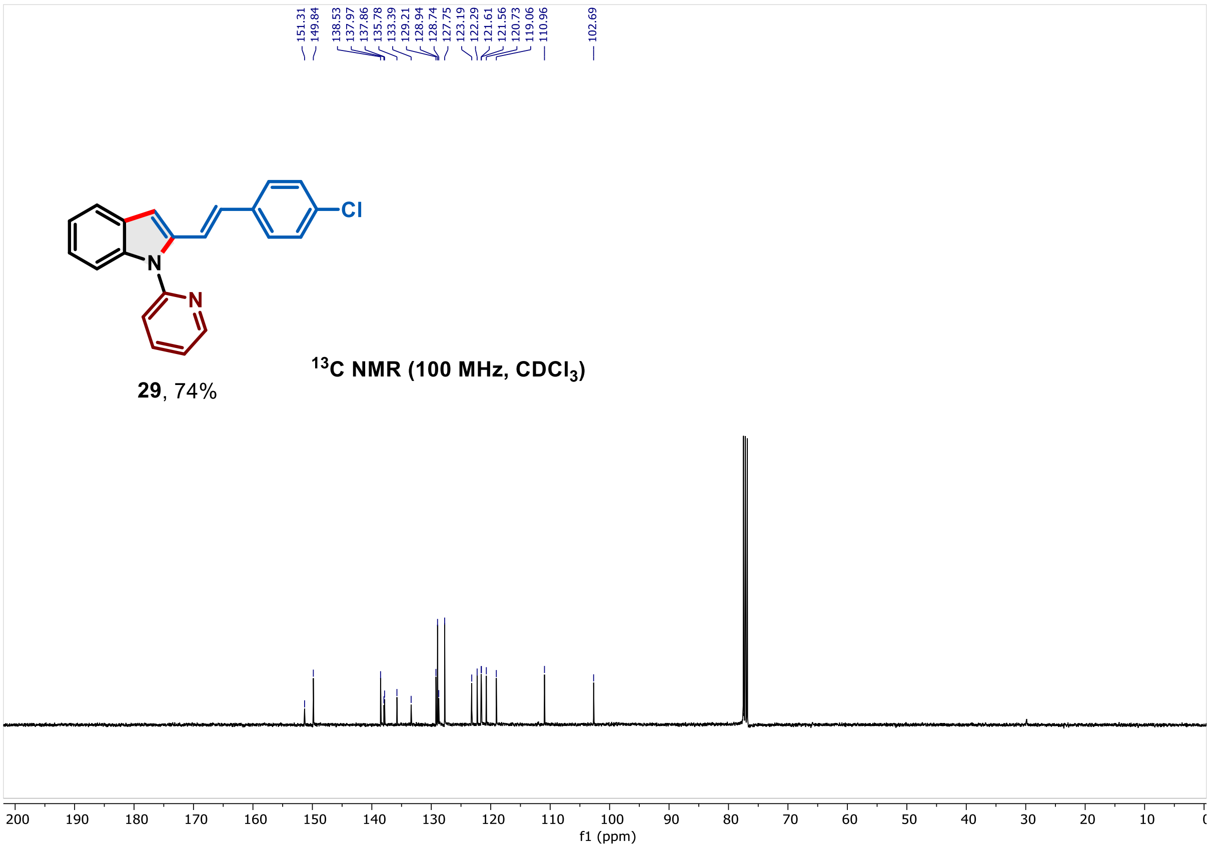
151.31 149.84 138.53 137.97 137.86 135.78 133.39 129.21 128.94 128.74 127.75 123.19 122.29 121.61 121.56 120.73 119.06 110.96 102.69
Cl
N
N
29, 74%
^{13}C NMR (100 MHz, CDCl$_3$)
200 190 180 170 160 150 140 130 120 110 100 90 80 70 60 50 40 30 20 10 0
f1 (ppm)

30

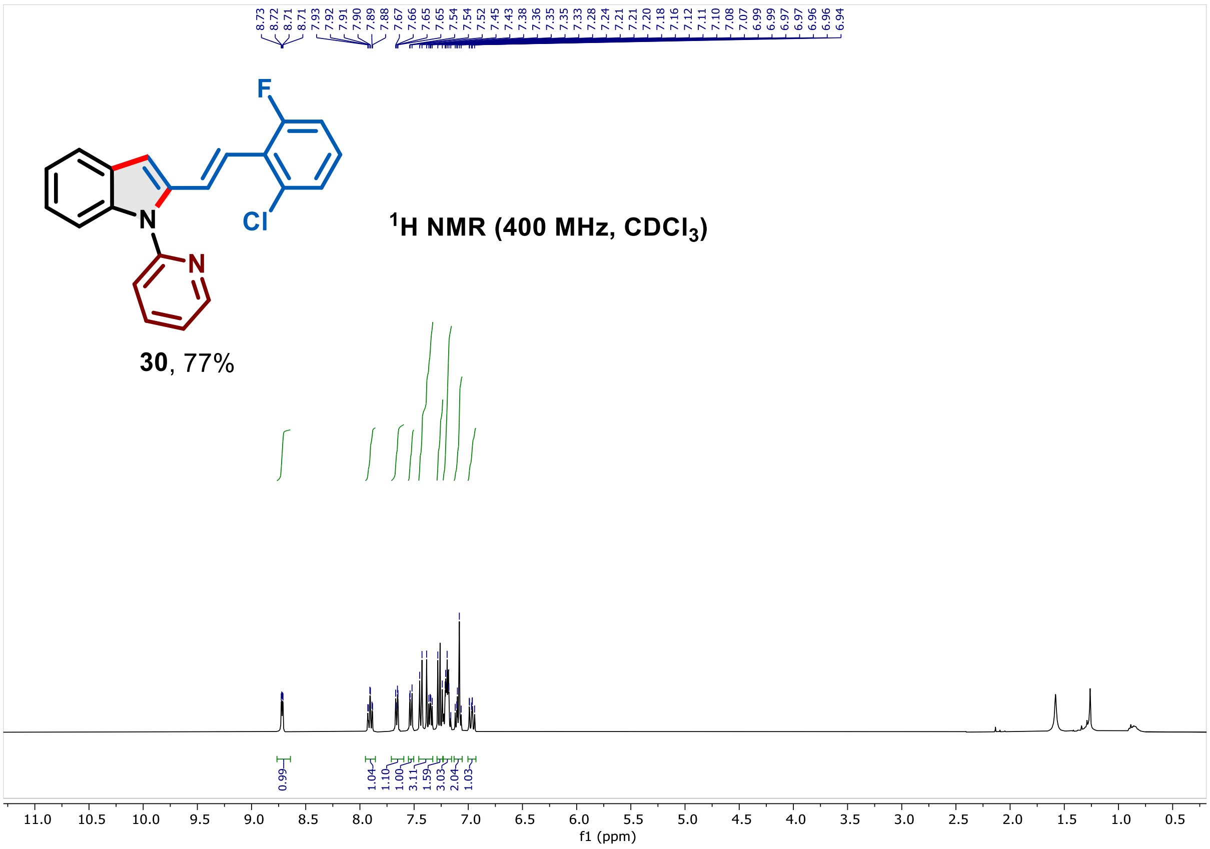

8.73 8.72 8.71 8.71 7.93 7.92 7.91 7.90 7.89 7.88 7.67 7.66 7.65 7.65 7.54 7.54 7.52 7.45 7.43 7.38 7.36 7.35 7.35 7.33 7.28 7.24 7.21 7.21 7.20 7.18 7.16 7.12 7.11 7.10 7.08 7.07 6.99 6.99 6.97 6.97 6.96 6.96 6.94
F
Cl
N
N
30, 77%
^{1}H NMR (400 MHz, CDCl$_3$)
0.99 1.04 1.10 1.00 3.11 1.59 3.03 2.04 1.03
11.0 10.5 10.0 9.5 9.0 8.5 8.0 7.5 7.0 6.5 6.0 5.5 5.0 4.5 4.0 3.5 3.0 2.5 2.0 1.5 1.0 0.5
f1 (ppm)

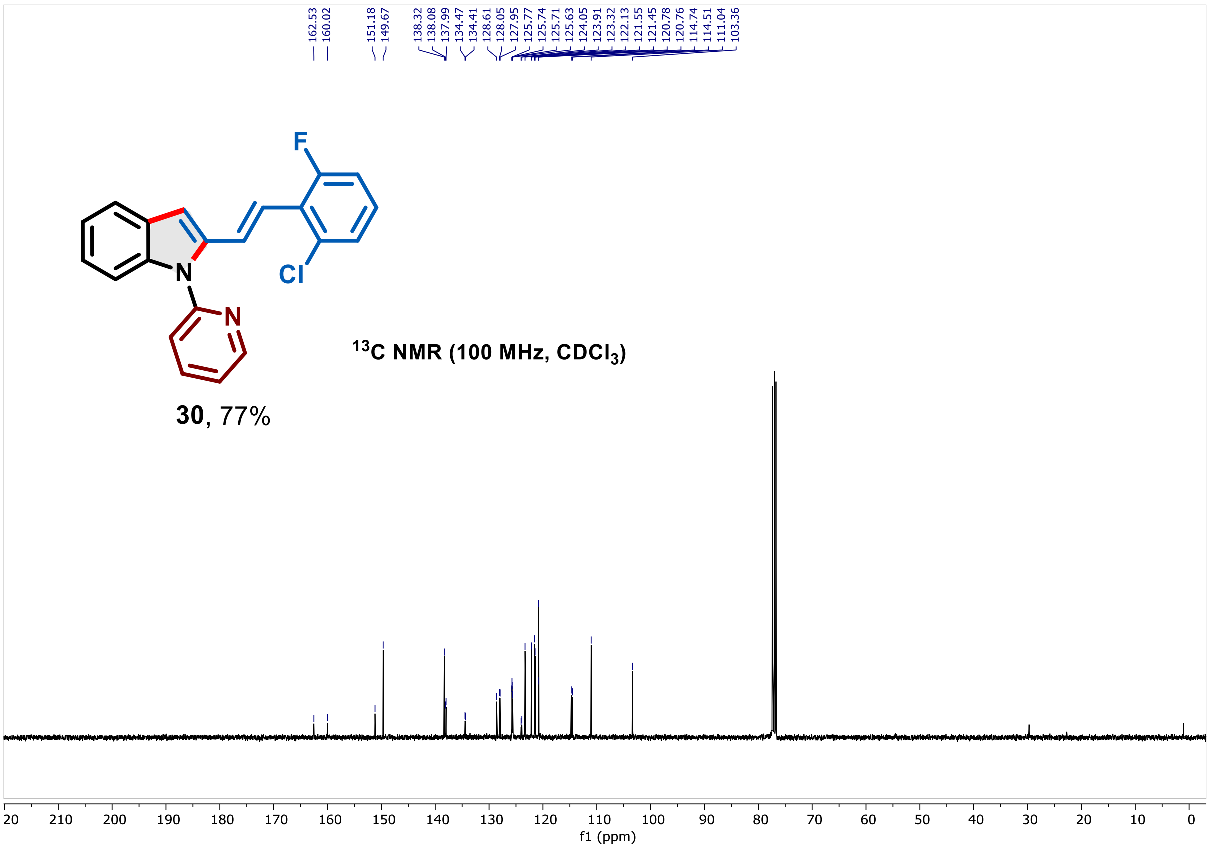

162.53 160.02 151.18 149.67 138.32 138.08 137.99 134.47 134.41 128.61 128.05 127.95 125.77 125.74 125.71 125.63 124.05 123.91 123.32 122.13 121.55 121.45 120.78 120.76 114.74 114.51 111.04 103.36
F
Cl
N
N
30, 77%
^{13}C NMR (100 MHz, CDCl$_3$)
20 210 200 190 180 170 160 150 140 130 120 110 100 90 80 70 60 50 40 30 20 10 0
f1 (ppm)

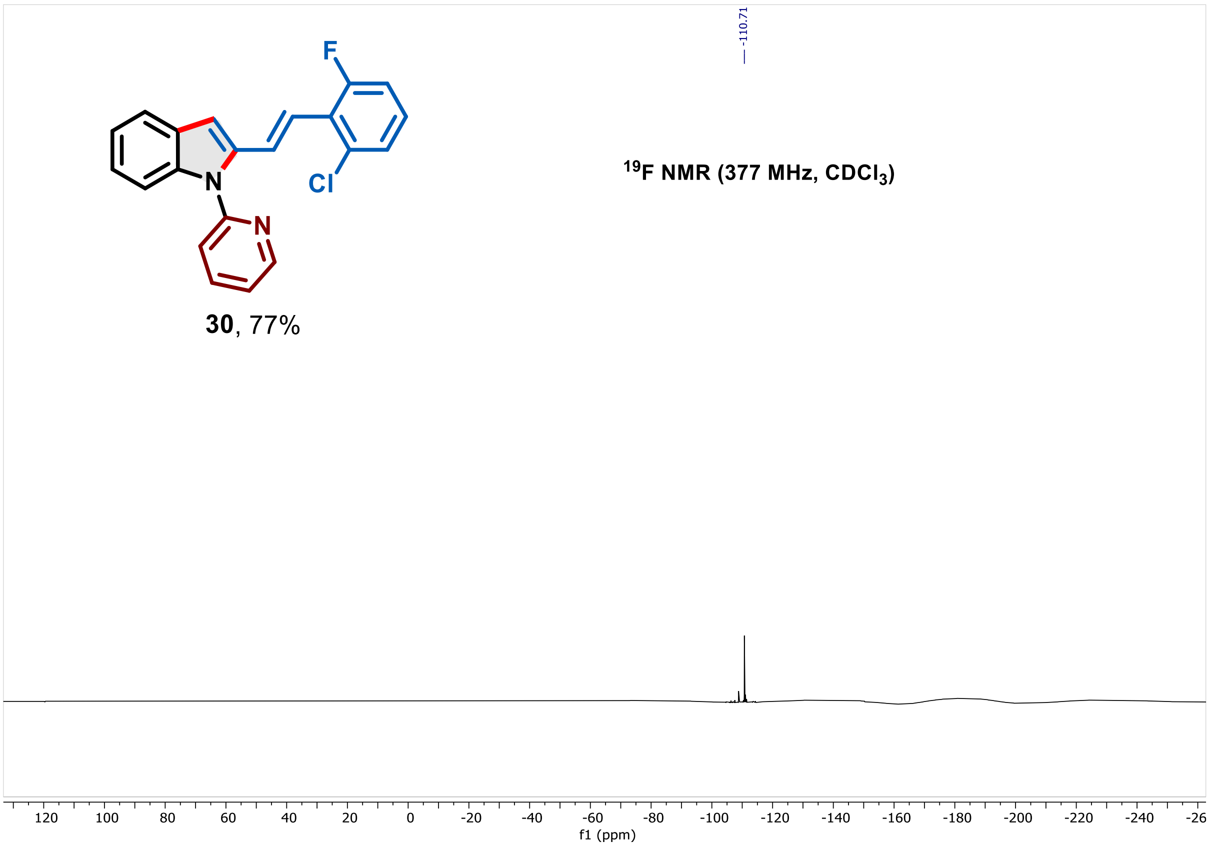

-110.71
F
Cl
N
N
30, 77%
19F NMR (377 MHz, CDCl3)
f1 (ppm)

31

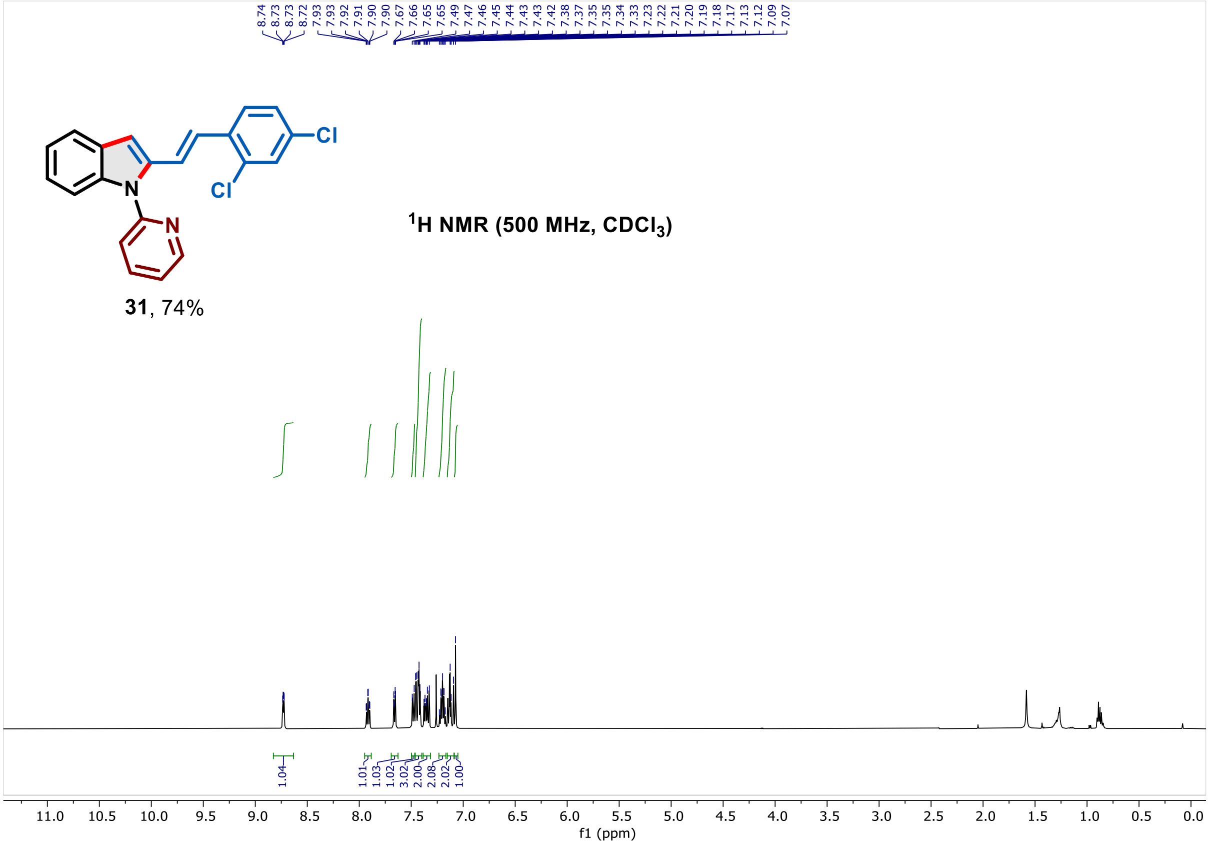

8.74
8.73
8.73
8.72
7.93
7.93
7.92
7.91
7.90
7.90
7.67
7.66
7.65
7.65
7.49
7.47
7.46
7.45
7.44
7.43
7.43
7.42
7.38
7.37
7.35
7.35
7.34
7.33
7.23
7.22
7.21
7.20
7.19
7.18
7.17
7.13
7.12
7.09
7.07
Cl
Cl
N
N
31, 74%
1H NMR (500 MHz, CDCl3)
1.04
1.01
1.03
1.02
3.02
2.00
2.08
2.02
1.00
f1 (ppm)

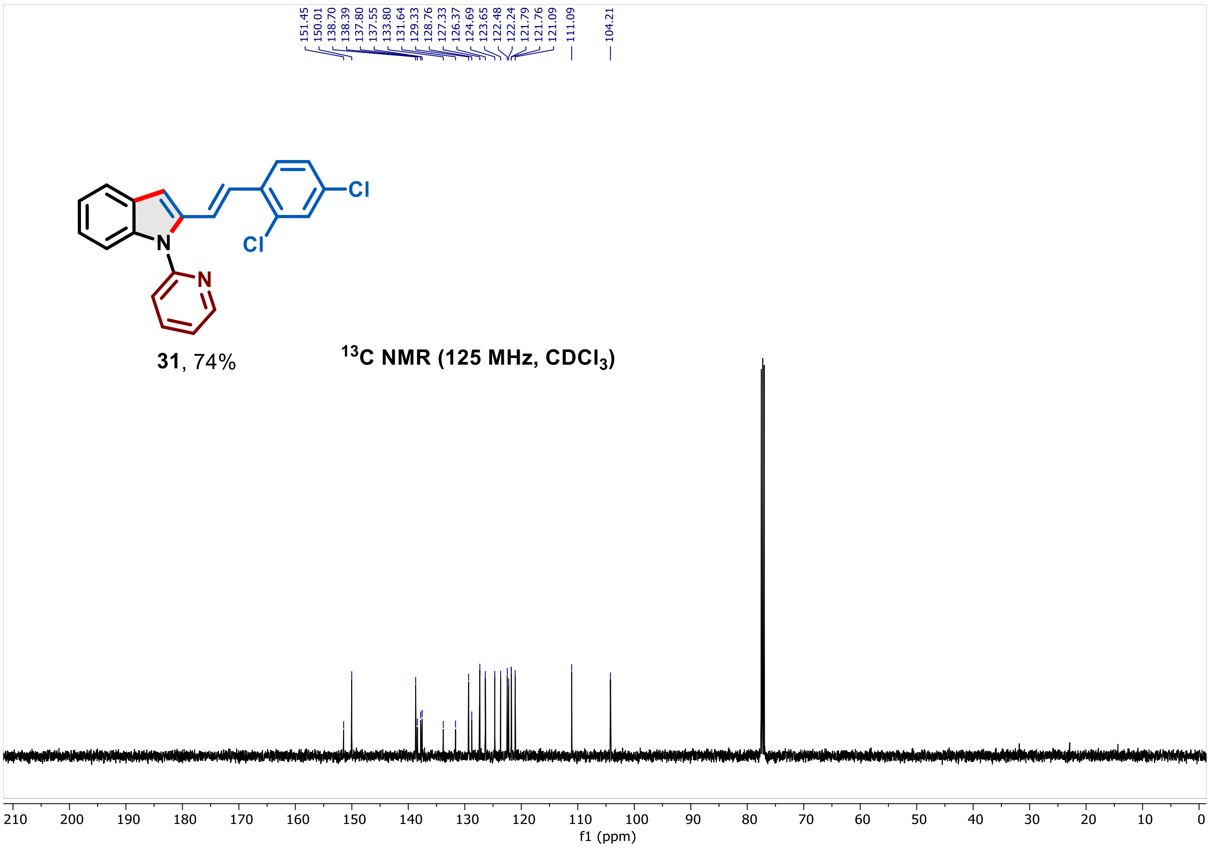
151.45
150.01
138.70
138.39
137.80
137.55
133.80
131.64
129.33
128.76
127.33
126.37
124.69
123.65
122.48
122.24
121.79
121.76
121.09
111.09
104.21
Cl
Cl
N
N
31, 74%
13C NMR (125 MHz, CDCl3)
f1 (ppm)

32

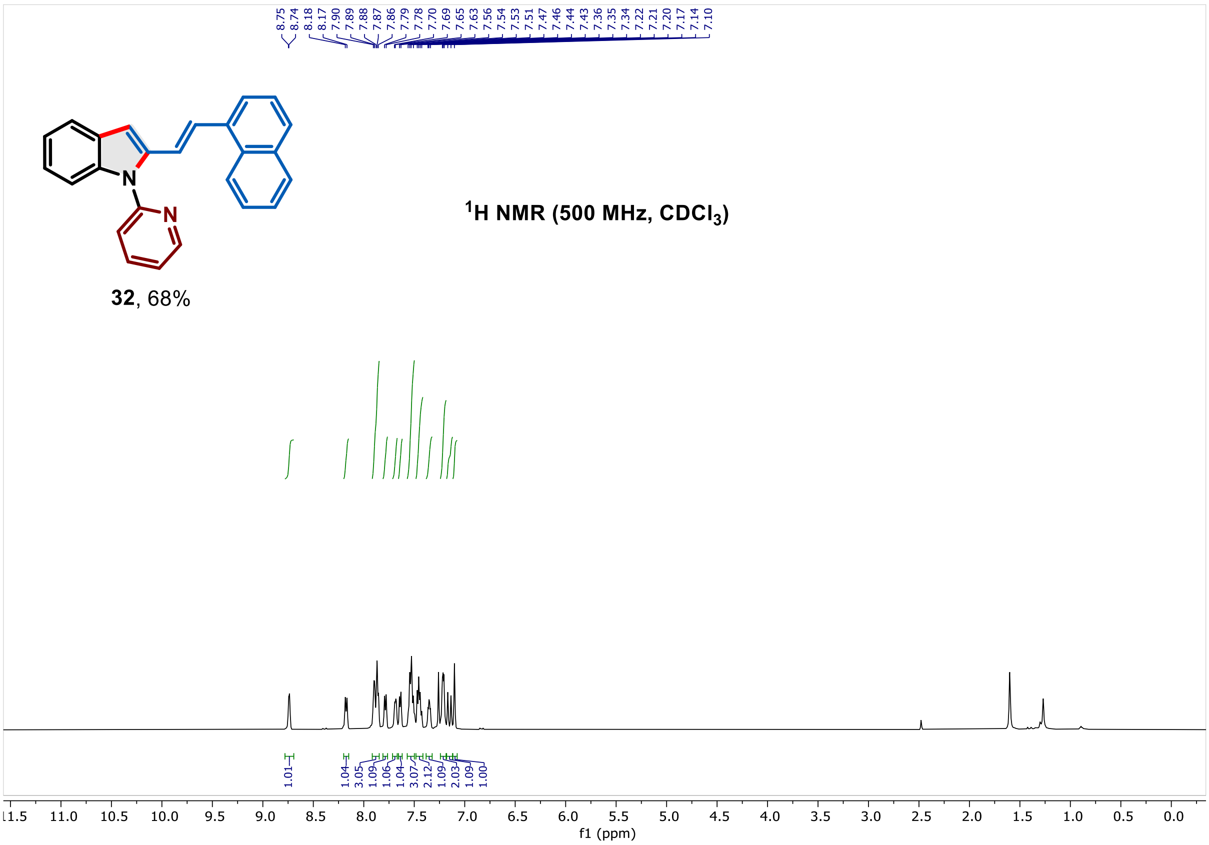
8.75
8.74
8.18
8.17
7.90
7.89
7.88
7.87
7.86
7.79
7.78
7.70
7.69
7.65
7.63
7.56
7.54
7.53
7.51
7.47
7.46
7.44
7.43
7.36
7.35
7.34
7.22
7.21
7.20
7.17
7.14
7.10
N
N
32, 68%
1H NMR (500 MHz, CDCl3)
1.01
1.04
3.05
1.09
1.06
1.04
3.07
2.12
1.09
2.03
1.09
1.00
f1 (ppm)

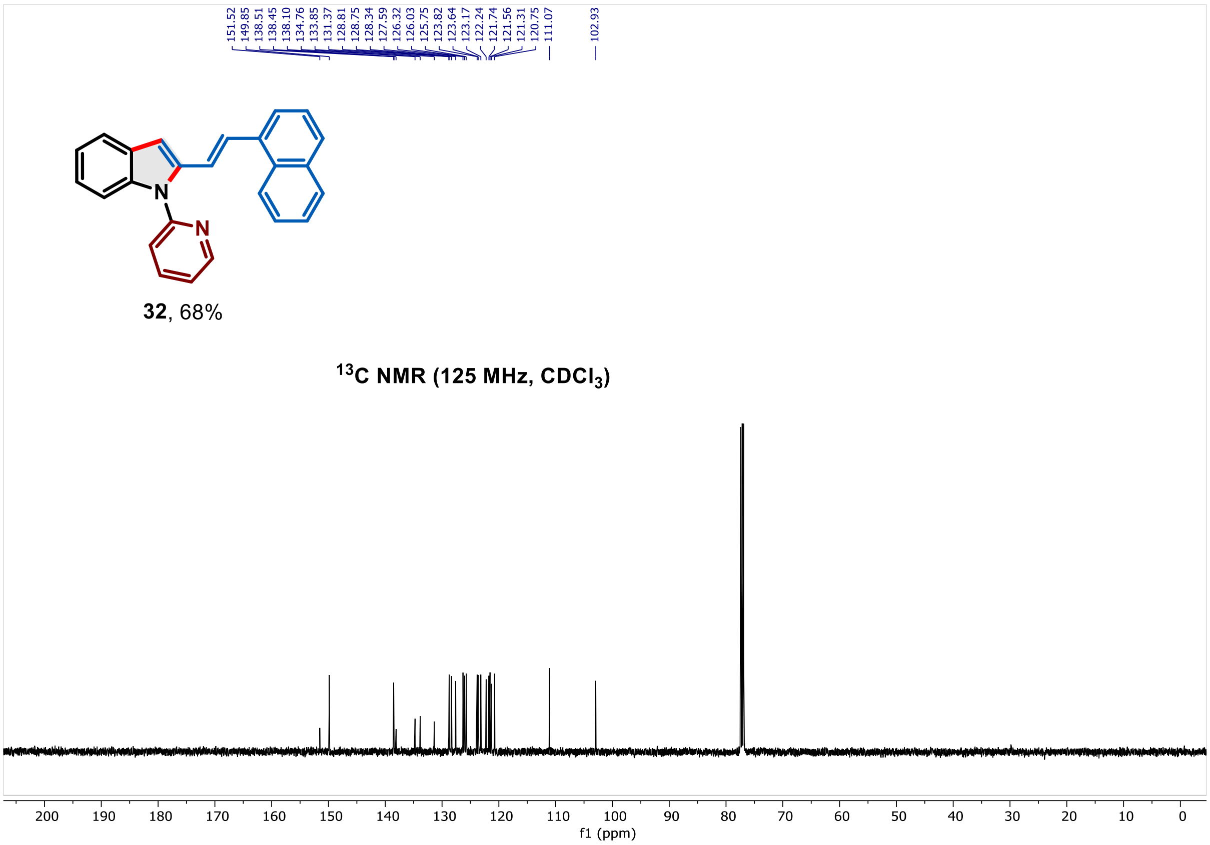
151.52
149.85
138.51
138.45
138.10
134.76
133.85
131.37
128.81
128.75
128.34
127.59
126.32
126.03
125.75
123.82
123.64
123.17
122.24
121.74
121.56
121.31
120.75
111.07
102.93
N
N
32, 68%
13C NMR (125 MHz, CDCl3)
200 190 180 170 160 150 140 130 120 110 100 90 80 70 60 50 40 30 20 10 0
f1 (ppm)

33

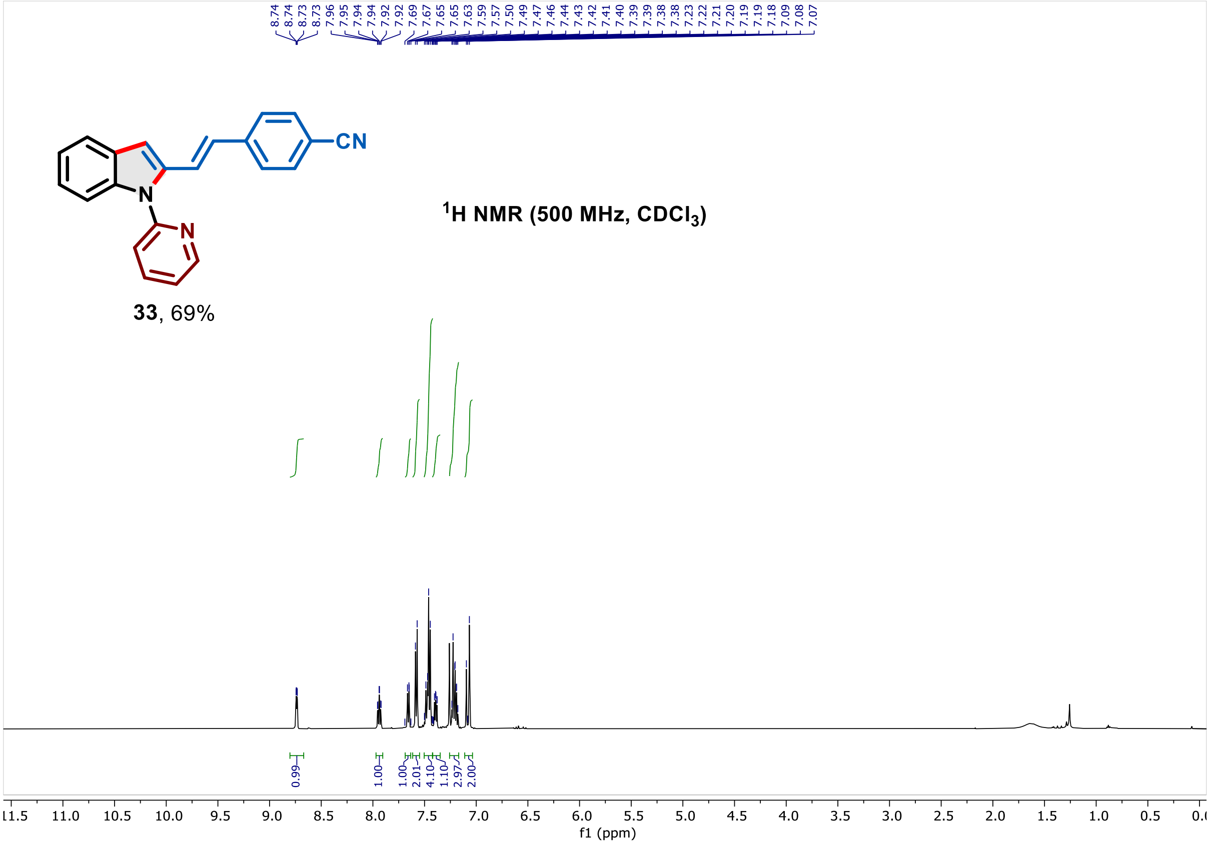
8.74
8.74
8.73
8.73
7.96
7.95
7.94
7.94
7.92
7.92
7.69
7.67
7.65
7.65
7.63
7.59
7.57
7.50
7.49
7.47
7.46
7.44
7.43
7.42
7.41
7.40
7.39
7.39
7.38
7.38
7.23
7.22
7.21
7.20
7.19
7.19
7.18
7.09
7.08
7.07
CN
N
N
33, 69%
1H NMR (500 MHz, CDCl3)
0.99
1.00
1.00
2.01
4.10
1.10
2.97
2.00
11.5 11.0 10.5 10.0 9.5 9.0 8.5 8.0 7.5 7.0 6.5 6.0 5.5 5.0 4.5 4.0 3.5 3.0 2.5 2.0 1.5 1.0 0.5 0.0
f1 (ppm)

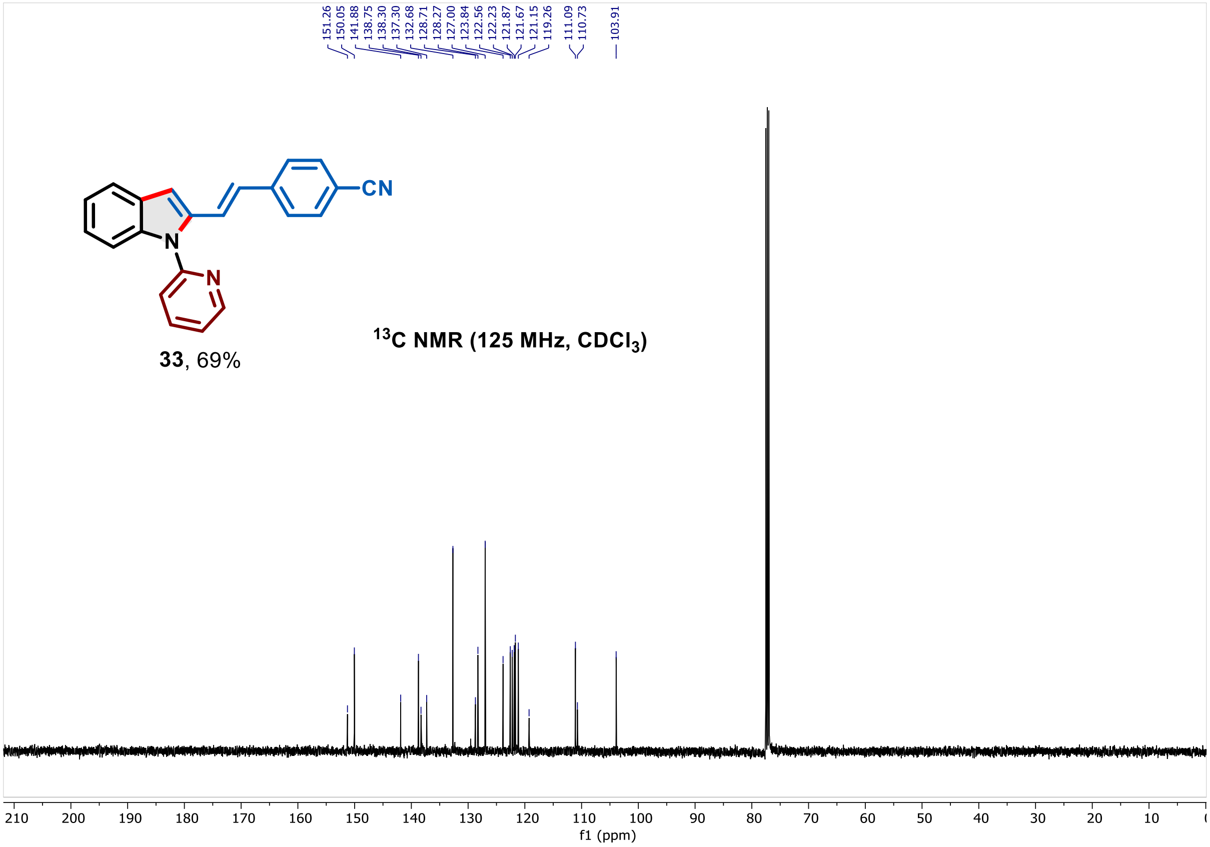
151.26
150.05
141.88
138.75
138.30
137.30
132.68
128.71
128.27
127.00
123.84
122.56
122.23
121.87
121.67
121.15
119.26
111.09
110.73
103.91
CN
N
N
33, 69%
13C NMR (125 MHz, CDCl3)
f1 (ppm)

34

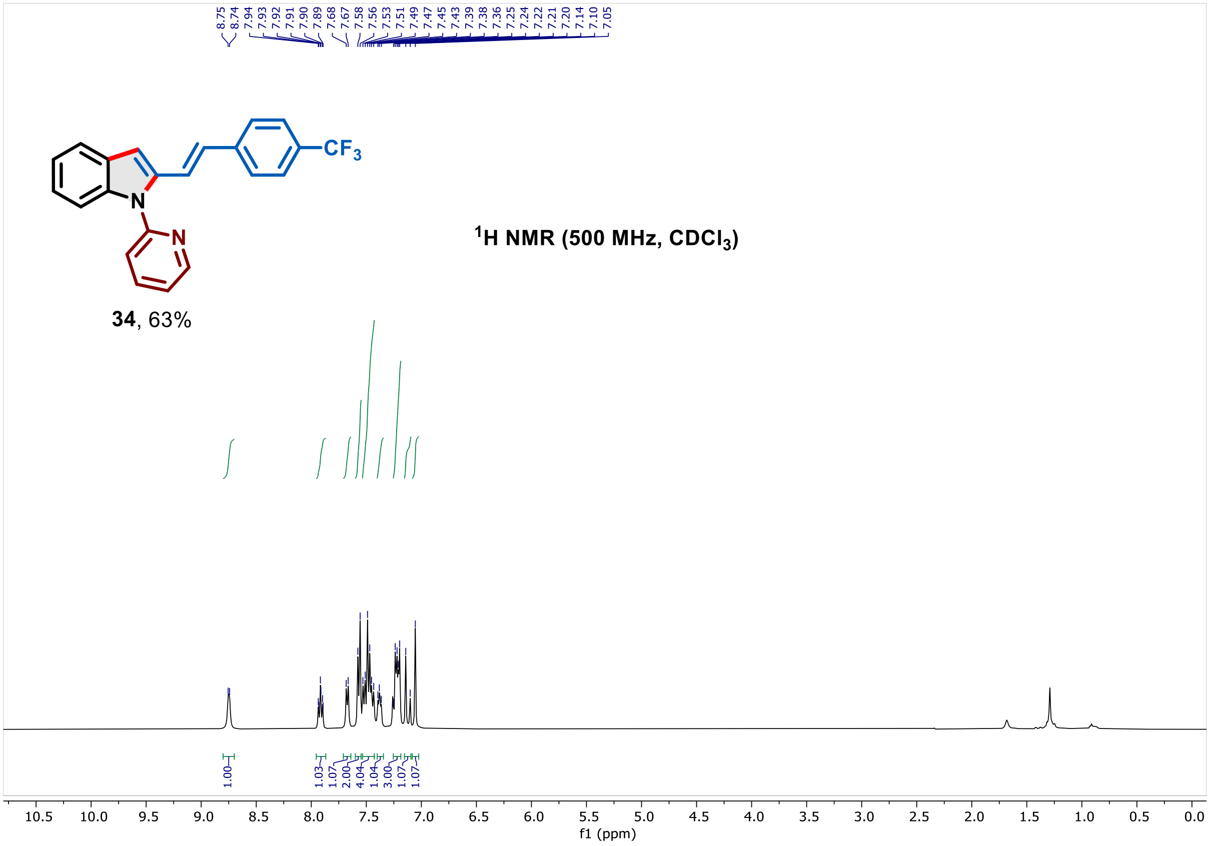
8.75
8.74
7.94
7.93
7.92
7.91
7.90
7.89
7.68
7.67
7.58
7.56
7.53
7.51
7.49
7.47
7.45
7.43
7.39
7.38
7.36
7.25
7.24
7.22
7.21
7.20
7.14
7.10
7.05
CF3
N
N
34, 63%
1H NMR (500 MHz, CDCl3)
1.00
1.03
1.07
2.00
4.04
1.04
3.00
1.07
1.07
f1 (ppm)

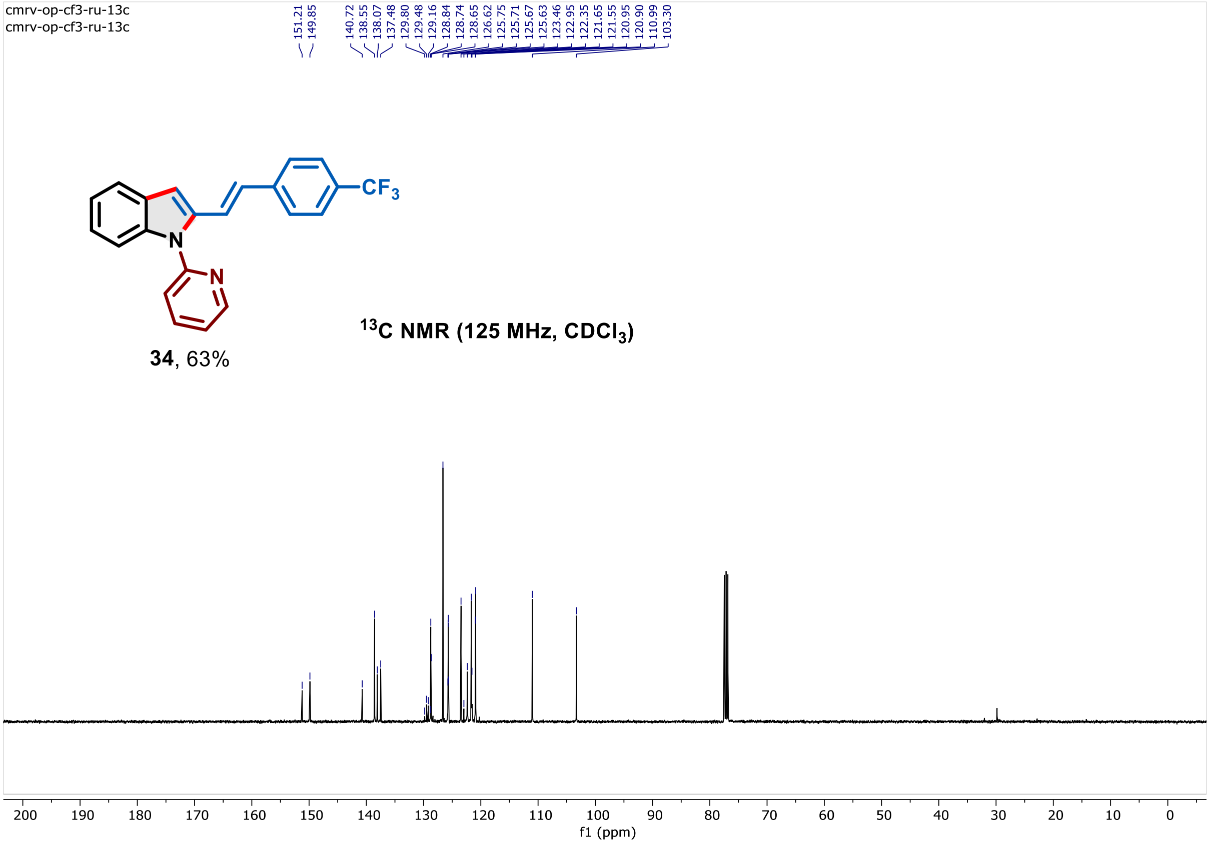
cmrv-op-cf3-ru-13c
cmrv-op-cf3-ru-13c
151.21
149.85
140.72
138.55
138.07
137.48
129.80
129.48
129.16
128.84
128.74
128.65
126.62
125.75
125.71
125.67
125.63
123.46
122.95
122.35
121.65
121.55
120.95
120.90
110.99
103.30
CF3
N
N
34, 63%
13C NMR (125 MHz, CDCl3)
f1 (ppm)

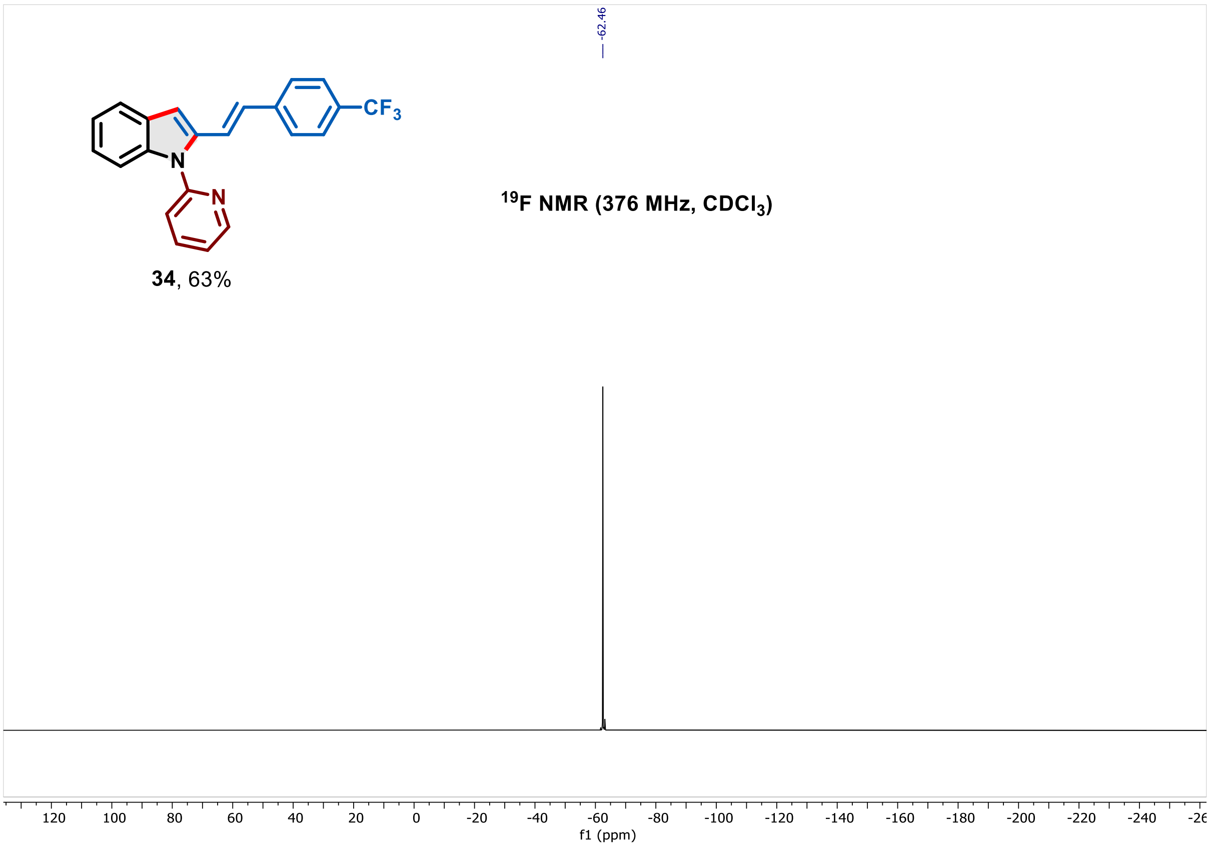
-62.46
CF3
N
N
34, 63%
19F NMR (376 MHz, CDCl3)
f1 (ppm)

35

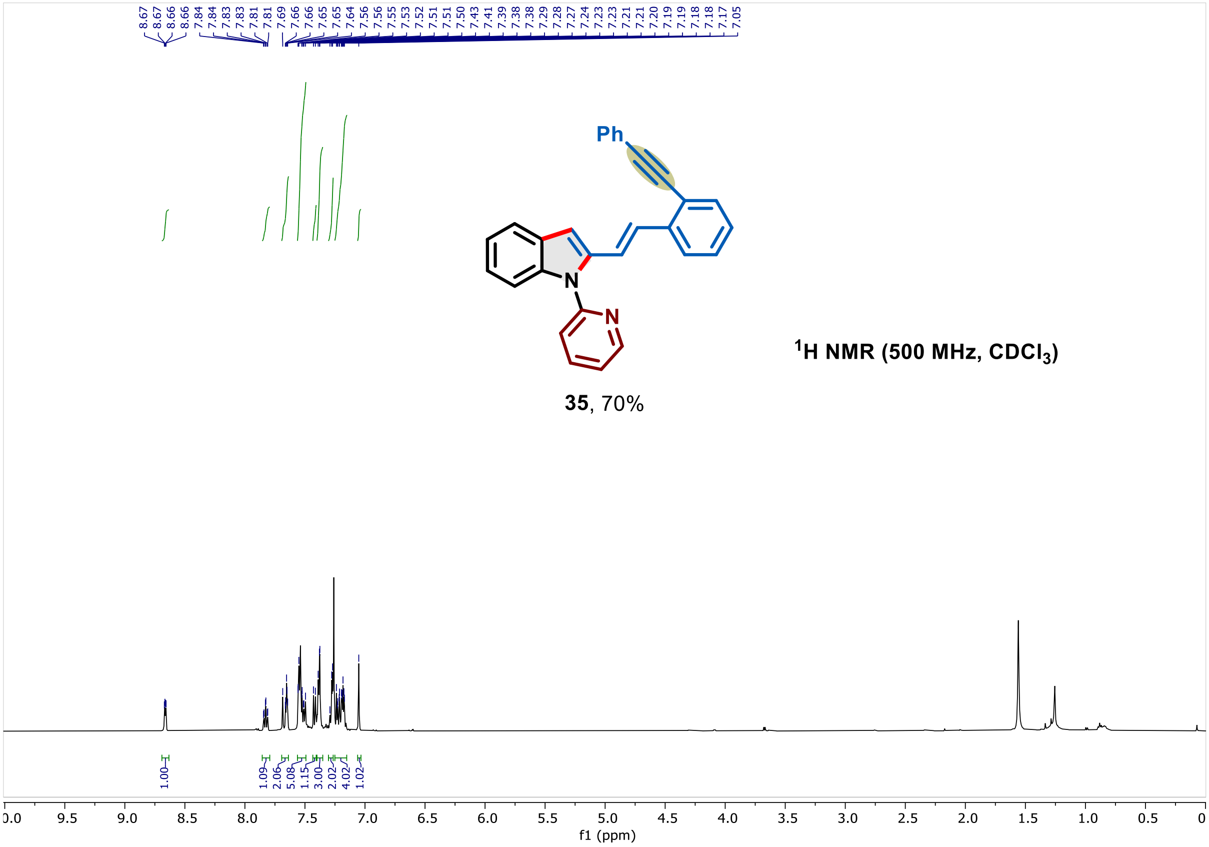
8.67 8.67 8.66 8.66 7.84 7.84 7.83 7.83 7.81 7.81 7.69 7.66 7.66 7.65 7.65 7.64 7.56 7.56 7.55 7.53 7.52 7.51 7.51 7.50 7.43 7.41 7.39 7.38 7.38 7.29 7.28 7.27 7.24 7.23 7.23 7.21 7.21 7.20 7.19 7.19 7.18 7.18 7.17 7.05
Ph
N
N
35, 70%
1H NMR (500 MHz, CDCl3)
1.00 1.09 2.06 5.08 1.15 3.00 2.02 4.02 1.02
f1 (ppm)

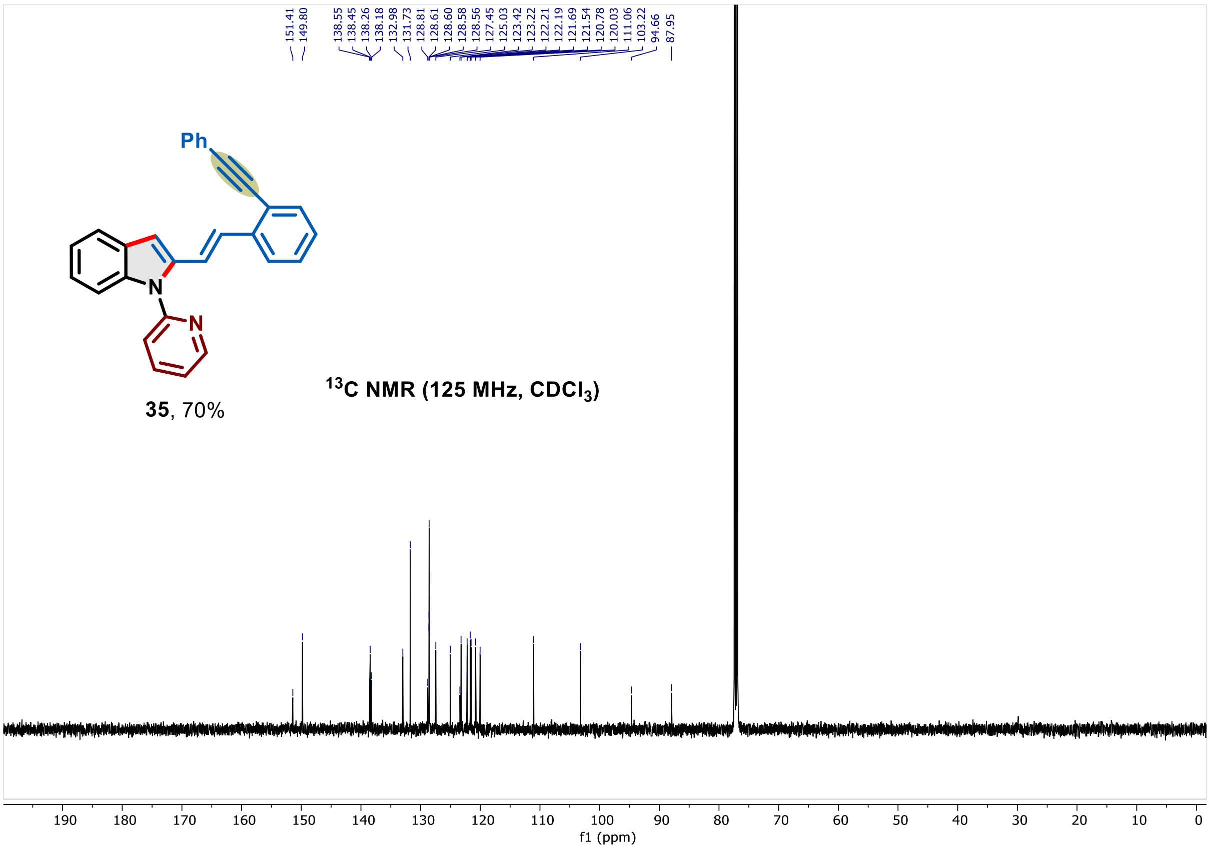
151.41 149.80 138.55 138.45 138.26 138.18 132.98 131.73 128.81 128.61 128.60 128.58 128.56 127.45 125.03 123.42 123.22 122.21 122.19 121.69 121.54 120.78 120.03 111.06 103.22 94.66 87.95
Ph
N
N
35, 70%
13C NMR (125 MHz, CDCl3)
f1 (ppm)

36

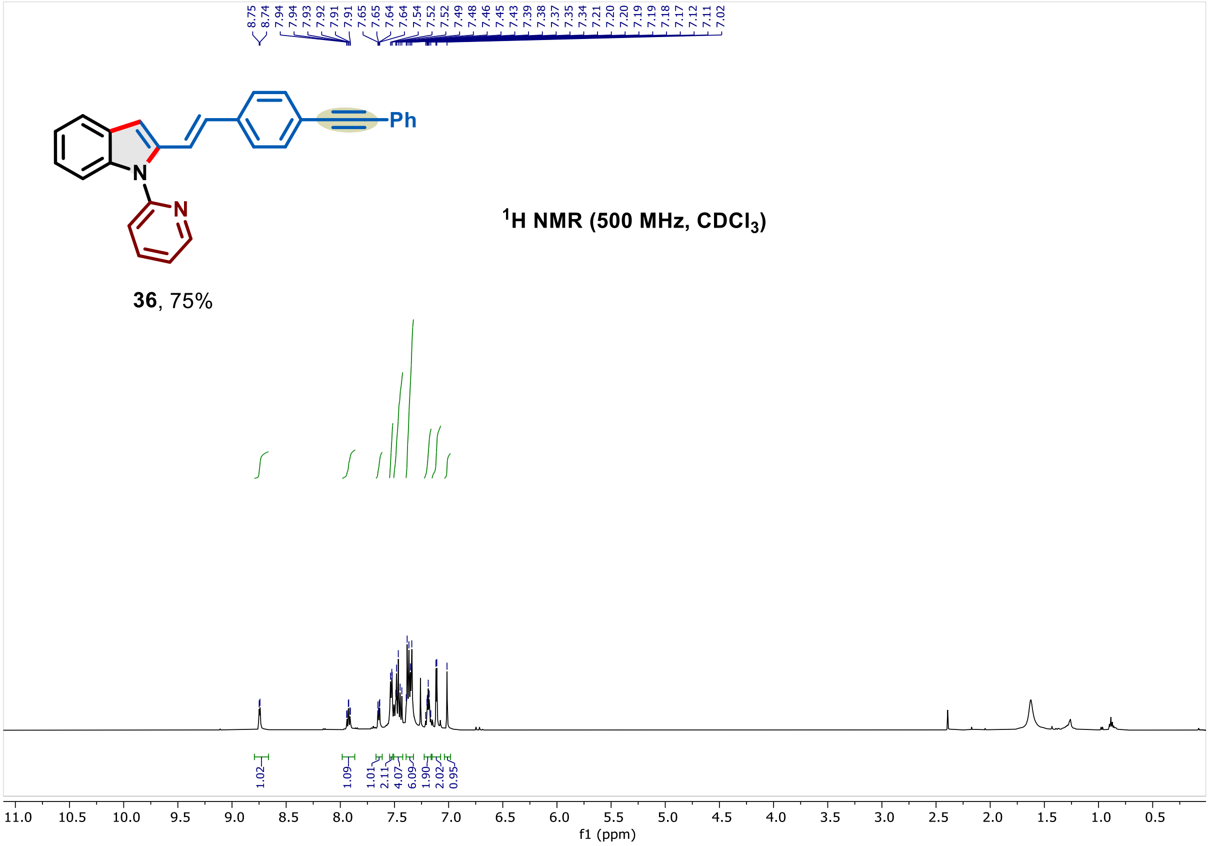
8.75
8.74
7.94
7.94
7.93
7.92
7.91
7.91
7.65
7.65
7.64
7.64
7.54
7.52
7.52
7.49
7.48
7.46
7.45
7.43
7.39
7.38
7.37
7.35
7.34
7.21
7.20
7.20
7.19
7.19
7.18
7.17
7.12
7.11
7.02
Ph
N
N
36, 75%
1H NMR (500 MHz, CDCl3)
1.02
1.09
1.01
2.11
4.07
6.09
1.90
2.02
0.95
f1 (ppm)

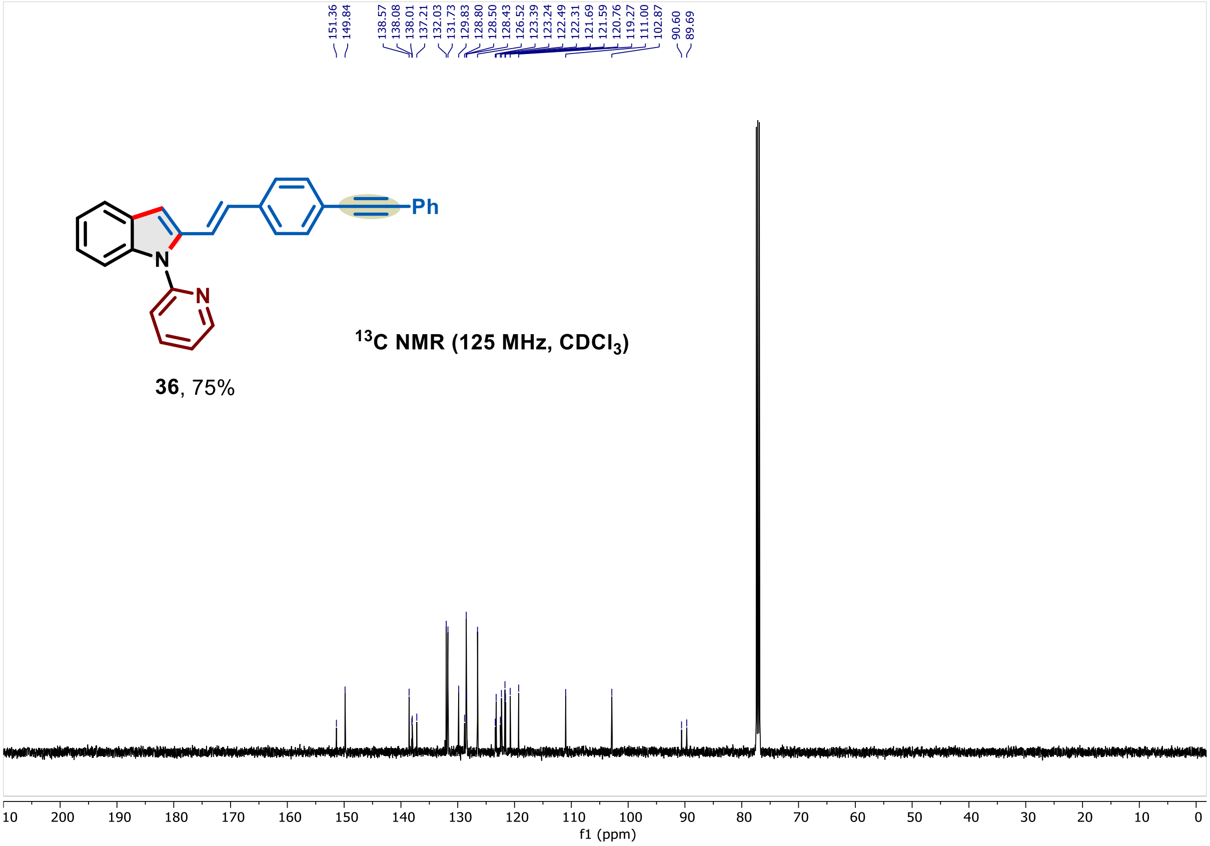
151.36
149.84
138.57
138.08
138.01
137.21
132.03
131.73
129.83
128.80
128.50
128.43
126.52
123.39
123.24
122.49
122.31
121.69
121.59
120.76
119.27
111.00
102.87
90.60
89.69
Ph
N
N
13C NMR (125 MHz, CDCl3)
36, 75%
f1 (ppm)

37

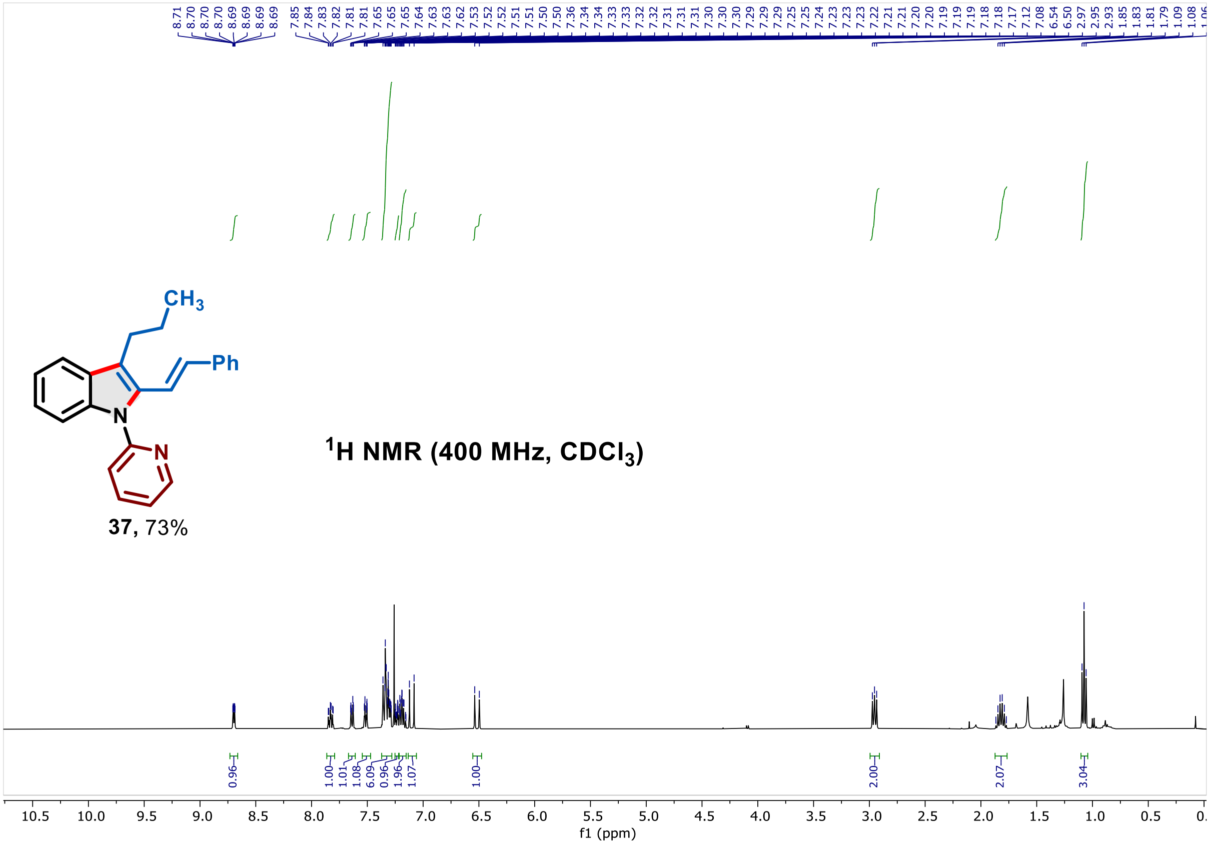
CH3
Ph
N
N
37, 73%
1H NMR (400 MHz, CDCl3)
f1 (ppm)

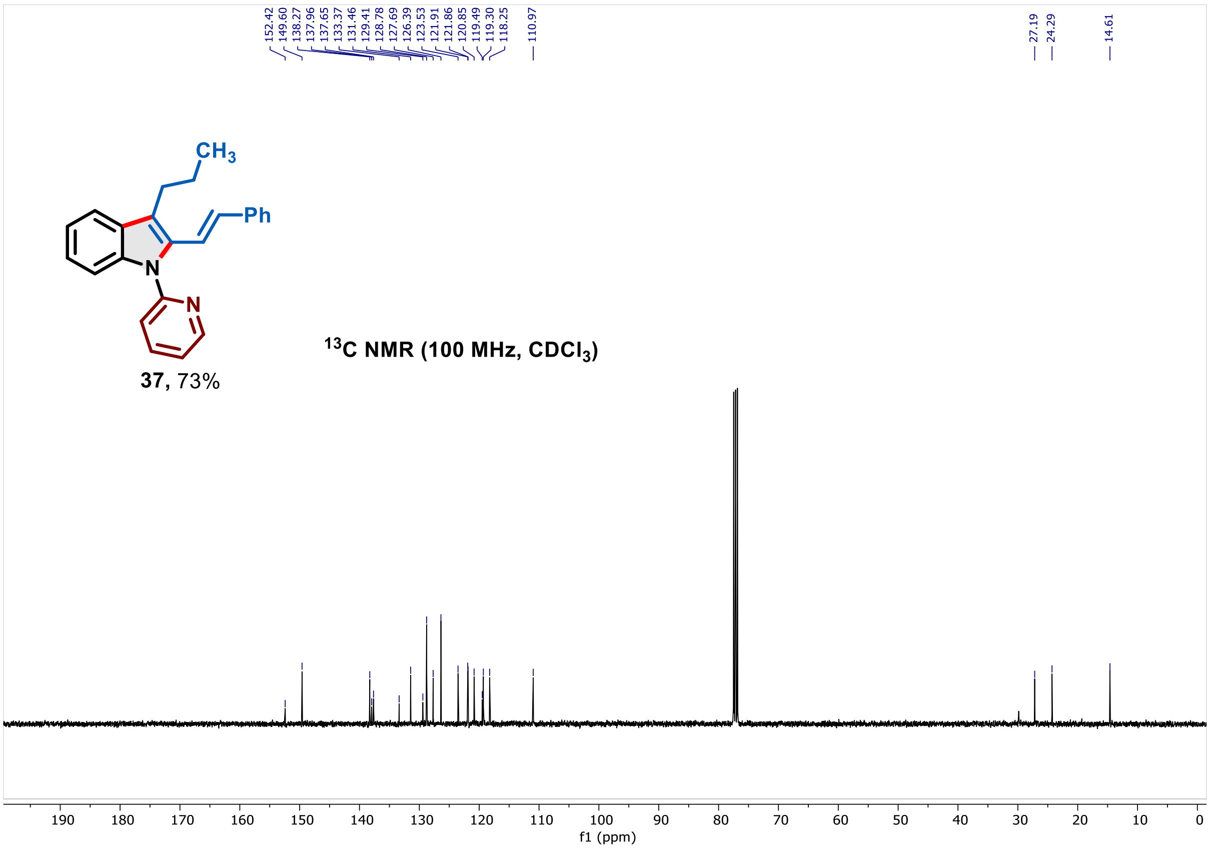
152.42
149.60
138.27
137.96
137.65
133.37
131.46
129.41
128.78
127.69
126.39
123.53
121.91
121.86
120.85
119.49
119.30
118.25
110.97
27.19
24.29
14.61
CH3
Ph
N
N
37, 73%
13C NMR (100 MHz, CDCl3)
f1 (ppm)

38

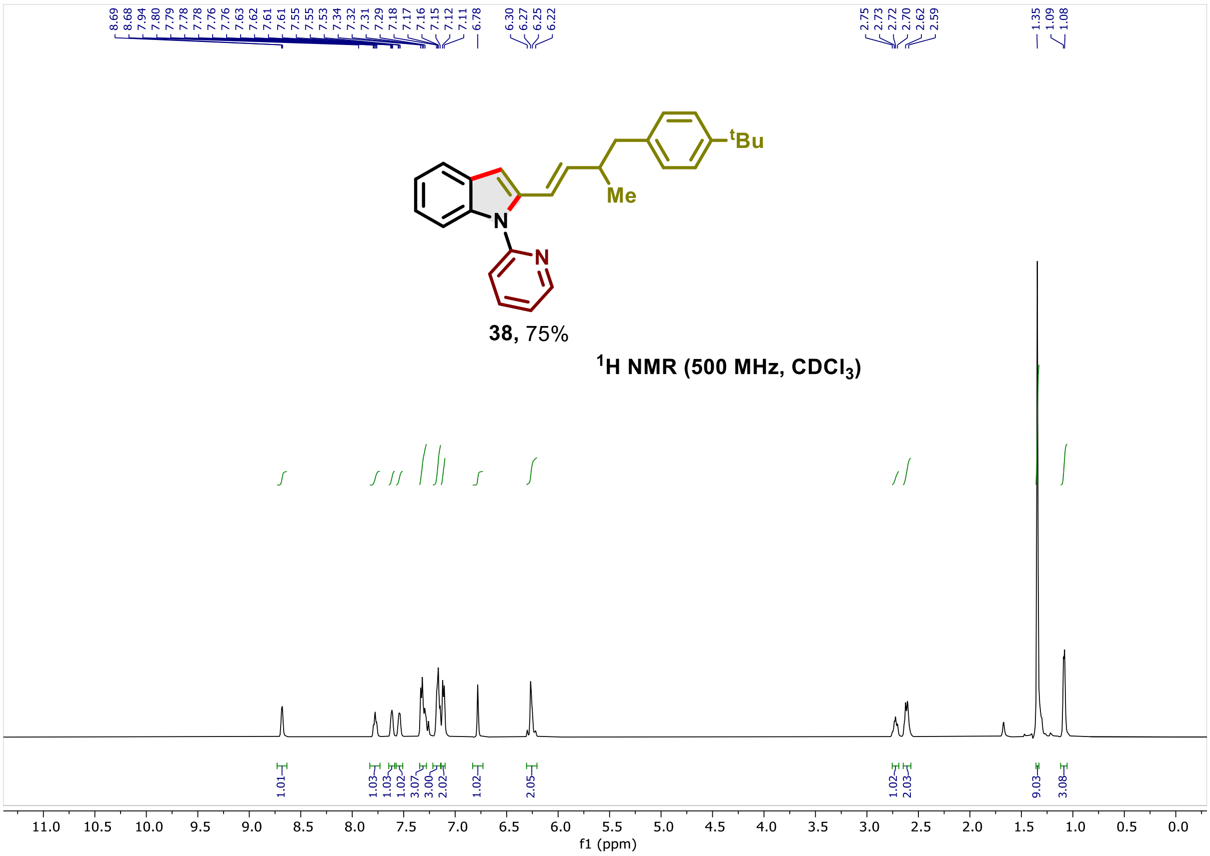
8.69 8.68 7.94 7.80 7.79 7.78 7.78 7.76 7.76 7.63 7.62 7.61 7.61 7.55 7.55 7.53 7.34 7.32 7.31 7.29 7.18 7.17 7.16 7.15 7.12 7.11 6.78 6.30 6.27 6.25 6.22
2.75 2.73 2.72 2.70 2.62 2.59 1.35 1.09 1.08
N
N
Me
tBu
38, 75%
1H NMR (500 MHz, CDCl3)
1.01 1.03 1.03 1.02 3.07 3.00 2.02 1.02 2.05 1.02 2.03 9.03 3.08
f1 (ppm)

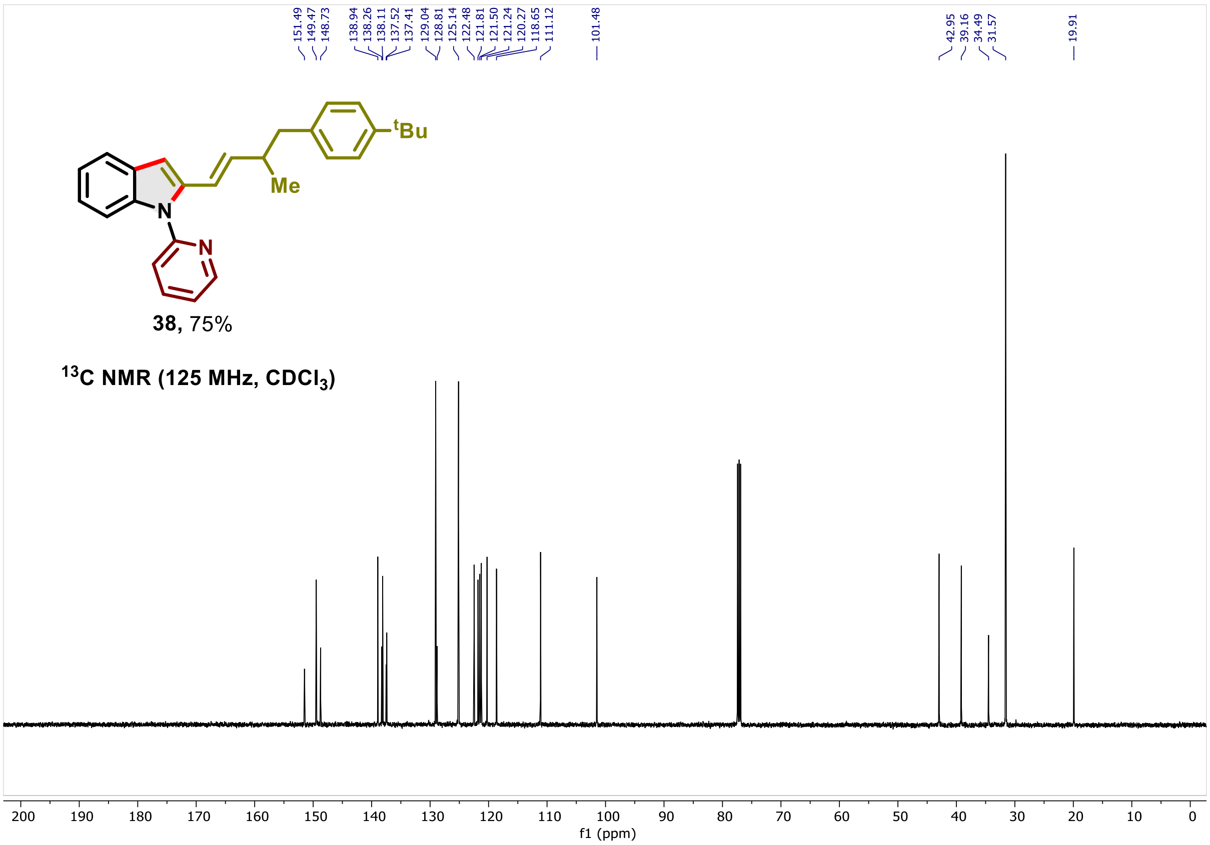
151.49 149.47 148.73 138.94 138.26 138.11 137.52 137.41 129.04 128.81 125.14 122.48 121.81 121.50 121.24 120.27 118.65 111.12 101.48
42.95 39.16 34.49 31.57 19.91
N
N
Me
tBu
38, 75%
13C NMR (125 MHz, CDCl3)
f1 (ppm)

39

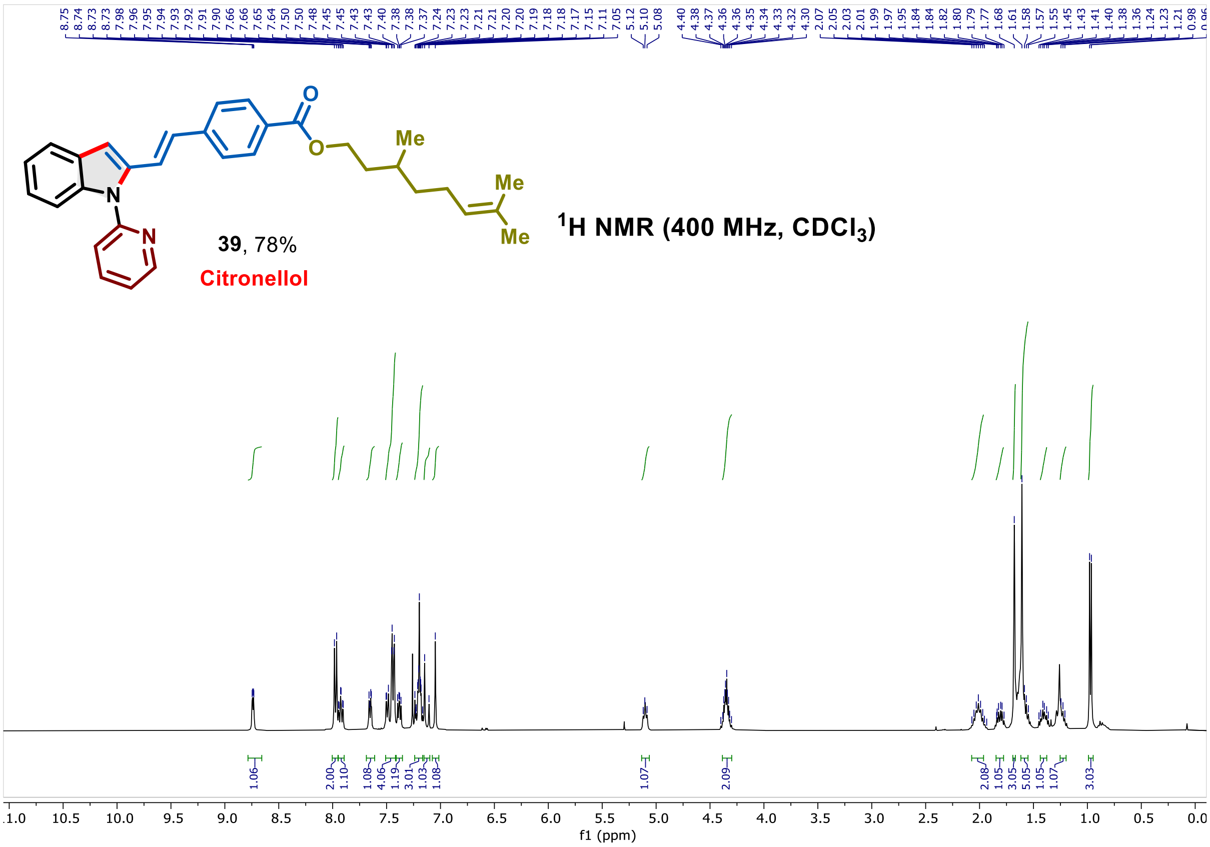
39, 78%
Citronellol
1H NMR (400 MHz, CDCl3)
f1 (ppm)

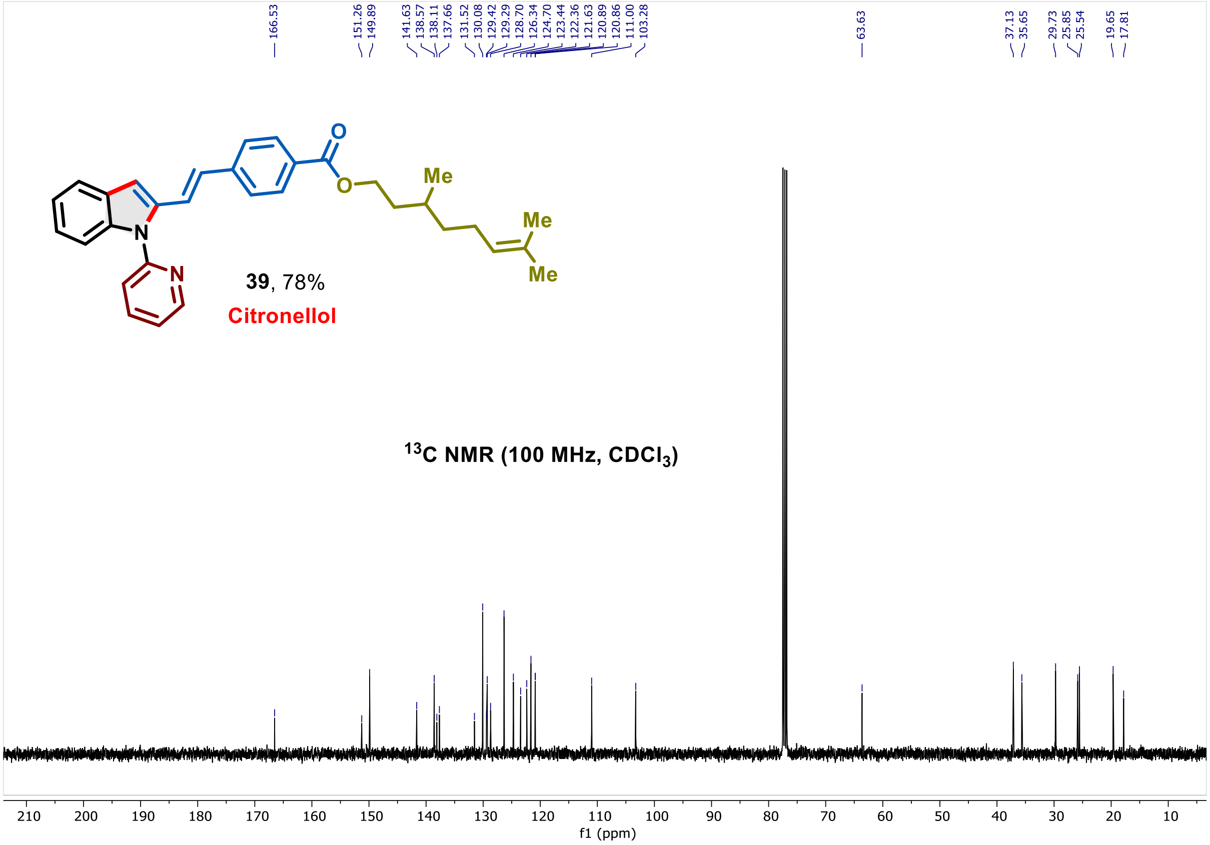
39, 78%
Citronellol
13C NMR (100 MHz, CDCl3)
f1 (ppm)

40

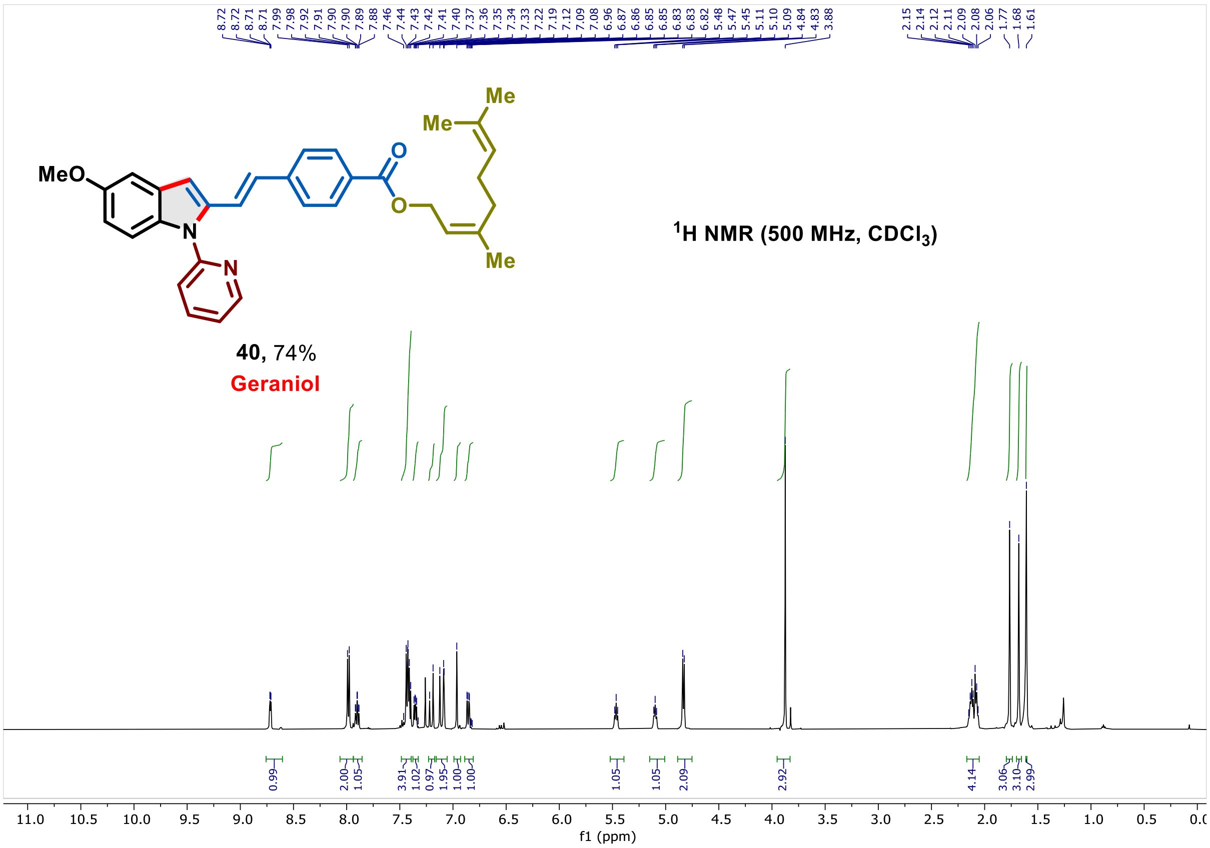

MeO
N
N
O
O
Me
Me
Me
40, 74%
Geraniol
^{1}H NMR (500 MHz, CDCl$_3$)
f1 (ppm)

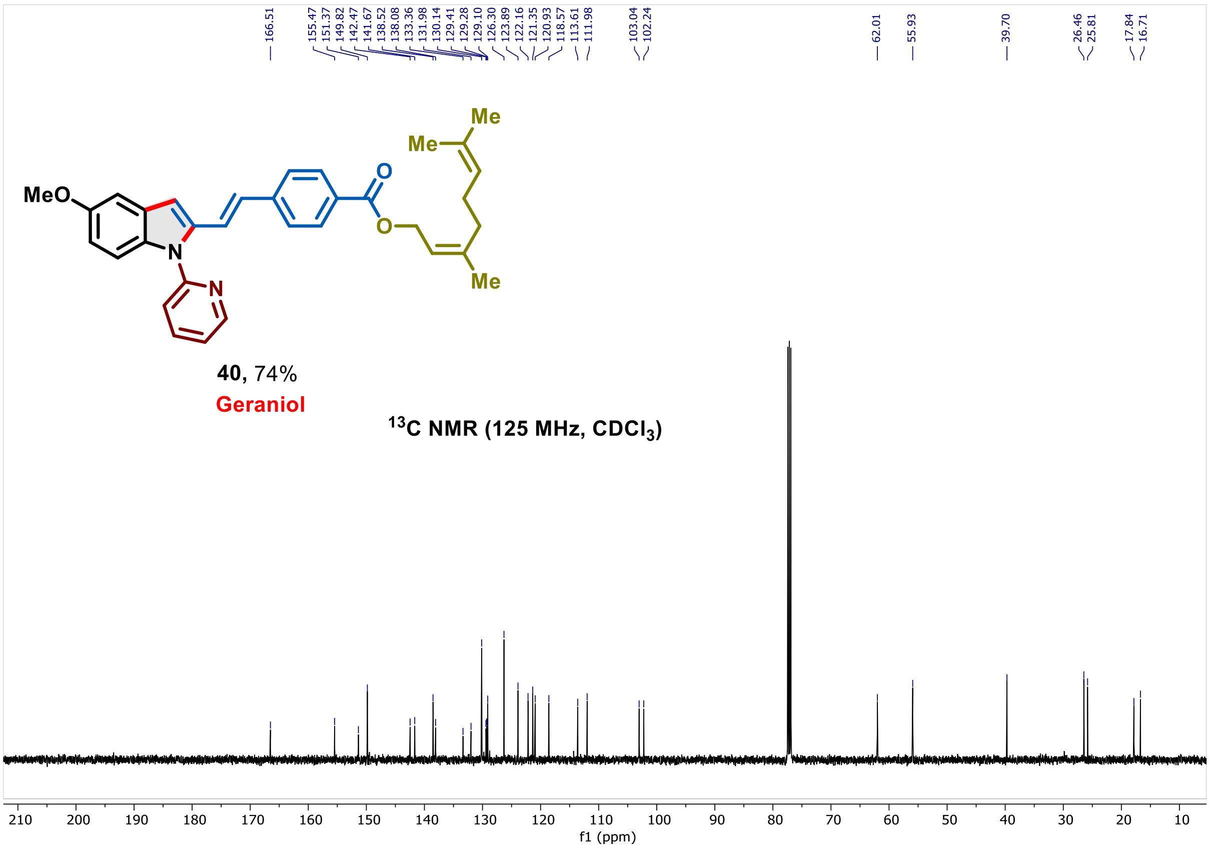

MeO
N
N
O
O
Me
Me
Me
40, 74%
Geraniol
^{13}C NMR (125 MHz, CDCl$_3$)
f1 (ppm)

41

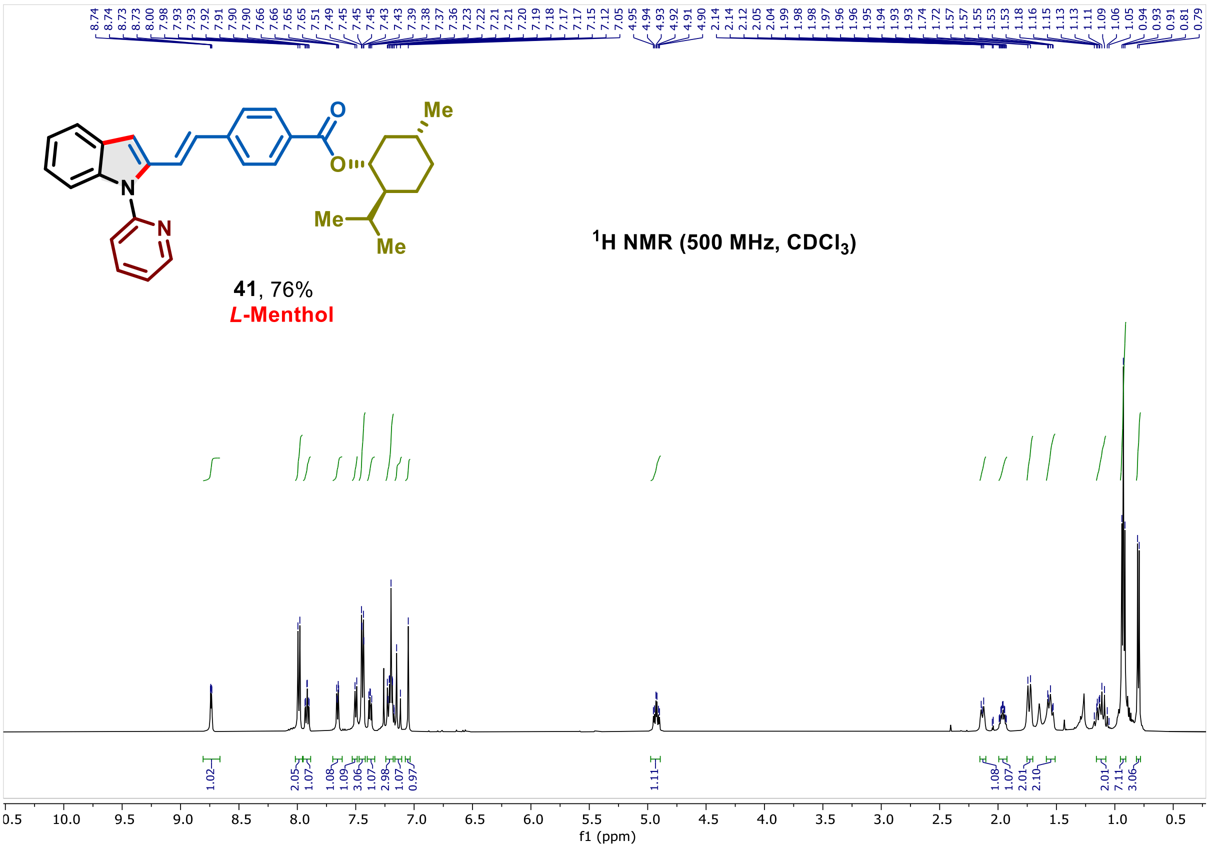

41, 76%
L-Menthol
^{1}H NMR (500 MHz, $CDCl_3$)
f1 (ppm)

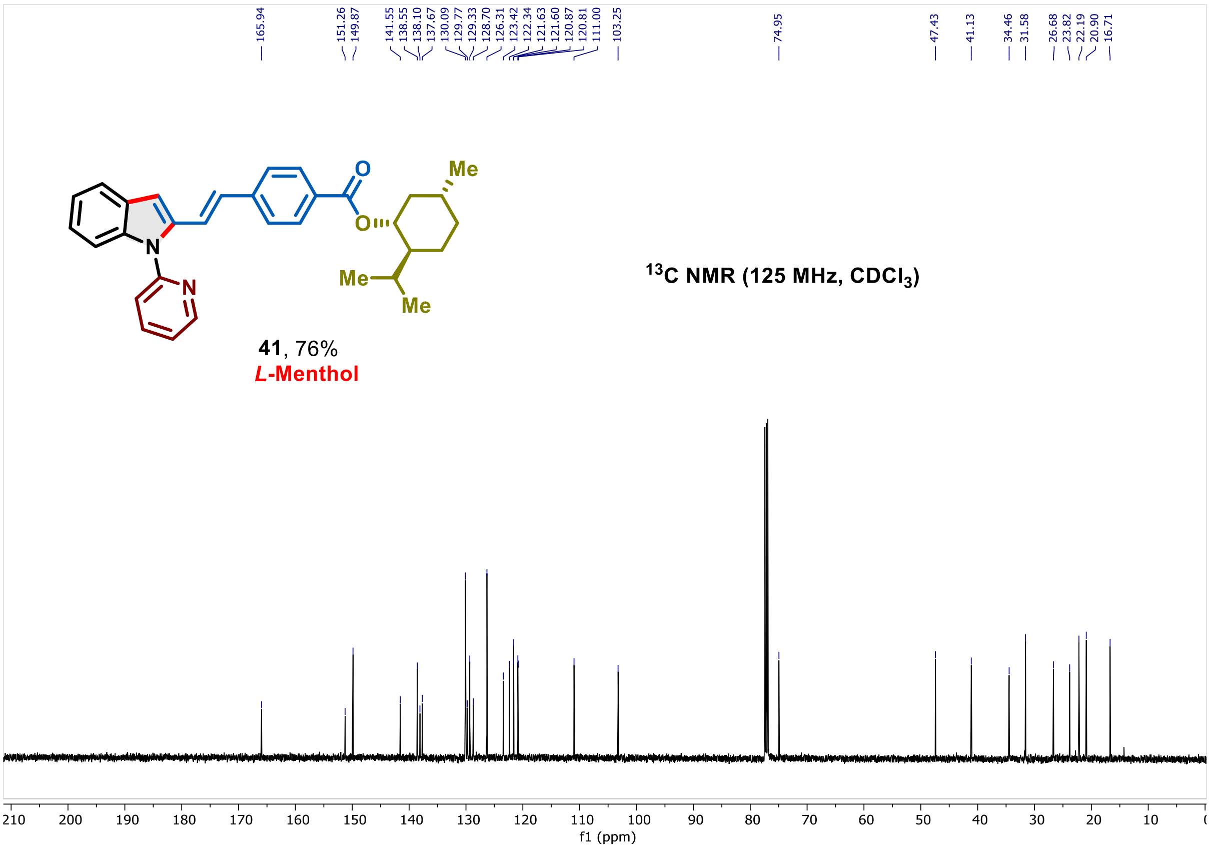

41, 76%
L-Menthol
^{13}C NMR (125 MHz, $CDCl_3$)
f1 (ppm)

42

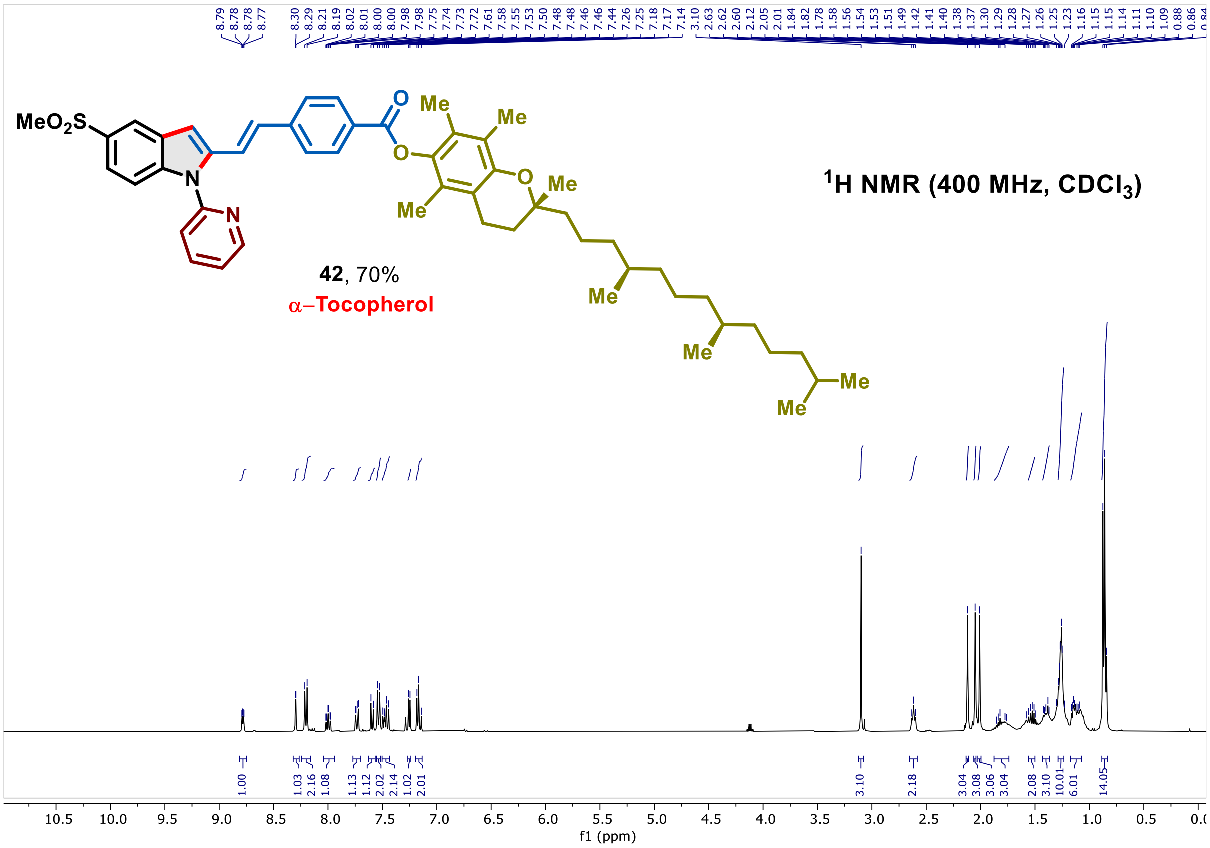
MeO2S
N
N
O
O
O
Me
Me
Me
Me
Me
Me
Me
Me
42, 70%
α–Tocopherol
1H NMR (400 MHz, CDCl3)
f1 (ppm)

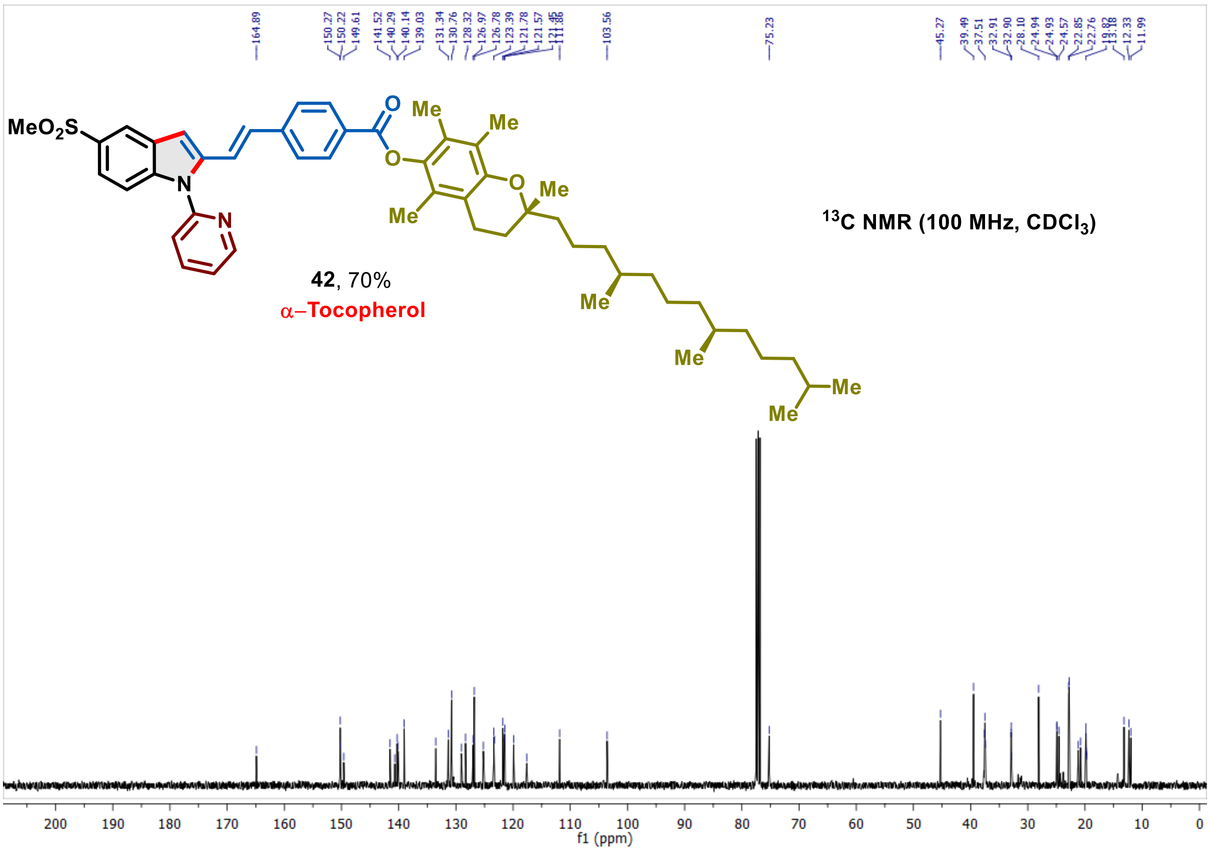
MeO2S
N
N
O
O
O
Me
Me
Me
Me
Me
Me
Me
Me
42, 70%
α–Tocopherol
13C NMR (100 MHz, CDCl3)
f1 (ppm)

43

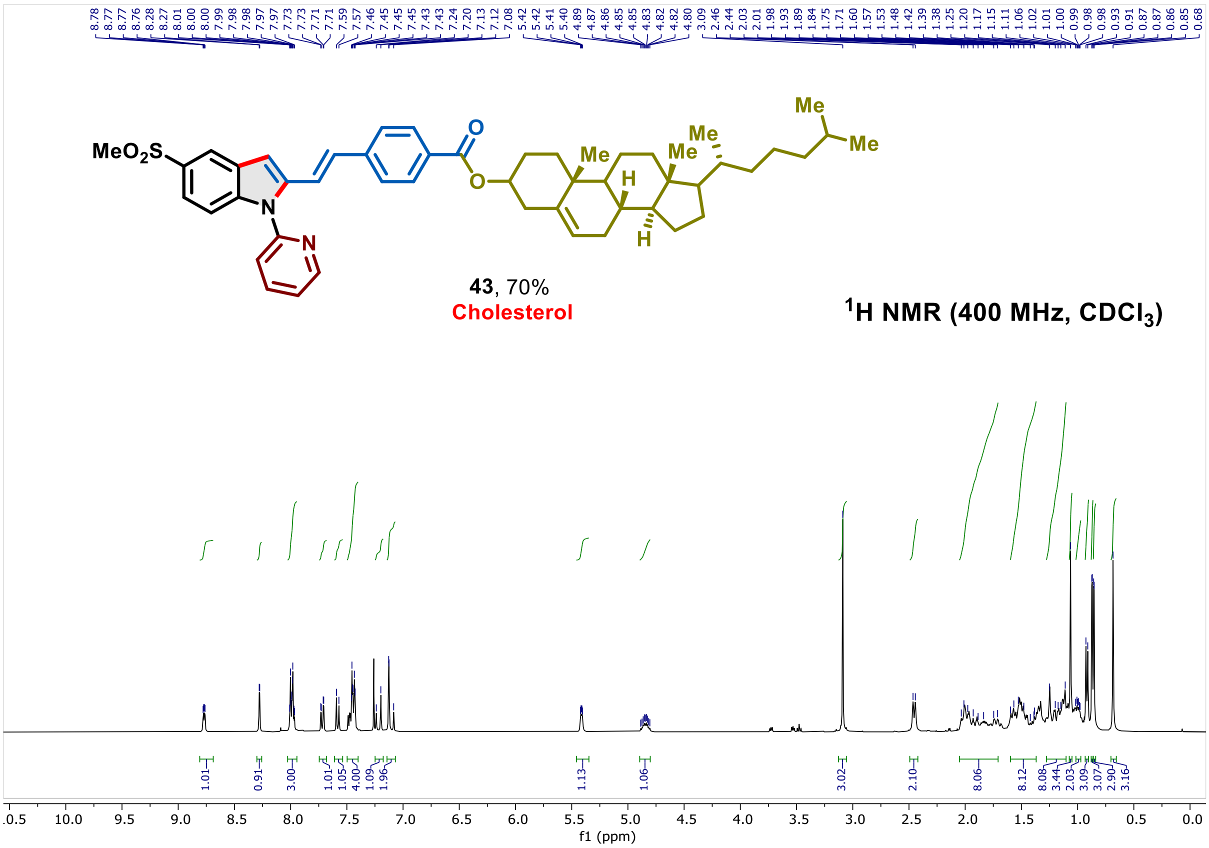
MeO2S
N
N
O
O
Me
Me
Me
Me
H
H
43, 70%
Cholesterol
1H NMR (400 MHz, CDCl3)
f1 (ppm)

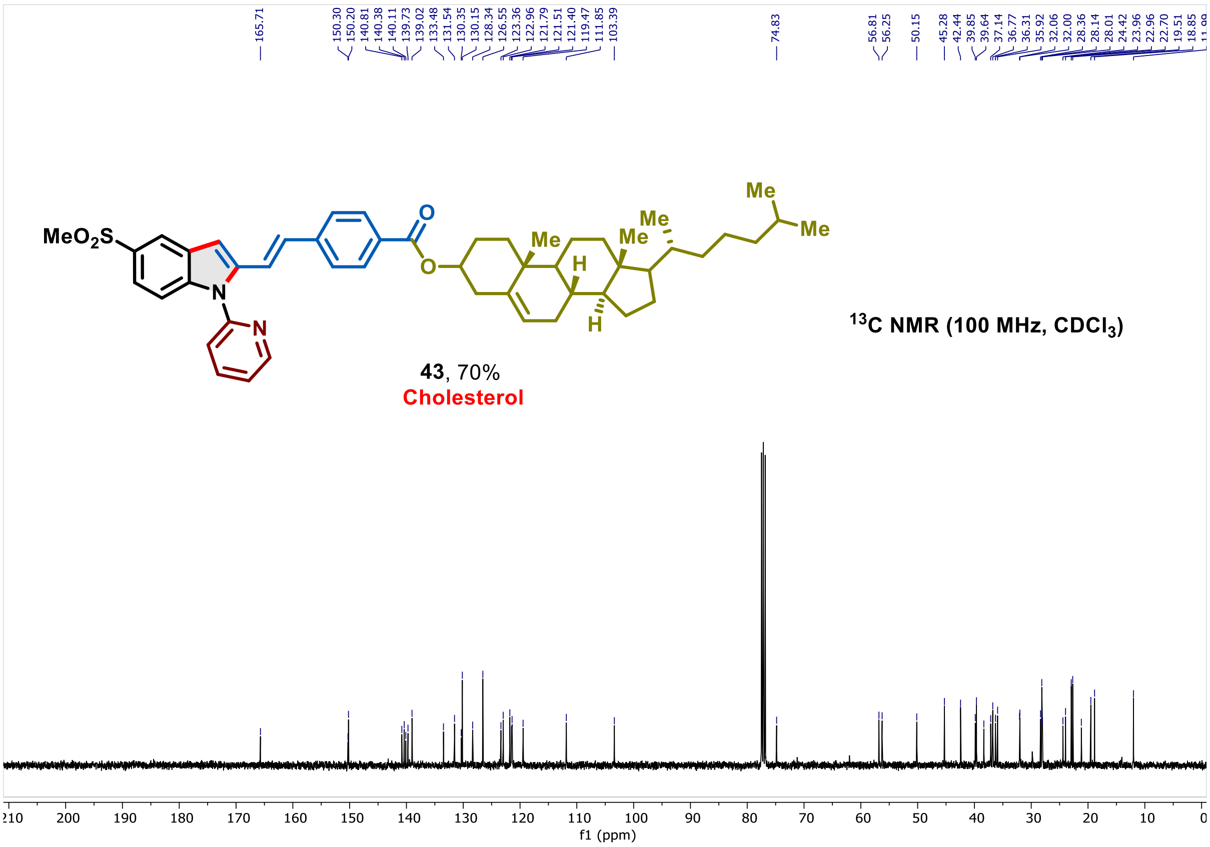
MeO2S
N
N
O
O
Me
Me
Me
Me
H
H
43, 70%
Cholesterol
13C NMR (100 MHz, CDCl3)
f1 (ppm)

44

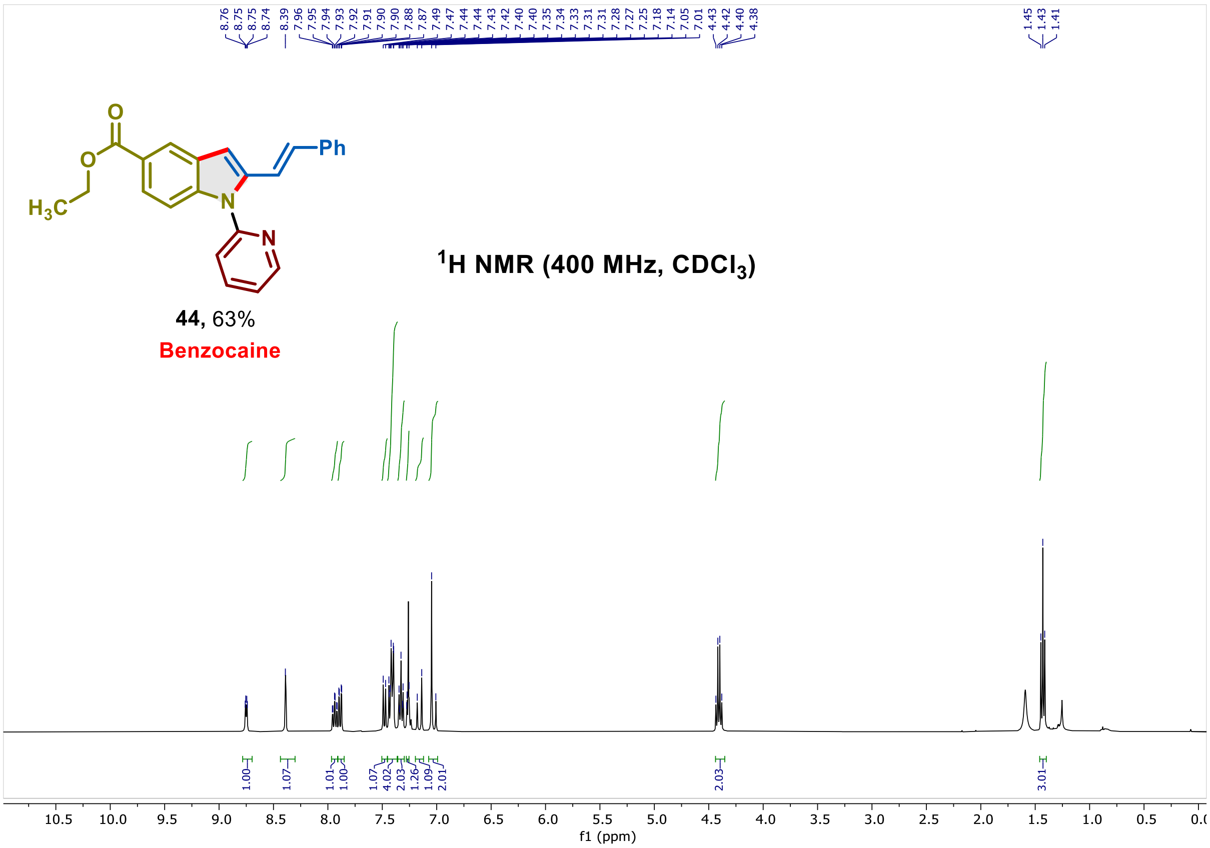
8.76
8.75
8.75
8.74
8.39
7.96
7.95
7.94
7.93
7.92
7.91
7.90
7.90
7.88
7.87
7.49
7.47
7.47
7.44
7.44
7.43
7.42
7.40
7.40
7.35
7.34
7.33
7.31
7.31
7.28
7.27
7.25
7.18
7.14
7.05
7.01
4.43
4.42
4.40
4.38
1.45
1.43
1.41
O
O
H_3C
N
N
Ph
44, 63%
Benzocaine
^{1}H NMR (400 MHz, $CDCl_3$)
1.00
1.07
1.01
1.00
1.07
4.02
2.03
1.26
1.09
2.01
2.03
3.01
f1 (ppm)

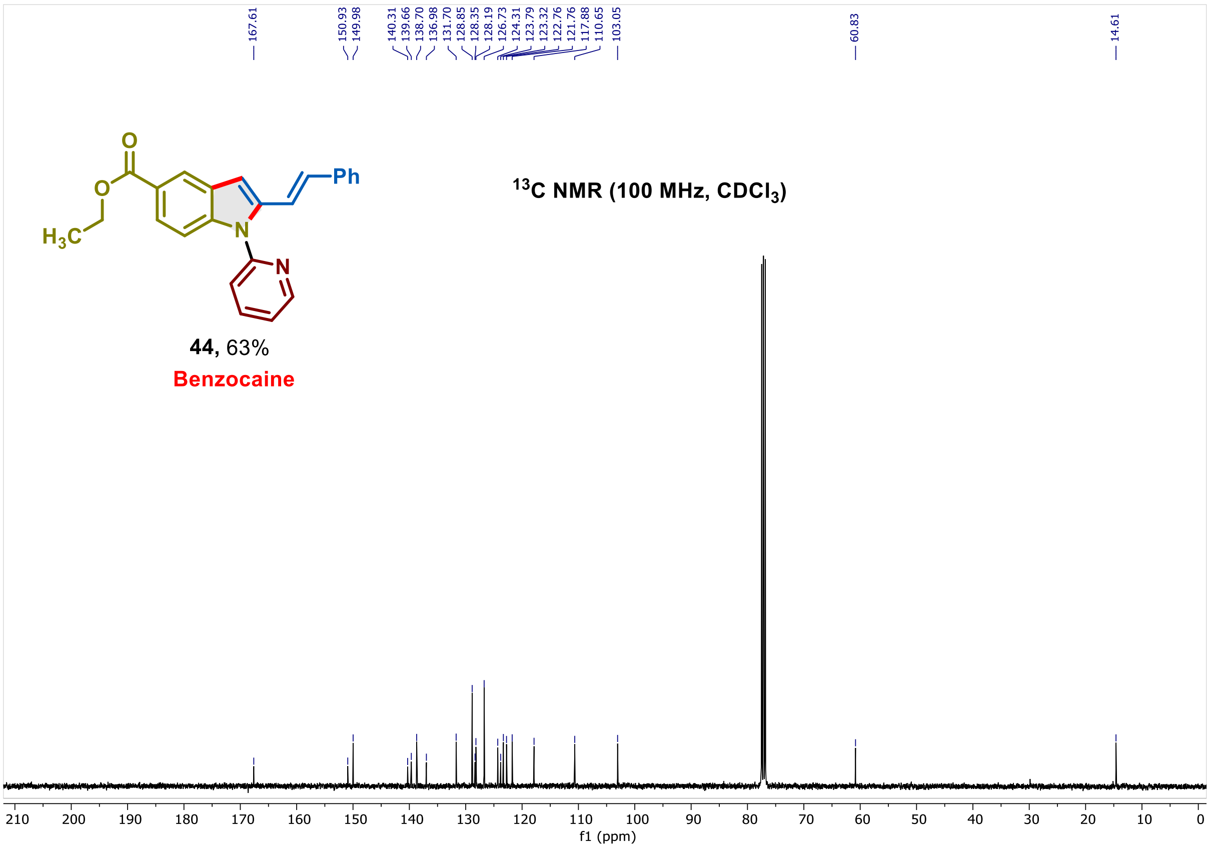
167.61
150.93
149.98
140.31
139.66
138.70
136.98
131.70
128.85
128.35
128.19
126.73
124.31
123.79
123.32
122.76
121.76
117.88
110.65
103.05
60.83
14.61
O
O
H_3C
N
N
Ph
44, 63%
Benzocaine
^{13}C NMR (100 MHz, $CDCl_3$)
f1 (ppm)

45

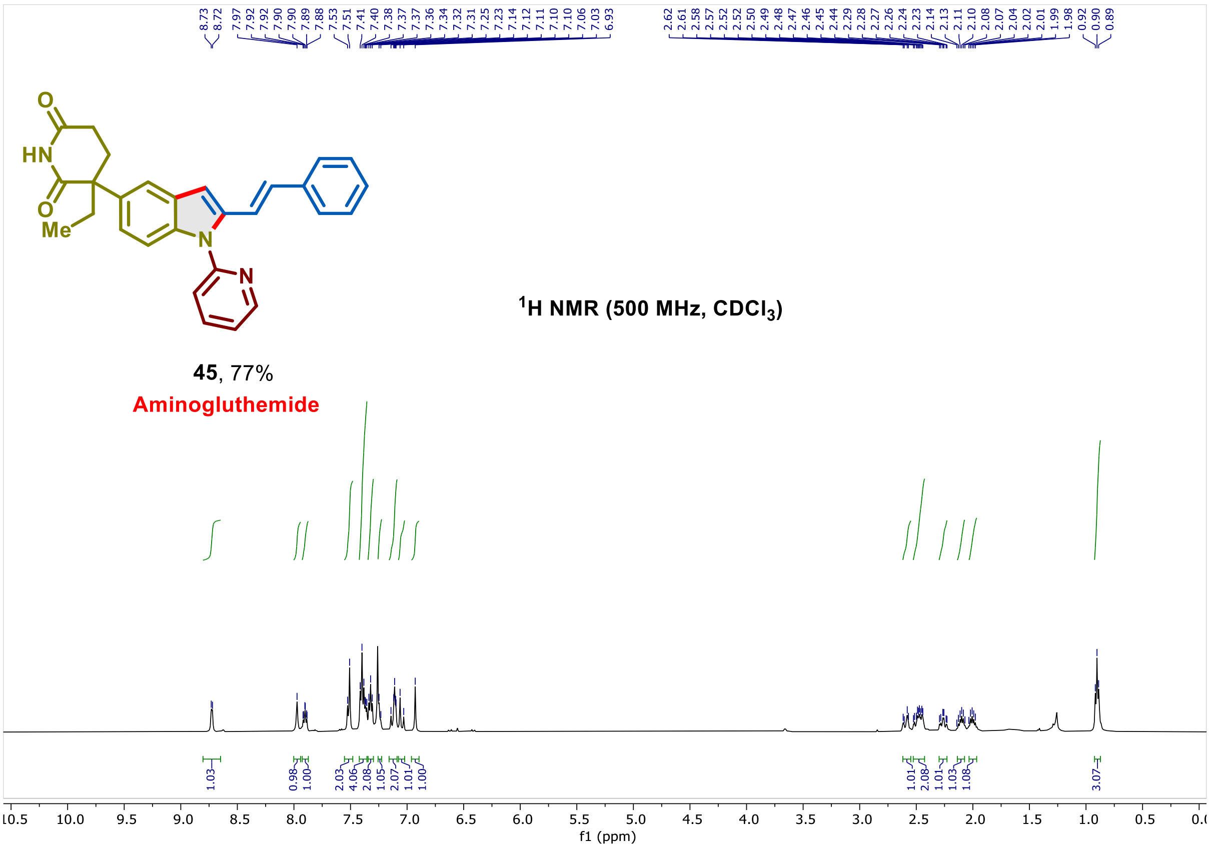

O
HN
O
Me
N
N
45, 77%
Aminogluthemide
1H NMR (500 MHz, CDCl3)
f1 (ppm)

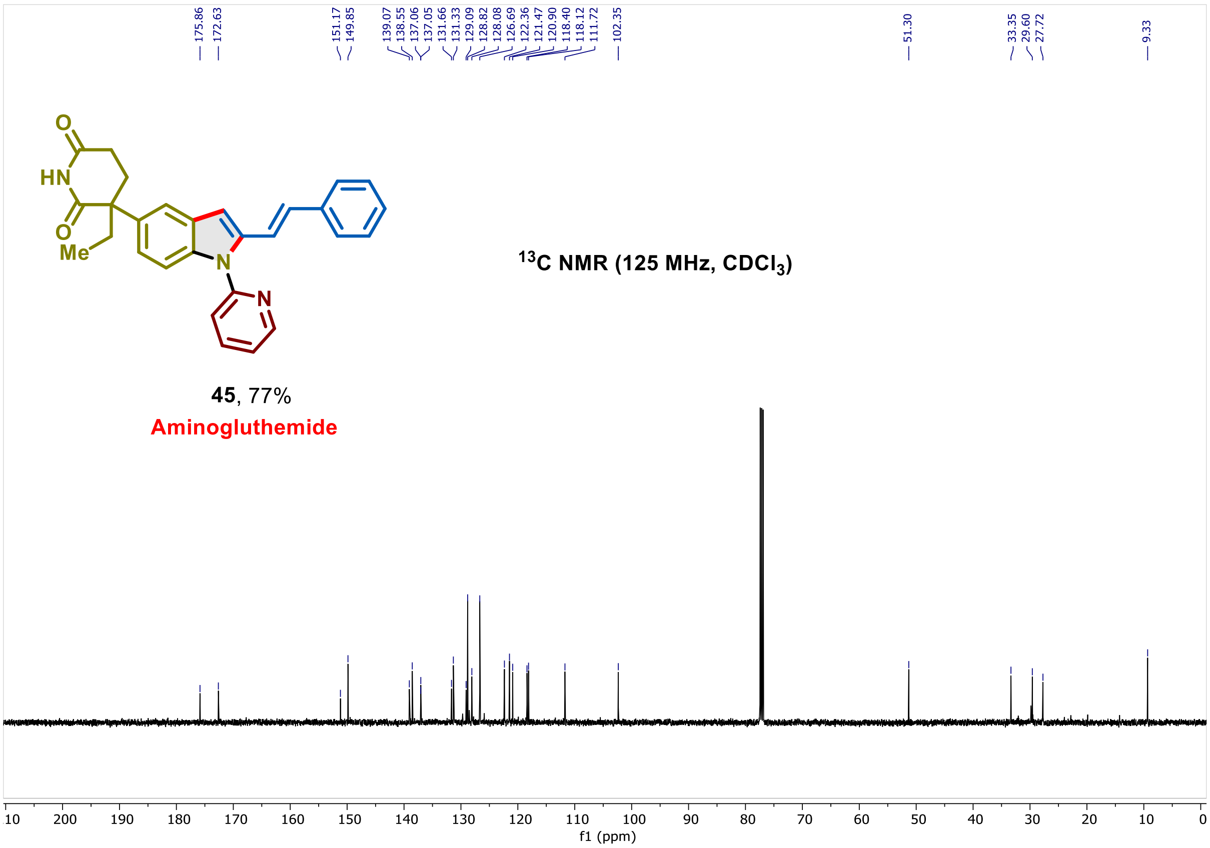

O
HN
O
Me
N
N
45, 77%
Aminogluthemide
13C NMR (125 MHz, CDCl3)
f1 (ppm)

46

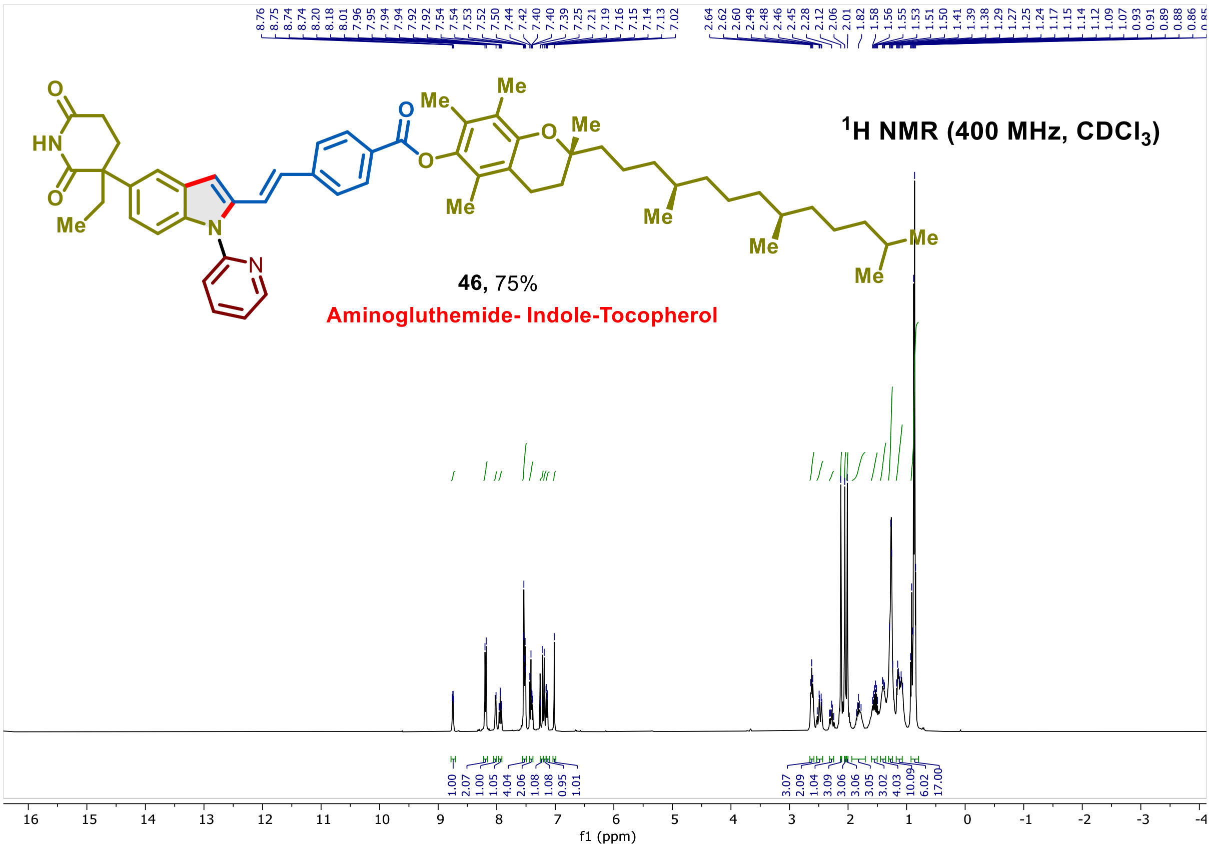

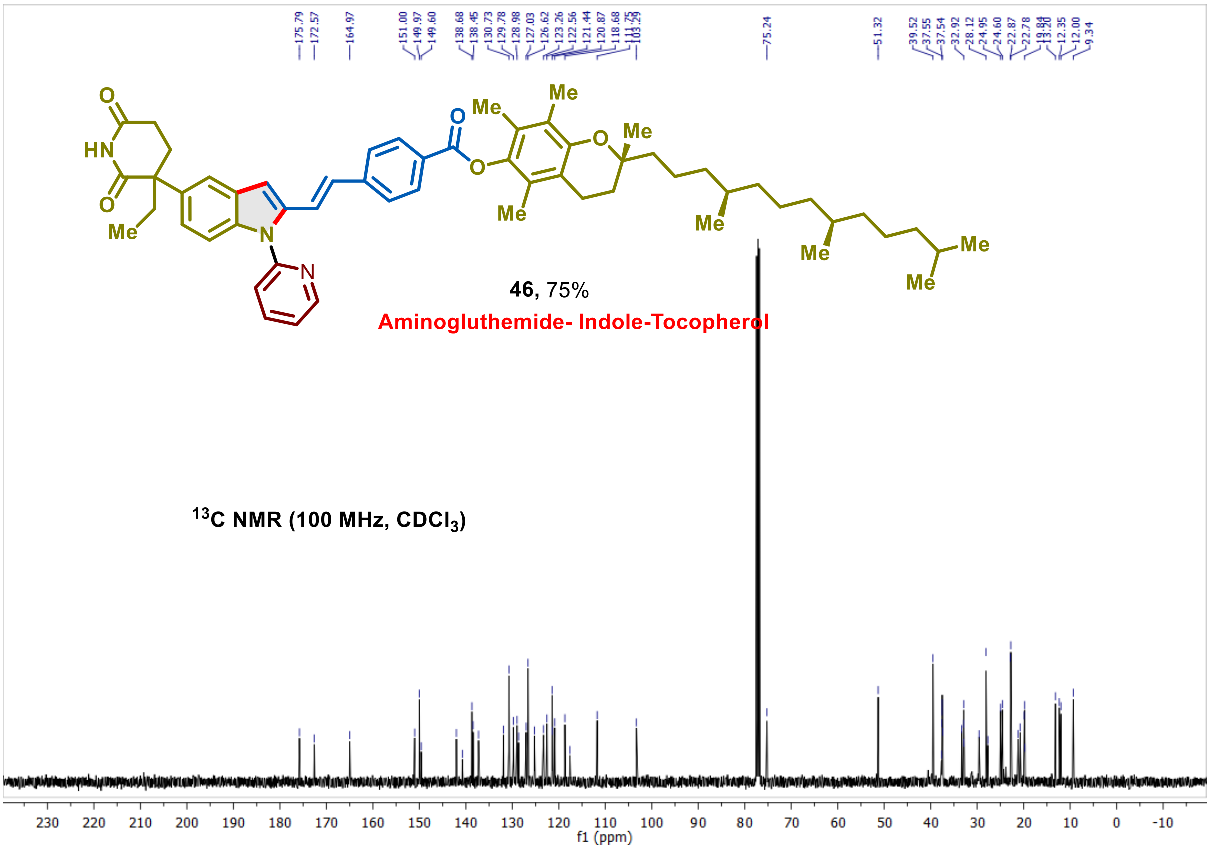

50

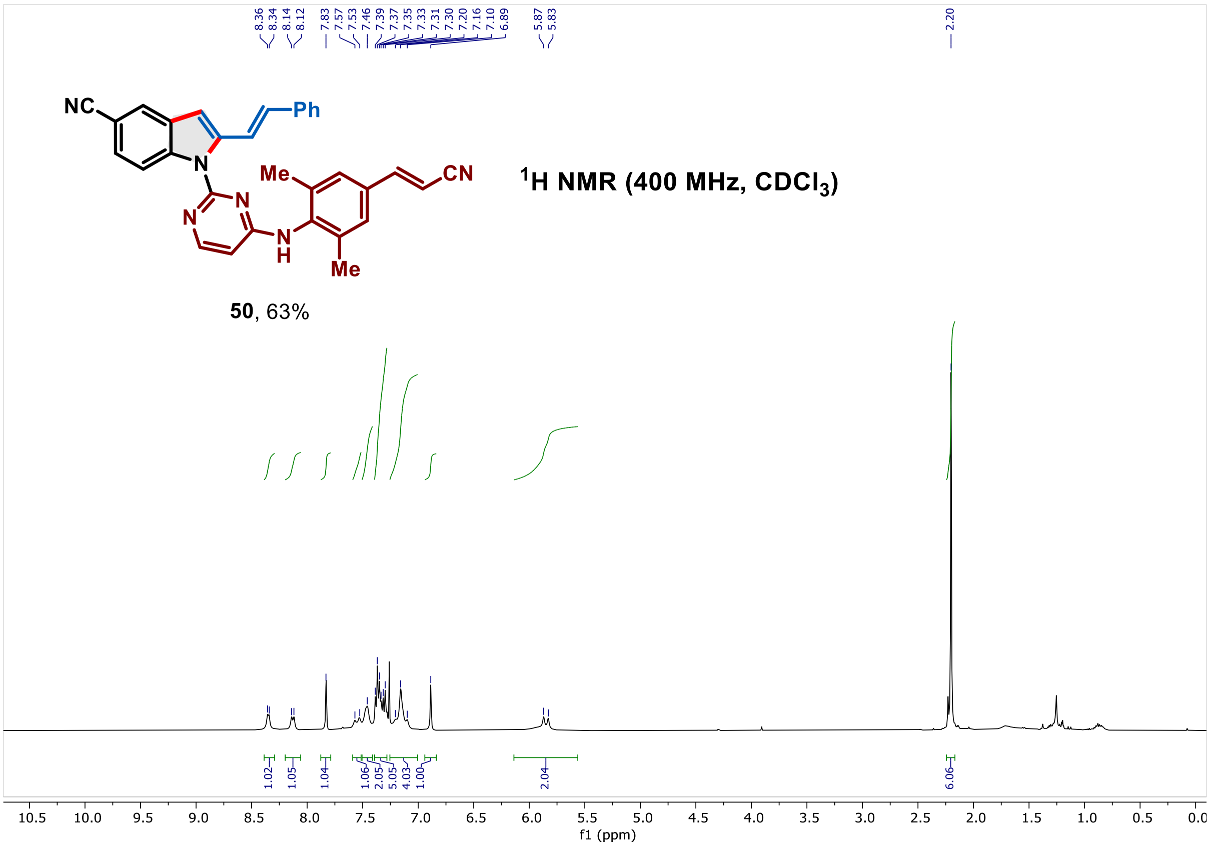
1H NMR (400 MHz, CDCl3)
50, 63%
f1 (ppm)

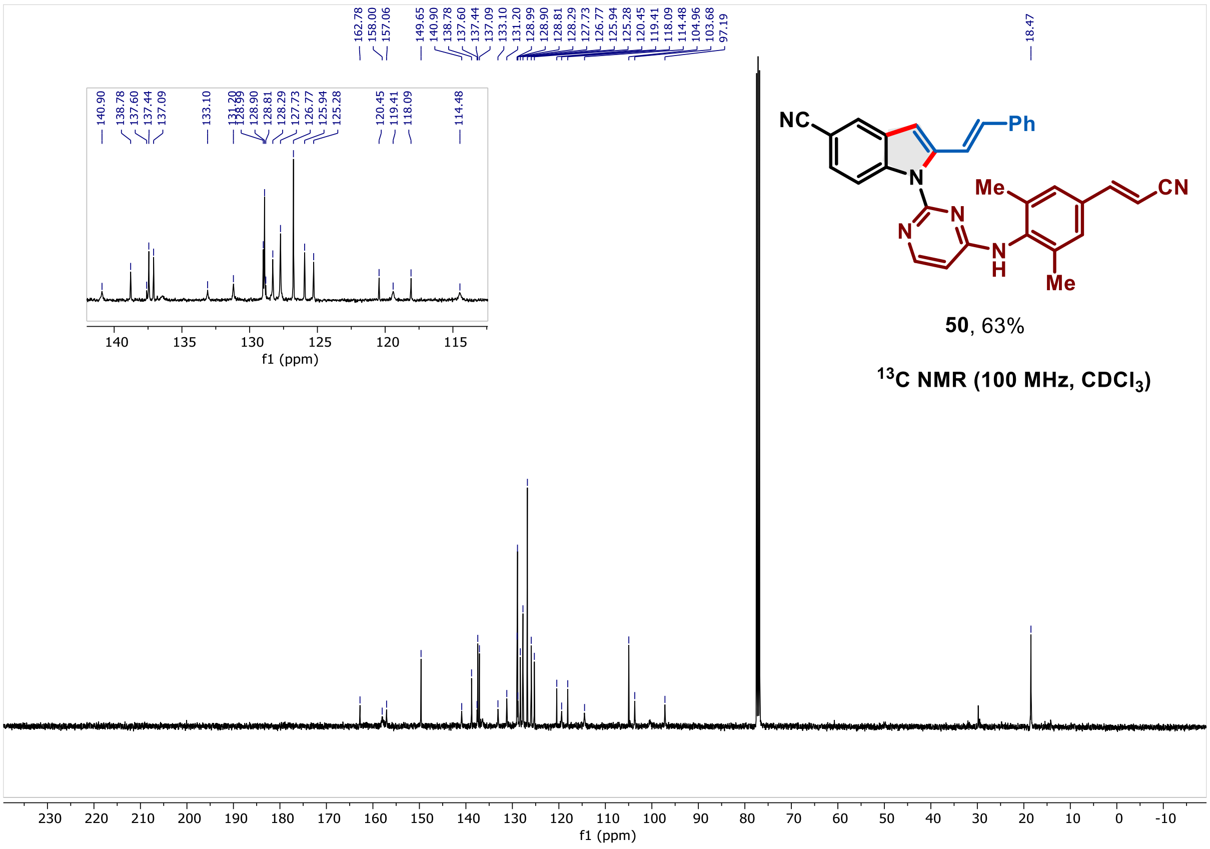
13C NMR (100 MHz, CDCl3)
50, 63%
f1 (ppm)

51

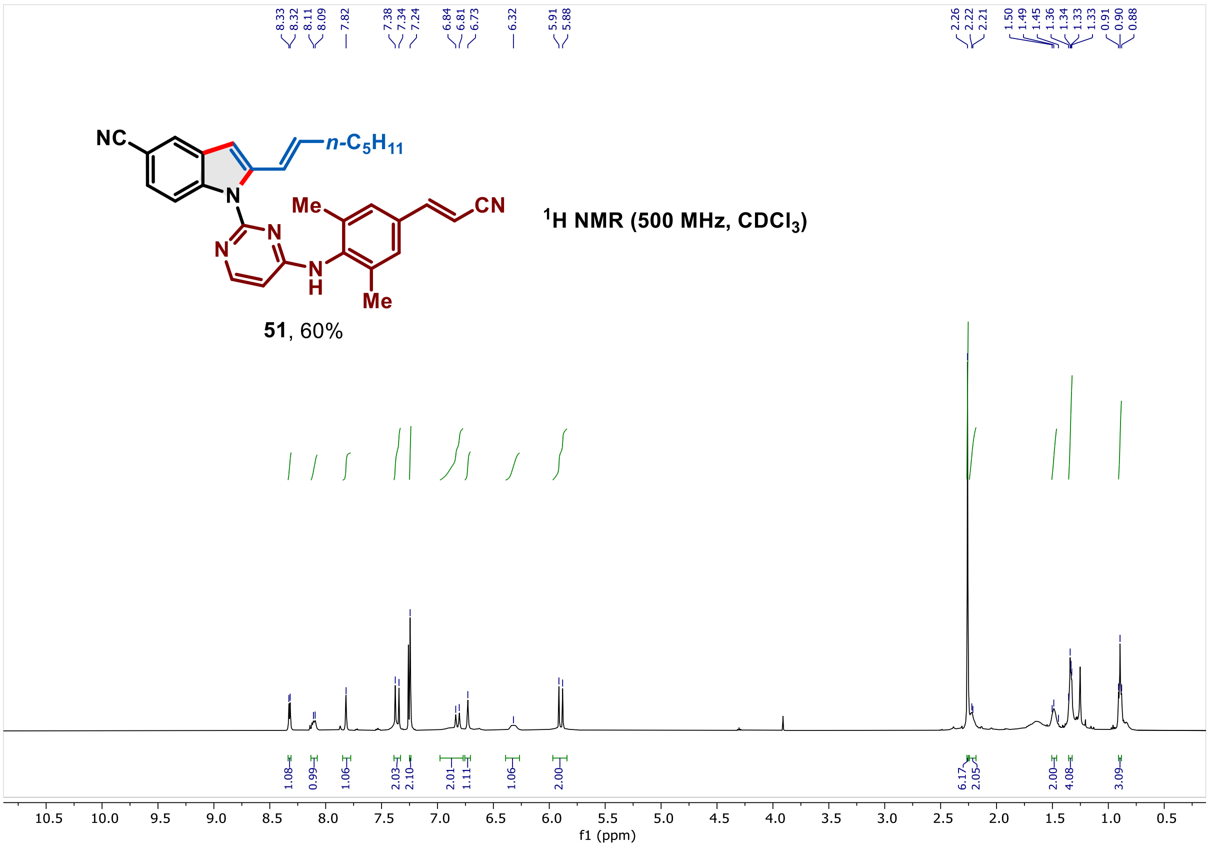
NC
n-C5H11
N
Me
CN
N
N
N
H
Me
51, 60%
1H NMR (500 MHz, CDCl3)
8.33 8.32 8.11 8.09 7.82 7.38 7.34 7.24 6.84 6.81 6.73 6.32 5.91 5.88 2.26 2.22 2.21 1.50 1.49 1.45 1.36 1.34 1.33 1.33 0.91 0.90 0.88
1.08 0.99 1.06 2.03 2.10 2.01 1.11 1.06 2.00 6.17 2.05 2.00 4.08 3.09
10.5 10.0 9.5 9.0 8.5 8.0 7.5 7.0 6.5 6.0 5.5 5.0 4.5 4.0 3.5 3.0 2.5 2.0 1.5 1.0 0.5
f1 (ppm)

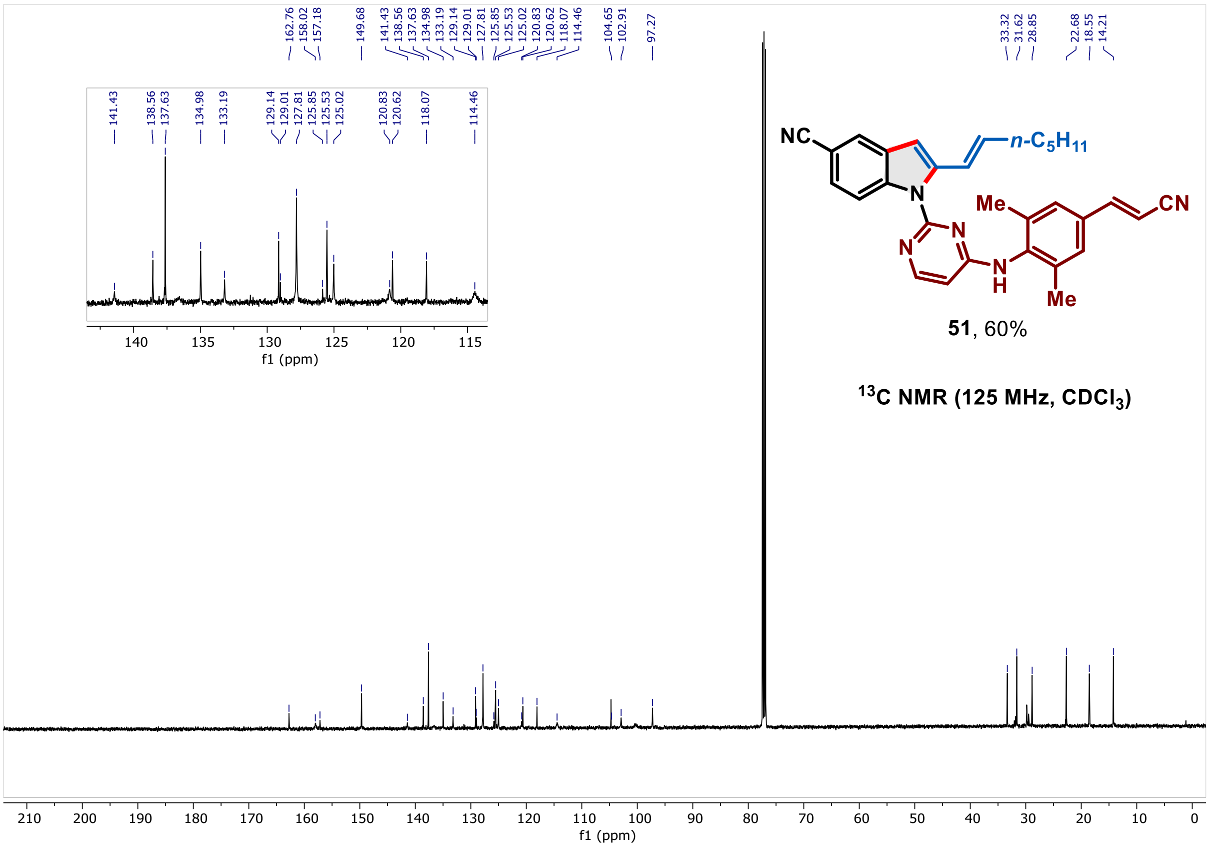
162.76 158.02 157.18 149.68 141.43 138.56 137.63 134.98 133.19 129.14 129.01 127.81 125.85 125.53 125.02 120.83 120.62 118.07 114.46 104.65 102.91 97.27 33.32 31.62 28.85 22.68 18.55 14.21
140 135 130 125 120 115
f1 (ppm)
NC
n-C5H11
N
Me
CN
N
N
N
H
Me
51, 60%
13C NMR (125 MHz, CDCl3)
210 200 190 180 170 160 150 140 130 120 110 100 90 80 70 60 50 40 30 20 10 0
f1 (ppm)

54

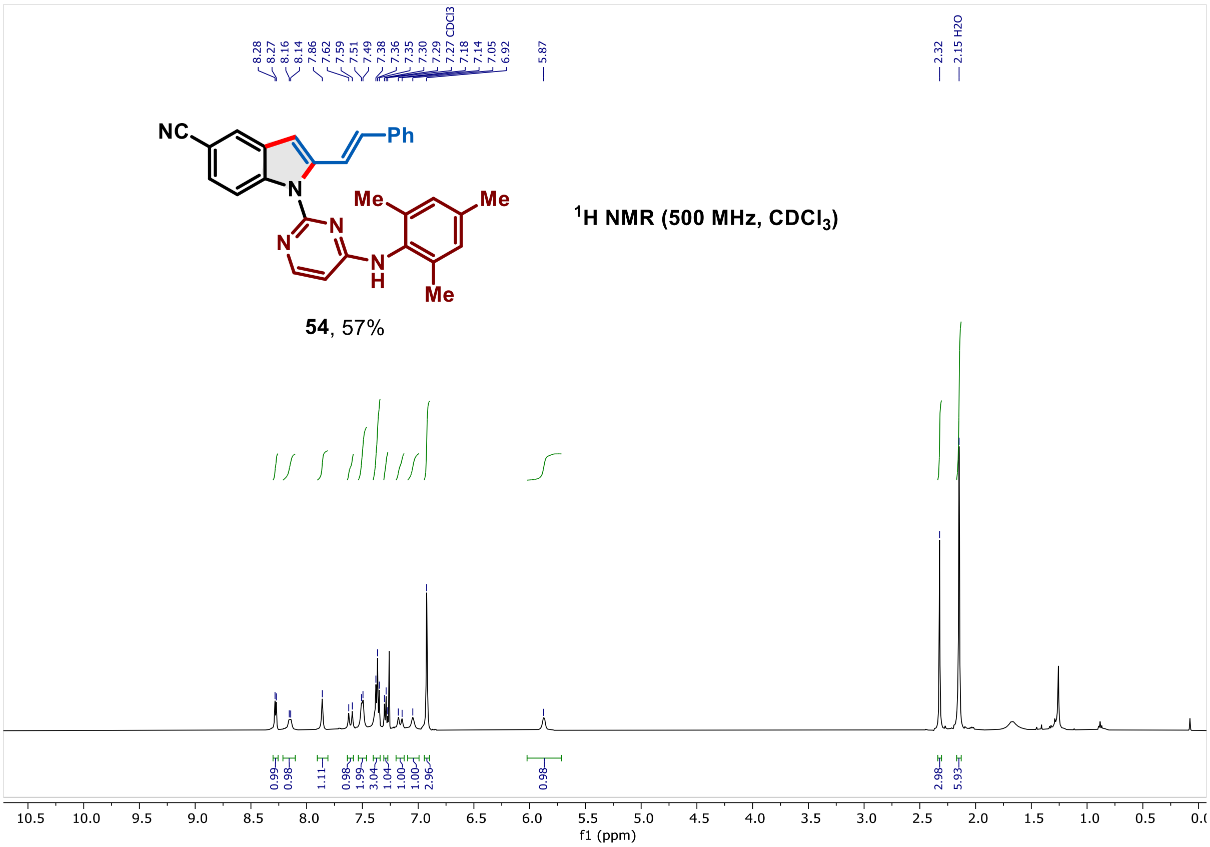
8.28
8.27
8.16
8.14
7.86
7.62
7.59
7.51
7.49
7.38
7.36
7.35
7.30
7.29
7.27 CDCl3
7.18
7.14
7.05
6.92
5.87
2.32
2.15 H2O
NC
Ph
N
Me
Me
N
N
N
H
Me
54, 57%
1H NMR (500 MHz, CDCl3)
0.99
0.98
1.11
0.98
1.99
3.04
1.04
1.00
1.00
2.96
0.98
2.98
5.93
10.5 10.0 9.5 9.0 8.5 8.0 7.5 7.0 6.5 6.0 5.5 5.0 4.5 4.0 3.5 3.0 2.5 2.0 1.5 1.0 0.5 0.0
f1 (ppm)

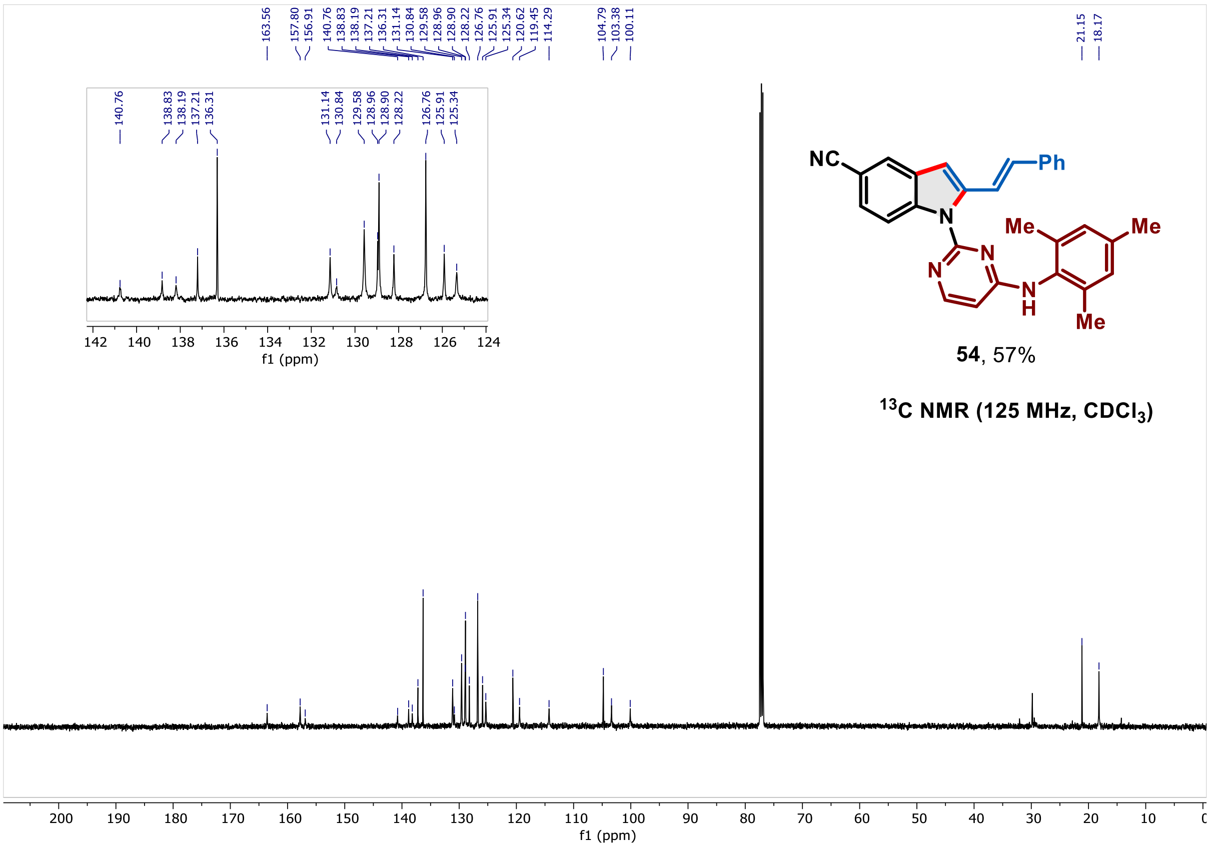
163.56
157.80
156.91
140.76
138.83
138.19
137.21
136.31
131.14
130.84
129.58
128.96
128.90
128.22
126.76
125.91
125.34
120.62
119.45
114.29
104.79
103.38
100.11
21.15
18.17
142 140 138 136 134 132 130 128 126 124
f1 (ppm)
NC
Ph
N
Me
Me
N
N
N
H
Me
54, 57%
13C NMR (125 MHz, CDCl3)
200 190 180 170 160 150 140 130 120 110 100 90 80 70 60 50 40 30 20 10 0
f1 (ppm)

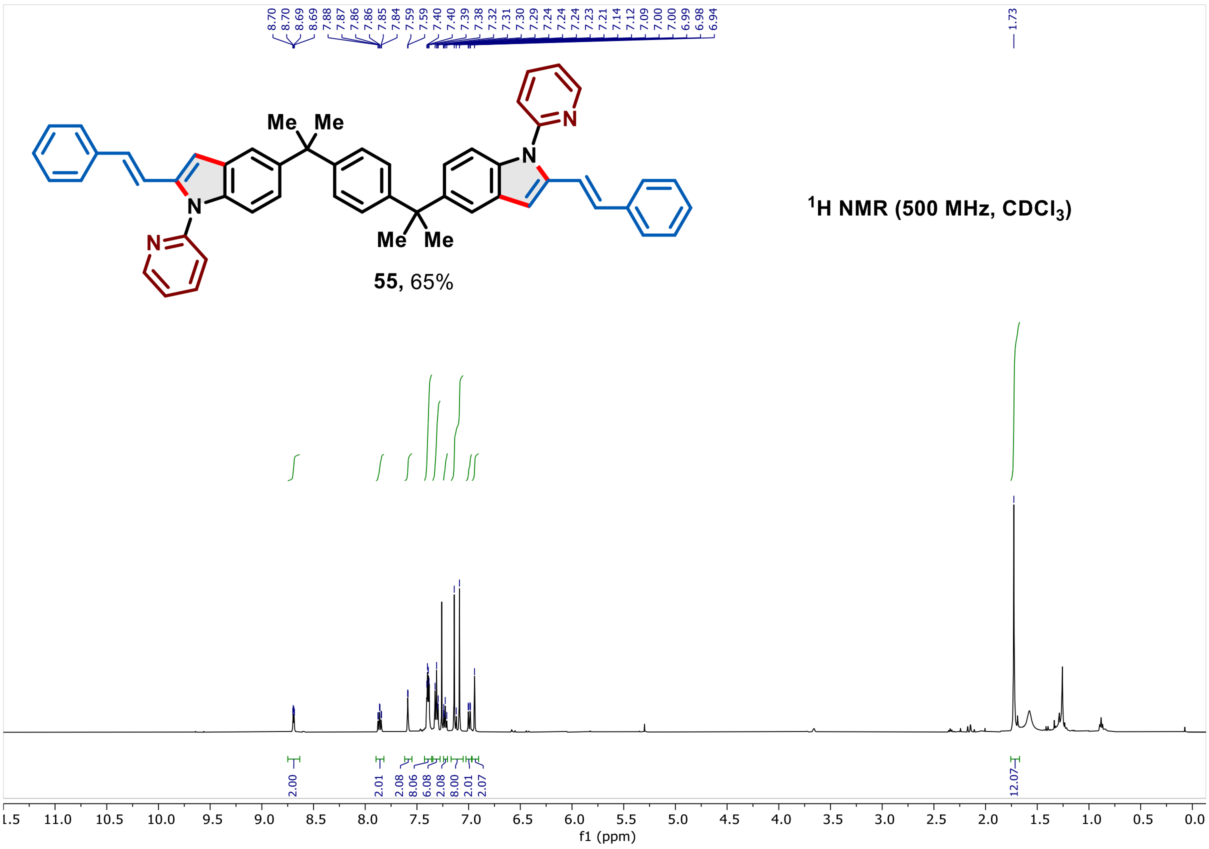

Me Me
Me Me
N
N
N
N
55, 65%
^{1}H NMR (500 MHz, CDCl$_3$)
f1 (ppm)

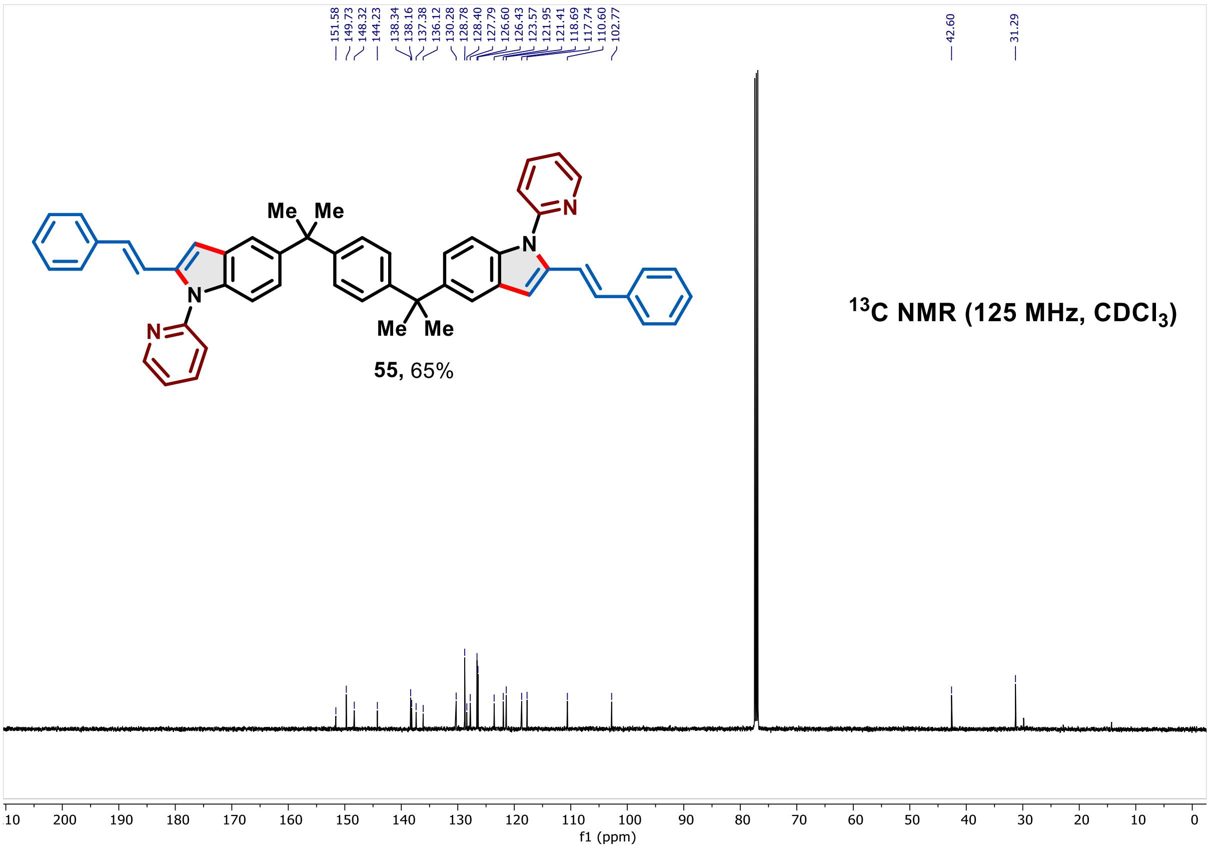

Me Me
Me Me
N
N
N
N
55, 65%
^{13}C NMR (125 MHz, CDCl$_3$)
f1 (ppm)

56

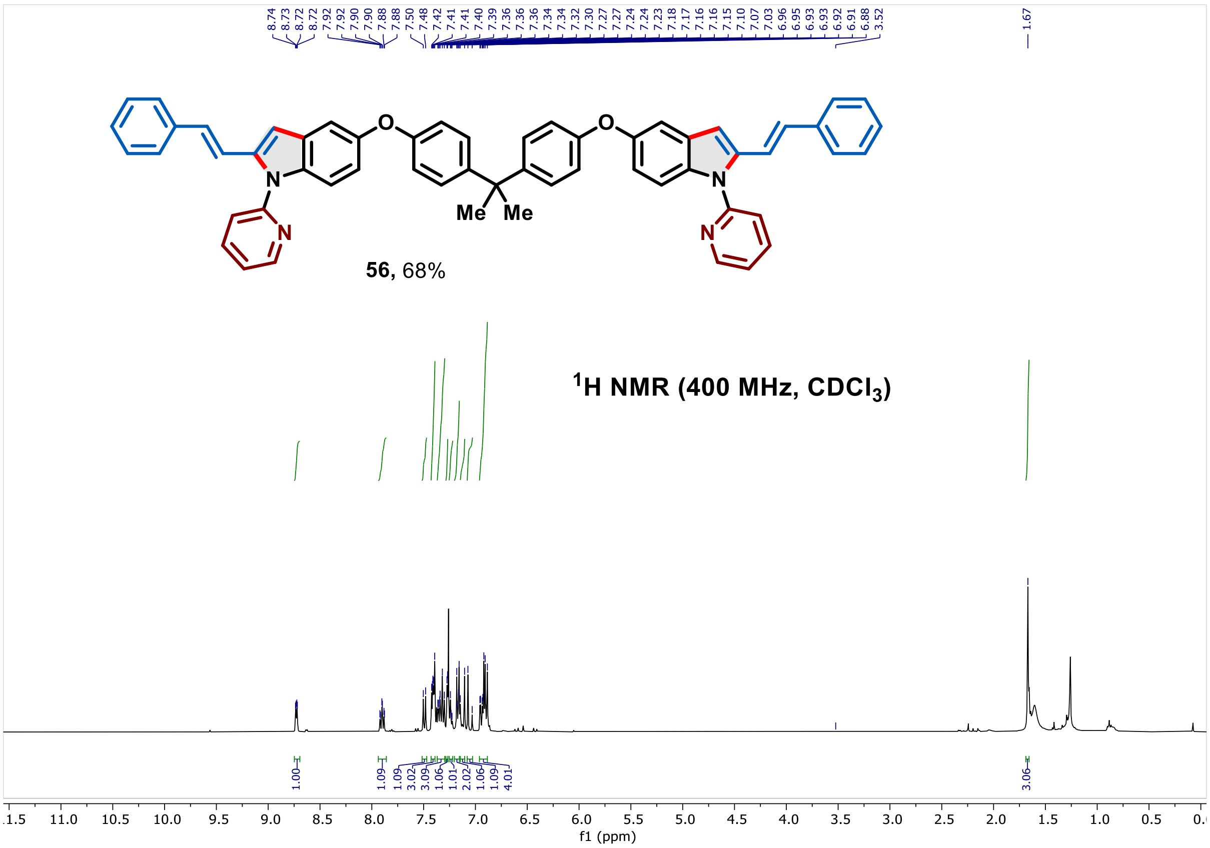

8.74 8.73 8.72 8.72 7.92 7.92 7.90 7.90 7.88 7.88 7.50 7.48 7.42 7.41 7.41 7.40 7.39 7.36 7.36 7.36 7.34 7.34 7.32 7.30 7.27 7.27 7.24 7.24 7.23 7.18 7.17 7.16 7.15 7.10 7.07 7.03 6.96 6.95 6.93 6.93 6.92 6.91 6.88 3.52
1.67
O
O
N
N
N
N
Me
Me
56, 68%
1H NMR (400 MHz, CDCl3)
1.00
1.09 1.09 3.02 3.09 1.06 1.01 2.02 1.06 1.09 4.01
3.06
f1 (ppm)

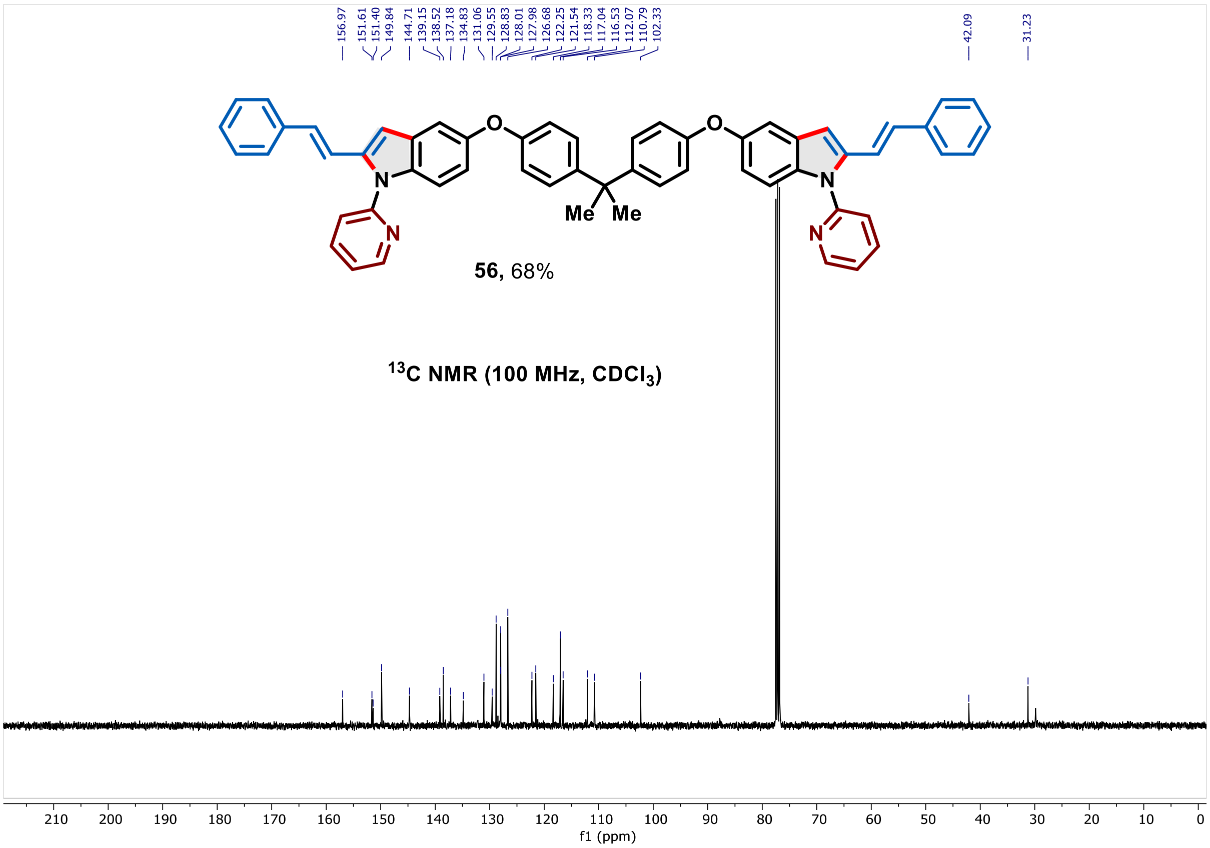

156.97 151.61 151.40 149.84 144.71 139.15 138.52 137.18 134.83 131.06 129.55 128.83 128.01 127.98 126.68 122.25 121.54 118.33 117.04 116.53 112.07 110.79 102.33
42.09
31.23
O
O
N
N
N
N
Me
Me
56, 68%
13C NMR (100 MHz, CDCl3)
f1 (ppm)

57

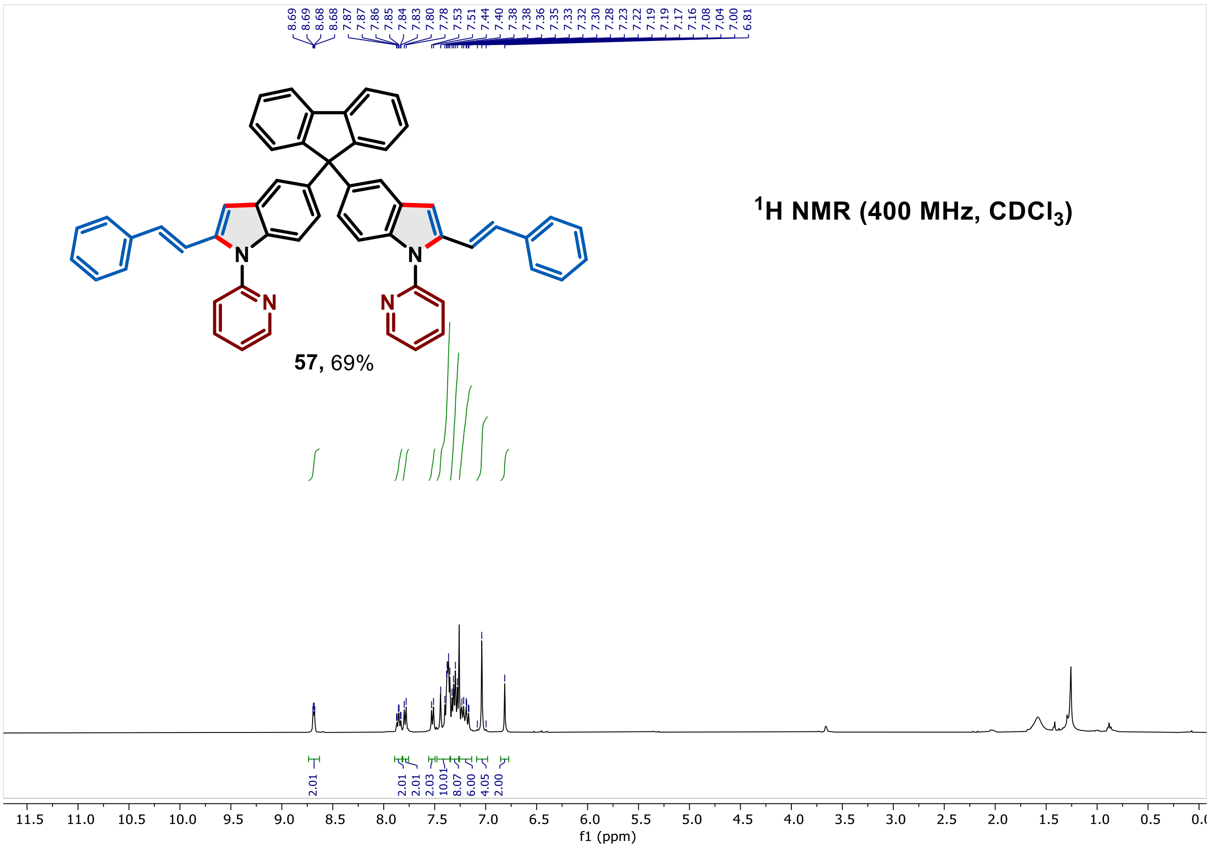
8.69 8.69 8.68 8.68 7.87 7.87 7.86 7.85 7.84 7.83 7.80 7.78 7.53 7.51 7.44 7.40 7.38 7.38 7.36 7.35 7.33 7.32 7.30 7.28 7.23 7.22 7.19 7.19 7.17 7.16 7.08 7.04 7.00 6.81
N
N
N
N
57, 69%
^{1}H NMR (400 MHz, CDCl$_3$)
2.01 2.01 2.01 2.03 10.01 8.07 6.00 4.05 2.00
f1 (ppm)

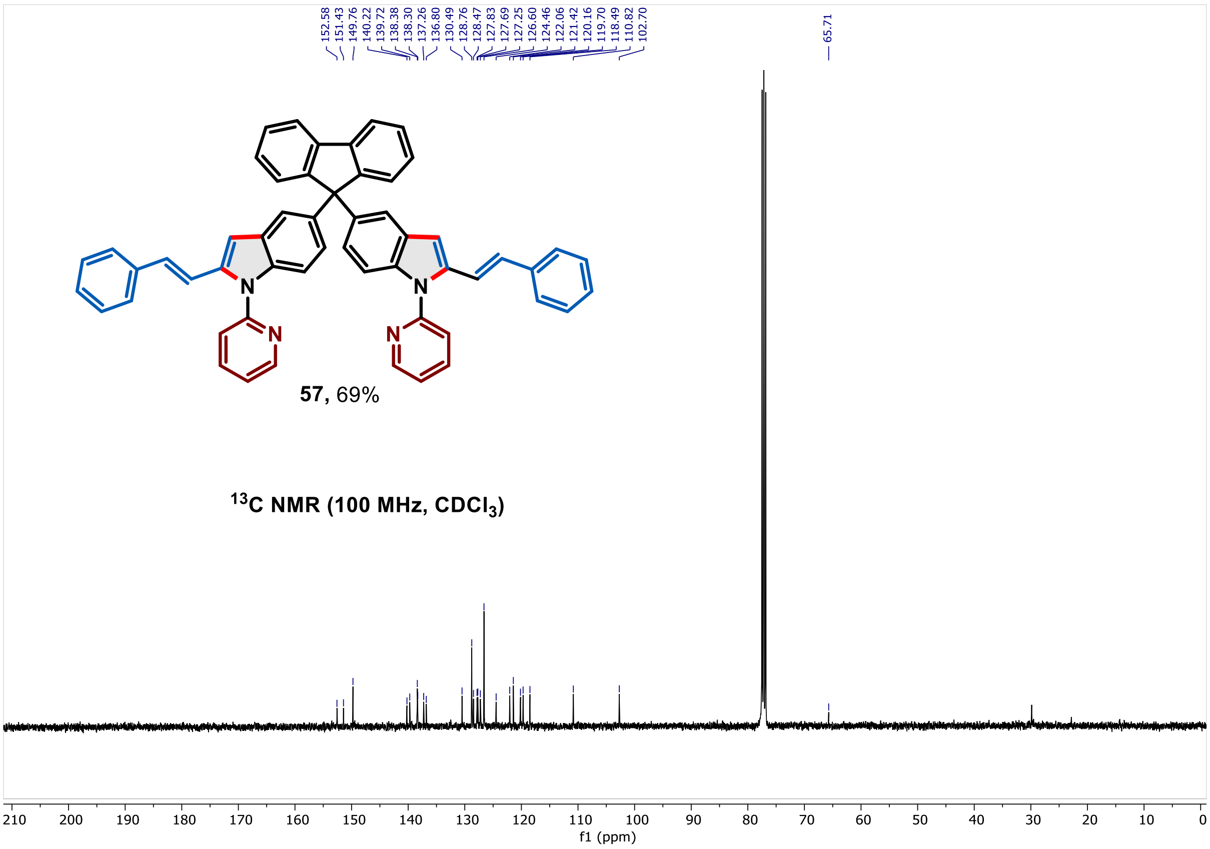
152.58 151.43 149.76 140.22 139.72 138.38 138.30 137.26 136.80 130.49 128.76 128.47 127.83 127.69 127.25 126.60 124.46 122.06 121.42 120.16 119.70 118.49 110.82 102.70
65.71
N
N
N
N
57, 69%
^{13}C NMR (100 MHz, CDCl$_3$)
f1 (ppm)

58

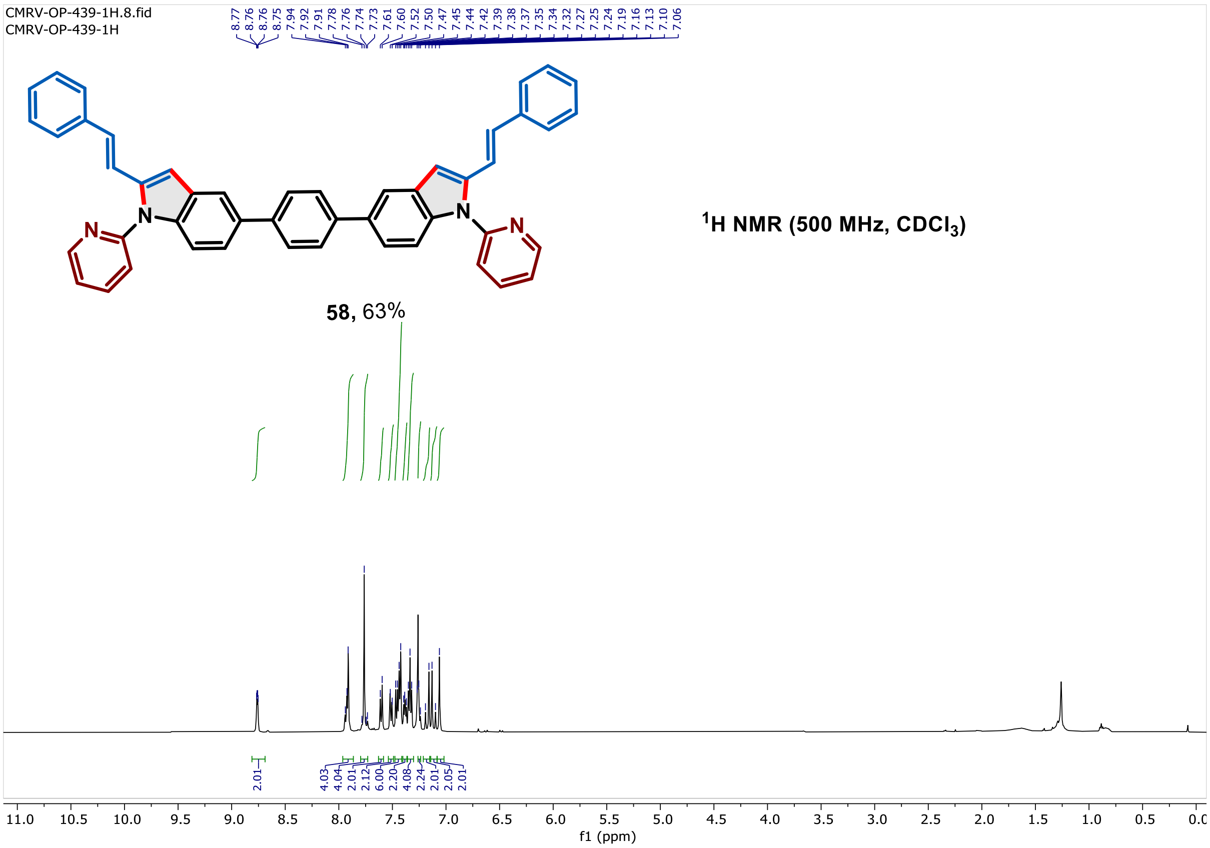
CMRV-OP-439-1H.8.fid
CMRV-OP-439-1H
8.77 8.76 8.76 8.75 7.94 7.92 7.91 7.78 7.76 7.74 7.73 7.61 7.60 7.52 7.50 7.47 7.45 7.44 7.42 7.39 7.38 7.37 7.35 7.34 7.32 7.27 7.25 7.24 7.19 7.16 7.13 7.10 7.06
N
N
N
N
58, 63%
^{1}H NMR (500 MHz, $CDCl_3$)
2.01
4.03 4.04 2.01 2.12 6.00 2.20 4.08 2.24 2.01 2.05 2.01
11.0 10.5 10.0 9.5 9.0 8.5 8.0 7.5 7.0 6.5 6.0 5.5 5.0 4.5 4.0 3.5 3.0 2.5 2.0 1.5 1.0 0.5 0.0
f1 (ppm)

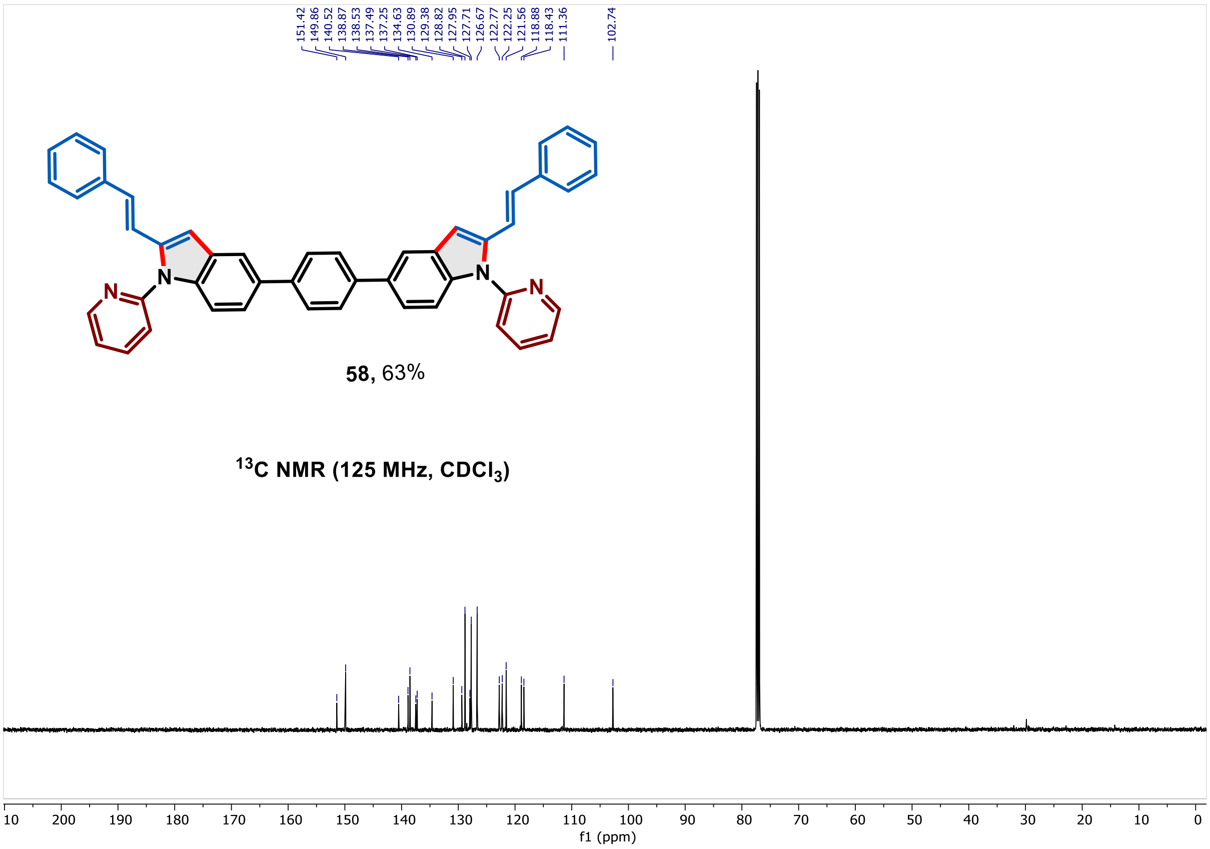
151.42 149.86 140.52 138.87 138.53 137.49 137.25 134.63 130.89 129.38 128.82 127.95 127.71 126.67 122.77 122.25 121.56 118.88 118.43 111.36 102.74
N
N
N
N
58, 63%
^{13}C NMR (125 MHz, $CDCl_3$)
10 200 190 180 170 160 150 140 130 120 110 100 90 80 70 60 50 40 30 20 10 0
f1 (ppm)

59

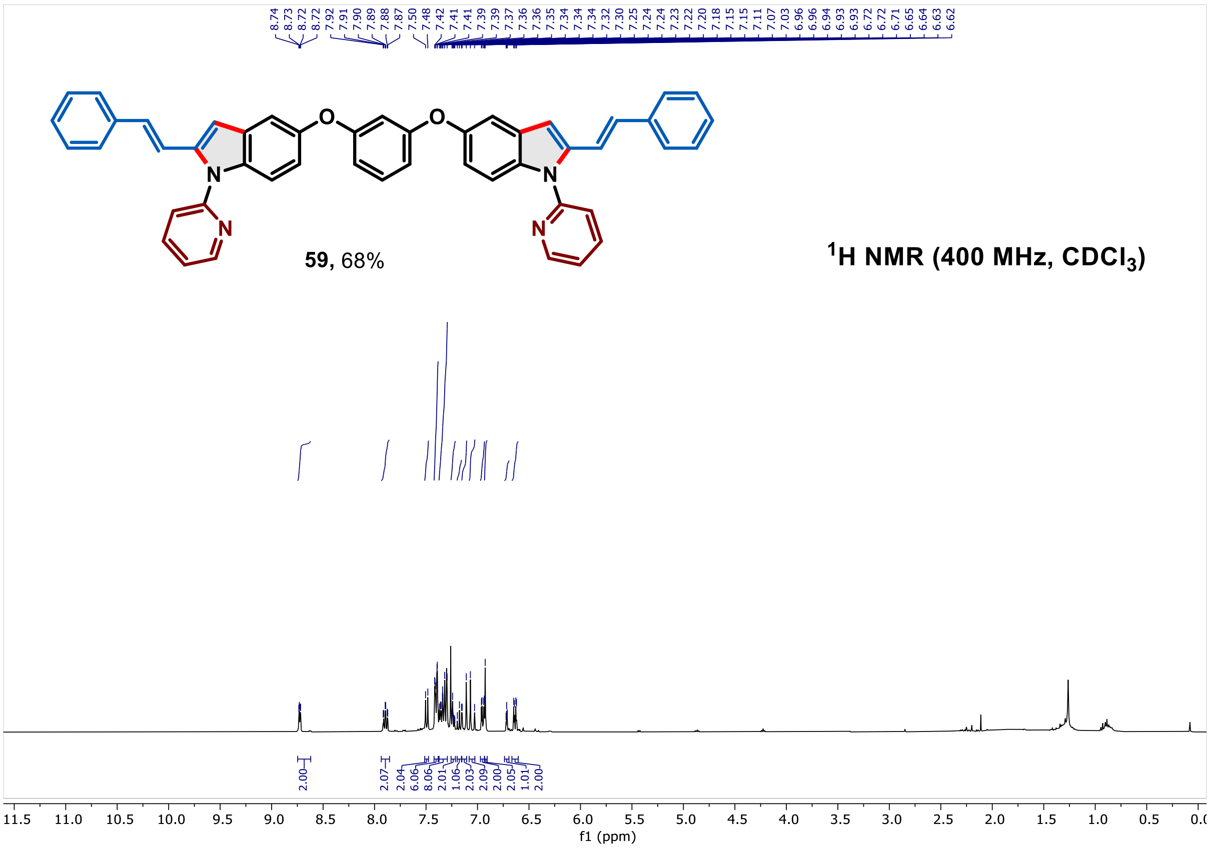
8.74 8.73 8.72 8.72 7.92 7.91 7.90 7.89 7.88 7.87 7.50 7.48 7.42 7.41 7.41 7.39 7.39 7.37 7.36 7.36 7.35 7.34 7.34 7.34 7.32 7.30 7.25 7.24 7.24 7.23 7.22 7.20 7.18 7.15 7.15 7.11 7.07 7.03 6.96 6.96 6.94 6.93 6.93 6.72 6.72 6.71 6.65 6.64 6.63 6.62
N
N
O
O
N
N
59, 68%
^{1}H NMR (400 MHz, CDCl$_3$)
2.00
2.07 2.04 6.06 8.06 2.01 1.06 2.03 2.09 2.00 2.05 1.01 2.00
11.5 11.0 10.5 10.0 9.5 9.0 8.5 8.0 7.5 7.0 6.5 6.0 5.5 5.0 4.5 4.0 3.5 3.0 2.5 2.0 1.5 1.0 0.5 0.0
f1 (ppm)

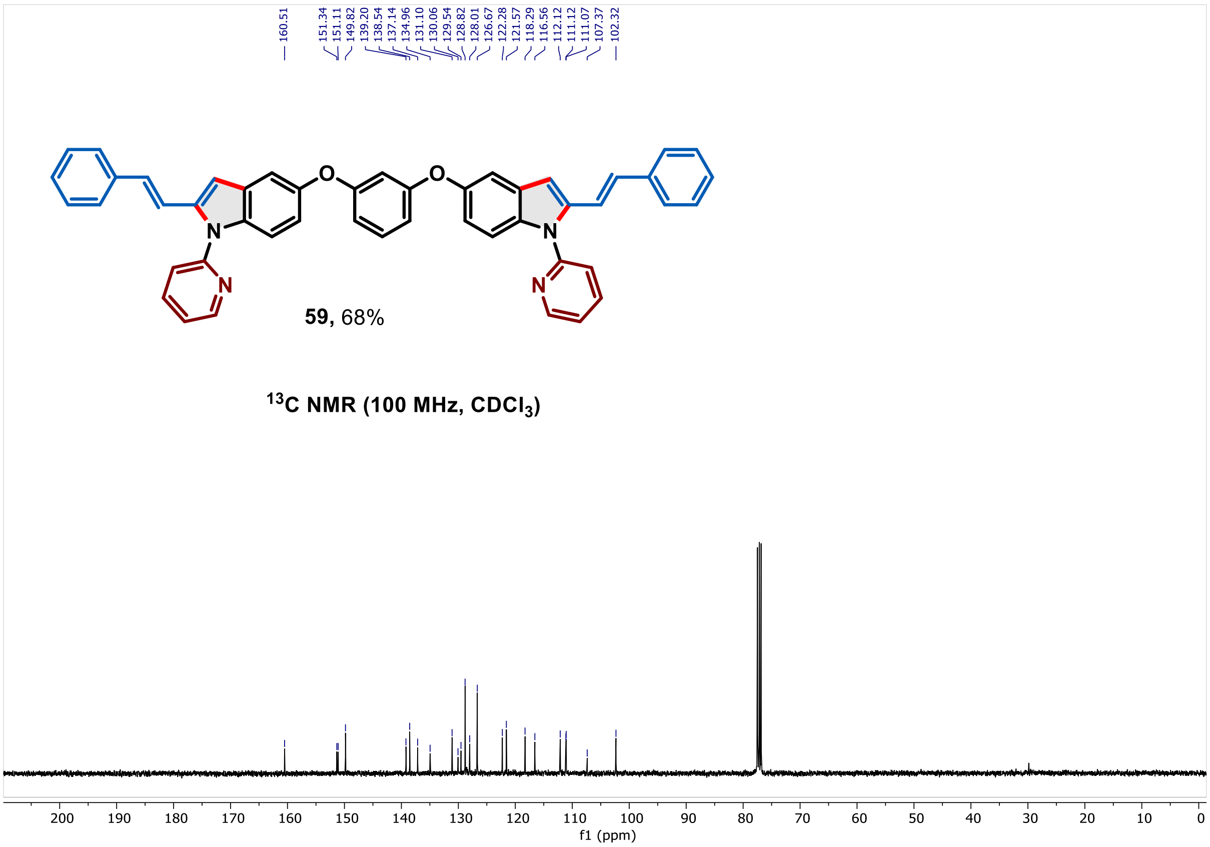
160.51
151.34 151.11 149.82 139.20 138.54 137.14 134.96 131.10 130.06 129.54 128.82 128.01 126.67 122.28 121.57 118.29 116.56 112.12 111.12 111.07 107.37 102.32
N
N
O
O
N
N
59, 68%
^{13}C NMR (100 MHz, CDCl$_3$)
200 190 180 170 160 150 140 130 120 110 100 90 80 70 60 50 40 30 20 10 0
f1 (ppm)

60

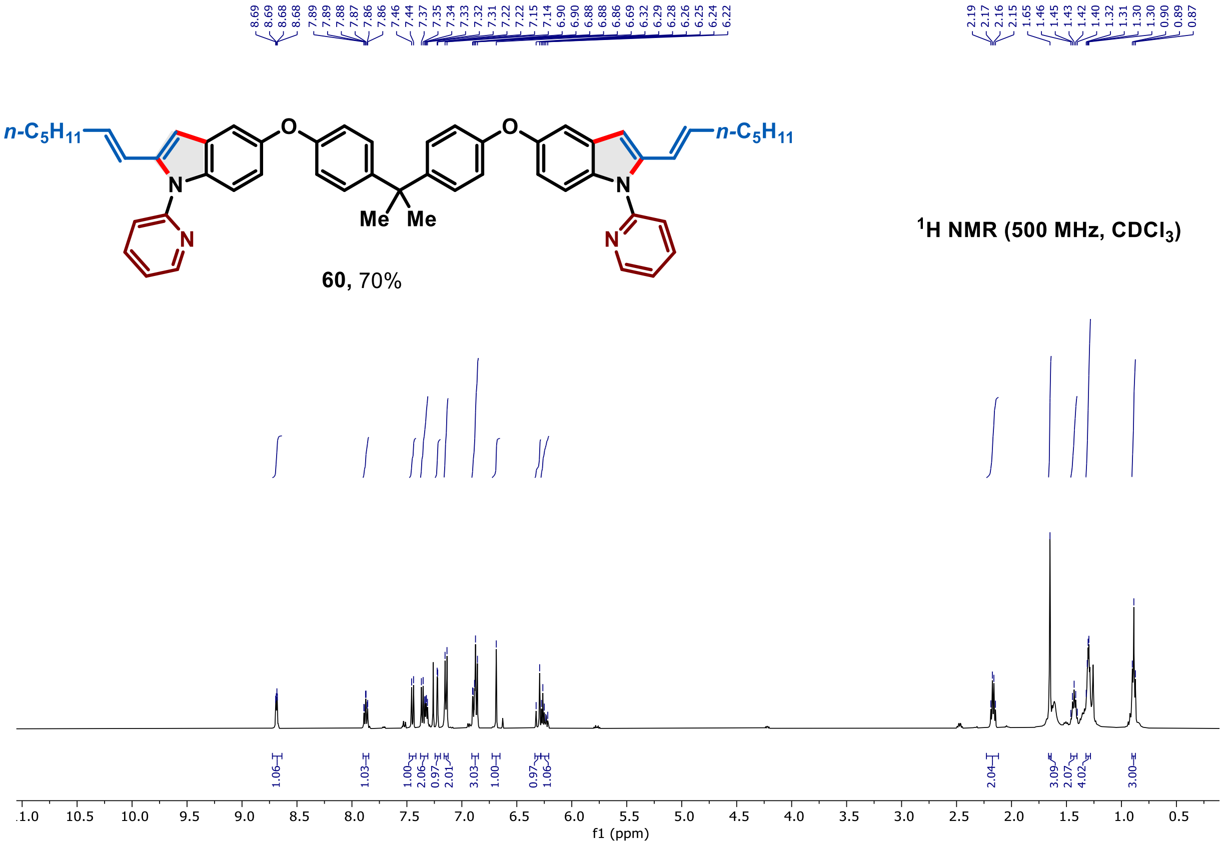

60, 70%
^{1}H NMR (500 MHz, $CDCl_3$)
f1 (ppm)

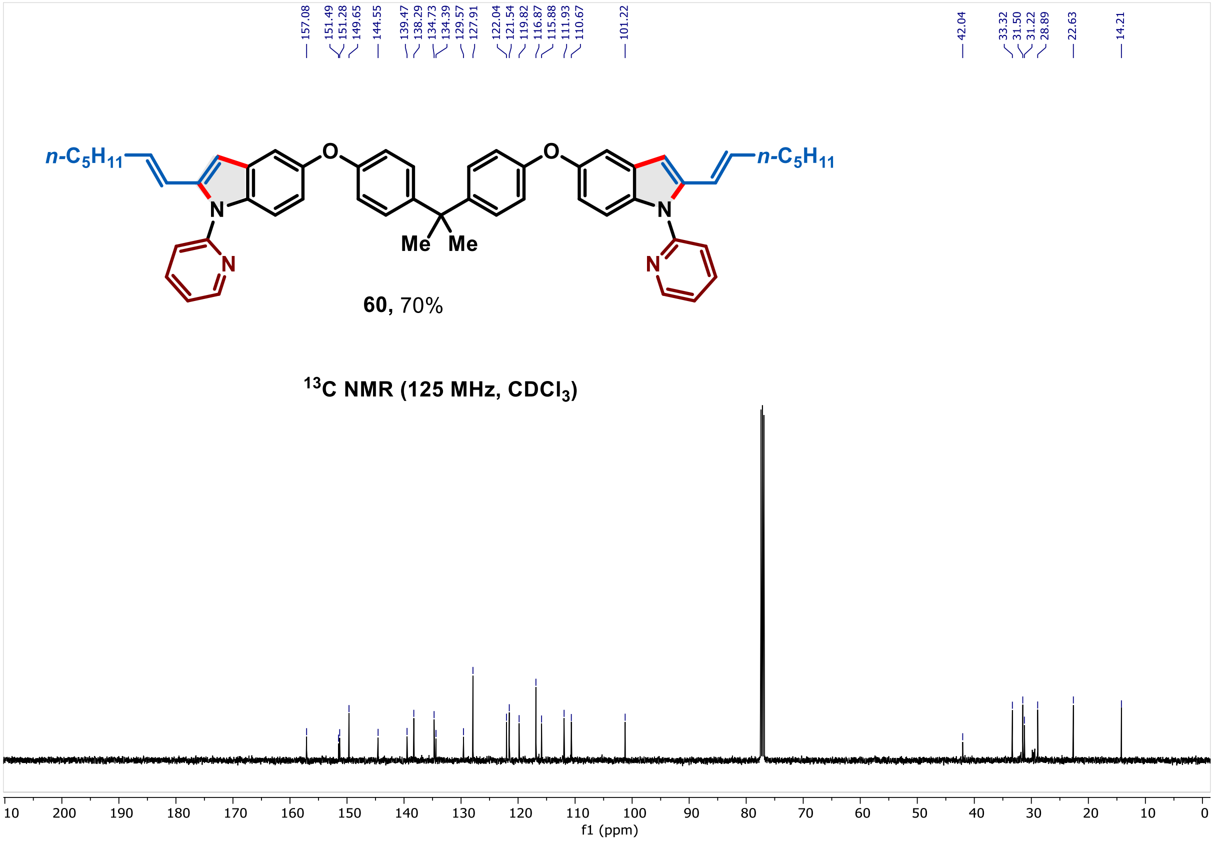

60, 70%
^{13}C NMR (125 MHz, $CDCl_3$)
f1 (ppm)

62

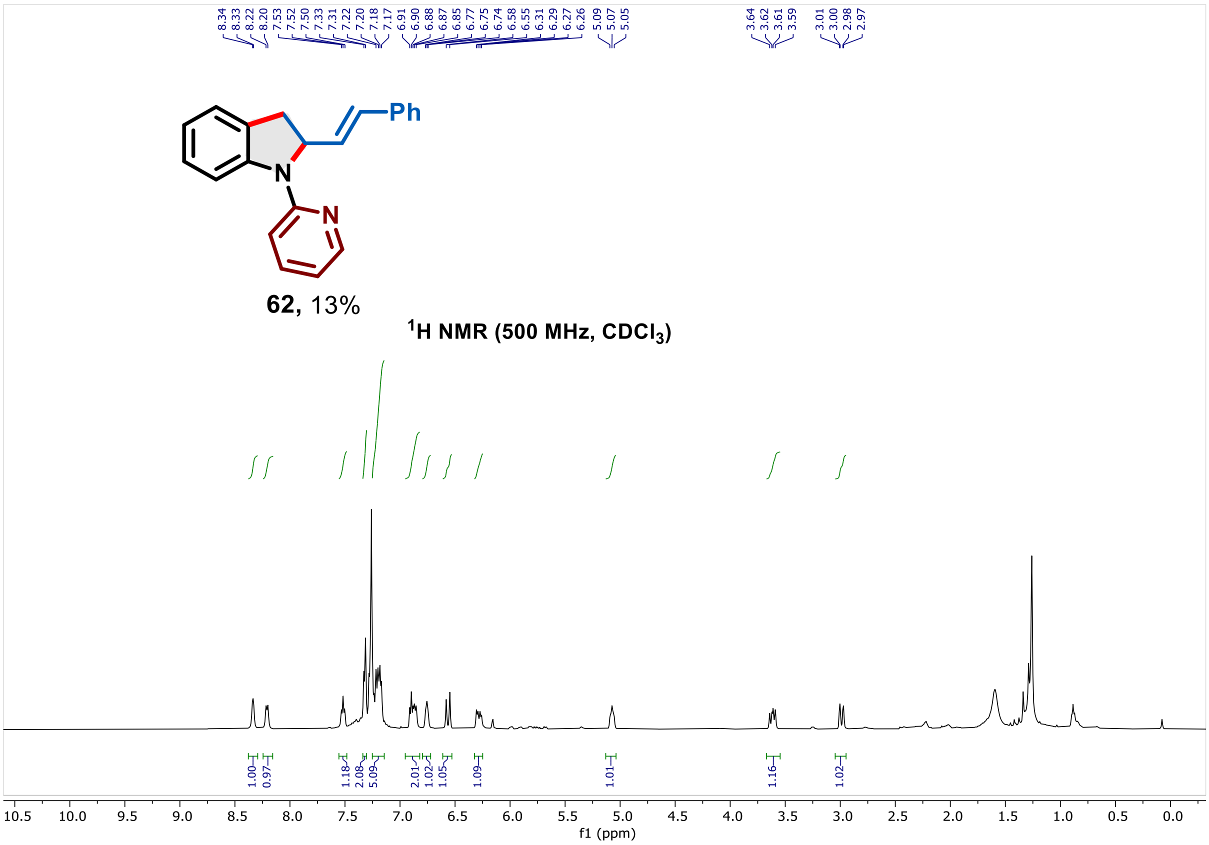
8.34 8.33 8.22 8.20 7.53 7.52 7.50 7.33 7.31 7.22 7.20 7.18 7.17 6.91 6.90 6.88 6.87 6.85 6.77 6.75 6.74 6.58 6.55 6.31 6.29 6.27 6.26 5.09 5.07 5.05
3.64 3.62 3.61 3.59 3.01 3.00 2.98 2.97
Ph
N
N
62, 13%
1H NMR (500 MHz, CDCl3)
1.00 0.97 1.18 2.08 5.09 2.01 1.02 1.05 1.09 1.01 1.16 1.02
f1 (ppm)

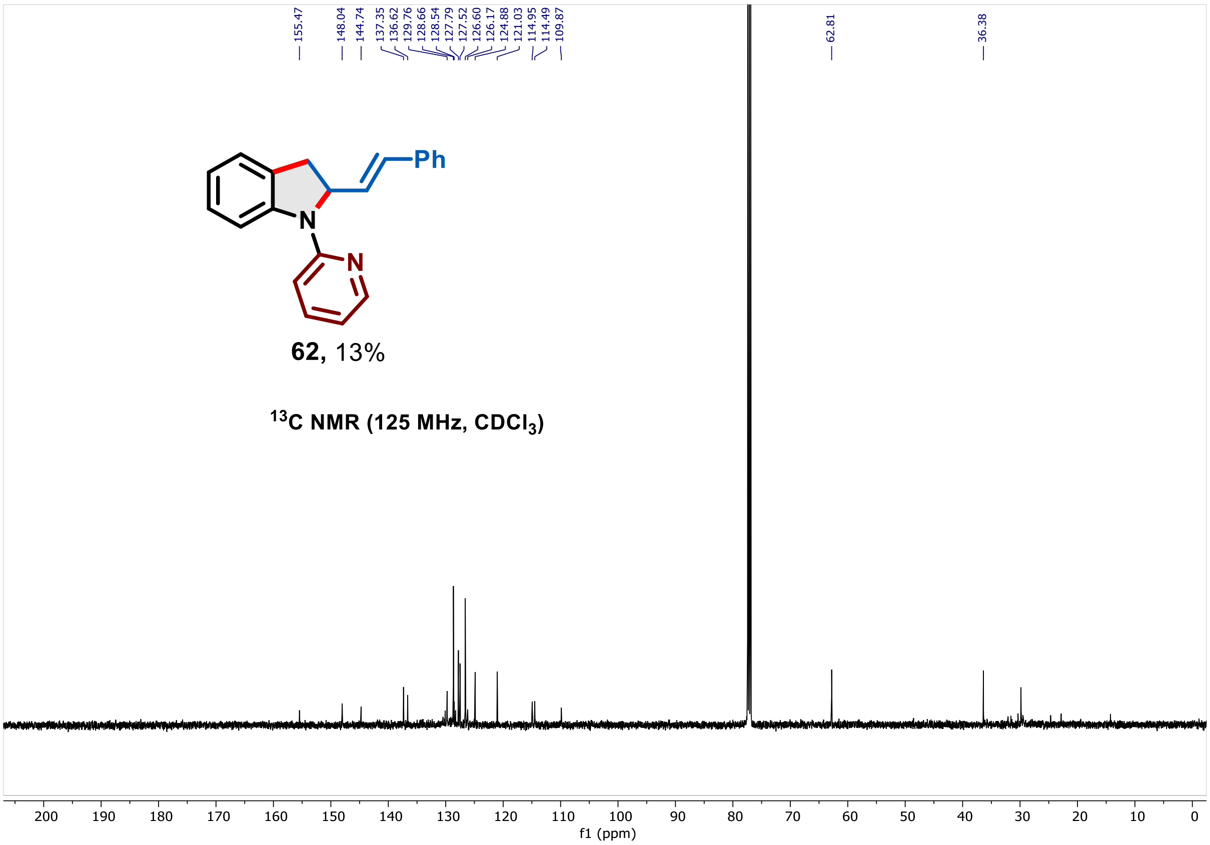
155.47 148.04 144.74 137.35 136.62 129.76 128.66 128.54 127.79 127.52 126.60 126.17 124.88 121.03 114.95 114.49 109.87
62.81
36.38
Ph
N
N
62, 13%
13C NMR (125 MHz, CDCl3)
f1 (ppm)

63

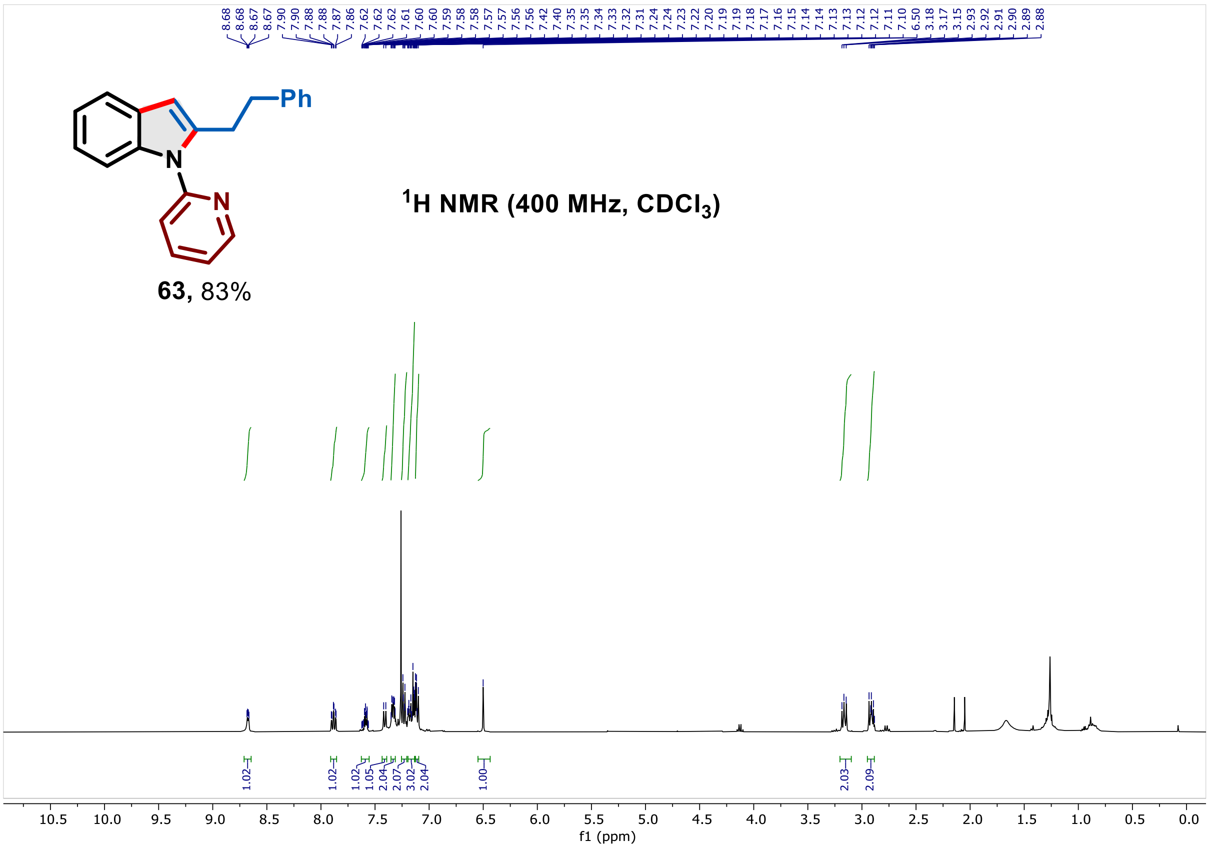

Ph
N
N
63, 83%
¹H NMR (400 MHz, CDCl3)
f1 (ppm)

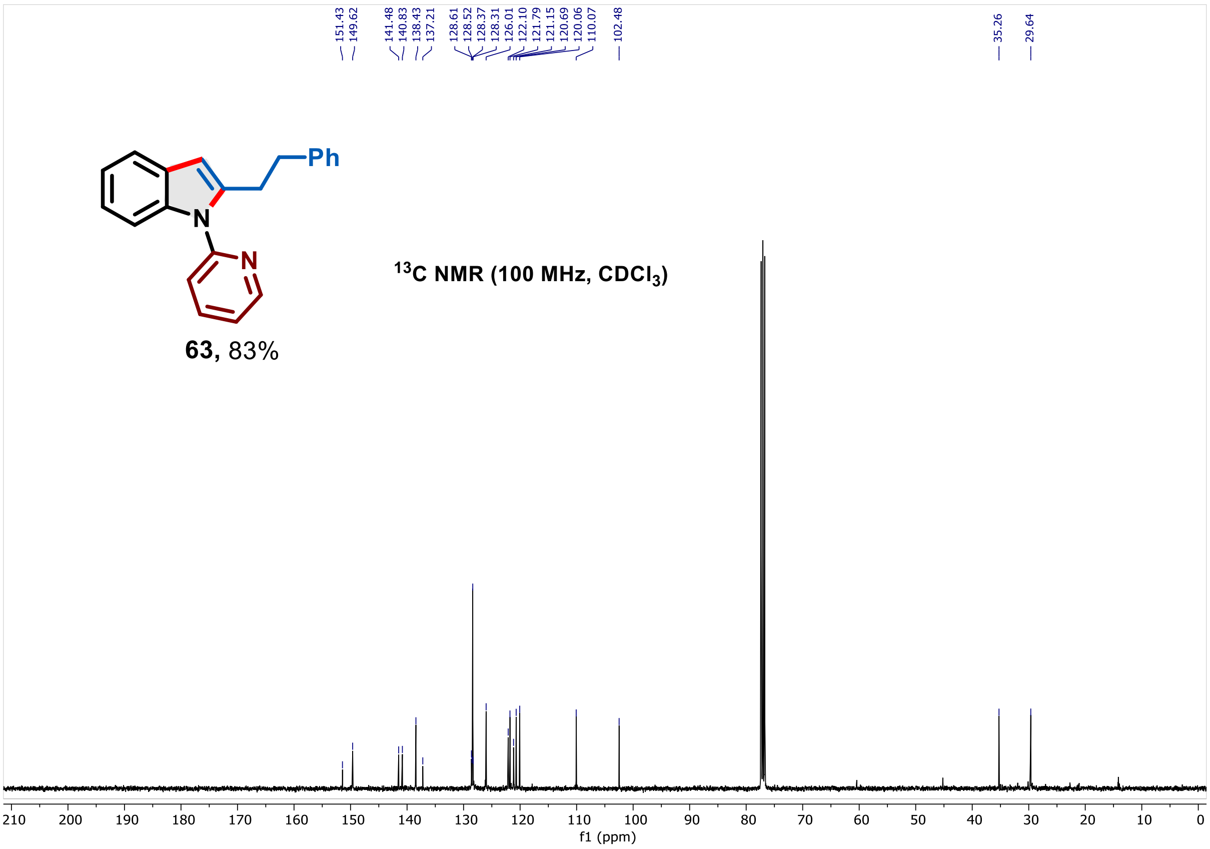

Ph
N
N
63, 83%
¹³C NMR (100 MHz, CDCl3)
f1 (ppm)

64

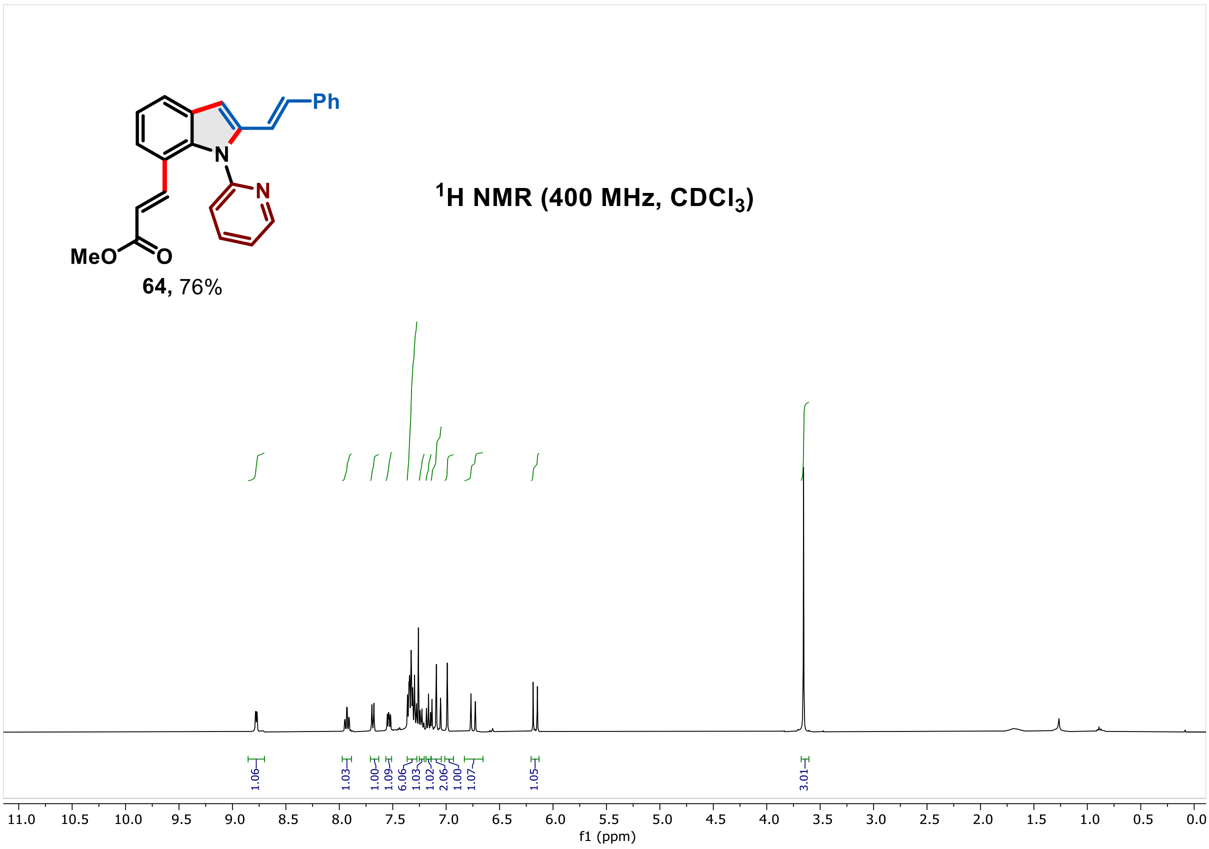
Ph
N
N
MeO
O
64, 76%
1H NMR (400 MHz, CDCl3)
1.06
1.03
1.00
1.09
6.06
1.03
1.02
2.06
1.00
1.07
1.05
3.01
11.0
10.5
10.0
9.5
9.0
8.5
8.0
7.5
7.0
6.5
6.0
5.5
5.0
4.5
4.0
3.5
3.0
2.5
2.0
1.5
1.0
0.5
0.0
f1 (ppm)

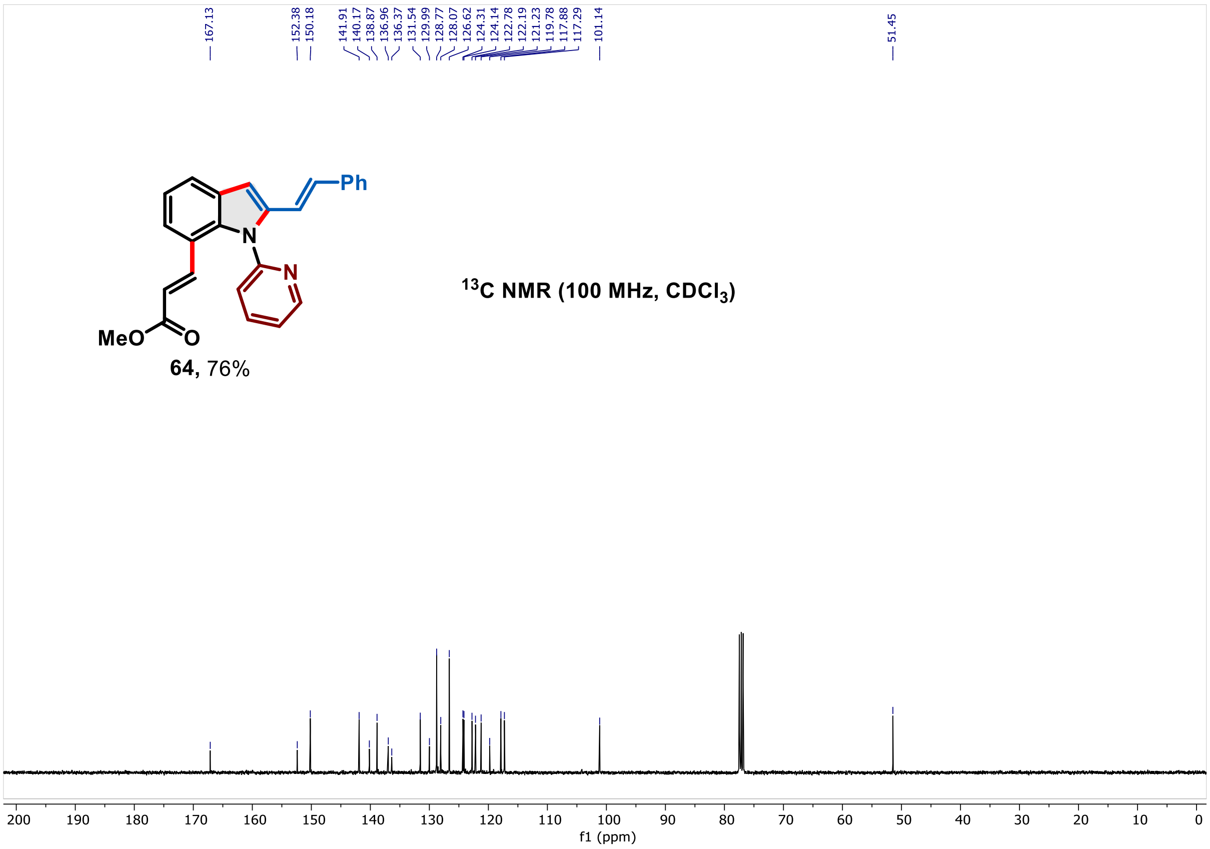
167.13
152.38
150.18
141.91
140.17
138.87
136.96
136.37
131.54
129.99
128.77
128.07
126.62
124.31
124.14
122.78
122.19
121.23
119.78
117.88
117.29
101.14
51.45
Ph
N
N
MeO
O
64, 76%
13C NMR (100 MHz, CDCl3)
200
190
180
170
160
150
140
130
120
110
100
90
80
70
60
50
40
30
20
10
0
f1 (ppm)

65

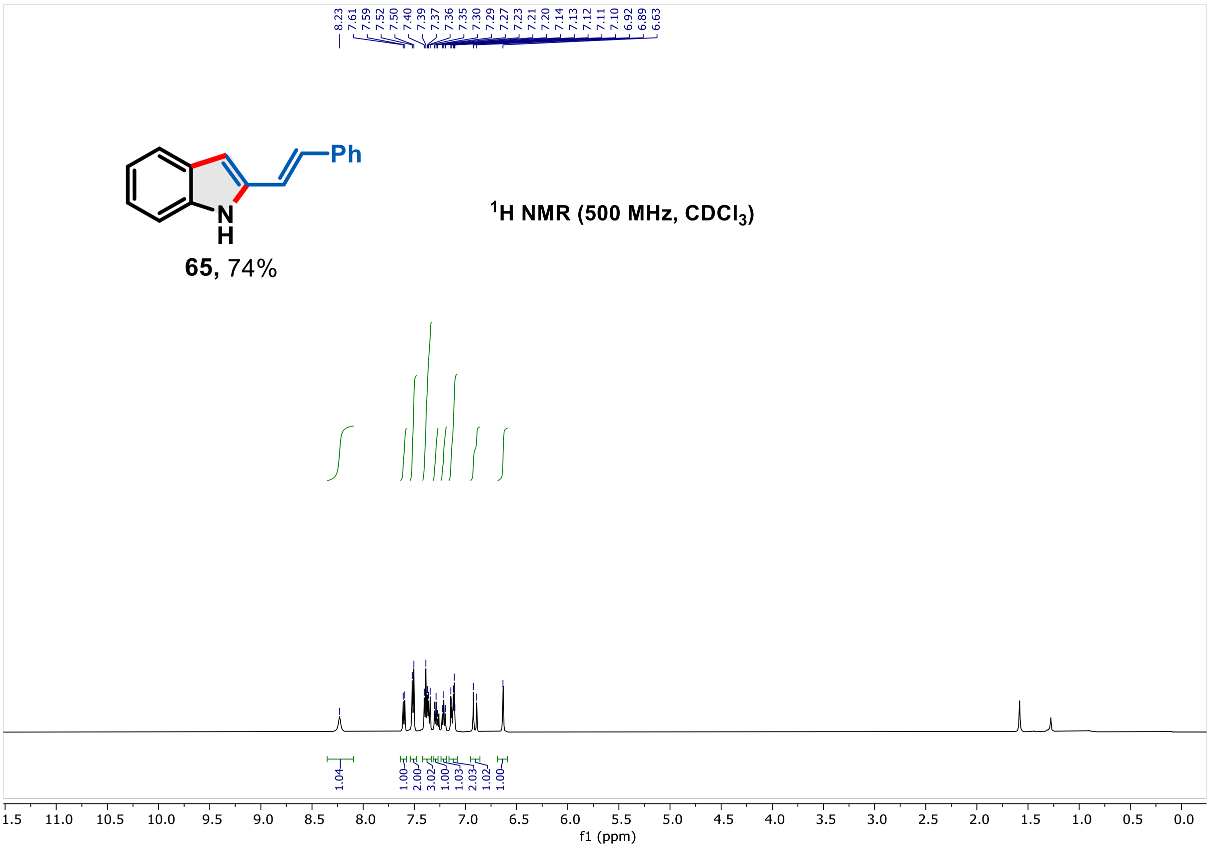
Ph
N
H
65, 74%
1H NMR (500 MHz, CDCl3)
f1 (ppm)

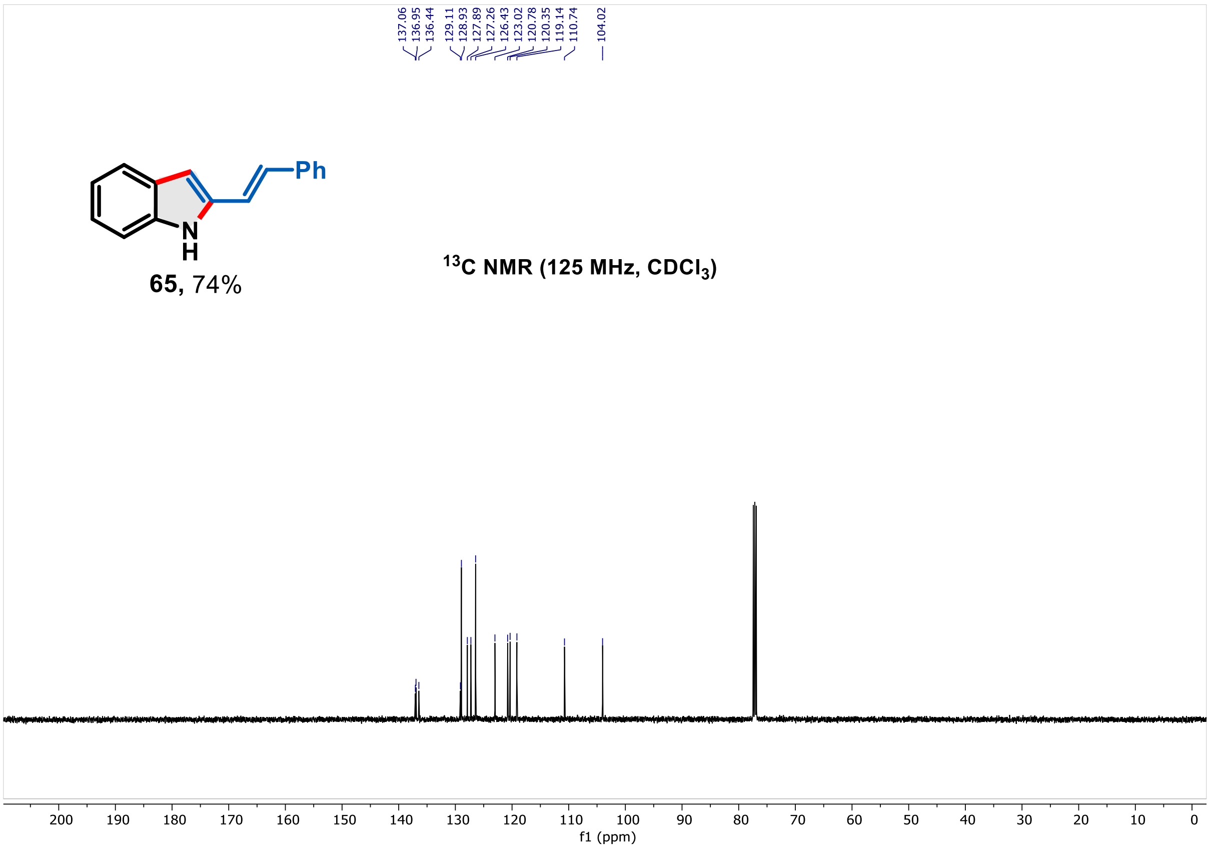
Ph
N
H
65, 74%
13C NMR (125 MHz, CDCl3)
f1 (ppm)

66

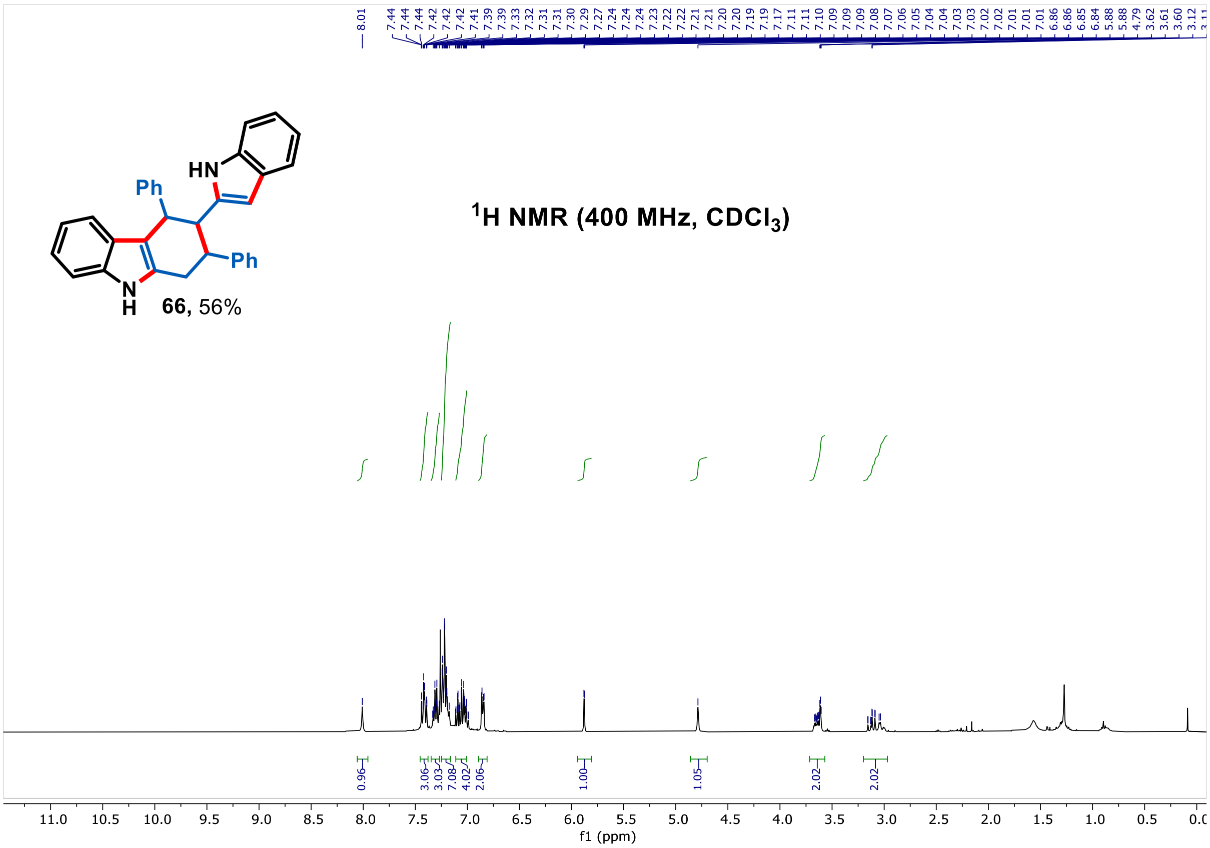

HN
Ph
Ph
N
H
66, 56%
1H NMR (400 MHz, CDCl3)
f1 (ppm)

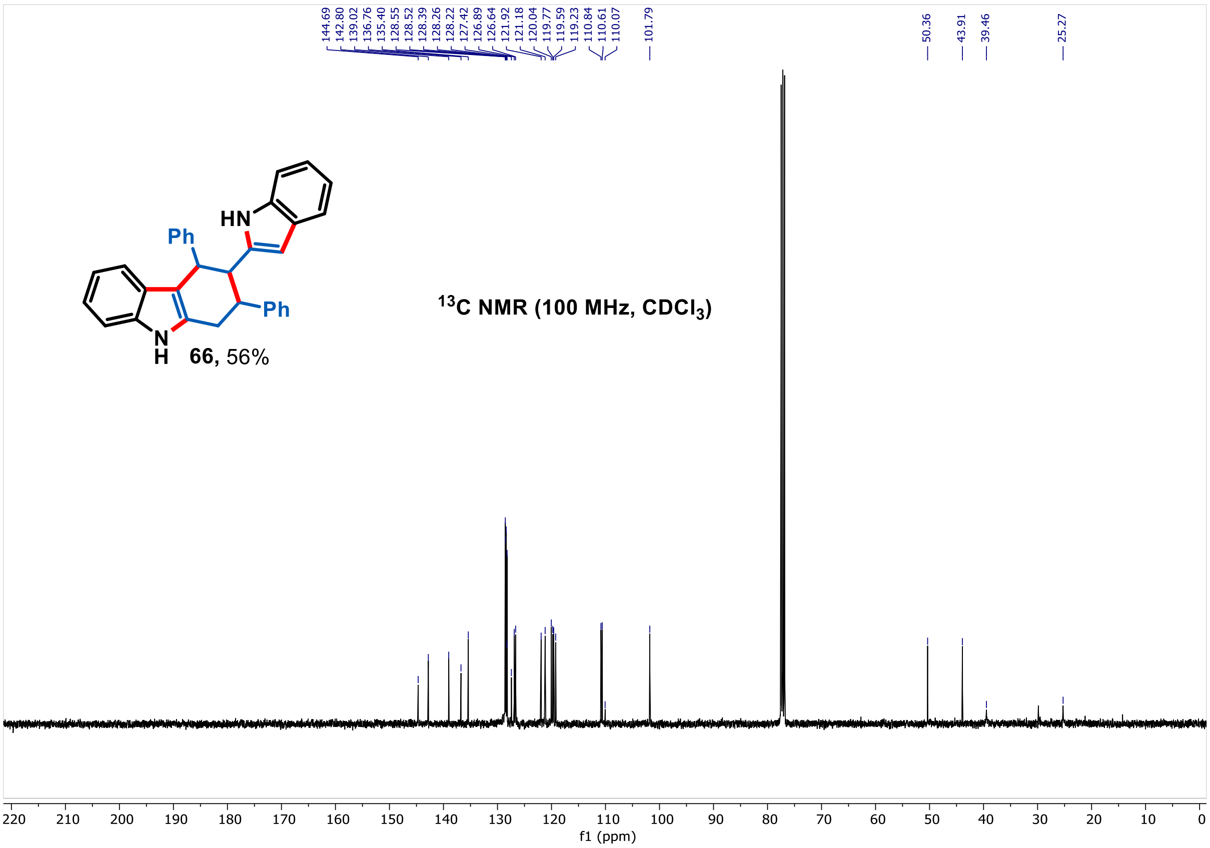

HN
Ph
Ph
N
H
66, 56%
13C NMR (100 MHz, CDCl3)
f1 (ppm)

67

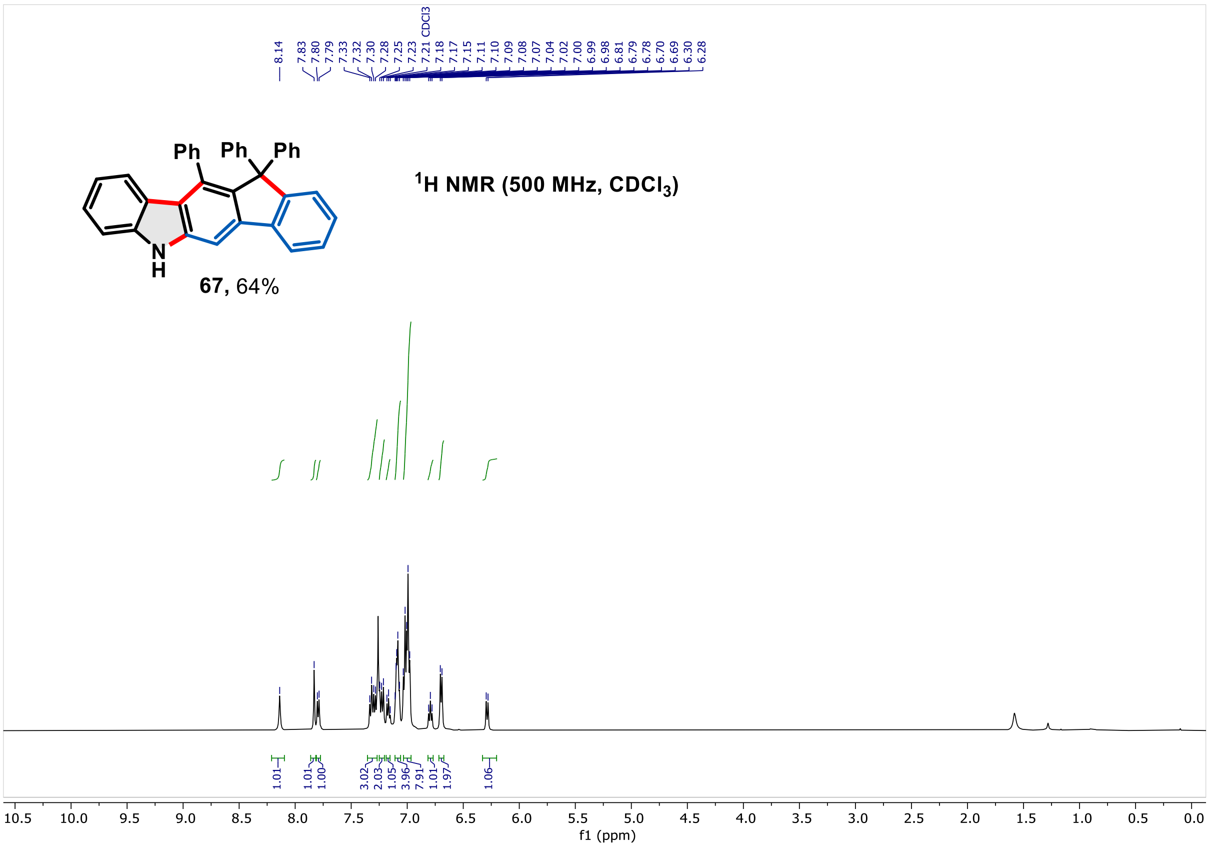

8.14 7.83 7.80 7.79 7.33 7.32 7.30 7.28 7.25 7.23 7.21 CDCl3 7.18 7.17 7.15 7.11 7.10 7.09 7.08 7.07 7.04 7.02 7.00 6.99 6.98 6.81 6.79 6.78 6.70 6.69 6.30 6.28
Ph Ph Ph
N
H
67, 64%
1H NMR (500 MHz, CDCl3)
1.01 1.01 1.00 3.02 2.03 1.05 3.96 7.91 1.01 1.97 1.06
10.5 10.0 9.5 9.0 8.5 8.0 7.5 7.0 6.5 6.0 5.5 5.0 4.5 4.0 3.5 3.0 2.5 2.0 1.5 1.0 0.5 0.0
f1 (ppm)

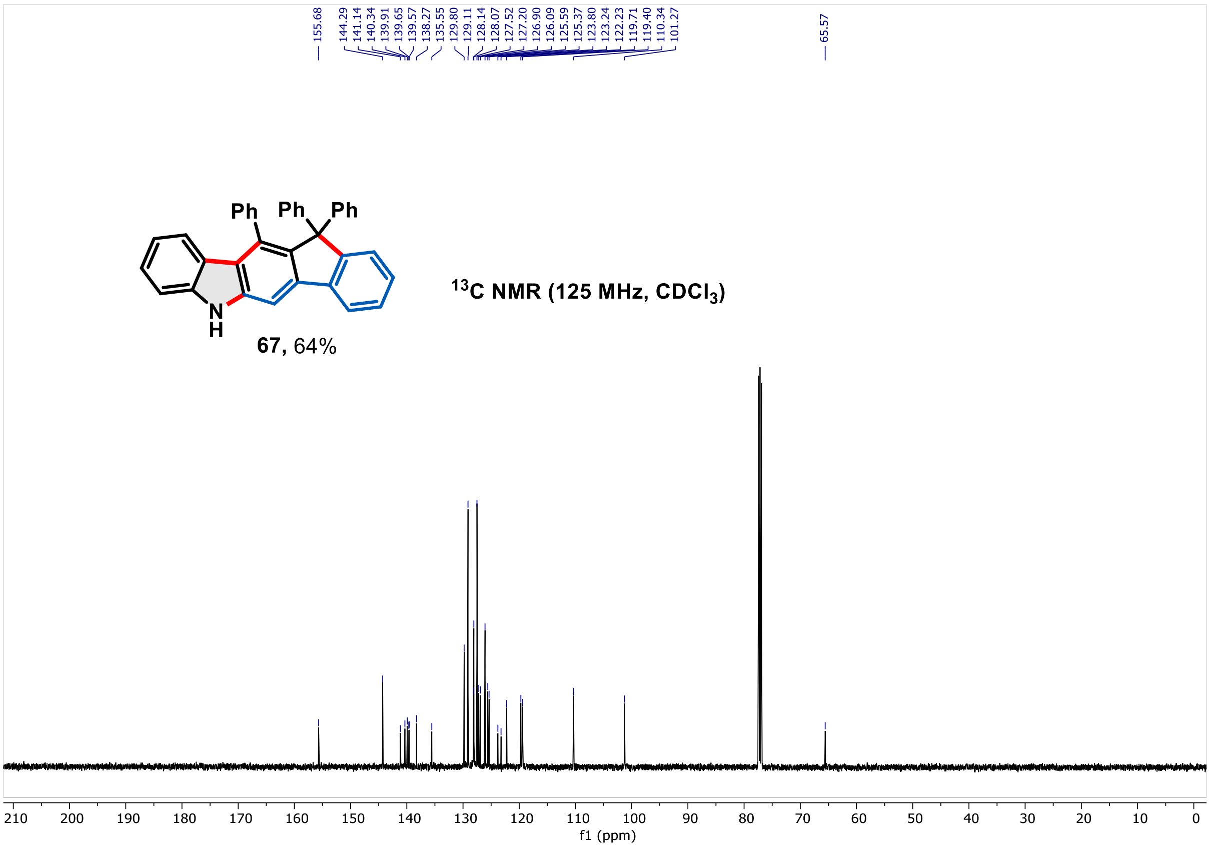

155.68 144.29 141.14 140.34 139.91 139.65 139.57 138.27 135.55 129.80 129.11 128.14 128.07 127.52 127.20 126.90 126.09 125.59 125.37 123.80 123.24 122.23 119.71 119.40 110.34 101.27
65.57
Ph Ph Ph
N
H
67, 64%
13C NMR (125 MHz, CDCl3)
210 200 190 180 170 160 150 140 130 120 110 100 90 80 70 60 50 40 30 20 10 0
f1 (ppm)
